# In-text citation’s frequencies-based recommendations of relevant research papers

**DOI:** 10.7717/peerj-cs.524

**Published:** 2021-06-04

**Authors:** Abdul Shahid, Muhammad Tanvir Afzal, Abdullah Alharbi, Hanan Aljuaid, Shaha Al-Otaibi

**Affiliations:** 1 Institute of Computing, Kohat University of Science & Technology, Kohat, Pakistan; 2 Department of Computer Science, NAMAL Institute, Mianwali, Pakistan; 3 Department of Information Technology, College of Computers and Information Technology, Taif University, Taif, Saudi Arabia; 4 Computer Sciences Department, College of Computer and Information Sciences, Princess Nourah Bint Abdulrahman University (PNU), Riyadh, Saudi Arabia; 5 Information Systems Department, College of Computer and Information Sciences, Princess Nourah Bint Abdulrahman University, Riyadh, Saudi Arabia

**Keywords:** Citations, In-text Citation, Relevant Documents, Digital Libraries

## Abstract

From the past half of a century, identification of the relevant documents is deemed an active area of research due to the rapid increase of data on the web. The traditional models to retrieve relevant documents are based on bibliographic information such as Bibliographic coupling, Co-citations, and Direct citations. However, in the recent past, the scientific community has started to employ textual features to improve existing models’ accuracy. In our previous study, we found that analysis of citations at a deep level (i.e., content level) can play a paramount role in finding more relevant documents than surface level (i.e., just bibliography details). We found that cited and citing papers have a high degree of relevancy when in-text citations frequency of the cited paper is more than five times in the citing paper’s text. This paper is an extension of our previous study in terms of its evaluation of a comprehensive dataset. Moreover, the study results are also compared with other state-of-the-art approaches i.e., content, metadata, and bibliography. For evaluation, a user study is conducted on selected papers from 1,200 documents (comprise about 16,000 references) of an online journal, Journal of Computer Science (J.UCS). The evaluation results indicate that in-text citation frequency has attained higher precision in finding relevant papers than other state-of-the-art techniques such as content, bibliographic coupling, and metadata-based techniques. The use of in-text citation may help in enhancing the quality of existing information systems and digital libraries. Further, more sophisticated measure may be redefined be considering the use of in-text citations.

## Introduction

The scientific data is increasing at a rapid pace. According to Jinha, more than 50 million journal papers and billions of conference papers have been published. Further, 1.3 billion books have been digitized by Google (Jinha, 2010). There are different digital repositories such as Web of Science, SCOPUS, and PubMed for indexing these documents. For example, PubMed has indexed 27.5 million records, representing approximately 7k journals (Funk et al., 2017).

Similarly, SCOPUS indexes and Google Scholar have indexed millions and billions of documents, respectively (Scopus, 2021; Gusenbauer, 2019). Thus, identifying important research papers from such a huge repository is a challenging task. Generally, thousands of papers are returned from these systems for a user query. Thus, finding relevant research papers from the available huge size of information becomes harder day by day. To find relevant research papers, most of the scientific community relies on bibliographic information models. The widely known retrieval models that utilize citation information are bibliographic coupling, co-citations, and direct citations. These models do not use textual information. In the recent past, the scientific community has realized that significant improvements in the traditional approaches may be achieved with textual information from scientific articles. The reasons are multifaceted, such as the availability of full-text and advancement in text processing technologies. This helped the users to figure out a solution to the well-known problem of considering all citations equally. The in-text citation frequencies have been reported as key attributes in discovering the important/influential citations (Shahid et al., 2020; Shahid et al., 2021; Zhu et al., 2015). Apart from this, various research studies have reported the importance of in-text citations for various purposes. For example, (Kevin et al., 2018) have exploited the text of PubMed Central Open Access subset and Elsevier journals and reported on various characteristics in a detailed manner. For example, average numbers of sentences per document, percentages of sentences containing mentions, and references per sentence, etc. Similarly, in-text frequency-weighted citation counting methods have been used to evaluate authors’ citation impact (Zhao & Andreas, 2016).

Recently, researchers have analyzed the citing sentences to apprehend the reasons for citation (Muhammad et al., 2021). The same was suggested earlier by Teufel and Kaplan. They had proposed automatic processing of citation context of the cited paper to find the most relevant documents (Simone, Siddharthan & Tidhar, 2006; Dain, Iida & Tokunaga, 2009). Moreover, citation proximity, citation order analysis, and bytecode usage of in-text citations of the cited papers in the citing paper have also been proposed recently to identify relevant documents (Khan, Shahid & Afzal, 2018; Mehmood et al., 2019; Raja & Afzal, 2019; Boyack, Small & Klavans, 2013).

In our previous work, we conducted a study that revealed in-text citation frequencies of the cited paper hold the potential to determine the relevant papers. Our work can be considered as an extension of the direct citations model (Shahid, Afzal & Qadir, 2011). In our proposed approach, the direct citations are further traced out in the citing papers’ body text. It was identified that the more cited paper is referred to in citing paper’s body text, the more it has relevance with the citing paper. Hence, in-text citation frequencies play a vital role in the identification of relevant papers.

This paper presented a detailed user study to evaluate the in-text citation role in finding relevant papers. Unfortunately, there is no standard benchmark dataset in the literature based on which such sort of study can be evaluated. In such a scenario, one of the best alternatives could be the expert’s evaluation. Therefore, this study harnesses the expert opinion to evaluate the relevance between cited and cited-by documents.

The experiments are conducted on the dataset of 1,200 documents (containing about 16,000 references) of an online journal, the Journal of Universal Computer Science (J.UCS). Computer science applications can be seen in almost all disciplines and breakthrough discoveries are made by combining contributions with other fields like Bioinformatics, Geoinformatics, and Data Science, etc. Thus, selecting a Computer Science field in the first experiment could help comprehensively evaluate the proposed approach as authors of different domains are contributing to Computer Science. Thus, it makes a diversified citation pattern as authors are coming from diversified domains and geographical locations. The proposed system’s evaluation is performed into two dimensions: (1) system accuracy—in-text citation’s frequencies computation, and (2) in terms of relevant document identifications. The in-text citation accuracy of the extracted frequencies is 78%, which was obtained through manual verification. For the second type of evaluation of the proposed system, we conducted a user study to formulate a gold standard dataset. Later, several experiments were performed (i.e., content, metadata, and citations) to acquire the most relevant documents. The relevant documents were also identified with the help of In-text citation frequencies. Finally, the top five recommendations produced by different state-of-the-art techniques were compared with user study-based recommendations (i.e., gold standard). The outcomes indicated that in-text citation frequencies have a higher precision in identifying the most relevant documents, i.e., 0.96. The other state-of-the-art techniques like content, bibliography, and metadata have relatively low precision of 0.76, 0.56, and 0.48, respectively. Thus, augmenting in-text citations can significantly increase the usage of state-of-the-art techniques.

The rest of the paper is organized as follows as in ‘Related Work’; we presented the area’s related work. In ‘Proposed Technique’, we have presented the working methodology of this research. In ‘System Evaluation’, the system evaluation is presented. In ‘Discussion’, the results and limitations of the study are presented. Finally, in ‘Conclusion’, the study has been concluded.

## Related Work

The growth of scientific publications is exponential. In the mid of the last century, there were around 60K journals articles, and Larsen and Ins estimated that there would be 1 million (Peder & Von Ins, 2010). Further, focusing on the British Library Lending Division’s indexed data, we found that 43K journals were indexed in 1982. Referring to some recent digital libraries, for example, PLOS, which was started in 2006, has published 10K articles in just four years. Similarly, we can see the scientifically acknowledged systems such as the ISI Web of Knowledge (http://www.webofknowledge.com) , Google Scholar (http://scholar.google.com.pk/) and CiteSeer (http://citeseer.ist.psu.edu/) have also indexed large sets of information. To get an idea, we performed a search query for the term “page rank” over Google Scholar. It returned 3,020,000 papers. And the citations of the very first article in the results are 14,767. Thus, it is almost impossible for a user to comprehend this exhaustively. It means there is a need to have a system that can produce better or more refined results.

The current state-of-the-art system employs multiple approaches to address this issue. These approaches can be summarized as (1) content, (2) metadata, (3) collaborative filtering, and (4) citation-based approaches. This paper has performed experiments based on different such data sources (i.e., content, metadata, and citations). Therefore, the contemporary approaches based on these data sources are discussed below.

In content-based approaches, the contents of the papers are used to compute the similarities between the papers. It is not feasible to use the raw content of the paper. Thus first, the content is processed to find out the important terms of the paper. For this task, different techniques have been proposed, such as Term Frequency-Inverse Document Frequency (TF-IDF), Automatic Keyphrase Extraction (KEA), n-grams, etc. (Salton & McGill, 1983; Steve & Gordon, 2022). Sometimes, these approaches may not be feasible to use as the article’s content may not be available. However, abstracts of the articles are always available, and thus most of the authors have exploited abstracts to compute the similarity between articles.

The metadata-based comparison of the articles is the most convenient approach. Mos the digital libraries rely on these approaches. The metadata of articles such as author, paper title, keywords are always publicly available and thus mainly used to find similar papers. Different techniques have been proposed using these details, such as ((Sajid et al., 2021)). However, due to limited information, these techniques may not have the capability to find interesting papers. Further metadata-based techniques cannot find the relationships between the articles.

The next category of approaches is citation-based techniques. Citations are very important in the scientific community and are being used for different impact factors and h-index (Garfield, 2006; Hirsch, 2005). The techniques exploit citation network information. One article cites another article. and so, the terms were citing, and cited articles are used. These citations create a citation network. There are two state-of-the-art techniques lies in the category are bibliographic coupling and co-citation (Kessler, 1963; Small, 1973). In the former approach, two articles are considered more relevant if there are common references in them. In contrast, in the latter approach, two articles are considered relevant if they are cited together in future articles.

The citations are very useful information as the authors themselves declare their relevancy with literature. However, the state-of-the-art systems use citations at the surface level. They cannot distinguish among citations, leaving the burden on the reader’s should to skim all the information on his own. Therefore, there is a need for advanced mechanisms that exploit the citation information in more depth to reveal interesting papers.

This paper has compared the in-text citation frequencies-based approach with content, metadata, and citations based (bibliographic coupling) approaches. The results indicated a higher percentage gain in finding more relevant documents than the contemporary approaches.

## Proposed Technique

**Figure 1:**
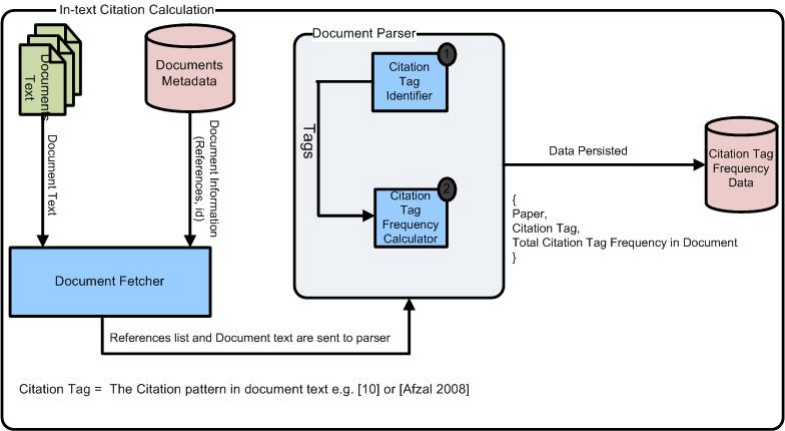
System architecture for computing in-text citation frequencies of references in the body text of the article.

**Figure 2:**
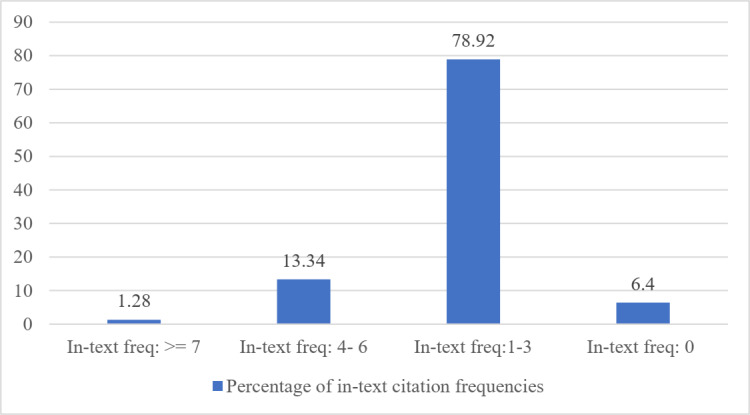
The distribution of in-text citation frequencies in various ranges.

The system architecture of this study is shown in Fig. 1. It consists of various components such as Document Fetcher, Document Parser, and then finally saving the refined data in the database for further analysis. The former module extracts document from J. UCS. Afterward, each research article is processed. In the parsing stage, citation tags of the references were identified and then their occurrences were discovered in the body text of that article. The J. UCS use multiple reference styles and thus, it was necessary to extract citation tags to find its citation frequency. The detailed analysis of the data is explained below:

There were more than 1,200 articles. The metadata data and contents of documents were parsed for in-text citation frequencies computations. More than 16,000 references were extracted from these 1,200 papers using common heuristics. Subsequently, all citation tags of each cited article in the reference list were identified. Afterward, the in-text citation frequencies of each citation tag were calculated. The citation tag is the information in a reference that is used to cite that reference in the body-text of the document, e.g., “(Muhammad et al., 2021)”, “(Afzal et al., 2007)”. After manual inspection of the dataset, we found that different authors use different citation tags such as 20, [Afzal, 2009], and (Muhammad et al., 2021). We have considered all these patterns in our implementation by employing regular expressions. The extracted citation tags were used to calculate the in-text citation frequencies in each citing paper. Some references had zero in-text citation frequencies i.e., those references which were never cited in the citing paper but were available in its references section. Those were manually verified as well. For all 16k references, numbers of papers belonging to different in-text citation frequency ranges have been shown in Fig. 2. In this Figure, some references had more than seven in-text citation frequencies, i.e., Those references were referred more than seven times within the same citing paper (the maximum 21 were recorded).

Further, 2016 instances were found that were having in-text citations of 7. Similarly, 2,134 instances had in-text citations between 4 to 6. Most of the in-text citations lied between 1 to 3, and finally, as shown in the last bar certain citations were not cited even a single time in the paper. There are only 1.28% of percent references in this data cited more than or equal to seven times by the citing papers.

Similarly, there were significant numbers of references that were having zero in-text citation frequency. Further, this study generated a ranked list of papers mentioned in the references according to the in-text citation frequencies for 1,200 articles. It means references of a paper were ranked based on in-text citation frequencies in descending order.

This study’s overall objective is to identify in-text citation frequencies in the citing paper’s body text and evaluate its usefulness in the identification of relevant documents. In-text citation frequency refers to the number of occurrences of a cited article in the citing paper’s body. In any research study, an appropriate dataset plays an essential role in assessing a system’s quality or accuracy. For this purpose, a comprehensive dataset of an online journal titled Journal of Universal Computer Science (J. UCS) was selected. In Computer Science, we were looking for a dataset that holds the following properties:

(1) It should cover all Computer Science areas; it should not be focused on one sub-domain in Computer Science. Thus, J. UCS is such a journal that covers all areas of Computer Science.

(2) The authors should come from different geographical locations and diversified backgrounds. At J. UCS, such trends can be viewed from the Geographical mashup available on the J. UCS website that clearly shows the authors’ geographical distribution. This aspect is very crucial to comprehensively evaluate in-text citation patterns as patterns from one community coming from one geographical location would be too limited to assess the effectiveness of the proposed approach on diversified patterns.

(3) It should be open access journal to retrieve and use its content by a proposed approach. The J. UCS is an open-access journal and it also provides a fair chance to the evaluation approach.

(4) Furthermore, the references should be of diversified nature. They should have errors/omissions/issues in the bibliography. The PDF should be of different versions so that the proposed approach could be verified comprehensively. Such issues are reported in the literature for J. UCS (Afzal et al., 2007).

## System Evaluation

In our experiments, we evaluated the system in two main dimensions. First, it was determined that (1) whether the in-text citation frequencies have been extracted correctly? and (2) whether the in-text citation-based recommendations help the researchers identify the most relevant papers? For this purpose, ten citing papers were randomly selected from our initial dataset. This selection was made based on the following aspects: (a) where references have been cited frequently within the text, (b) where references were cited averagely, and (c) where references were cited just once in the body text of the citing papers.

In this way, 226 reference pairs were selected for performing the evaluation. The selected papers for evaluation are shown in Table 1. In Table 1, the column with the label “IDs” represents the IDs in our database, the next column shows the titles of the papers, the columns with the label “Vol” and “Issue” represent the volumes and issues in which these articles have been published, “References” column values depict the total number of references found in a particular paper, and the “correct” and “incorrect” columns represent the verified values of the total number of references occurrences in body-text of the paper. Finally, the first column i.e., serial number, has been associated with each paper to refer them further in other tables.

**Table 1:** Randomly selected paper for conduction of user studies to evaluate the role of in-text citation in finding relevant papers.

**SrNo**	**IDs**	**Titles**	**Vol**	**Issue**	**References**	**Correct**	**Incorrect**
**1**	1001	Behavioral Institutions and Refinements in Generalized Hidden Logics	12	8	35	27	8
**2**	1136	Constant Size Ciphertext HIBE in the Augmented Selective-ID Model and its Extensions	13	10	14	10	4
**3**	114	Hausdorff Measure and Lukasiewicz Languages	11	12	23	16	7
**4**	1140	An Approach to Polygonal Approximation of Digital Curves Based on Discrete Particle Swarm Algorithm	10	10	17	15	2
**5**	118	Sequential Computability of a Function. Effective Fine Space and Limiting Recursion	11	12	17	11	6
**6**	218	Incremental Maintenance of Data Warehouses Based on Past Temporal Logic Operators	10	9	35	33	2
**7**	248	An Automatic Verification Technique for Loop and Data Reuse Transformations based on Geometric Modeling of Programs	9	3	32	24	8
**8**	299	Lazy Cyclic Reference Counting	9	8	16	13	3
**9**	53	Consensus-Based Hybrid Adaptation of Web Systems User Interfaces	11	2	26	17	9
**10**	58	On Theoretical Upper Bounds for Routing Estimation	11	6	11	11	0
		Total			226	177	49

### Evaluation of in-text citation calculation

The accuracy of the proposed system is shown in Fig. 1 was verified manually. The actual in-text frequencies from documents were manually tagged and compared with the output generated by the system. The system’s accuracy for computing in-text citation frequencies was 78% (i.e., 177/226 from Table 1).

The reasons for false identification (i.e., 22% from Table 1) of in-text citation frequencies were: (1) while citing a reference, authors have added/skipped some characters, and (2) reference in the format such as “(Jinha, 2010; Funk et al., 2017; Scopus, 2021; Gusenbauer, 2019; Shahid et al., 2020; Shahid et al., 2021)” where references number 2 to 5 are implicitly cited. Such deficiencies will be addressed in the future. However, in this work, these issues were manually corrected for further evaluation.

### Evaluation of the proposed approach

The relevant paper recommendations produced by the proposed technique were also needed to be evaluated against a baseline. Unfortunately, there is no baseline dataset in the literature based on which such sort of study can be evaluated. In such a scenario, one of the best alternatives could be the expert’s opinion-based evaluation. Therefore, we conducted a user study to define a baseline for evaluation. In this study, we selected 20 different experts. These experts were post-graduate students who were actively involved in their research. The reason for selecting 20 subjects was to get multiple feedbacks for the same paper. It was made sure that users had considerable knowledge before giving a selected paper. Afterward, 10 (in some cases less than 10) cited papers of the selected citing papers were randomly selected. This selection was performed by considering that in-text citation frequency such as 1, 2, and 3 up to 21 (the maximum) should have reasonable representations. Finally, the experts were asked to select the top five most relevant cited documents. Thus, each user classified citations of the target paper into two categories i.e., (1) citations belonging to the top-5 most relevant papers (yes), (2) and citations that do not belong to top-5 recommended list (No).

#### User rating—gold standard

For each citing paper, we received two different classifications of citations from the experts. Later on, *cohen kappa* was used to compute inter-user agreement. It is important to discover inter expert agreement because baseline can only be established in high correlation value found between them. As we have categorical data, and *cohen kappa* is a well-known correlation coefficient used in such cases. Its generic form is shown in Eq. (1). The Kappa values for each citation classification by two annotators were computed and finally averaged.

(1) \begin{eqnarray*}K= \frac{P-P \left( e \right) }{1-P \left( e \right) } \end{eqnarray*}

*P* is the relative observed agreement among annotators, where

$P \left( e \right) $** is the hypothetical probability of by chance agreement

The average correlation between experts’ opinions was recorded as 0.88. It indicates that there is strong agreement between the subjects’ ratings. As there was 88% agreement among the user’s opinion and 12% of the time, users were not agreed on similar annotations. Therefore, in the following experiments, we did not consider those papers where the users’ consensus was not identical. The experts placed the same cited papers in top-5 most relevant papers for citing papers 1, 2, 3, 4, 6, and 8. Whereas in papers 5, 7, and 9, there was no conflict on 4 cases in top-5 recommendations. Finally, for a paper with id =10, there was harmony on three citations recommended in the top-5 list by two annotators.

#### In-citation’s frequencies-based recommendations

In-text citation frequencies refer to the cited paper occurrences in the body-text of the citing papers. The in-text citation frequencies were computed using our developed tool.

The in-text citation frequencies results are shown in Table 2. This table shows the number of occurrences of cited papers in the citing papers. The top row shows the citing papers and the first column represents the considered references (i.e., cited papers). As already discussed, we had selected ten cited papers. Therefore, Table 2 contains ten rows. This table’s values can be interpreted as, for example, the cited paper “R2” of paper 2 has been cited 21 times in its body-text. This is shown with gray background in Table 2. Some references were not considered during the user study, and they are shown with an “NC” (not considered) value.

**Table 2:** In-text citation frequencies of cited papers in citing papers.

**P/ R**	**1**	**2**	**3**	**4**	**5**	**6**	**7**	**8**	**9**	**10**
**R1**	1	7	2	2	1	1	1	6	1	2
**R2**	4	21	3	1	6	2	1	2	1	1
**R3**	5	4	1	2	3	2	2	6	1	11
**R4**	3	1	5	1	7	5	2	3	1	1
**R5**	4	2	2	13	1	5	2	10	3	2
**R6**	9	3	12	0	8	16	3	1	5	1
**R7**	10	10	9	1	17	3	1	5	10	1
**R8**	3	3	n/a	1	4	6	n/a	1	12	1
**R9**	3	6	n/a	n/a	4	4	n/a	n/a	2	n/a
**R10**	n/a	2	n/a	n/a	2	2	n/a	n/a	14	n/a

Finally, top-5 cited papers were identified based on their in-text citation frequencies in the citing paper. In the end, in-text citation frequencies-based recommendations were compared with Gold standard recommendations. The accuracy percentages of the top-5 recommendations for each paper are shown in Table 3. It was found that 96% of the time, the top-5 relevant papers identified by in-text citation frequencies were like the expert’s identifications. Table 3 also depicts the average accuracy of top-5 recommendations computed in different experiments. The details of those experiments are provided in the following sections.

**Table 3:** The accuracy scores of recommendations generated by each approach for different input articles.

IDs	In-text citations	Content based	Bibliographic coupling	Title terms
1,001	0.80	0.60	0.40	0.40
1,136	1.00	0.80	0.60	0.60
114	0.80	0.60	0.40	0.40
1,140	1.00	0.80	0.60	0.60
118	1.00	0.60	0.40	0.40
218	1.00	0.60	0.80	0.40
248	1.00	1.00	0.60	0.60
299	1.00	0.80	0.60	0.80
53	1.00	0.80	0.20	0.20
58	1.00	1.00	1.00	0.40
**Avg**	**0.96**	**0.76**	**0.56**	**0.48**

#### Papers’ content-based recommendations

These approaches are also referred to as word-level similarity techniques. The word-level similarity is considered as one of the best similarity techniques by the digital library community. For computing the word-level similarities, first, the contents of the articles were indexed with Apache Lucene’s help (http://lucene.apache.org/). It has been used in numerous research tasks. Further, it was easy to setup. Research articles were provided as a basic unit of information for indexing to perform experiments. In the Lucene environment, we used TF-IDF for key terms extraction to compute vector representation of the documents. Later, documents’ similarities were computed with the help of cosine similarity. The overall similarities results are shown in Table 4. The cosine similarity score can lie between 0 and 1. The former describes dissimilarity, whereas the latter corresponds to the same documents. Finally, based on the cosine similarity score, the cited papers were ranked in top-5 most relevant papers and later compared with the expert ratings. The average accuracy of 74% was recorded for content-based recommendations. The recommendations accuracy of content-based approach for individual papers is shown in the second column of Table 3.

**Table 4:** The cosine similarity values generated based on TF-IDF terms vectors for cited and citing papers.

P/ R	1	2	3	4	5	6	7	8	9	10
R1	0.29	0.28	0.19	0.28	0.26	0.21	0.04	0.26	0.14	0.12
R2	0.34	0.35	0.28	0.03	0.27	0.28	0.2	0.25	0.18	0.33
R3	0.3	0.25	0.17	0.19	0.38	0.19	0.29	0.43	0.15	0.38
R4	0.3	0.18	0.2	0.35	0.18	0.3	0.21	0.38	0.21	0.16
R5	0.49	0.22	0.4	0.43	0.5	0.2	0.23	0.5	0.19	0.24
R6	0.21	0.2	0.13	0.07	0.43	0.27	0.74	0.45	0.34	0.11
R7	0.24	0.46	0.07	0.09	0.37	0.27	0.08	0.34	0.32	0.04
R8	0	0.24	n/a	0.11	0.21	0.23	n/a	0.19	0.48	0.02
R9	0.11	0.19	n/a	n/a	0.19	0.21	n/a	n/a	0.03	n/a
R10	n/a	0.1	n/a	n/a	0	0.17	n/a	n/a	0.05	n/a

**Figure 3:**
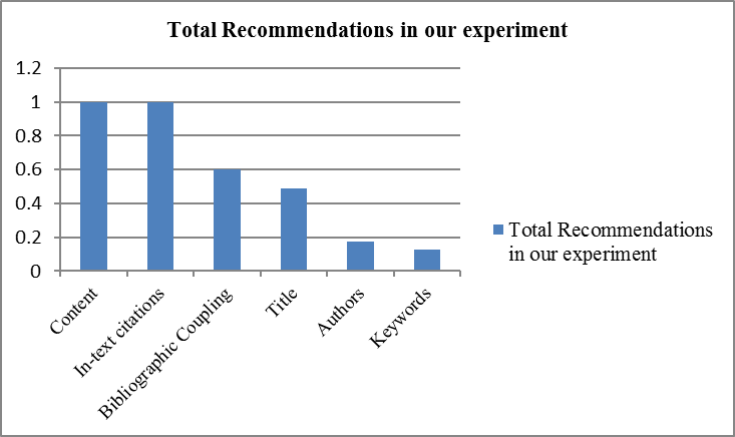
The total number of recommendations produced by each technique.

The total numbers of recommendations made by different techniques (experimented in this paper) are shown in Fig. 3. The content-based technique has produced many recommendations for each of the source papers. Hence, it can be stated that the content-based approach has potential as it requires only two documents to compute relevancy between them.

#### Bibliographic analysis

In citation-based approaches, bibliographic coupling and co-citations are the most widely known techniques. The bibliographic coupling is static, whereas co-citation is a dynamic technique. By static, we mean that bibliographic scores do not change while co-citation scores can be increased with time. In the current setup, the possible choice was bibliographic coupling. For this, common references between citing and cited papers were manually identified. The overall results are shown in Table 5. The values in Table 5 represented the number of common references between the citing and cited papers. For example, 11 common references were found between citing paper “114” (i.e., at serial number 3) and its cited paper “R3” (displayed with gray background in Table 5). Afterward, relevant documents were ranked in the top-5 recommendations list based on the number of common references between citing and cited papers. The recommendation of relevant papers for each citing paper was then compared with the gold standard.

**Table 5:** The number of common references between cited and citing papers.

P/ R	1	2	3	4	5	6	7	8	9	10
R1	4	5	0	7	5	2	0	4	0	3
R2	2	8	0	0	0	2	0	4	0	1
R3	3	2	11	1	1	0	1	0	0	4
R4	5	4	0	7	4	7	0	1	0	2
R5	0	0	0	0	0	1	3	5	0	2
R6	15	5	13	0	0	3	22	8	0	0
R7	2	11	0	0	8	3	1	1	1	0
R8	4	0	n/a	0	8	4	n/a	1	0	0
R9	0	4	n/a	n/a	3	3	n/a	n/a	0	n/a
R10	n/a	1	n/a	n/a	0	1	n/a	n/a	0	n/a

Thus, 56% of accuracy was attained. The percentage values for each paper are shown in column 3 of Table 3.

As already discussed, the bibliographic coupling technique is based on common references. Therefore, there is a possibility that two papers may not have any common references and thus, there could be zero recommendations. This may affect the overall recall of the system. In our case, only 60% of recommendations were made by bibliographic coupling-based technique (as shown in Fig. 3).

### Metadata based relevant documents

An article’s metadata could be Title, author(s), keywords, ACM topics (if any), etc. The implementation of metadata-based techniques is the easiest one. This paper ranked papers based on their metadata similarities, such as terms of papers’ title, keywords, and authors matching.

#### Paper’s title.

In this section, we compared the titles of the papers. We developed an automatic approach for comparing citing and cited papers’ titles. First, the titles were extracted and then tokenized based on white spaces. Afterward, stop words (i.e., “for,” “a,” “an” etc.) were removed and the remaining terms were stemmed using the Porter stemming algorithm (Porter, 1980). The overall results are shown in Table 6. In this table, the values represent the number of terms matched between the titles of citing and cited papers. For example, two terms were matched between the Title of the paper “1001” (i.e., at serial number 1) and the Title of reference (cited paper) “R3” (shown with gray background in Table 6). Finally, documents are ranked based on the Title’s terms matching.

**Table 6:** The similarity between cited and citing papers by considering their titles.

P/ R	1	2	3	4	5	6	7	8	9	10
R1	0	1	0	1	0	3	0	1	0	1
R2	0	1	1	3	1	1	1	0	0	0
R3	2	0	1	0	1	2	1	3	0	0
R4	0	0	0	6	1	1	0	3	2	1
R5	0	0	3	0	5	0	1	3	0	0
R6	1	0	0	0	0	0	6	2	0	0
R7	0	3	0	0	2	1	0	2	0	0
R8	0	0	n/a	0	1	1	n/a	0	1	0
R9	0	0	n/a	n/a	0	3	n/a	n/a	0	n/a
R10	n/a	0	n/a	n/a	0	1	n/a	n/a	0	n/a

The achieved results (top-5 recommendations) were compared with the Gold standard. The overall accuracy of 48% was recorded.

Since all research papers contain titles, therefore, title-based recommendations can provide recommendations in most cases. However, in our scheme, the total recommendations made by Title’s term matching are very low i.e., 48% (where title terms were matched at least one time), as shown in Fig. 3.

#### Paper’s keywords.

In this experiment, keywords of citing and cited papers were extracted. An automatic solution was built to extract the cited and citing papers (if any). It was found that most of the papers do not contain keywords and only three recommendations were made out of a total of 87 cases.

The overall recommendations made by the keywords-based similarity technique are shown in Table 7. In this table, the values represent the number of keywords matched between cited and citing papers. For example, there was only keyword matched between the “1136” (i.e., at serial number 2) and reference “R1” papers keywords (as shown with the gray background in Table 7).

**Table 7:** The similarity score between cited and citing papers using their keywords.

P/ R	1	2	3	4	5	6	7	8	9	10
R1	0	1	0	0	0	0	0	0	0	0
R2	0	0	0	0	0	0	0	0	0	0
R3	0	0	0	0	0	0	0	0	0	1
R4	0	0	0	0	0	0	0	0	0	1
R5	0	0	0	0	0	0	0	0	0	0
R6	0	0	0	0	0	0	0	0	0	0
R7	0	0	0	0	0	0	0	0	0	0
R8	0	0	0	n/a	0	0	n/a	0	0	0
R9	0	0	n/a	n/a	0	0	n/a	n/a	0	n/a
R10	n/a	0	n/a	n/a	0	0	n/a	n/a	0	n/a

#### Paper’s authors.

In this experiment, relevant documents were identified by comparing authors of the cited and citing papers. Like its predecessor, an automatic solution was designed to match the authors of the papers.

The experiments on the selected dataset revealed that keywords and authors of the papers do not play any significant role in recommending relevant papers. It can be seen in Fig. 3 that only 17% and 12% of papers were recommended by authors and keywords of the papers, respectively. The overall results of authors-based relevant papers identification are shown in Table 8. The values in Table 8 indicate that the number of authors matched between cited and cited paper.

**Table 8:** The score of similar authors for cited and citing papers.

P/ R	1	2	3	4	5	6	7	8	9	10
R1	0	0	0	0	0	0	0	0	0	0
R2	0	0	0	0	1	0	0	0	0	0
R3	0	0	0	0	1	0	0	1	0	0
R4	0	0	1	0	0	0	0	1	0	0
R5	0	0	1	0	1	0	0	1	0	0
R6	1	0	0	0	1	0	1	1	0	0
R7	0	1	0	0	1	0	0	0	0	0
R8	0	0	n/a	0	0	0	n/a	0	1	0
R9	0	0	n/a	n/a	0	0	n/a	n/a	0	n/a
R10	n/a	0	n/a	n/a	0	0	n/a	n/a	0	n/a

**Figure 4:**
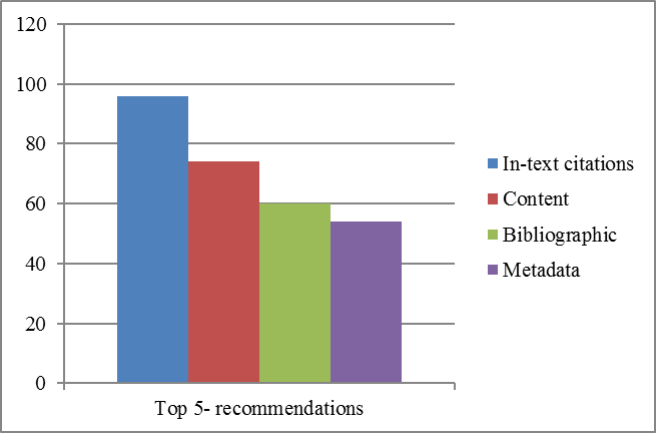
Top five recommendations of in-text citations, content, bibliographic coupling and metadata based techniques.

The overall recommendations accuracy of the techniques discussed above is shown in Fig. 4. In this Figure, in-text citation-based ranking accuracy concerning baseline is shown with a blue bar. Similarly, content, bibliographic coupling, and metadata-based recommendations accuracy concerning baseline are shown in brown, green, and purple bars. The results indicate that in-text citation frequencies-based recommendations have outperformed other state-of-the-art techniques.

## Discussion

This experiment assumed that the domain expert could determine the most relevant, less irrelevant papers corresponding to a particular paper. Based on this assumption, the gold standard dataset was defined, and later results, various options were compared against expert ranking. Our results indicated that in-text citation frequency plays a vital role in the identification of relevant papers. It was found that the percentage gain of in-text citation frequencies-based recommendations was 26%, 71%, and 100% for content, bibliographic coupling, and metadata-based techniques, respectively.

In this experiment, in-text citation frequency-based identification of relevant documents has outperformed the keyword-based recommendations. However, certain considerations should be addressed further. We have summarized them as below:

• In the future, a range of key term extraction technique should be used instead of using a single key term extraction technique.
• Experiments are conducted on a small amount of data. To analyze the proposed scheme’s real potential, these experiments should be conducted on large and diversified datasets belonging to multiple journals and conferences.
• Despite the benefits of the proposed approach, it also adds additional overhead for computation of accurate identification of in-text citations in a paper’s body-text.

In summary, the proposed approach and state-of-the-art technique are complementary and should be used in amalgamation. Based on merits and demerits, it is suggested to augment the in-text citation-based recommendation approach with other state-of-the-art techniques to improve results.

## Conclusion

The traditional techniques for relevant document identification are based on content, metadata, and bibliography information. For so long, these are being utilized by the researchers. However, most of these techniques do not consider the information in a paper’s body (i.e., textual information). In this research paper, we have evaluated and compared the in-text citation frequencies-based approach with contemporary techniques.

These approaches have been evaluated with the help of user studies. The outcomes of comparisons revealed that in-text citation frequency-based relevant document recommendations had outperformed other state-of-the-art approaches like content-based (terms based extracted using TF-IDF), metadata-based (paper’s Title, keywords, paper’s authors), and bibliography-based (bibliographic coupling).

In comparison to the baseline, it was found that in-text citation frequencies-based recommendations have a higher precision in identifying the most relevant documents, i.e., 0.96. At the same time, other state-of-the-art techniques have relatively low precision of 0.76, 0.56, and 0.48 for content, bibliographic coupling, and metadata-based techniques, respectively.

In the future, we intend to extend our experiments on diversified data set covering multiple aspects such as the substantial number of journals and conferences. Moreover, we are interested in evaluating in-text citation frequencies and their distribution to identify the relationship’s specific nature (e.g., extension, background study, etc.) between cited and citing documents.

##  Supplemental Information

10.7717/peerj-cs.524/supp-1 Supplemental Information 1 Code used in the experimentation

10.7717/peerj-cs.524/supp-2 Supplemental Information 2 Dataset used in the experimentation. These articles are available under a CC BY-ND 4.0 license.

## Additional Information and Declarations

### Data Availability

The following information was supplied regarding data availability: The data set and code are available in the Supplemental Files. Data were collected from The International Journal of Universal Computer Science (JUCS): http://www.jucs.org/.
