# Supplementary material for: In-text citation’s frequencies-based recommendations of relevant research papers
Source: PeerJ Comput Sci. 2021 Jun 4;7:e524. doi: 10.7717/peerj-cs.524 (PMC8189020; doi:10.7717/peerj-cs.524)
Supplement: Supplemental Information 2 — These articles are available under a CC BY-ND 4.0 license. [file peerj-cs-07-524-s002.zip › Dataset/Papers Dataset.zip!/Papers Dataset/jucs_1001.pdf]

Journal of Universal Computer Science, vol. 12, no. 8 (2006), 1020-1049
submitted: 7/2/05, accepted: 4/7/06, appeared: 28/8/06 © J.UCS

Behavioral Institutions and Refinements in Generalized Hidden Logics

Manuel A. Martins
Department of Mathematics
University of Aveiro, Portugal
martins@mat.ua.pt

Abstract: We investigate behavioral institutions and refinements in the context of the object oriented paradigm. The novelty of our approach is the application of generalized abstract algebraic logic theory of hidden heterogeneous deductive systems (called *hidden k-logics*) to the algebraic specification of object oriented programs. This is achieved through the *Leibniz congruence* relation and its combinatorial properties.

We reformulate the notion of hidden k-logic as well as the behavioral logic of a hidden k-logic as institutions. We define refinements as hidden signature morphisms having the extra property of preserving logical consequence. A stricter class of refinements, the ones that preserve behavioral consequence, is studied. We establish sufficient conditions for an ordinary signature morphism to be a behavioral refinement.

Key Words: Hidden Logics, Leibniz Congruence, Behavioral Equivalence Relation, Behavioral logic, Institutions, Refinements, Behavioral Refinements.
Category: F.3.1, F.3.2, F.4.1.

1 Introduction

Some computational systems often have interfaces that encapsulate the local states of objects and operations that modify them (together, these features have been called the *object oriented* (OO) *paradigm*). Thus, many programming languages have encapsulated mechanisms that hide internal data types; this provides abstraction from unimportant details, and may also be intended as protection for the internal data. Equational logic has been used as the underlying logic in many formal approaches to program specification. The programs are represented as formulas and the algebraic data types specified in this formal way can be viewed as abstract machines on which the programs are to be run. This gives an algebraic semantics for programs. Object oriented programs present a special challenge for equational methods. In the case of an OO program, a more appropriate model for the abstract machine is a state transition system. A state of an OO program is a way of protect information about the abstract machine. Data is split into a *visible* part and a *hidden* part, with the latter representing the objects. Programs are assumed to output only visible data. Hidden data can only be indirectly compared by considering the outputs of programs that take them as input. As a way of reaching the above-mentioned challenge we can replace the standard equality predicate by *behavioral equivalence*.

We take an abstract algebraic approach to the OO paradigm. The data structures are sorted algebras endowed with a designated subset of the visible part of the algebra, called a *filter*, which represents the set of truth values. Two elements are considered *behaviorally equivalent*, in a given implementation $\mathcal{A}$, if they cannot be "distinguished" in $\mathcal{A}$ by any visible program taking them as input. We apply the standard abstract algebraic logic (AAL) theory of k-deductive systems to the hidden heterogeneous case. This is achieved by using the *Leibniz congruence* relation and its properties. k-deductive systems are well known and discussed in AAL (see [Blok and Pigozzi 1989]). They are used as a context in which deductive systems (1-deductive systems, which are usually called "assertional logics"), equational logic, and the logic of partially ordered algebras can be treated simultaneously as parts of a single unified theory. The Leibniz congruence is the abstraction of the notion of logical equivalence in the classical propositional calculus which allows the generalization of the well known Lindenbaum-Tarski process to many deductive systems.

Hidden k-logics are a natural generalization of k-deductive systems. Hidden k-logics are used to specify systems whose data may be heterogeneous. Throughout this work, hidden k-logics are the underlying logics used for program specification. They are useful mainly because they encompass not only the 2-dimensional hidden and standard equational and inequational logics, but also Boolean logics; these are 1-dimensional multisorted logics with Boolean as the only visible sort, and with equality-test operations for some of the hidden sorts in place of equality predicates (see [Pigozzi 1991]). They also include all assertional logics in the purview of AAL. Thus, the use of hidden k-logics unify the treatment of all this kind of logics and provides a bridge between AAL and specification theory. Hidden k-logics were introduced by Martins and Pigozzi in [Martins and Pigozzi 2003], where the authors presented their basic properties. Then, the theory was widely developed in [Martins 2004]. Using hidden k-logics we can distinguish internal data (*hidden data*) and real data (*visible data*). This advantage is central in the specification of OO systems, since for some programs it is worth considering those kinds of encapsulated data representations either by security reasons or to simplify the process of updating and improving program implementations.

In this paper, we reformulate the notion of hidden k-logic in order to obtain an institution, which we call a *hidden k-institution*. In each hidden k-institution the signature category is fixed, as well as the set of sentences associated with each signature. We obtain different hidden k-institutions by varying the category of models. Then, we associate with any hidden k-institution other institutions, called *behavioral institutions*, whose signature morphisms have the property of preserving the behavior of the models under translations. In any behavioral hidden institution the models are the k-data structures, the sentences are the

conditional equations and the satisfaction relation is the behavioral consequence. The objects in the signature category are also fixed as the class of all hidden signatures. Behavioral hidden institutions may only differ in their class of morphisms, i.e., given a hidden k-institution $\mathcal{I}^k$, we may obtain several behavioral institutions associated with $\mathcal{I}^k$ by considering different classes of hidden signature morphisms. Moreover, they only have to be behavioral hidden signature morphisms with respect to the class of models in $\mathcal{I}^k$. Our definition of behavioral hidden institution for the special case of hidden equational logic captures almost all the behavioral institutions given in the literature.

We also study the signature morphisms that allow us to refine specifications, usually called *refinements*. From an abstract specification we construct a more concrete one; this is the basis of the *stepwise refinement process*. In the context of hidden k-logics, the notion of behavioral refinement is more appropriate, since it expresses the fact that the behavioral consequence relation is preserved (i.e, the behavioral logic is preserved). We give sufficient conditions for a refinement to be a behavioral refinement.

2 Hidden subsignatures and signature morphisms

Let SORT be a nonempty set whose elements are called *sorts*. A nonempty sequence $S_0, \dots, S_n$ of sorts in SORT ($n < \omega$) is called a *type over* SORT. We will write a type as $S_0, \dots, S_{n-1} \to S_n$. Types are represented by greek letters τ, σ, etc. The set of all types is denoted by TYPE. We distinguish visible and hidden data by splitting, in the definition of signature, the set of sorts in visible and hidden part. A *hidden* (*sorted*) *signature* is a triple $\Sigma = \langle \mathrm{SORT}, \mathrm{VIS}, \mathrm{OP} \rangle$, where SORT is a nonempty set of sorts, VIS is a subset of SORT, which we call the set of *visible sorts*, and $\mathrm{OP} = \langle \mathrm{OP}_\tau : \tau \in \mathrm{TYPE} \rangle$, where OP_τ is a countable set of operation symbols of type τ. We call the sorts in HID := SORT$\backslash$VIS *hidden sorts*. We also require the sets of operation symbols to be pairwise disjoint in order to avoid overloading of names (i.e., for any distinct $\tau, \tau' \in \mathrm{TYPE}$, $\mathrm{OP}_\tau \cap \mathrm{OP}_{\tau'} = \emptyset$). We assume $X = \langle X_S : S \in \mathrm{SORT} \rangle$ to be a fixed locally countable sorted set of variables. We define the sorted set $\mathrm{Te}_\Sigma(X)$ of *terms* (or formulas) in the signature Σ as usual. We say that a term t is a *ground term* (or a variable-free term) if it does not have variables. We say that a hidden signature is *standard* if there is a ground term of each sort.

By a Σ-*algebra* (we simply say an algebra, if Σ is clear from the context) we mean a pair $\mathbf{A} = \big\langle \langle A_S : S \in \mathrm{SORT} \rangle, \langle \mathrm{OP}^{\mathbf{A}}_\tau : \tau \in \mathrm{TYPE} \rangle \big\rangle$, where A_S is a nonempty set for each $S \in \mathrm{SORT}$ and, for each $\tau \in \mathrm{TYPE}$ ($\tau = S_0, \dots, S_{n-1} \to S_n$), $\mathrm{OP}^{\mathbf{A}}_\tau = \big\{ O^{\mathbf{A}} : O \in \mathrm{OP}_\tau \big\}$, where $O^{\mathbf{A}}$ is an operation on A of type τ, that is $O^{\mathbf{A}} : A_{S_0} \times \cdots \times A_{S_{n-1}} \to A_{S_n}$. We assume that $A_S \neq \emptyset$, for all $S \in \mathrm{SORT}$ (note that this assumption holds automatically if Σ is standard). With this

assumption we exclude some data structures of practical interest. However, the metamathematics is simpler in this case and most results of universal algebra hold in their usual form.

A *sorted congruence on* a Σ-algebra $\mathbf{A}$ is a sorted binary relation $\theta \subseteq A^2$ such that: for each $S \in \mathrm{SORT}$, θ_S is an equivalence relation on A_S; and for every operation symbol $O \in \mathrm{OP}_\tau$, with $\tau = S_0, \ldots, S_{n-1} \to S_n$, and every pair of sequences $\langle a_0, \ldots, a_{n-1}\rangle$, $\langle a'_0, \ldots, a'_{n-1}\rangle \in A_{S_0} \times \cdots \times A_{S_{n-1}}$, we have

$$O^{\mathbf{A}}(a_0, \ldots, a_{n-1})\ \theta_{S_n}\ O^{\mathbf{A}}(a'_0, \ldots, a'_{n-1}),$$

whenever $a_i\ \theta_{S_i}\ a'_i$ for $i < n$.

We will write $a_i \equiv a'_i\ (\theta_{S_i})$, $i = 1, \ldots, n-1$, or simply $\langle a_0, \ldots, a_{n-1}\rangle \equiv \langle a'_0, \ldots, a'_{n-1}\rangle\ (\theta)$ to mean that $a_i\ \theta_{S_i}\ a'_i$, for each $i < n$ (often we omit the reference to the sort, and we write $a_i \equiv a'_i\ (\theta)$).

We define in the natural way the operations in $\mathrm{Te}_\Sigma(X)$ to get the *term algebra* over the signature Σ.

It is well known that $\mathrm{Te}_\Sigma(X)$ has the universal mapping property over X in the sense that, for every Σ-algebra $\mathbf{A}$ and every sorted map $h : X \to A$, called an *assignment*, there is a unique sorted homomorphism $h^* : \mathrm{Te}_\Sigma(X) \to A$. In the sequel we will not distinguish these two maps. In particular a map from X to the set of terms, and its unique extension to an endomorphism of $\mathrm{Te}_\Sigma(X)$, is called a *substitution*. Since X is assumed fixed, we normally write Te_Σ in place of $\mathrm{Te}_\Sigma(X)$; similarly, we may write simply Te when Σ is clear from the context.

Let $\Sigma = \langle \mathrm{SORT}, \mathrm{VIS}, \mathrm{OP}\rangle$ and $\Sigma' = \langle \mathrm{SORT}', \mathrm{VIS}', \mathrm{OP}'\rangle$ be two hidden signatures. We say that Σ is *a hidden subsignature of* Σ', in symbols $\Sigma \subseteq \Sigma'$ if (a) $\mathrm{SORT} \subseteq \mathrm{SORT}'$; (b) $\mathrm{OP} \subseteq \mathrm{OP}'$ and (c) the visible parts are equal, that is $\mathrm{VIS} = \mathrm{VIS}'$ and $\mathrm{OP}_{\mathrm{VIS}} = \mathrm{OP}'_{\mathrm{VIS}'}$, where $\mathrm{OP}_{\mathrm{VIS}}$ is the subset of OP of all *strictly visible* operation symbols (i.e., operation symbols of type $S_0, \ldots, S_{n-1} \to S_n$ with $S_n \in \mathrm{VIS}$ and $S_i \in \mathrm{VIS}$, $i < n$). The minimal hidden subsignature of a given hidden signature Σ is called the *visible subsignature of* Σ and it is denoted by Σ_{VIS}. In any visible subsignature we have SORT = VIS, thus Σ_{VIS} can be seen as the (ordinary) sorted signature $\Sigma_{\mathrm{VIS}} := \langle \mathrm{VIS}, \mathrm{OP}_{\mathrm{VIS}}\rangle$.

Definition 1. Given two hidden signatures $\Sigma = \langle \mathrm{SORT}, \mathrm{VIS}, \mathrm{OP}\rangle$ and $\Sigma' = \langle \mathrm{SORT}', \mathrm{VIS}, \mathrm{OP}'\rangle$ having the same visible subsignature, a *hidden signature morphism from* Σ *to* Σ' is a mapping $\sigma : \Sigma \to \Sigma'$ (i.e., $\sigma = (\sigma_{\mathrm{SORT}}, \sigma_{\mathrm{OP}})$, with $\sigma_{\mathrm{SORT}} : \mathrm{SORT} \to \mathrm{SORT}'$ and $\sigma_{\mathrm{OP}} : \mathrm{OP} \to \mathrm{OP}'$) satisfying the following conditions:

(i) for all $V \in \mathrm{VIS}$, $\sigma_{\mathrm{SORT}}(V) = V$;

(ii) for all $O \in \mathrm{OP}_{\mathrm{VIS}}$, $\sigma_{\mathrm{OP}}(O) = O$;

(iii) If $O \in \mathrm{OP}$ is an operation symbol of type $S_0, \ldots, S_{n-1} \to S_n$ not strictly visible, then $\sigma_{\mathrm{OP}}(O)$ is an operation symbol in OP' of type $\sigma_{\mathrm{SORT}}(S_0), \ldots, \sigma_{\mathrm{SORT}}(S_{n-1}) \to \sigma_{\mathrm{SORT}}(S_n)$.

The identity mapping on Σ is a hidden signature morphism and the composition of two hidden signature morphisms is also a hidden signature morphism. Therefore, the class of all hidden signatures together with the hidden signature morphisms defines a category, called the *category of hidden signatures* which we denote by HSign. It is not difficult to see that HSign has pushouts (see [Martins 2004]).

Let $\mathrm{Alg}(\Sigma)$ be the category whose objects are the Σ-algebras and the morphisms are the algebra-morphisms. For each hidden signature morphism $\sigma : \Sigma \to \Sigma'$ we define a forgetful functor $F_\sigma : \mathrm{Alg}(\Sigma') \to \mathrm{Alg}(\Sigma)$ in the following way:

- for each Σ'-algebra $\mathbf{A}'$, $F_\sigma(\mathbf{A}') = \mathbf{A}'\restriction_\sigma$, and
- for each Σ'-homomorphism $h' : \mathbf{A}' \to \mathbf{B}'$, $F_\sigma(h') = h'\restriction_\sigma$.

Recall that the reduct $\mathbf{A}'\restriction_\sigma$ is defined by: for $S \in \mathrm{SORT}$, $(A'\restriction_\sigma)_S := A'_{\sigma(S)}$; and for each operation symbol of type $S_0, \ldots, S_{n-1} \to S_n$, $O^{\mathbf{A}'\restriction_\sigma} := \sigma(O)^{\mathbf{A}'}$. Similarly, for a Σ'-homomorphism $h' : \mathbf{A}' \to \mathbf{B}'$, where $\mathbf{A}'$ and $\mathbf{B}'$ are Σ'-algebras, the σ-reduct of h' is the Σ-homomorphism $h'\restriction_\sigma : \mathbf{A}'\restriction_\sigma \to \mathbf{B}'\restriction_\sigma$ defined by $(h'\restriction_\sigma)_S := h'_{\sigma(S)}$.

If R is a sorted relation over an algebra $\mathbf{A}'$, then the σ-reduct of R, $R\restriction_\sigma$, is the sorted relation on $A'\restriction_\sigma$ defined for any $a, a' \in A_{\sigma(S)}$ by $a\,(R\restriction_\sigma)_S\,a'$ if $a\,R_{\sigma(S)}\,a'$.

Let Σ be a hidden subsignature of Σ'. The inclusion mapping $i : \Sigma \to \Sigma'$ is a hidden signature morphism. We call i a *hidden enrichment mapping* and Σ' a *hidden enrichment of* Σ. The reduct $-\restriction_i$ is simply denoted by $-\restriction_\Sigma$. If $\mathrm{SORT} = \mathrm{SORT}'$ then a hidden enrichment is called a *hidden extension* and Σ' an *algebraic hidden extension* of Σ. Given a hidden extension Σ' of Σ, a Σ'-algebra $\mathbf{A}$ and an operation (function) f on A of type $S_0, \ldots, S_{n-1} \to S_n$, we say that an equivalence relation $\equiv$ on A, is *compatible* with f if for any $\bar{a}, \bar{a'} \in A_{S_0} \times \cdots \times A_{S_{n-1}}$, $(a_i \equiv_{S_i} a'_i, i < n)$ implies $f(\bar{a}) \equiv_{S_n} f(\bar{a'})$.

Example 1. **(State Transition Systems with evaluation by natural numbers)**
Let Σ be the signature used to specify a 2-state transition specification and Σ' be the signature used to specify a 1-state transition specification both with evaluation by natural numbers (see Figure 1).

By defining $\sigma : \Sigma \to \Sigma'$ to be the identity mapping between the visible subsignatures and, $\sigma_{\mathrm{SORT}}(\mathit{state}1) = \sigma_{\mathrm{SORT}}(\mathit{state}2) = \mathit{state}$; $\sigma_{\mathrm{OP}}(a) = \sigma_{\mathrm{OP}}(b) = f$ and $\sigma_{\mathrm{OP}}(c) = \sigma_{\mathrm{OP}}(d) = g$, we have that σ is a hidden signature morphism. $\Diamond$

2-state

Sorts :	$bool,\ nat,\ state1,\ state2$
Vis :	$bool,\ nat$
Operation symbols	
a :	$state1 \to nat$
b :	$state2 \to nat$
c :	$state2 \to state1$
d :	$state1 \to state2$
$\leq$:	$nat, nat \to bool$
s :	$nat \to nat$
$true$:	$\to bool$
$false$:	$\to bool$
$zero$:	$\to nat$

1-state

Sorts :	$bool,\ nat,\ state$
Vis :	$bool,\ nat$
Operation symbols	
f :	$state \to nat$
g :	$state \to state$
$\leq$:	$nat, nat \to bool$
s :	$nat \to nat$
$true$:	$\to bool$
$false$:	$\to bool$
$zero$:	$\to nat$

Figure 1: State Transition Systems.

The difficulty to extend a hidden signature morphism σ to a mapping between the term algebras over each signature comes from the fact that σ may not be injective on the set of sorts SORT, or not surjective on the set of operation symbols OP$'$ or both. One way to get around the first problem is to consider distinct sets of variables for each term algebra. Let X be the globally countable SORT-sorted set of variables used to form the Σ-terms. We define $X' = \langle X'_{S'} : S' \in \text{SORT}' \rangle$ to be the SORT$'$-sorted set such that $X'_{S'} = \bigcup_{\sigma(S)=S'} X_S$, if $S' = \sigma(S)$ for some $S \in \text{SORT}$ and, an arbitrary (but fixed) countable set $X'_{S'}$ of variables (disjoint from any other $X'_{S''}$), otherwise. Any assignment $h : X' \to A$ induces an assignment $h{\restriction_\sigma} : X \to A{\restriction_\sigma}$ for the variables X into the reduct $A{\restriction_\sigma}$ of A defined by $(h{\restriction_\sigma})_S(x{:}S) := h_{\sigma(S)}\big(x{:}\sigma(S)\big) \in (A{\restriction_\sigma})_S$.

First we note that $\big(\text{Te}_{\Sigma'}(X')\big){\restriction_\sigma}$ is a Σ-algebra. The mapping σ may be extended to a mapping $\widehat{\sigma}$ from $\text{Te}_\Sigma(X)$ to $\big(\text{Te}_{\Sigma'}(X')\big){\restriction_\sigma}$ defined recursively as follows:

Definition 2. Let $\sigma : \Sigma \to \Sigma'$ be a hidden signature morphism. We define $\widehat{\sigma} : \text{Te}_\Sigma(X) \to \big(\text{Te}_{\Sigma'}(X')\big){\restriction_\sigma}$ as follows (if it is clear from the context we simply write σ):

(i) if $t = x{:}S$, then $\widehat{\sigma}(t) = x{:}\sigma(S)$;

(ii) if $t = c$, then $\widehat{\sigma}(t) = \sigma(c)$;

(iii) if $t = O(t_0, ..., t_{n-1})$, with O an operation symbol of type $S_0, ..., S_{n-1} \to S_n$ then $\widehat{\sigma}(t) = \sigma(O)(\widehat{\sigma}(t_0), ..., \widehat{\sigma}(t_{n-1}))$.

To provide a context that allows us to deal simultaneously with specification logics that are assertional (for example ones with a Boolean sort) and equational, we introduce the notion of a k-term for any nonzero natural number k; a *k-term* of sort S over Σ is just a sequence of k Σ-terms of sort S. The k-terms are indicated by overlining ($\bar{\varphi}{:}S = \langle \varphi_0{:}S, ..., \varphi_{k-1}{:}S\rangle$). We denote the set of all k-terms by $\mathrm{Te}^k_\Sigma := \langle \mathrm{Te}^k_S : S \in \mathrm{SORT}\rangle$; and the set of all visible k-terms, $\langle (\mathrm{Te}^k_\Sigma)_V : V \in \mathrm{VIS}\rangle$, by $(\mathrm{Te}^k_\Sigma)_{\mathrm{VIS}}$.

For each $h : X' \to A$, we define $h^z_a : X' \to A$ by $h^z_a(u) = \begin{cases} h(u) & , u \neq z \\ a & , u = z \end{cases}$. Given a hidden signature morphism $\sigma : \Sigma \to \Sigma'$ and a Σ'-algebra $\mathbf{A}$, $h{\restriction}_\sigma$ denotes the mapping from X to $A'{\restriction}_\sigma$ defined by $(h{\restriction}_\sigma)_S(x{:}S) = h_{\sigma(S)}(x)$. Since $\bigcup_{S\in\mathrm{SORT}} X_S \subseteq \bigcup_{S\in\mathrm{SORT}'} X'_S$, any $g : X \to A'{\restriction}_\sigma$ can be extended to a mapping $g' : X' \to A$ such that $g'{\restriction}_\sigma = g$.

Lemma 3. *Let $\sigma : \Sigma \to \Sigma'$ be a hidden signature morphism, $\mathbf{A}'$ be a Σ'-algebra and $t \in \mathrm{Te}_\Sigma(X)$. Then,*

(i) *$g(t) = g'(\widehat{\sigma}(t))$, for every assignment $g : X \to A'{\restriction}_\sigma$ and every $g' : X' \to A'$ such that $g'{\restriction}_\sigma = g$.*

(ii) *$h{\restriction}_\sigma(t) = h(\widehat{\sigma}(t))$, for every assignment $h : X' \to A'$.*

2.1 Data structures

A *k-data structure* (or a *k-abstract machine*) over Σ is a pair $\mathcal{A} = \langle \mathbf{A}, F\rangle$, where $\mathbf{A}$ is a Σ-algebra and $F \subseteq A^k_{\mathrm{VIS}}$ (For any sorted set A, A_{VIS} denotes the sorted set $\langle A_V : V \in \mathrm{VIS}\rangle$). An example of a 2-data structure is any model of the free hidden equational logic over Σ (HEL_Σ) considered below (Definition 10). The standard model of HEL_Σ is of the form $\langle \mathbf{A}, id_{A_{\mathrm{VIS}}}\rangle$, where $\mathbf{A}$ is a Σ-algebra and $id_{A_{\mathrm{VIS}}}$ is the identity relation on the visible part of A, but one gets more general 2-data structures as models by taking any congruence relation on the visible part of A in place of $id_{A_{\mathrm{VIS}}}$. We can also consider the free Boolean logic over Σ if it has a Boolean sort. Here the standard models are the 1-data structures $\langle \mathbf{A}, \{\mathit{true}\}\rangle$, where $\mathbf{A}$ is a Σ-algebra such that A_{VIS} is the two-element Boolean algebra. In a general model, A_{VIS} is an arbitrary Boolean algebra and $\{\mathit{true}\}$ is replaced by an arbitrary filter on A_{VIS}.

Let $\mathcal{A} = \langle \mathbf{A}, F\rangle$ be a k-data structure over a signature Σ' and $\sigma : \Sigma \to \Sigma'$ be a hidden signature morphism. The *σ-reduct* of $\mathcal{A}$ is the k-data structure $\langle \mathbf{A}{\restriction}_\sigma, F\rangle$. When it is clear from the context, we simply write $\mathcal{A}{\restriction}_\sigma$ for $\langle \mathbf{A}{\restriction}_\sigma, F\rangle$. We should note that in $\mathcal{A}{\restriction}_\sigma$ we do not consider the σ-reduct of the filter F, since σ is the identity on the visible subsignatures and $F \subseteq A^k_{\mathrm{VIS}}$. A congruence relation θ on $\mathbf{A}$ is *compatible* with F if, for all $V \in \mathrm{VIS}$ and for all $\bar{a}, \bar{a}' \in A^k_V$, $a_i \equiv a'_i(\theta_V)$ for all $i \leq k$, implies ($\bar{a} \in F_V$ iff $\bar{a}' \in F_V$). It is not difficult to see

that the largest congruence relation on $\mathbf{A}$ compatible with F always exists (see [Martins 2004]).

Definition 4. Let $\langle \mathbf{A}, F \rangle$ be a k-data structure. The *Leibniz congruence* of F on $\mathbf{A}$ is the largest congruence relation on $\mathbf{A}$ compatible with F. It is denoted by $\Omega_{\mathbf{A}}(F)$, or simply $\Omega(F)$ when $\mathbf{A}$ is clear from the context.

One of the main properties of the Leibniz congruence is its preservation under inverse images of surjective homomorphisms.

Lemma 5 ([Martins and Pigozzi 2003]). *Let $\mathcal{A} = \langle \mathbf{A}, F \rangle$ be a k-data structure over Σ, and let $\mathbf{B}$ be a Σ algebra and $h : \mathbf{B} \to \mathbf{A}$ a surjective homomorphism. Then $h^{-1}(\Omega_{\mathbf{A}}(F)) = \Omega_{\mathbf{B}}(h^{-1}(F))$.*

Recall that if $\mathbf{A}$ is a Σ-algebra, $\bar{\varphi}(x_0{:}T_0, \ldots, x_{n-1}{:}T_{n-1})$ is a k-term and $\langle a_0, \ldots, a_{n-1} \rangle \in A_{T_0} \times \cdots \times A_{T_{n-1}}$, then we denote by $\bar{\varphi}^{\mathbf{A}}(a_0, \ldots, a_{n-1})$ the value that $\bar{\varphi}$ takes in A when the variables $x_0, \ldots, x_{n-1}$ are interpreted respectively by $a_0, \ldots, a_{n-1}$. More algebraically, $\bar{\varphi}^{\mathbf{A}}(a_0, \ldots, a_{n-1}) = h(\varphi)$, where $h : X \to A$ is any assignment such that $h(x_i) = a_i$ for all $i \leq n-1$.

A (visible) k-context over Σ $\big($resp. $\Sigma'\big)$ is a (visible) k-term $\bar{\varphi}(z{:}S, x_0{:}T_0, \ldots, x_{m-1}{:}T_{m-1}){:}V \in \mathrm{Te}^k_{\Sigma}(X)$ $\big($resp. $\mathrm{Te}^k_{\Sigma'}(X')\big)$, with a distinguished variable z of sort S and parametric variables $x_0, \ldots, x_{m-1}$. The set of all k-contexts over Σ $\big($resp. $\Sigma'\big)$ with distinguished variable z of sort S is denoted by $C^k_{\Sigma}[z{:}S]$ $\big($resp. $C^k_{\Sigma'}[z{:}S]\big)$. We call the 1-contexts simply *contexts* and we denote the set of all contexts over Σ $\big($resp. $\Sigma'\big)$ by $C_{\Sigma}[z{:}S]$ $\big($resp. $C_{\Sigma'}[z{:}S]\big)$. If $\varphi(z{:}S, \widehat{u}{:}\widehat{Q}) \in C^k_{\Sigma}[z{:}S]_V$ then $\widehat{\sigma}(\varphi)$ is a k-context with distinguished variable z of sort $\sigma(S)$ $\big($i.e., $\widehat{\sigma}(\varphi) \in C^k_{\Sigma'}[z{:}\sigma(S)]_V\big)$.

Example 2. **(State Transition Systems with evaluation by natural numbers – revisited)**
In this example σ identifies some contexts. Let $\varphi_1(z) = s(a(c(z{:}state2)){:}nat$ and $\varphi_2(z) = s(b(d(u{:}state1)){:}nat$. Their images under σ are almost the same, i.e, only their distinguished variables are different. Namely,

$$\sigma(\varphi_1) = s(f(g(z{:}state))){:}nat \text{ and } \sigma(\varphi_2) = s(f(g(u{:}state))){:}nat.$$

If the hidden signature morphism σ is surjective, as in this example, then so is $\widehat{\sigma}$. There are also examples where the hidden signature morphism is not surjective. In that case we may have k-contexts over Σ' that are not images of any k-contexts over Σ. Of course, this phenomenon will interfere with the preservation of the behavioral equivalence under hidden signature morphisms. We will see below some sufficient conditions that guarantee such preservation.$\diamondsuit$

A systematic study of the properties of the Leibniz congruence in hidden k-logics can be found in [Martins 2004]; in particular a proof of the following

characterization of the Leibniz congruence can be found there[1].

Theorem 6 ([Martins and Pigozzi 2003]). *Let Σ be a hidden signature and let $\mathcal{A} = \langle \mathbf{A}, F\rangle$ be a k-data structure over Σ. Then, for every $S \in$ SORT and for all $a, a' \in A_S$, $a \equiv a'$ $(\Omega(F)_S)$ iff for every visible k-context $\bar{\varphi}(z{:}S, u_0{:}Q_0, \dots, u_{m-1}{:}Q_{m-1}){:}V$ and for all $\langle b_0, \dots, b_{m-1}\rangle \in A_{Q_0} \times \cdots \times A_{Q_{m-1}}$,*

$$\bar{\varphi}^{\mathbf{A}}(a, b_0, \dots, b_{m-1}) \in F_V \quad \text{iff} \quad \bar{\varphi}^{\mathbf{A}}(a', b_0, \dots, b_{m-1}) \in F_V. \tag{1}$$

3 Hidden logic

For the purposes of this work is convenient to define a hidden k-logic as an abstract closure relation on the set of k-terms, independently of any specific choice of axioms and rules of inference. By a *closure relation* on $\Xi \subseteq \mathrm{Te}^k_\Sigma$ we mean a binary relation $\vdash \subseteq \mathcal{P}(\Xi) \times \Xi$ between subsets of Ξ and individual elements of Ξ satisfying for all $\Gamma, \Delta \subseteq \Xi$ the following conditions: (1) $\Gamma \vdash \bar{\gamma}$ for each $\bar{\gamma} \in \Gamma$; (2) $\Gamma \vdash \bar{\varphi}$ and $\Delta \vdash \bar{\gamma}$ for each $\bar{\gamma} \in \Gamma$ implies $\Delta \vdash \bar{\varphi}$. The closure relation is *finitary* if $\Gamma \vdash \bar{\varphi}$ implies $\Delta \vdash \bar{\varphi}$ for some globally finite subset Δ of Γ. It is *substitution-invariant* if $\Gamma \vdash \bar{\varphi}$ implies $\sigma(\Gamma) \vdash \sigma(\bar{\varphi})$ for every substitution $\sigma : X \to \mathrm{Te}_\Sigma$. Every closure relation $\vdash$ on Ξ has a natural extension to a relation, also denoted by $\vdash$, between subsets of Ξ. It is defined by $\Gamma \vdash \Delta$ if $\Gamma \vdash \bar{\varphi}$ for each $\bar{\varphi} \in \Delta$.

Definition 7. A *hidden k-logical system* (*hidden k-logic* for short) over a hidden signature Σ is a pair $\mathcal{L} = \langle \Sigma, \vdash_\mathcal{L}\rangle$, where Σ is a hidden signature and $\vdash_\mathcal{L}$ is a substitution-invariant closure relation on the set $(\mathrm{Te}^k_\Sigma)_{\mathrm{VIS}}$ of visible k-terms. A hidden k-logic is *specifiable* if $\vdash_\mathcal{L}$ is finitary.

A hidden k-logic with VIS = SORT will be called a *visible k-logic*, or simply a k-logic. As usually, in this framework k-terms will be called *k-formulas* and the set Te^k_Σ will be represented by $\mathrm{Fm}^k(\mathcal{L})$.

Hidden k-logics are useful mainly because they encompass not only the 2-dimensional hidden and standard equational logics, but also Boolean logics; these are 1-dimensional multisorted logics with Boolean as the only visible sort, and with equality-test operations for some of the hidden sorts in place of equality predicates. It also includes all assertional logics in the purview of AAL. By this way we obtain a unified theory for a variety of logical systems such as sentential logics, equational logics, order logics, and the hidden versions of all of these (see [Martins 2004]).

Normally a specifiable hidden k-logic is presented by a set of axioms (visible terms) and inference rules of the general form

[1] In the case of single-sorted 1-data structures, this result was well known in the literature of sentential logic; see for example [Blok and Pigozzi 1989].

$$\frac{\bar{\varphi}_0 : V_0, \ldots, \bar{\varphi}_{n-1} : V_{n-1}}{\bar{\varphi}_n : V_n}, \tag{2}$$

where $\bar{\varphi}_0, \ldots, \bar{\varphi}_n$ are all visible k-terms. A visible k-term $\bar{\psi}$ is *directly derivable* from a set Γ of visible k-terms by a rule such as (2) if there is a substitution $h : X \to \mathrm{Te}_\Sigma$ such that $h(\bar{\varphi}_n) = \bar{\psi}$ and $h(\bar{\varphi}_0), \ldots, h(\bar{\varphi}_{n-1}) \in \Gamma$.

Given a set AX of visible k-terms and a set IR of inference rules, we say that $\bar{\psi}$ is *derivable* from Γ by the set AX and the set IR if there is a finite sequence of k-terms, $\bar{\psi}_0, \ldots, \bar{\psi}_{n-1}$ such that $\bar{\psi}_{n-1} = \bar{\psi}$, and for each $i < n$ either (a) $\bar{\psi}_i \in \Gamma$, or (b) $\bar{\psi}_i$ is a substitution instance of a k-term in AX or (c) $\bar{\psi}_i$ is directly derivable from $\{\bar{\psi}_j : j < i\}$ by one of the inference rules in IR. It is well known, and straightforward to show, that a hidden k-logic $\mathcal{L}$ is specifiable iff there exists a (possibly) infinite set of axioms and inference rules such that, for any visible k-terms $\bar{\psi}$ and any set Γ of visible k-terms, $\Gamma \vdash_{\mathcal{L}} \bar{\psi}$ iff $\bar{\psi}$ is derivable from Γ by the given set of axioms and rules.

Let $\mathcal{L}$ be a (not necessarily specifiable) hidden k-logic. By a *theorem* of $\mathcal{L}$ we mean a (necessarily visible) k-term $\bar{\varphi}$ such that $\vdash_{\mathcal{L}} \bar{\varphi}$, i.e., $\emptyset \vdash_{\mathcal{L}} \bar{\varphi}$. The set of all theorems is denoted by $\mathrm{Thm}(\mathcal{L})$. A rule such as (2) is said to be a *derivable rule* of $\mathcal{L}$ if $\{\bar{\varphi}_0, \ldots, \bar{\varphi}_{n-1}\} \vdash_{\mathcal{L}} \bar{\varphi}_n$. A set of visible k-terms T closed under the consequence relation, i.e., $T \vdash_{\mathcal{L}} \bar{\varphi}$ implies $\bar{\varphi} \in T$, is called a *theory* of $\mathcal{L}$. The set of all theories is denoted by $\mathrm{Th}(\mathcal{L})$; if forms a complete lattice under set-theoretic inclusion. Given any set of visible k-terms Γ, the set of all consequences of Γ, in symbols $\mathrm{Con}_{\mathcal{L}}(\Gamma)$, is the smallest theory that contains Γ. Clearly, $\mathrm{Con}_{\mathcal{L}}(\Gamma) = \{\, \bar{\varphi} \in (\mathrm{Te}^k_\Sigma)_{\mathrm{VIS}} : \Gamma \vdash_{\mathcal{L}} \bar{\varphi}\}$.

If $\sigma : \Sigma \to \Sigma'$ is a hidden signature morphism and, $\mathcal{L}$ and $\mathcal{L}'$ are hidden k-logics over Σ and Σ' respectively, taking into account the discussion above, it will be worth considering $\mathrm{Fm}^k(\mathcal{L}) = \mathrm{Te}^k_\Sigma(X)$ and $\mathrm{Fm}^k(\mathcal{L}') = \mathrm{Te}^k_{\Sigma'}(X')$.

3.1 Semantics

Let $\mathcal{A} = \langle \mathbf{A}, F \rangle$ be a k-data structure. A visible k-term $\bar{\varphi}{:}V$ is said to be a *semantic consequence* of a set of visible k-terms Γ in $\mathcal{A}$, in symbols $\Gamma \models_{\mathcal{A}} \bar{\varphi}$, if, for every assignment $h : X \to A$, $h(\bar{\varphi}) \in F_V$ whenever $h(\bar{\psi}) \in F_W$ for every $\bar{\psi}{:}W \in \Gamma$. A visible k-term $\bar{\varphi}$ is a *validity* of $\mathcal{A}$, and conversely $\mathcal{A}$ is a *model* of $\bar{\varphi}$, if $\models_{\mathcal{A}} \bar{\varphi}$. A rule such as (2) is a *validity*, or a *valid rule*, of $\mathcal{L}$, and conversely $\mathcal{A}$ is a *model* of the rule, if $\{\bar{\varphi}_0, \ldots, \bar{\varphi}_{n-1}\} \models_{\mathcal{A}} \bar{\varphi}_n$. A formula $\bar{\varphi}$ is a *semantic consequence* of Γ for an arbitrary class K of k-data structures over Σ, in symbols $\Gamma \models_K \bar{\varphi}$, if $\Gamma \models_{\mathcal{A}} \bar{\varphi}$ for each $\mathcal{A} \in K$. Similarly, a k-term or rule is a *validity of* K if it is a validity of each member of K.

$\mathcal{A}$ is a *model* of a hidden k-logic $\mathcal{L}$ if every consequence of $\mathcal{L}$ is a semantic consequence of $\mathcal{A}$, i.e., $\Gamma \vdash_{\mathcal{L}} \bar{\varphi}$ always implies $\Gamma \models_{\mathcal{A}} \bar{\varphi}$. The class of all models of $\mathcal{L}$ is denoted by $\mathrm{Mod}(\mathcal{L})$. If $\mathcal{L}$ is a specifiable hidden k-logic, then $\mathcal{A}$ is a model of $\mathcal{L}$ iff every axiom and rule of inference is a validity of $\mathcal{A}$. The class of all

reduced models of $\mathcal{L}$, i.e., all models $\langle \mathbf{A}, F\rangle$ such that $\Omega(F) = id_A$, is denoted by $\mathrm{Mod}^*(\mathcal{L})$.

The proof of the following result can be found in [Martins and Pigozzi 2003]. For sentential logics the result is well known; see for example [Wójcicki 1988].

Theorem 8 (Completeness theorem for k-logics). *For any hidden k-logic $\mathcal{L}$,*

$$\vdash_{\mathcal{L}} \;=\; \models_{\mathrm{Mod}(\mathcal{L})} \;=\; \models_{\mathrm{Mod}^*(\mathcal{L})},$$

i.e., for every set of k-terms Γ and any k-term $\bar{\varphi}$, $\Gamma \vdash_{\mathcal{L}} \bar{\varphi}$ iff $\Gamma \models_{\mathrm{Mod}(\mathcal{L})} \bar{\varphi}$ iff $\Gamma \models_{\mathrm{Mod}^*(\mathcal{L})} \bar{\varphi}$.

We now present a version of the *Satisfaction Lemma* for k-data structures over hidden signatures. The equational version for ordinary algebras is due to Burstall and Goguen (see [Goguen and Burstall 1992]). For the hidden equational case the result can be found in [Roşu 2000].

Lemma 9 (Satisfaction Lemma). *Let σ be a hidden signature morphism from Σ to Σ', $\Gamma \cup \{\bar{\varphi}\} \subseteq \mathrm{Te}^k_{\Sigma}(X)_{\mathrm{VIS}}$ and $\mathcal{A}' = \langle \mathbf{A}', F\rangle$ a k-data structure over Σ'. Then*

$$\sigma(\Gamma) \models_{\mathcal{A}'} \sigma(\bar{\varphi}) \quad \text{iff} \quad \Gamma \models_{\mathcal{A}'\restriction_\sigma} \bar{\varphi}.$$

Proof. ($\Rightarrow$) Assume that $\sigma(\Gamma) \models_{\langle \mathbf{A}', F\rangle} \sigma(\bar{\varphi})$. Let $g : X \to A'\restriction_\sigma$ be an arbitrary assignment. Suppose that $g(\Gamma) \subseteq F$. Let $g' : X' \to A'$ be an assignment such that $g'\restriction_\sigma = g$. Thus, $g(\Gamma) = g'\restriction_\sigma(\Gamma) = g'(\sigma(\Gamma))$.

Hence, $g'(\sigma(\Gamma)) \subseteq F$. By hypothesis, $g'(\sigma(\bar{\varphi})) \in F$. Since $g'(\sigma(\bar{\varphi})) = g'\restriction_\sigma(\bar{\varphi}) = g(\bar{\varphi})$ then $g(\bar{\varphi}) \in F$.

($\Leftarrow$) Assume now $\Gamma \models_{\langle \mathbf{A}'\restriction_\sigma, F\rangle} \bar{\varphi}$ and let $g : X' \to A'$ be an assignment. Suppose that $g(\sigma(\Gamma)) \subseteq F$. Then, $g\restriction_\sigma(\Gamma) = g(\sigma(\Gamma)) \subseteq F$. Hence, by hypothesis, $g\restriction_\sigma(\bar{\varphi}) \in F$. Since $g\restriction_\sigma(\bar{\varphi}) = g(\sigma(\bar{\varphi}))$, then $g(\sigma(\bar{\varphi})) \in F$. □

3.2 Hidden equational logic

Let Σ be a hidden signature. In our approach to the hidden equational case, a 2-term $\langle t, s\rangle$ over Σ is intended to represent the equation $t \approx s$ and a rule $\dfrac{\langle t_0, s_0\rangle, \ldots, \langle t_{n-1}, s_{n-1}\rangle}{\langle t_n, s_n\rangle}$ represents the conditional equation $t_0 \approx s_0, \ldots, t_{n-1} \approx s_{n-1} \to t_n \approx s_n$. The set of all equations over Σ is denoted by Eq_{Σ}.

As a consequence of the restriction to visible k-terms in our formalization of hidden k-logics, the non-visible part of our hidden equational logic is truly hidden; indeed no representation of the equality predicate between elements of the hidden domains even exists in the object language. In reasoning about hidden data in the object language, only visible properties expressible in the form of conditional equations are allowed. Equality predicates over the hidden sorts

are however present in our second version of equational logic (technically, this is accomplished by simply modifying the signature by making all sorts visible). But this is done solely for the purpose of being able to express behavioral equivalence in the object language. The interplay between these two versions of equational logics is a characteristic feature of our abstract algebraic logic approach to the OO paradigm (see [Martins 2004] and [Martins and Pigozzi 2003]).

Definition 10 (Free hidden equational logic). Let Σ be a hidden signature and VIS its set of visible sorts. The *free hidden equational logic* over Σ, in symbols HEL_Σ, is the specifiable hidden 2-logic presented as follows.

Axioms: $x{:}V \approx x{:}V$, for all $V \in \mathrm{VIS}$

Inference rules: for each $V, W \in \mathrm{VIS}$,

$$(\mathrm{IR}_1) \qquad \frac{x{:}V \approx y{:}V}{y{:}V \approx x{:}V}\ ,$$

$$(\mathrm{IR}_2) \qquad \frac{x{:}V \approx y{:}V, y{:}V \approx z{:}V}{x{:}V \approx z{:}V}\ ,$$

$$(\mathrm{IR}_3) \qquad \frac{\varphi{:}V \approx \psi{:}V}{\vartheta(x/\varphi){:}W \approx \vartheta(x/\psi){:}W}, \text{ for each } \vartheta \in \mathrm{Te}_W \text{ and each } x \in X_V.$$

The (*unrestricted*) *free equational logic* over Σ, EQL_Σ, contains an equality predicate for each sort, visible and hidden. The axioms and inference rules of the free EQL_Σ are the same as those of the free HEL_Σ, except that now V and W are allowed to range over all sorts. Thus the free EQL_Σ can be viewed as the free $\mathrm{HEL}_{\Sigma'}$, where Σ' differs from Σ only in that all sorts are assumed to be visible.

Remark. An extension of equational logic, which encompasses algebras with possibly empty domains, has been considered in the literature. If algebras with empty domains are allowed, then any equation with a quantified variable $x{:}S$ will be vacuously satisfied by any algebra having empty S-domain. This happens even in the case when the variable does not occur in the equation. Thus, if we want to deal with such algebras, in order to guarantee that the completeness theorem for an equational logic holds, we have to view an equation as a triple (X, t, t'), where X is a set of quantified variables that does not necessarily coincide with $Var(t) \cup Var(t')$. Since in our approach the carrier sets of algebras are nonempty, we can assume that only those variables which occur in t or in t' are universally quantified, and hence we may omit them. This is tacitly assumed in Definition 10.

We should note that the completeness theorems, Theorem 8 and Theorem 13, are valid in general only under the assumption that all sort domains of

models are nonempty. If this restriction is lifted, then a more complex formalization of equational logic is required; see for example [Ehrig and Mahr 1985]. However, a complete and sound equational calculus can still be formulated (see [Goguen and Meseguer 1985]). ♢

If we add additional axioms and inference rules of visible sort to a free HEL_{Σ} (a free EQL_{Σ}) we obtain an (*applied*) *hidden equational logic* ((*applied*) *equational logic*) that we simply denote by HEL_{Σ} (EQL_{Σ}). We refer to these new axioms and inference rules as *extra-logical*; in view of the completeness theorem (Theorem 13 below) they correspond respectively to identities and conditional identities, respectively, of the class of models of $\mathcal{L}$.

A k-data structure $\mathcal{A} = \langle \mathbf{A}, F\rangle$ is a model of the free HEL_{Σ} iff F is a congruence on the visible part of $\mathbf{A}$. In this case F is called a VIS-*congruence*. In the free EQL_{Σ} the models are the 2-data structures $\langle \mathbf{A}, F\rangle$ where F is a congruence with no part of it hidden, i.e., a congruence on the entire algebra $\mathbf{A}$; the theories are the congruences on the term algebra. Models of the free HEL_{Σ} (the free EQL_{Σ}) of the form $\langle \mathbf{A}, id_{A_{\mathrm{VIS}}}\rangle$ ($\langle \mathbf{A}, id_A\rangle$) are called *equality models*. The class of all equality models of a HEL_{Σ} (an EQL_{Σ}) $\mathcal{L}$ is denoted by $\mathrm{Mod}^{=}(\mathcal{L})$. Since every equality model is uniquely determined by its algebraic reduct, we shall not bother in distinguishing them in the sequel. Thus, for every HEL_{Σ} $\mathcal{L}$ we identify $\mathrm{Mod}^{=}(\mathcal{L})$ with $\{\, A : \langle \mathbf{A}, id_{A_{\mathrm{VIS}}}\rangle \in \mathrm{Mod}^{=}(\mathcal{L}) \,\}$, and similarly for the equality models of a EQL_{Σ}.

When applied to hidden equational logics, Theorem 6 has an alternative formulation.

Theorem 11 ([Martins and Pigozzi 2003]). *Let Σ be a hidden signature and let $\mathcal{A} = \langle \mathbf{A}, F\rangle$ be a model of the free* HEL_{Σ}*. Then, for every $S \in \mathrm{SORT}$ and all $a, a' \in A_S$, $a \equiv a'$ $\big(\Omega(F)_S\big)$ iff for every visible context $\varphi(z{:}S, u_0{:}Q_0, \ldots, u_{m-1}{:} Q_{m-1}){:}V$ and for all $\langle b_0, \ldots, b_{m-1}\rangle \in A_{Q_0} \times \ldots \times A_{Q_{m-1}}$,*

$$\big\langle \varphi^{\mathbf{A}}(a, b_0, \ldots, b_{m-1}), \varphi^{\mathbf{A}}(a', b_0, \ldots, b_{m-1})\big\rangle \in F_V .$$

For equality models ($F = id_{A_{\mathrm{VIS}}}$) this result was obtained independently by Goguen and Malcolm [Goguen and Malcolm 2000]. For hidden equational logics the Leibniz relation has the following useful property which also can be found in [Goguen and Malcolm 1999, Goguen and Malcolm 2000] for the case of equality models.

Corollary 12 ([Martins and Pigozzi 2003]). *Let $\mathcal{A} = \langle \mathbf{A}, F\rangle$ be a model of the free* HEL_{Σ}*. Then $\Omega(F)$ is the largest congruence in $\mathbf{A}$ whose visible part is F.*

The following completeness theorem for hidden and unrestricted equational logic is a special case of Theorem 8 (see [Martins and Pigozzi 2003]).

Theorem 13 (Completeness theorem for hidden equational logic). *Let* $\mathcal{L}$ *be a* HEL_{Σ}. *Then the following are equivalent for every visible conditional equation* ξ.

(i) ξ *is a derivable rule of* $\mathcal{L}$*;*

(ii) ξ *is a valid rule of* $\mathrm{Mod}(\mathcal{L})$*;*

(iii) ξ *is a quasi-identity of* $\mathrm{Mod}^{=}(\mathcal{L})$*;*

(iv) ξ *is a quasi-identity of* $\mathrm{Mod}^{*}(\mathcal{L})$.

In particular, a visible or unrestricted equation ψ *is a theorem of* $\mathcal{L}$ *iff it is validity of* $\mathrm{Mod}(\mathcal{L})$ *iff it is an identity of* $\mathrm{Mod}^{=}(\mathcal{L})$ *iff it is an identity of* $\mathrm{Mod}^{*}(\mathcal{L})$.

3.3 Behavioral logic

Intuitively, two hidden data elements of the same type are *behaviorally equivalent* if any visible procedure whose parameter is of this common type returns the same result when executed with either of the two corresponding objects as input. The notion arises from the alternative view of a data structure as a transition system in which the hidden data elements represent states of the system and the operations (called *methods*) that return hidden elements induce transitions between states.

Behavioral equivalence has been widely used as a tool for importing the techniques and intuitions of transition systems into the algebraic paradigm. The concept of *behaviorally valid consequence* was introduced in order to reason effectively about behavioral equivalence. The main method of behaviorally valid consequence proof theory has been coinduction combined with ordinary equational deduction.

The idea of behavioral validity for equations and conditional equations is due to Reichel (see [Reichel 1985]). These notions and their proof theory have been studied by a number of researchers; namely Goguen, Malcolm and Roşu ([Goguen and Malcolm 1999], [Goguen and Malcolm 2000], [Goguen et al. 2002], [Roşu 2000] and [Roşu and Goguen 2001]); Bidoit, Hennicker and Kurz ([Bidoit et al. 1995], [Bidoit et al. 2003] and [Hennicker 1997]); Bouhoula and Rusinowitch [Bouhoula and Rusinowitch 1995] and Leavens, Pigozzi and Martins ([Leavens and Pigozzi 2002], [Martins and Pigozzi 2003] and [Martins 2004]).

The behavioral logic of a hidden k-logic is defined as a relation between sets of equations and individual equations as follows.

Definition 14. Let $\mathcal{A} = \langle \mathbf{A}, F \rangle$ be a k-data structure over a hidden signature Σ.

(i) An equation $t \approx t'$ is said to be a *behaviorally valid consequence in* $\mathcal{A}$ of a set E of equations, in symbols $E \models^{\text{beh}}_{\mathcal{A}} t \approx t'$, if, for every assignment $h : X \to A$, $h(t) \equiv h(t')$ $(\Omega(F))$ whenever $h(s) \equiv h(s')$ $(\Omega(F))$ for every equation $s \approx s'$ in E.

(ii) An equation $t \approx t'$ is *behaviorally valid in* $\mathcal{A}$ if $\models^{\text{beh}}_{\mathcal{A}} t \approx t'$. A conditional equation $t_0 \approx t'_0, \ldots, t_{n-1} \approx t'_{n-1} \to t_n \approx t'_n$ is *behaviorally valid in* $\mathcal{A}$ if $\{t_0 \approx t'_0, \ldots, t_{n-1} \approx t'_{n-1}\} \models^{\text{beh}}_{\mathcal{A}} t_n \approx t'_n$.

Definition 15. Let K be a class of k-data structures over a hidden signature Σ.

(i) An equation $t \approx t'$ is said to be a *behavioral consequence in* K *of a set* E of equations, in symbols $E \models^{\text{beh}}_{K} t \approx t'$, if $E \models^{\text{beh}}_{\mathcal{A}} t \approx t'$ for every $\mathcal{A} \in K$.

(ii) An equation or conditional equation is *behaviorally valid in* K if it is behaviorally valid in every $\mathcal{A} \in K$.

Definition 16 (Behavioral validity over $\mathcal{L}$). Let $\mathcal{L}$ be a hidden k-logic over a hidden signature Σ.

(i) An equation $t \approx t'$ is said to be a *behavioral consequence over* $\mathcal{L}$ *of a set* E of equations, in symbols $E \models^{\text{beh}}_{\mathcal{L}} t \approx t'$, if $E \models^{\text{beh}}_{\mathcal{A}} t \approx t'$ for every $\mathcal{A} \in \text{Mod}(\mathcal{L})$.

(ii) An equation or conditional equation is *behaviorally valid over* $\mathcal{L}$ if it is behaviorally valid in every $\mathcal{A} \in \text{Mod}(\mathcal{L})$.

Since $\models^{\text{beh}}_{\mathcal{L}} = \models_K$, with K being the class of 2-data structures $\{\langle \mathbf{A}, \Omega(F) \rangle : \langle \mathbf{A}, F \rangle \in \text{Mod}(\mathcal{L})\}$ we have

Theorem 17 ([Martins 2004]). *Let* $\mathcal{L}$ *be a hidden* k*-logic. Then* $\models^{\text{beh}}_{\mathcal{L}}$ *is a substitution-invariant closure relation on* $\text{Fm}(\mathcal{L})^2$*, i.e.,* $\models^{\text{beh}}_{\mathcal{L}}$ *is a hidden 2-logic, which we call the* behavioral logic of $\mathcal{L}$.

4 Behavioral Institutions

Institutions are the abstract formalization of the process of transforming a first-order model into another over a different signature by reduction, together with the associated translation of formulas between the two signatures which preserves satisfiability. More precisely, assume that $\Sigma' \subseteq \Sigma$ are two signatures (first-order languages) and $\mathcal{M}$ is a first-order structure over Σ. Any first-order formula φ over Σ' is also a Σ-formula, and the "translation" of φ into itself

preserves satisfaction in the sense that φ is satisfiable in $\mathcal{M}$ if and only if it is satisfiable in the Σ'-reduct of $\mathcal{M}$. The notion of institution was introduced by Goguen and Burstall in [Goguen and Burstall 1992] to abstract the notion of logical system, like first-order logic, for which this *satisfaction condition* holds (see also [Goguen and Roşu 2002]).

The basic definitions and properties of institutions may be found in the book *Algebraic Foundations of System Specification* [Artesiano et al. 1999], Chapter 4, by Tarlecki, or in [Tarlecki 2000]. Let us recall the definition:

Definition 18 ([Goguen and Burstall 1992]). An institution is a 4-tuple $\mathcal{I} = \langle \mathrm{Sign}, \mathrm{Sen}, \mathrm{Mod}, \models \rangle$ consisting of:

(i) a category Sign, whose objects are called *signatures*;

(ii) a functor $\mathrm{Sen} : \mathrm{Sign} \to \mathrm{Set}$ which assigns a set, called the set of *sentences*, to each signature;

(iii) a functor $\mathrm{Mod} : \mathrm{Sign} \to \mathrm{Cat}^{op}$ that for each signature Σ gives a category whose objects are called *Σ-models* and the morphisms are called *Σ-model morphisms*;

(iv) a relation $\models_\Sigma$ between models and sentences (i.e., $\models_\Sigma \subseteq \mathrm{Mod}(\Sigma)\times\mathrm{Sen}(\Sigma)$), called the *satisfaction relation* such that for each signature morphism $\sigma : \Sigma \to \Sigma'$, each $M' \in \mathrm{Mod}(\Sigma')$ and each $\varphi \in \mathrm{Sen}(\Sigma)$ the following condition holds:

$$\mathcal{M}' \models_\Sigma \mathrm{Sen}(\sigma)(\varphi) \quad \text{iff} \quad \mathrm{Mod}(\sigma)(\mathcal{M}') \models_\Sigma \varphi. \tag{3}$$

Condition (3) is usually called *satisfaction condition*. We denote the σ-reduct $\mathrm{Mod}(\sigma)(\mathcal{M})$ by $\mathcal{M}{\restriction}_\sigma$ and the sentence translation $\mathrm{Sen}(\sigma)(\varphi)$ by $\widehat{\sigma}(\varphi)$.

Now we define a hidden k-institution. The signature category is fixed, as well as the set of sentences associated to each signature. We obtain different hidden k-institutions by varying the category of models. By using the Satisfaction Lemma we may prove that each one of them is in fact an institution.

Definition 19. A *hidden k-institution* is a 4-tuple $\mathcal{I}^k = \langle \mathrm{Sign}, \mathrm{Sen}, \mathrm{Mod}, \models \rangle$, where

(i) Sign is the category HSign whose objects are all hidden signatures and whose morphisms are the hidden signature morphisms;

(ii) Given a hidden signature Σ, the set of sentences over Σ, $\mathrm{Sen}(\Sigma)$, is the set of all visible k-formulas over Σ. If $\sigma : \Sigma \to \Sigma'$ is a hidden signature morphism then $\mathrm{Sen}(\sigma) : \mathrm{Sen}(\Sigma) \to \mathrm{Sen}(\Sigma')$ is the mapping taking a k-formula $\bar{\varphi}$ over Σ to its translation under σ, $\sigma(\bar{\varphi})$;

(iii) For each hidden signature Σ, $\mathrm{Mod}(\Sigma)$ is a category whose objects are k-data structures $\langle \mathbf{A}, F\rangle$ and the morphisms are the k-data structure homomorphisms. Recall, that a data structure homomorphism between two k-data structures is a homomorphism between the underlying algebras that maps the designated filter of the domain into a subset of the designated filter of the target k-data structures. Moreover, if $\sigma : \Sigma \to \Sigma'$ is a hidden signature morphism then

$$\mathrm{Mod}(\sigma) : \mathrm{Mod}(\Sigma') \to \mathrm{Mod}(\Sigma)$$
$$\langle \mathbf{A}, F\rangle \mapsto \langle \mathbf{A}{\restriction_\sigma}, F\rangle$$

and, if $f : \langle \mathbf{A}, F\rangle \to \langle \mathbf{B}, G\rangle$ is a k-data structure morphism, then $\mathrm{Mod}(\sigma)(f)$ is the reduct of f, $f{\restriction_\sigma}$, which is also a k-data structure morphism;

(iv) For each k-data structure $\mathcal{A} = \langle \mathbf{A}, F\rangle \in \mathrm{Mod}(\Sigma)$ and each sentence $\bar{\varphi} \in \mathrm{Sen}(\Sigma)$, $\langle \mathbf{A}, F\rangle$ *satisfies* $\bar{\varphi}$, in symbols $\langle \mathbf{A}, F\rangle \models_\Sigma \bar{\varphi}$, if $\models_{\mathcal{A}} \bar{\varphi}$.

Given a hidden k-institution, each object of the model category, $\mathrm{Mod}(\Sigma)$, defines a hidden k-logic, namely $\models_{\mathrm{Mod}(\Sigma)}$. Hence, each hidden k-institution determines a family of hidden k-logics. This family is interconnected by the satisfaction condition.

4.1 Institution for behavioral logic

The notion of behavioral hidden signature morphism with respect to a given class of k-data structures is central to formulate an institution for the behavioral logic. The principal characteristic of a behavioral hidden institution for a hidden k-institution $\mathcal{I}^k$ is that its signature morphisms have to be behavioral with respect to the class of models on $\mathcal{I}^k$. Informally, a behavioral hidden signature morphism is just a hidden signature morphism preserving behaviors, which assures the satisfaction condition. Here, we introduce this concept using the Leibniz operator, the central notion in our approach. To produce examples of behavioral institutions, we will establish some sufficient conditions for a hidden signature morphism to be behavioral.

Definition 20. Let $\sigma : \Sigma \to \Sigma'$ be a hidden signature morphism and K a class of k-data structures over Σ'. We say that σ is a *behavioral hidden signature morphism with respect to* K if for each k-data structure $\mathcal{A} = \langle \mathbf{A}, F\rangle \in K$ we have

$$\Omega_{\mathbf{A}}(F){\restriction_\sigma} = \Omega_{\mathbf{A}{\restriction_\sigma}}(F). \tag{4}$$

In the case where Σ' is a hidden extension of Σ and σ is the inclusion mapping we say that Σ' is a *behavioral conservative hidden extension of* Σ *with respect to* K.

We should note that condition (4) is the same as the one considered by Hennicker et al. to define observational signature morphisms (see [Kurz 2002], [Kurz and Hennicker 2002] and [Hennicker and Bidoit 1999]).

Since $\varOmega_{\mathbf{A}\restriction_\sigma}(F)$ is, by definition, the largest congruence on $\mathbf{A}\restriction_\sigma$ compatible with F and $\varOmega_{\mathbf{A}}(F)\restriction_\sigma$ is also a congruence on $\mathbf{A}\restriction_\sigma$ compatible with F, we always have $\varOmega_{\mathbf{A}}(F)\restriction_\sigma \subseteq \varOmega_{\mathbf{A}\restriction_\sigma}(F)$.

Remark. Now, we establish some remarks about behavioral hidden signature morphisms.

• If Σ' is an algebraic hidden extension of Σ, then the inclusion mapping $i : \Sigma \to \Sigma'$ is a behavioral hidden signature morphism with respect to a class K of k-data structures if and only if, for any $\mathcal{A} \in K$, $\varOmega_{\mathbf{A}\restriction_\Sigma}(F)$ is compatible with the interpretation in $\mathbf{A}$ of any operation symbol in $\mathrm{OP}' \setminus \mathrm{OP}$. In fact, this last condition implies that $\varOmega_{\mathbf{A}\restriction_\Sigma}(F)$ is a congruence on $\mathbf{A}$. Clearly, it also is compatible with F. Thus, $\varOmega_{\mathbf{A}\restriction_\Sigma}(F) \subseteq \varOmega_{\mathbf{A}}(F) = \varOmega_{\mathbf{A}}(F)\restriction_\Sigma$.

• For each hidden signature Σ, a *standard algebraic extension* of Σ is any signature $\Sigma[C] := \Sigma \cup \{c : \to S \mid S \in \mathrm{HID}\}$ obtained from Σ by adding a new constant for each hidden sort S. The inclusion mapping $i : \Sigma \to \Sigma[C]$ is a behavioral hidden signature morphism with respect to any class K of k-data structures over $\Sigma[C]$. In fact, let $\mathcal{A} = \langle \mathbf{A}, F\rangle$ be a k-data structure over $\Sigma[C]$. Suppose that $a \equiv a' \ \big(\varOmega_{\mathbf{A}\restriction_\Sigma}(F)\big)$. Let $\bar{\varphi}(z, \widehat{x}{:}\widehat{Q}) \in C^k_{\Sigma[C]}[z{:}S]_{\mathrm{VIS}}$ and $\widehat{b} \in A_{\widehat{Q}}$. We define $\bar{\varphi}'(z, \widehat{x}, \widehat{y})$ to be the k-context obtained from $\bar{\varphi}$ by replacing each occurrence of the new constants by a new variable of the same sort. By hypothesis, we have that $\bar{\varphi}'^{\mathbf{A}\restriction_\Sigma}(a, \widehat{b}, (c^{\mathbf{A}})_{c\in I}) \in F$ iff $\bar{\varphi}'^{\mathbf{A}\restriction_\Sigma}(a', \widehat{b}, (c^{\mathbf{A}})_{c\in I}) \in F$. That is, $\bar{\varphi}^{\mathbf{A}}(a, \widehat{b}) \in F$ iff $\bar{\varphi}^{\mathbf{A}}(a', \widehat{b}) \in F$. Therefore, $a \equiv a' \ (\varOmega_{\mathbf{A}}(F))$. ♢

The next Lemma is the behavioral version of the Satisfaction Lemma. We have to require the signature morphism to be behavioral with respect to the class K of k-data structures we are considering.

Lemma 21 (Behavioral Satisfaction Lemma). *Let K be a class of k-data structures over a hidden signature Σ' and $\sigma : \Sigma \to \Sigma'$ be a behavioral hidden signature morphism with respect to K. Then for any $\mathcal{A} = \langle \mathbf{A}, F\rangle \in K$ and every $E \cup \{t \approx t'\} \subseteq \mathrm{Eq}_\Sigma$ the following condition holds:*

$$E \models^{\mathrm{beh}}_{\mathcal{A}\restriction_\sigma} t \approx t' \quad \text{iff} \quad \sigma(E) \models^{\mathrm{beh}}_{\mathcal{A}} \sigma(t) \approx \sigma(t').$$

Proof. ($\Rightarrow$) Assume $E \models^{\mathrm{beh}}_{\mathcal{A}\restriction_\sigma} t \approx t'$ and let $g : X' \to A$ be an assignment. Suppose that $g(\sigma(E)) \subseteq \varOmega_{\mathbf{A}}(F)$. Then, $g\restriction_\sigma(E) \subseteq \varOmega_{\mathbf{A}}(F)\restriction_\sigma = \varOmega_{\mathbf{A}\restriction_\sigma}(F)$. Hence, by hypothesis, $g\restriction_\sigma(t \approx t') \in \varOmega_{\mathbf{A}\restriction_\sigma}(F)$. Since $g\restriction_\sigma(t \approx t') = g(\sigma(t) \approx \sigma(t'))$, then $g(\sigma(t) \approx \sigma(t')) \in \varOmega_{\mathbf{A}}(F)$.

($\Leftarrow$) Assume now that $\sigma(E) \models^{\mathrm{beh}}_{\mathcal{A}} \sigma(t) \approx \sigma(t')$. Let $g : X \to A\restriction_\sigma$ be an arbitrary assignment. Suppose that $g(E) \subseteq \varOmega_{\mathbf{A}\restriction_\sigma}(F)$. Let $g' : X' \to A$ be an

assignment such that $g'{\restriction_\sigma} = g$. We have that $g(E) = g'{\restriction_\sigma}(E) = g'(\sigma(E))$. So, $g'(\sigma(E)) \subseteq \varOmega_{\mathbf{A}\restriction_\sigma}(F) = \varOmega_{\mathbf{A}}(F){\restriction_\sigma}$. Then, $g'(\sigma(t \approx \sigma(t'))) \in \varOmega_{\mathbf{A}}(F){\restriction_\sigma}$. That is, $g((t \approx t')) \in \varOmega_{\mathbf{A}\restriction_\sigma}(F)$. □

This Lemma suggests the following definition of behavioral hidden institution for a given hidden k-institution $\mathcal{I}^k$. The category of models is the same as the one in $\mathcal{I}^k$ and the satisfaction relation is fixed as being the behavioral consequence relation. We may obtain different behavioral institutions for a given hidden k-institution $\mathcal{I}^k$ by varying the class of morphisms from $\varSigma$ to $\varSigma'$ among the classes of behavioral hidden signature morphisms with respect to $\mathrm{Mod}(\varSigma')$.

A systematic way of defining an institution for the behavioral equivalence in the context of observability and reachability was presented in [Kurz 2002]. The following definition can be seen has an instance of the notion of behavior institution presented by Kurz in [Kurz 2002] (see also [Kurz and Hennicker 2002]).

Definition 22. Let $\mathcal{I}^k = \langle \mathrm{Sign}, \mathrm{Sen}, \mathrm{Mod}, \models \rangle$ be a hidden k-institution. A *behavioral hidden institution for* $\mathcal{I}^k$ is a 4-tuple $\mathcal{I}^{\mathrm{beh}} = \langle \mathrm{Sign}^{\mathrm{beh}}, \mathrm{Sen}^{\mathrm{beh}}, \mathrm{Mod}, \models^{\mathrm{beh}} \rangle$, where Mod is just the category Mod in $\mathcal{I}^k$ and $\mathrm{Sign}^{\mathrm{beh}}$ is the category whose objects are all hidden signatures in Sign and whose morphisms, from $\varSigma$ to $\varSigma'$, are behavioral hidden signature morphisms with respect to $\mathrm{Mod}(\varSigma')$ (but not necessarily all). Given a hidden signature $\varSigma$, $\mathrm{Sen}^{\mathrm{beh}}(\varSigma)$ is the set of all conditional equations over $\varSigma$. Finally, the satisfaction relation is defined by means of the Leibniz congruence on the underlying algebra over the designated filter (i.e., the behavior of the k-data structure) as follows:

for each k-data structure $\mathcal{A} = \langle \mathbf{A}, F \rangle$ over $\varSigma$ and each sentence $\xi \in \mathrm{Sen}(\varSigma)$, $\langle \mathbf{A}, F \rangle$ *satisfies* ξ, in symbols $\langle \mathbf{A}, F \rangle \models_\varSigma \xi$, if $\models^{\mathrm{beh}}_{\mathcal{A}} \xi$.

If ξ is the conditional equation $C \to t \approx t'$ then

$$\langle \mathbf{A}, F \rangle \models_{\varSigma'} \sigma(\xi) \text{ iff } \sigma(C) \models^{\mathrm{beh}}_{\langle \mathbf{A}, F \rangle} \sigma(t) \approx \sigma(t').$$

Hence, by Lemma 21, we can show that the satisfaction condition holds. Consequently, $\mathcal{I}^{\mathrm{beh}}$ is an institution.

Given a hidden k-institution, the problem we have in defining a behavioral hidden institution lies in the proper choice of the signature morphisms, which have to be behavioral hidden signature morphisms with respect to the class of models. We are going to present some conditions that guarantee that a hidden signature morphism is a behavioral hidden signature with respect to specific classes of k-data structures. This allows us to formulate different behavioral hidden institutions. The institutions for the behavioral logic we propose here are formulated in the context of loose-data semantics, instead of fixed semantics studied by Goguen et al. in [Goguen and Malcolm 1999], [Roşu 2000] and [Goguen and Roşu 1999]. Their institutions may also be seen as behavioral hidden institutions in our sense.

Example 3. We say that a hidden signature $\sigma : \Sigma \to \Sigma'$ is *quasi-surjective* if for every operation symbol $O' \in \mathrm{OP}'$ for which there exists a $S \in \mathrm{HID}$ and a $c' \in C_{\Sigma'}[z{:}\sigma(S)]_{\mathrm{VIS}}$ with O' occurring in c'; there is an operation symbol $O \in \mathrm{OP}$ such that $\sigma(O) = O'$. For any quasi-surjective hidden signature morphism $\sigma : \Sigma \to \Sigma'$ and any context $c' \in C_{\Sigma'}[z{:}\sigma(S)]_{\mathrm{VIS}}$ there is a $c \in C_{\Sigma}[z{:}S]_{\mathrm{VIS}}$ such that $\sigma(c) = c'$. This implies the following result, which shows that a quasi-surjective hidden signature morphism is behavioral with respect to any class of k-data structures..

Lemma 23. *Let $\sigma : \Sigma \to \Sigma'$ be a quasi-surjective hidden signature morphism. Then for each k-data structure $\mathcal{A} = \langle \mathbf{A}, F\rangle$ over Σ', $\Omega_{\mathbf{A}}(F) \restriction_{\sigma} = \Omega_{\mathbf{A}\restriction_{\sigma}}(F)$.*

For any hidden k-institution $\mathcal{I}^k = \langle \mathrm{Sign}, \mathrm{Sen}, \mathrm{Mod}, \models\rangle$, we may define the hidden behavioral institution for $\mathcal{I}^k$ to be the institution $\mathcal{I}^{\mathrm{beh}} = \langle \mathrm{Sign}^{\mathrm{beh}}, \mathrm{Sen}^{\mathrm{beh}}, \mathrm{Mod}, \models^{\mathrm{beh}}\rangle$, where the category of signatures $\mathrm{Sign}^{\mathrm{beh}}$ has hidden signatures as objects and quasi-surjective hidden signature morphism as morphisms, and the satisfaction relation is defined in a natural way by means of the Leibniz congruence over the models' filter. The proof that this formulation is indeed an institution uses the behavioral version of the satisfaction lemma stated for k-data structures $\mathcal{A} = \langle \mathbf{A}, F\rangle$ in a class K and the fact that the morphisms are behavioral hidden signature morphisms with respect to the class K (see Lemma 23). The signature morphisms in this behavioral hidden institution are defined syntactically. Consequently, the behavioral hidden institutions do not depend on the class of models we have in the hidden k-institution. ♢

Now we are going to establish two sufficient conditions for a hidden signature morphism $\sigma : \Sigma \to \Sigma'$ to be behavioral with respect to the class of models of a hidden k-logic. These results may be used to formulate others behavioral hidden institutions by restricting the signature morphisms to the ones satisfying the condition expressed in each of the premises of each theorem.

Theorem 24. *Let Σ and Σ' be two hidden signatures and $\mathcal{L}'$ be a hidden k-logic over Σ'. Let $\sigma : \Sigma \to \Sigma'$ be a hidden signature morphism. If for every $\bar{\varphi}'(z, \widehat{x}{:}\sigma(\widehat{Q}), \widehat{y}{:}\widehat{R}) \in C^k_{\Sigma'}[z{:}\sigma(S)]_{\mathrm{VIS}}$ there is a $\bar{\varphi}(z{:}S, \widehat{x}{:}\widehat{Q}) \in C^k_{\Sigma}[z{:}S]_{\mathrm{VIS}}$ such that* (a) *$\bar{\varphi}'(z, \widehat{x}, \widehat{y}) \vdash_{\mathcal{L}'} \sigma(\bar{\varphi}(z, \widehat{x}))$ and* (b) *$\sigma(\bar{\varphi}(z, \widehat{x})) \vdash_{\mathcal{L}'} \bar{\varphi}'(z, \widehat{x}, \widehat{y})$, then σ is a behavioral hidden signature morphism with respect to* $\mathrm{Mod}(\mathcal{L}')$.

Proof. Let $\mathcal{A} = \langle \mathbf{A}, F\rangle \in \mathrm{Mod}(\mathcal{L}')$ and $a, a' \in A$ such that $a \equiv a'(\Omega_{\mathbf{A}\restriction_{\sigma}}(F))$.

Let $\bar{\varphi}'(z, \widehat{x}{:}\sigma(\widehat{Q}), \widehat{y}{:}\widehat{R}) \in C^k_{\Sigma'}[z{:}\sigma(S)]_{\mathrm{VIS}}$, $\widehat{b} \in A_{\widehat{Q}}$ and $\widehat{c} \in A_{\widehat{R}}$ such that $\bar{\varphi}'^{\mathbf{A}}(a, \widehat{b}, \widehat{c}) \in F$. By hypothesis, there is $\bar{\varphi}(z{:}S, \widehat{x}{:}\widehat{Q}) \in C^k_{\Sigma}[z{:}S]_{\mathrm{VIS}}$ satisfying (a). This implies, $(\sigma\bar{\varphi})^{\mathbf{A}}(a, \bar{b}) \in F$. Moreover, we have $(\sigma\bar{\varphi})^{\mathbf{A}}(a, \bar{b}) = \bar{\varphi}^{\mathbf{A}\restriction_{\sigma}}(a, \bar{b})$. Since we are assuming $a \equiv a'(\Omega_{\mathbf{A}\restriction_{\sigma}}(F))$, by the characterization of the Leibniz congruence, $\bar{\varphi}^{\mathbf{A}\restriction_{\sigma}}(a', \bar{b}) \in F$. Hence, $(\sigma(\bar{\varphi}))^{\mathbf{A}}(a', \bar{b}) \in F$. And finally, by (b)

$\bar{\varphi}'(a', \bar{b}, \bar{c}) \in F$. The other implication may be proven similarly. Therefore, $a \equiv a' \left(\Omega_{\mathbf{A}}(F)\restriction_{\sigma(S)}\right)$. □

In the hidden equational case we have a simpler sufficient condition for a hidden signature morphism to be a behavioral hidden signature morphism (for a similar result see Goguen and Malcolm in [Goguen and Malcolm 1999]):

Theorem 25. *Let Σ and Σ' be two hidden signatures and $\mathcal{L}'$ be a hidden equational logic over Σ'. Let $\sigma : \Sigma \to \Sigma'$ be a hidden signature morphism. If for each hidden sort S and each $\varphi' \in C_{\Sigma'}[z{:}\sigma(S)]_{\mathrm{VIS}}$, there is a $\varphi \in C_{\Sigma}[z{:}S]_{\mathrm{VIS}}$, such that $\varphi' \equiv \sigma(\varphi) \left(\Omega(\mathrm{Thm}(\mathcal{L}'))\right)$ (i.e., $\vdash_{\mathcal{L}'} \varphi' \approx \sigma(\varphi)$), then σ is a behavioral hidden signature morphism with respect to* $\mathrm{Mod}(\mathcal{L}')$.

Proof. Let $\mathcal{A} = \langle \mathbf{A}, F\rangle \in \mathrm{Mod}(\mathcal{L}')$ and $a, a' \in (A\restriction_{\sigma})_S$. Suppose that $a \equiv a' \left(\Omega_{\mathbf{A}\restriction_{\sigma}}(F)_S\right)$.

Let $\varphi' \in C_{\Sigma'}[z{:}\sigma(S)]_{\mathrm{VIS}}$ and $h : X' \to A$. By hypothesis, there is $\varphi \in C_{\Sigma}[z{:}S]_{\mathrm{VIS}}$ such that $\vdash_{\mathcal{L}'} \varphi' \approx \sigma(\varphi)$. Since $\mathcal{A} \in \mathrm{Mod}(\mathcal{L}')$, by the completeness theorem of hidden equational logics,

$$h_a^z(\varphi') \equiv h_a^z(\sigma(\varphi))\ (F) \text{ and } h_{a'}^z(\varphi') \equiv h_{a'}^z(\sigma(\varphi))\ (F).$$

On the other hand, $h_a^z(\sigma(\varphi)) = h_a^z\restriction_{\sigma}(\varphi) = (h\restriction_{\sigma})_a^z(\varphi)$ and $h_{a'}^z(\sigma(\varphi)) = h_{a'}^z\restriction_{\sigma}(\varphi) = (h\restriction_{\sigma})_{a'}^z(\varphi)$.

By hypothesis, $(h\restriction_{\sigma})_a^z(\varphi) \equiv (h\restriction_{\sigma})_{a'}^z(\varphi)\ (F)$.
Therefore, $h_a^z(\sigma(\varphi)) \equiv h_{a'}^z(\sigma(\varphi))\ (F)$. Thus $h_a^z(\varphi') \equiv h_{a'}^z(\varphi')\ (F)$ because F is a VIS-congruence. Since this is true for every appropriate Σ'-context φ we have $a \equiv a'(\Omega_{\mathbf{A}}(F)_{\sigma(S)})$.

Therefore, $\Omega_{\mathbf{A}\restriction_{\sigma}}(F) \subseteq \Omega_{\sigma}(F)\restriction_{\sigma}$, for every k-data structure $\mathcal{A} \in \mathrm{Mod}(\mathcal{L}')$. So, σ is a behavioral refinement. □

5 Refinements

One of the main tasks in specification theory is the construction of models for a given specification logic. One of the most useful processes, in algebraic specification, to construct such a model is the *stepwise specification refinement*, which consists in refining, in an admissible way, step-by-step, the given specification in order to obtain a specification which is a precise description of an algebra. This is done step-by-step. A refinement is a step of this process, that is, a procedure to get a more concrete specification from a more abstract one. In some sense, a refinement can be seen as a way to reduce the freedom we have in building models of the specification. This freedom can be understood as the nondeterminism of the specification. We refine the specification in order that it becomes more

and more concrete and consequently the corresponding semantics gets less and less loose. Eventually, we may obtain only one model, up to isomorphism.

Another way of looking at a refinement in the equational case is the following: given a hidden equational logic $\mathcal{L}$, defined by a set of conditional equations E, and an implementation $\mathcal{A}$ we want to know if $\mathcal{A}$ is a model of any conditional equation in E. Sometimes, it is easier to work with another hidden equational logic $\mathcal{L}'$ (over the same signature as $\mathcal{L}$) defined by a set of conditional equations E', for which it is easier to show that $\mathcal{A}$ is a model. Hence, we just have to show that the equations in E are consequences of E'. If so, $\mathcal{L}'$ is called a *refinement* of $\mathcal{L}$. A general notion of refinement has to agree with the fact that any model of $\mathcal{L}'$ must be a model of $\mathcal{L}$ ($\mathrm{Mod}(\mathcal{L}') \subseteq \mathrm{Mod}(\mathcal{L})$).

The objective is to construct a sequence of specifications $\mathcal{L}_1, \ldots, \mathcal{L}_n$ such that, for each i, $\mathcal{L}_{i+1}$ is a refinement of $\mathcal{L}_i$ (in the sense that, the models of $\mathcal{L}_{i+1}$ are models of $\mathcal{L}_i$) and $\mathcal{L}_n$ is the desired description of an algebra, (see [Hannay 1999], [Sannella and Tarlecki 1988] and [Sannella and Tarlecki 1997]). By a description of an algebra we mean that $\mathcal{L}_n$ has only one model, up to isomorphism. This process is always possible when the refinements can be vertically composed, i.e., the composition of two refinements, when it is well defined, is still a refinement.

This intuitive notion of refinement, based on the condition $\mathrm{Mod}(\mathcal{L}') \subseteq \mathrm{Mod}(\mathcal{L})$, presupposes that the signatures are the same. We go beyond this restriction by allowing the signatures to differ by a hidden signature morphism. The more concrete implementation may rename or even identify some of the abstract sorts and operations. This allows us to define refinements between members of a larger class of hidden k-logics.

Definition 26. Let $\mathcal{L}$ and $\mathcal{L}'$ be two hidden k-logics over the signatures Σ and Σ', respectively. A hidden signature morphism σ from Σ to Σ' is a *refinement from $\mathcal{L}$ to $\mathcal{L}'$* (we also say that $\mathcal{L}'$ is a *σ-refinement of* $\mathcal{L}$) if for every $\Gamma \subseteq (\mathrm{Te}^k_\Sigma)_{\mathrm{VIS}}$ and every $\bar{\varphi} \in (\mathrm{Te}^k_\Sigma)_{\mathrm{VIS}}$ the following condition holds:

$$\Gamma \vdash_{\mathcal{L}} \bar{\varphi} \Rightarrow \sigma(\Gamma) \vdash_{\mathcal{L}'} \sigma(\bar{\varphi}).$$

The following theorem is a consequence of the Satisfaction Lemma (Lemma 9). It presents an alternative semantic definition of refinement that expresses that our notion of refinement follows the intuitive notion described above.

Theorem 27. *Let $\mathcal{L}$ and $\mathcal{L}'$ be two hidden k-logics and σ a hidden signature morphism from Σ to Σ'. Then the following conditions are equivalent:*

(i) *σ is a refinement from $\mathcal{L}$ to $\mathcal{L}'$;*

(ii) *for each k-data structure $\mathcal{A} = \langle \mathbf{A}, F \rangle$ over Σ', $\mathcal{A} \in \mathrm{Mod}(\mathcal{L}')$ implies $\mathcal{A}{\restriction_\sigma} \in \mathrm{Mod}(\mathcal{L})$.*

Proof. (i) $\Rightarrow$ (ii) Let $\mathcal{A} = \langle \mathbf{A}, F \rangle$ be a k-data structure over Σ' which is a model of $\mathcal{L}'$. Suppose that $\Gamma \vdash_{\mathcal{L}} \bar{\varphi}$. By hypothesis, $\sigma(\Gamma) \vdash_{\mathcal{L}'} \sigma(\bar{\varphi})$. Since $\mathcal{A}$ is a model of $\mathcal{L}'$ then by the Completeness Theorem, $\sigma(\Gamma) \models_{\mathcal{A}} \sigma(\bar{\varphi})$. By the Satisfaction Lemma, $\Gamma \models_{\mathcal{A}\restriction_\sigma} \bar{\varphi}$, this shows that $\mathcal{A}\restriction_\sigma$ is a model of $\mathcal{L}$.

(ii) $\Rightarrow$ (i) Suppose now that (ii) holds. Suppose that $\Gamma \vdash_{\mathcal{L}} \bar{\varphi}$. Let $\mathcal{A} = \langle \mathbf{A}, F \rangle$ be a model of $\mathcal{L}'$. Hence, by hypothesis, $\mathcal{A}\restriction_\sigma$ is a model of $\mathcal{L}$. By the Completeness Theorem, $\Gamma \models_{\mathcal{A}\restriction_\sigma} \bar{\varphi}$. Finally, by applying the Satisfaction Lemma, $\sigma(\Gamma) \models_{\mathcal{A}} \sigma(\bar{\varphi})$. Thus $\sigma(\Gamma) \vdash_{\mathcal{L}'} \sigma(\bar{\varphi})$. □

5.1 Behavioral refinements

One of the main interests in the study of hidden k-logic as underlying logic of specification of object oriented programs is their associated behavioral logics. A refinement of a hidden k-logic need not be a refinement of the corresponding behaviors: there may exist behavioral properties which are not preserved under refinement. We need to impose additional conditions on the signature morphisms in order to guarantee such properties are preserved. The hidden signature morphisms with this extra property are called *behavioral refinements* (see Definition 28). Moreover, they may not give refinements of the original logic. In the equational case we can prove that behavioral refinements are refinements. From the sufficient conditions we give for a hidden signature morphism to be behavioral, we find conditions for refinements to be behavioral refinements (see Corollaries 30 and 31).

Definition 28. Let $\mathcal{L}$ and $\mathcal{L}'$ be hidden k-logics over Σ and Σ', respectively. A hidden signature morphism σ from Σ to Σ' is a *behavioral refinement from* $\mathcal{L}$ *to* $\mathcal{L}'$ if for all $E \cup \{t \approx t'\} \subseteq \mathrm{Eq}_\Sigma$, the following condition holds:

$$E \models^{\mathrm{beh}}_{\mathcal{L}} t \approx t' \quad \Rightarrow \quad \sigma(E) \models^{\mathrm{beh}}_{\mathcal{L}'} \sigma(t) \approx \sigma(t').$$

In this case, we say that $\mathcal{L}'$ is *a behavioral refinement of* $\mathcal{L}$.

We should note that a behavioral refinement is not necessarily a refinement in the sense of Definition 26. However, since for hidden equational logics the restriction of $\models^{\mathrm{beh}}_{\mathcal{L}}$ to visible equations coincides with the logical consequence $\vdash_{\mathcal{L}}$, a behavioral refinement between hidden equational logics is always a refinement.

Our refinements (behavioral or not) can be vertically composed (see [Sannella and Tarlecki 1988]). That is, if $\mathcal{L}$, $\mathcal{L}'$ and $\mathcal{L}''$ are three hidden k-logics and σ_1 and σ_2 (behavioral) refinements from $\mathcal{L}$ to $\mathcal{L}'$ and from $\mathcal{L}'$ to $\mathcal{L}''$, respectively, then $\sigma_2 \circ \sigma_1$ is still a (behavioral) refinement from $\mathcal{L}$ to $\mathcal{L}''$.

A hidden signature morphism $\sigma : \Sigma \to \Sigma'$ which is behavioral with respect to $\mathrm{Mod}(\mathcal{L}')$, with $\mathcal{L}'$ a hidden k-logic over Σ', is always a behavioral refinement

from any hidden k-logic $\mathcal{L}$, over Σ, to $\mathcal{L}'$ whenever σ is a refinement from $\mathcal{L}$ to $\mathcal{L}'$. This is stated in the following theorem[2].

Theorem 29. *Let $\mathcal{L}$ and $\mathcal{L}'$ be hidden k-logics over Σ and Σ', respectively; and let $\sigma : \Sigma \to \Sigma'$ be a hidden signature morphism. If σ is a refinement from $\mathcal{L}$ to $\mathcal{L}'$ which is also a behavioral hidden signature morphism with respect to* $\mathrm{Mod}(\mathcal{L}')$ *(i.e., for every $\mathcal{A} = \langle \mathbf{A}, F\rangle \in \mathrm{Mod}(\mathcal{L}')$, $\Omega_{\mathbf{A}\restriction_\sigma}(F) = \Omega_{\mathbf{A}}(F)\restriction_\sigma$) then σ is a behavioral refinement from $\mathcal{L}$ to $\mathcal{L}'$.*

Proof. Assume $E \models^{\mathrm{beh}}_{\mathcal{L}} t \approx t'$. Let $\mathcal{A} = \langle \mathbf{A}, F\rangle$ be any model of $\mathcal{L}'$. Since σ is a refinement, $\mathcal{A}\restriction_\sigma$ is a model of $\mathcal{L}$. Then, by our assumption, $E \models^{\mathrm{beh}}_{\mathcal{A}\restriction_\sigma} t \approx t'$. By hypothesis σ is a behavioral hidden signature morphism with respect to $\mathrm{Mod}(\mathcal{L}')$, from Lemma 21 we have $\sigma(E) \models^{\mathrm{beh}}_{\mathcal{A}} \sigma(t) \approx \sigma(t')$. This shows that σ is a behavioral refinement. □

Using Theorems 24 and 25 we can formulate sufficient conditions for a refinement to be a behavioral refinement.

Corollary 30. *Let $\mathcal{L}$ and $\mathcal{L}'$ be hidden k-logics over Σ and Σ', respectively; and let $\sigma : \Sigma \to \Sigma'$ be a hidden signature morphism. If σ is a refinement from $\mathcal{L}$ to $\mathcal{L}'$ such that for every $\bar{\varphi}'(z, \widehat{x}{:}\sigma(\widehat{Q}), \widehat{y}{:}\widehat{R}) \in C^k_{\Sigma'}[z{:}\sigma(S)]_{\mathrm{VIS}}$ there is a $\bar{\varphi}(z{:}S, \widehat{x}{:}\widehat{Q}) \in C^k_{\Sigma}[z{:}S]_{\mathrm{VIS}}$ such that* (a) *$\varphi'(z,\widehat{x},\widehat{y}) \vdash_{\mathcal{L}'} \sigma(\bar{\varphi}(z,\widehat{x}))$ and* (b) *$\sigma(\bar{\varphi}(z,\widehat{x})) \vdash_{\mathcal{L}'} \bar{\varphi}'(z,\widehat{x},\widehat{y})$, then σ is a behavioral refinement from $\mathcal{L}$ to $\mathcal{L}'$.*

Corollary 31. *Let $\mathcal{L}$ and $\mathcal{L}'$ be hidden equational logics over Σ and Σ', respectively; and let $\sigma : \Sigma \to \Sigma'$ be a hidden signature morphism. If σ is a refinement from $\mathcal{L}$ to $\mathcal{L}'$ such that for each hidden sort S and each $\varphi' \in C_{\Sigma'}[z{:}\sigma(S)]_{\mathrm{VIS}}$, there is a $\varphi \in C_{\Sigma}[z{:}S]_{\mathrm{VIS}}$, such that $\varphi' \equiv \sigma(\varphi)$ $\big(\Omega(\mathrm{Thm}(\mathcal{L}'))\big)$ (i.e., $\vdash_{\mathcal{L}'} \varphi' \approx \sigma(\varphi)$), then σ is a behavioral refinement from $\mathcal{L}$ to $\mathcal{L}'$.*

Given two hidden equational logics $\mathcal{L}$ and $\mathcal{L}'$ over hidden signatures Σ and Σ', respectively. If $\sigma : \Sigma \to \Sigma'$ is a surjective mapping then σ is a refinement from $\mathcal{L}$ to $\mathcal{L}'$ if and only if σ is a behavioral refinement from $\mathcal{L}$ to $\mathcal{L}'$ (it is enough to see that for every context $\varphi' \in C_{\Sigma'}[z{:}\sigma(S)]_{\mathrm{VIS}}$ there is a $\varphi \in C_{\Sigma}[z{:}S]_{\mathrm{VIS}}$ such that $\sigma(\varphi') = \varphi$ and so $\vdash_{\mathcal{L}'} \varphi' \approx \sigma(\varphi)$).

5.1.1 Examples of refinements

Example 4. **(State Transition Systems with evaluation by natural numbers – revisited)**
Consider the following hidden equational logics $\mathcal{L}$ and $\mathcal{L}'$ over Σ and Σ', respectively (Σ and Σ' are defined in Example 1):

[2] As far as we know, in the general case, the converse of this result still is an open question.

Axioms of $\mathcal{L}$:

(AX1) axioms of natural numbers with partial order compatible with successor (i.e., $n \leq m$ implies $s(n) \leq s(m)$) and *zero* being the least element; plus the axioms for the booleans;

(AX2) $\big(a(x) \leq b(d(x))\big) \approx true.$

Axioms of $\mathcal{L}'$:

The same as $\mathcal{L}$ except that we replace the last axiom by

$$\big(f(x) \leq f(g(x))\big) \approx true.$$

It is not difficult to show that σ, defined in Example 1, is a behavioral refinement. In fact, we have already shown in Example 2 that σ is surjective. Thus, for every visible Σ'-context φ' there is a visible Σ-context such that $\sigma(\varphi) = \varphi'$, which, obviously, implies $\varphi' \equiv \sigma(\varphi) \ \big(\Omega(\mathrm{Thm}(\mathcal{L}'))\big)$. In order to apply Corollary 31, we only need to show that σ is a refinement from $\mathcal{L}$ to $\mathcal{L}'$. Let $\mathcal{A} \in \mathrm{Mod}(\mathcal{L}')$. Since the previous axiom is just the translation of (AX2) under σ, $\mathcal{A}\restriction_\sigma$ is a model of $\mathcal{L}$. By Theorem 27, σ is a refinement. Therefore, σ is a behavioral refinement from $\mathcal{L}$ to $\mathcal{L}'$. ♢

Example 5. Another behavioral refinement of the hidden equational logic $\mathcal{L}$ defined in the previous example is the hidden equational logic $\mathcal{L}''$ over the signature Σ and with the same axioms as $\mathcal{L}$ except the last one, which is replaced by $b(d(x)) \approx s(a(x))$. This can be shown similarly to the previous case. ♢

6 Related and future work

An extensive study concerning hidden k-logics was presented in [Martins 2004]. The author has shown that hidden k-logics are a natural generalization of deductive systems in the AAL field. Tools and arguments of AAL were used to establish results in the specification and verification theory of OO programs.

Hidden k-logics were firstly introduced in [Martins and Pigozzi 2003], where the authors dedicated a special attention to the equational case to derive properties about the behavioral logic of hidden equational logics. The main result is the characterization of the behaviorally specifiable logics as the finitely equivalential ones.

Different formulations of the notion of institution for behavioral logic can be found in the literature. They are all based on some version of the Satisfaction Lemma. The Satisfaction Lemma for the equational case and for ordinary algebras is due to Burstall and Goguen (see [Goguen and Burstall 1992]). For hidden logics this result can be found in [Roşu 2000].

Goguen and Roşu, [Goguen and Roşu 1999], define an institution for the behavioral logic of hidden equational logics over hidden fixed-data semantics. They define their signature morphisms as the hidden morphisms $\sigma : \Sigma \to \Sigma'$, which for any observable operations symbol[3] δ' in Σ' of type $S_0, \ldots, S_{n-1} \to V$, with at least one argument of hidden sort, there exists an observable operation symbol δ such that $\sigma(\delta) = \delta'$. Moreover, if we assume that all operation symbols are observable operation symbols, as we do in this paper, then this condition implies that their signature morphisms are surjective. This condition forces the satisfaction condition to hold without any restrictions on the operation symbols. We should emphasize that their institution is formulated in the context of fixed-data semantics.

Based on the definitions of hidden signature and hidden signature morphism given in [Goguen 1989], Burstall and Diaconescu present an abstract description of an institution for the behavioral logic of hidden equation logic (see [Burstall and Diaconescu 1994]). Their hidden signatures, besides being considered in the context of fixed-data semantics, also have restrictions about the kind of operations they may have. The signatures must have at most one argument of hidden sort. Such signatures are called *monadic fixed-data signatures.* The work of Burstall and Diaconescu goes further by discussing the notion of "hiding" in an institution.

In [Hennicker and Bidoit 1999] Hennicker and Bidoit give an institutional approach to the behavioral logic of observational logics. Their theory is in the context of loose-data semantics. The notion of signature morphism considered is very restrictive. Indeed, in [Hennicker and Bidoit 1999], they say: "An essential ingredient, which up to now, is still missing, is an appropriate morphism notion for observational logics". The institution has first-order (possibly infinitary) formulas as sentences and the signature morphisms are surjective hidden signature morphisms. The signature morphisms are not exactly surjective. Indeed, according with the fact that they deal with a predefined set of observable operations, the morphisms are surjective when restricted to the subsignature defined by the observable operations.

Here we go further taking into account both formulations. We give an adequate notion of behavioral hidden institution on the context of general hidden k-logics. Either of the previously discussed notions of institutions for the behavioral logic satisfy the general description we define. We also give a specific example of a behavioral hidden institution for a hidden k-institution $\mathcal{I}$ based on the notion of quasi-injective morphism. This institution is independent of the category of models we have in $\mathcal{I}$. Moreover, our behavioral institution deals with loose-data semantics and avoids the infinitary logic of Hennicker and Bidoit and

[3] Goguen et al. did not give any specific name to this kind of operation symbols. Here, we follow Bidoit's terminology. Observable operation symbols are the operation symbols used to build the admissible contexts.

the monadic requirements of Burstall and Diaconescu.

In [Kurz 2002] was presented a systematic way to construct an institution that accommodates the relation of behavioral equivalence (indistinguishability) in the context of observability and reachability. Moreover, Kurz has shown that his general framework can be applied to observational logic, constructor-based logic, and constructor-based observational logic. Reachability was dealt with as a dual of observability (see also [Kurz and Hennicker 2002]).

Behavioral refinements in the context of hidden logics have been studied by several researchers. A very abstract theory of refinements can be found in Sannella and Tarlecki ([Sannella and Tarlecki 1997], [Sannella 2000]).

There are various notions of refinement (also called implementations, see [Hannay 1999], [Sannella and Tarlecki 1988] and [Sannella and Tarlecki 1997]). Some of them are defined between general specifications which are described by giving its signature and its class of models (for example the Bidoit et al. approach). On the other hand, Goguen et al. in [Malcolm and Goguen 1996], consider refinements in the context of hidden equational logics which are defined by conditional equations. Hennicker and Bidoit develop a method of reasoning about behavioral refinements ([Hennicker 1997]). Goguen, Malcolm and Roşu use their coinduction methods to prove correctness of behavioral refinements between hidden equational logics ([Goguen and Malcolm 1999, Lin et al. 2000] and [Hannay 1999]).

In this work we use the notion of Leibniz congruence to give a sufficient condition for a refinement to be a behavioral refinement from $\mathcal{L}$ to $\mathcal{L}'$. Namely, we show that if the σ-reduct of the Leibniz congruence on any model of $\mathcal{L}'$ is equal to the Leibniz congruence on the σ-reduct of the model, then σ being a refinement from $\mathcal{L}$ to $\mathcal{L}'$ is enough for it to be a behavioral refinement. The main differences from the other approaches concern the fact that we deal with loose-data semantics instead of only fixed-data hidden signatures and we make use of the powerful notion of Leibniz congruence. However, we have to follow assumptions regarding our interpretation of the object oriented paradigm that requires the axioms to be visible conditional equations.

6.1 Future work

The final objective in the stepwise refinement process described above is to refine a specification "step-by-step" until we obtain a specification that is categorical in the sense that it has only one model up to isomorphisms. An interesting topic of research will be to find precise conditions, if possible applicable in practice, for a hidden k-logic to be categorical. As far as we know, in the general case of hidden k-logics this problem has not been solved yet.

One of the main tasks of programming is to improve and update existing programs. So, attention should be paid to designing programming languages to make these processes as efficient as possible. One way of achieving this goal is to encapsulate data representation and allow access to them only via programs that use them as input and with visible output. This will allow changes in the code of the visible part without look at the data which are now considered internal. On the other hand, even if we have already some of the data representation hidden we may want to encapsulate more data representations (this could be required by some additional security features). The reciprocal of this process is also of interest. Indeed, it may be pertinent to consider some internal data representation as visible in order to compute its real value at each time. Hence, they should be displayed and consequently its associated sort should be now considered as a visible sort. This suggests a generalization of the notion of refinement. A refinement of a hidden k-logic $\mathcal{L}$ has the same set of visible sorts as $\mathcal{L}$; more precisely, they have the same visible subsignature. It is natural to study the case when this requirement is omitted. This means the case when a refinement of a hidden k-logic may have a different set of visible sorts.

Acknowledgements

The author wishes to thank Don Pigozzi and Isabel Ferreirim for some fruitful discussions concerning this work. The author also gratefully thanks the valuable remarks of the anonymous referees.

References

[Artesiano et al. 1999] E. Astesiano, H.-J. Kreowski, and B. Krieg-Brückner, editors. *Algebraic foundations of systems specification.* IFIP State-of-the-Art Reports. Springer-Verlag, Berlin, 1999.

[Bidoit et al. 1995] M. Bidoit, R. Hennicker, and M. Wirsing. Behavioural and abstractor specifications. *Sci. Comput. Program.*, 25(2-3):149–186, 1995.

[Bidoit et al. 2003] M. Bidoit, R. Hennicker, and A. Kurz. Observational logic, constructor-based logic, and their duality. *Theor. Comput. Sci.*, 298(3):471–510, 2003.

[Blok and Pigozzi 1989] W. J. Blok and D. Pigozzi. Algebraizable logics. *Mem. Am. Math. Soc.*, 396, 1989.

[Bouhoula and Rusinowitch 1995] A. Bouhoula and M. Rusinowitch. Implicit induction in conditional theories. *J. Autom. Reasoning*, 14(2):189–235, 1995.

[Burstall and Diaconescu 1994] R. Burstall and R. Diaconescu. Hiding and behaviour: an institutional approach. In *A. William Roscoe, editor, A Classical Mind: Essays in Honour of C.A.R. Hoare*, pages 75–92. 1994.

[Ehrig and Mahr 1985] H. Ehrig and B. Mahr. *Fundamentals of algebraic specification 1. Equations and initial semantics.* EATCS. Monographs on Theoretical Computer Science, 6. Berlin: Springer-Verlag, 1985.

[Font et al. 2003] J. Font, R. Jansana and D. Pigozzi, A survey of abstract algebraic logic. *Studia Logica*, 74:13–97, 2003.

[Goguen 1989] J. Goguen. Types as theories. In *Topology and category theory in computer science*, Oxford Sci. Publ., pages 357–390. Oxford Univ. Press, New York, 1989.

[Goguen and Burstall 1992] J. Goguen and R. M. Burstall. Institutions: Abstract model theory for specification and programming. *J. Assoc. Comput. Mach.*, 39(1):95–146, 1992.

[Goguen et al. 2002] J. Goguen, K. Lin, and G. Roşu. Conditional circular coinductive rewriting with case analysis. In *16th International Workshop, WADT 2002*, volume 2755 of *Lecture Notes in Computer Science*, pages 216–232, Frauenchiemsee, Germany, September 2002.

[Goguen and Malcolm 1999] J. Goguen and G. Malcolm. Hidden coinduction: Behavioural correctness proofs for objects. *Math. Struct. Comput. Sci.*, 9(3):287–319, 1999.

[Goguen and Malcolm 2000] J. Goguen and G. Malcolm. A hidden agenda. *Theor. Comput. Sci.*, 245(1):55–101, 2000.

[Goguen and Meseguer 1985] J. Goguen and J. Meseguer. Completeness of many-sorted equational logic. *Houston J. Math.*, 11:307–334, 1985.

[Goguen and Roşu 1999] J. Goguen and G. Roşu. Hiding more of hidden algebra. In *Wing, Jeannette M. et al. (ed.), FM '99. Formal methods. World congress on Formal methods in the development of computing systems. Toulouse, France, September 20-24. Proceedings.* 1999.

[Goguen and Roşu 2002] J. Goguen and G. Roşu. Institution morphisms. *Formal Asp. Comput.*, 13 (3-5):274–307, 2002.

[Hannay 1999] J. E. Hannay. Specification refinement with System F. In *Flum, Jörg et al. (ed.), Computer science logic. 13th international workshop, CSL '99. 8th annual conference of the EACSL, Madrid, Spain, September 20-25, 1999. Proceedings. Berlin: Springer. Lect. Notes Comput. Sci. 1683*, pages 530–545. 1999.

[Hennicker 1997] R. Hennicker. Structural specifications with behavioural operators: semantics, proof methods and applications, Habilitationsschrift thesis, 1997.

[Hennicker and Bidoit 1999] R. Hennicker and M. Bidoit. Observational logic. In *Proc. AMAST '98, 7th International Conference on Algebraic Methodology and Software Technology. Lecture Notes in Computer Science, Berlin: Springer*, pages 263–277. 1999.

[Kurz and Hennicker 2002] A. Kurz and R. Hennicker. On institutions for modular coalgebraic specifications. *Theor. Comput. Sci.*, 280, (1-2):69–103, 2002.

[Kurz 2002] A. Kurz. Notions of behaviour and reachable-part and their institutions. In *16th International Workshop, WADT 2002*, volume 2755 of *Lecture Notes in Computer Science*, pages 312–327, Frauenchiemsee, Germany, September 2002.

[Leavens and Pigozzi 2002] G. T. Leavens and D. Pigozzi. Equational reasoning with subtypes. Technical Report TR #02-07, Iowa State University, July 2002. Available at `ftp://ftp.cs.iastate.edu/pub/techreports/ TR02-07/TR.pdf`.

[Lin et al. 2000] K. Lin J. Goguen and G. Roşu. Circular coinductive rewriting. In *Proceedings, Automated Software Engineering '00 (Grenoble France), IEEE Press*, pages 123–131. September 2000.

[Malcolm and Goguen 1996] G. Malcolm and J. Goguen. Proving correctness of refinement and implementation. Technical report, Oxford University Computing Laboratory, Technical Monograph PRG-114, 1996.

[Martins 2004] M. A. Martins. *Behavioral Reasoning in generalized Hidden Logics*. PhD thesis, University of Lisbon, Faculdade de Ciências, 2004.

[Martins and Pigozzi 2003] M. A. Martins and D. Pigozzi. Behavioral reasoning for conditonal equations. *Cadernos de Matemática, Universidade de Aveiro*, (CM03/I-19), June 2003. Preprint. Available at `http://www.math.iastate.edu/dpigozzi/papers/sweconbehequ.pdf`.

[Pigozzi 1991] D. Pigozzi. Equality-test and if-then-else algebras: Axiomatization and specification. *SIAM J. Comput.*, 20(4):766–805, 1991.

[Reichel 1985] H. Reichel. Behavioural validity of conditional equations in abstract data types. In *Contributions to general algebra 3, Proc. Conf., Vienna 1984*, pages 301–324. 1985.

[Roşu 2000] G. Roşu. *Hidden Logic.* PhD thesis, University of California, San Diego, 2000.

[Roşu and Goguen 2001] G. Roşu and J. Goguen. Circular coinduction. In *International Joint Conference on Automated Reasoning (IJCAR'01), Sienna, 2001.* Available at `http://www-cse.ucsd.edu/users/goguen/pps/ccoind.ps`.

[Sannella 2000] D. Sannella. Algebraic specification and program development by stepwise refinement. (Extended abstract). In *Bossi, Annalisa (ed.), Logic-based program synthesis and transformation. 9th international workshop, LOPSTR '99. Venice, Italy, September 22-24, 1999. Selected papers. Berlin: Springer. Lect. Notes Comput. Sci. 1817*, pages 1–9. 2000.

[Sannella and Tarlecki 1988] D. Sannella and A. Tarlecki. Toward formal development of programs from algebraic specifications: Implementations revisited. *Acta Inf.*, 25(3):233–281, 1988.

[Sannella and Tarlecki 1997] D. Sannella and A. Tarlecki. Essential concepts of algebraic specification and program development. *Formal Asp. Comput.*, 9(3):229–269, 1997.

[Tarlecki 2000] A. Tarlecki. Towards heterogeneous specifications. In *Gabbay, Dov M. et al. (ed.), Frontiers of combining systems 2. Selected papers from the 2nd international workshop (FroCoS'98), Amsterdam, Netherlands, October 2-4, 1998. Baldock: Research Studies Press. Stud. Log. Comput. 7*, pages 337–360. 2000.

[Wójcicki 1988] R. Wójcicki. *Theory of logical calculi. Basic theory of consequence operations.* Synthese Library, no. 199. Dordrecht: Kluwer Academic Publishers, 1988.
