# Supplementary material for: In-text citation’s frequencies-based recommendations of relevant research papers
Source: PeerJ Comput Sci. 2021 Jun 4;7:e524. doi: 10.7717/peerj-cs.524 (PMC8189020; doi:10.7717/peerj-cs.524)
Supplement: Supplemental Information 2 — These articles are available under a CC BY-ND 4.0 license. [file peerj-cs-07-524-s002.zip › Dataset/Papers Dataset.zip!/Papers Dataset/jucs_1136.pdf]

Journal of Universal Computer Science, vol. 13, no. 10 (2007), 1367-1395
submitted: 23/3/07, accepted: 26/10/07, appeared: 28/10/07 © J.UCS

Constant Size Ciphertext HIBE in the Augmented Selective-ID Model and its Extensions

Sanjit Chatterjee
(Department of Combinatorics and Optimization
University of Waterloo
Waterloo, Ontario, Canada, N2L 3G1
s2chatterjee@math.uwaterloo.ca)

Palash Sarkar
(Applied Statistics Unit
Indian Statistical Institute
203, B.T. Road, Kolkata
India 700108.
palash@isical.ac.in)

Abstract: At Eurocrypt 2005, Boneh, Boyen and Goh presented a constant-size ciphertext hierarchical identity-based encryption (HIBE) protocol. Our main contribution is to present a variant of the BBG-HIBE. The new HIBE is proved to be secure (without any degradation) in an extension of the s-ID model (denoted the s$^+$-ID model) and the components of the identities are from $\mathbb{Z}_p$, where p is a suitable large prime. The BBG-HIBE is proved to be secure in the selective-ID (s-ID) security model and the components of the identities are from $\mathbb{Z}_p^*$. In the s$^+$-ID model the adversary is allowed to vary the length of the challenge identity whereas this is not allowed in the s-ID model. The new HIBE shares all the good features of the BBG-HIBE. The drawback is that the public parameters and the private key are longer than that of the BBG-HIBE. We also provide two more extensions of the basic constant-size ciphertext HIBE. The first is a constant-size ciphertext HIBE secure in the generalised selective-ID model $\mathcal{M}_2$. The second one is a product construction composed of two HIBEs and a trade-off is possible between the private key size and the ciphertext size.

Key Words: hierarchical identity-based encryption, standard model, bilinear pairing.
Category: E.3

1 Introduction

An identity-based encryption (IBE) protocol offers certain flexibility over usual public key encryption protocols by allowing the public key to be any binary string. This notion was introduced by Shamir [Shamir, 1984] and the first efficient implementation with a proof of security in an appropriate security model was given by Boneh and Franklin [Boneh and Franklin, 2003]. In an IBE, the private key corresponding to an identity is generated by a private key generator (PKG) and is securely transmitted to the appropriate entity. Encryption is

done using the identity and the public parameters of the PKG whereas decryption requires the private key of the identity under which the message has been encrypted.

The role of the PKG is to distribute private keys. A generalization of IBE is the notion of a hierarchical IBE (HIBE) introduced by [Horwitz and Lynn, 2002] and [Gentry and Silverberg, 2002], which allows the task of generating private keys to be delegated to lower levels. Several constructions of HIBE exist in literature [Gentry and Silverberg, 2002, Boneh and Boyen, 2004, Boneh et al., 2005]. In the constructions of [Gentry and Silverberg, 2002, Boneh and Boyen, 2004] the length of the ciphertexts, the size of the private keys and consequently, the time required for encryption and decryption grow linearly with the number of levels in the HIBE.

In a recent work, a very interesting construction of HIBE was presented by Boneh, Boyen and Goh [Boneh et al., 2005], which we call BBG-HIBE. The main novelty of the BBG-HIBE is that the size of the ciphertext is independent of the depth of the HIBE. This also improves the efficiency of encryption and decryption. Perhaps more importantly, the constant-size ciphertext BBG-HIBE leads to improved constructions of forward secure public key and identity-based encryption where all messages encrypted before the secret key is compromised remain secret and public-key broadcast encryption protocols.

The full security model for IBE was introduced in [Boneh and Franklin, 2003] and later extended to HIBE in [Gentry and Silverberg, 2002]. A weaker security model was introduced in [Canetti et al., 2003, Canetti et al., 2004] and is called the selective-ID model (s-ID model in short). The selective-ID differs from the full model by restricting the adversary to commit to the challenge identity even before setting up the protocol. The HIBE proposed by Boneh-Boyen [Boneh and Boyen, 2004], which we call BB-HIBE, and the BBG-HIBE [Boneh et al., 2005] are the only known HIBE protocols secure in the selective-ID model. In [Chatterjee and Sarkar, 2006a] the selective-ID security model has been generalised to two new models, $\boldsymbol{\mathcal{M}}_1$ and $\boldsymbol{\mathcal{M}}_2$, and the authors proposed two HIBEs $\mathcal{H}_1$ and $\mathcal{H}_2$ secure in the respective models. The BBG-HIBE has been extended to model $\boldsymbol{\mathcal{M}}_2$ in [Chatterjee and Sarkar, 2006b] and the authors also proposed a construction secure in the full model.

Our Contributions: We modify the BBG-HIBE to obtain a new constant-size ciphertext HIBE, $\mathcal{G}_1$. Compared to the BBG-HIBE, the new HIBE $\mathcal{G}_1$ has the following advantages – it is secure (without any degradation) in an extension of the s-ID model (see below) and the components of the identity tuples are from $\mathbb{Z}_p$, where p is a suitable large prime. On the other hand, the disadvantage is that the size of the public parameters and the private key is longer than that of the BBG-HIBE. Note that even though the size of the private key is longer, the size of the decryption subkey is the same as that of BBG-HIBE. Since for

decryption, only the decryption subkey needs to be loaded onto a smart card, to a certain extent this mitigates the disadvantage of the private key being longer.

In the s-ID model, the adversary commits to an identity $\mathsf{v}^* = (\mathsf{v}_1^*, \ldots, \mathsf{v}_m^*)$ and in the challenge phase obtains an encryption under v^*. In particular, the length m of the challenge identity is fixed by the adversary in the commit stage itself. In the augmented version of the selective-ID model, which we call *selective*$^+$-ID model, in the challenge phase, the adversary is allowed to ask for an encryption under $\mathsf{v}^+ = (\mathsf{v}_1^*, \ldots, \mathsf{v}_{m'}^*)$, where $1 \leq m' \leq m$. This provides the adversary additional flexibility in choosing the target identity.

In the s-ID model, the adversary is restricted from making private key queries for *any* prefix of v^*. Consequently, a "natural" intuition is that the adversary be allowed to choose any prefix of v^* as a challenge identity. Unfortunately, the s-ID model does not allow this flexibility to the adversary. In the s$^+$-ID model, this flexibility is introduced and the challenge identity is allowed to be any prefix of v^*. Clearly, any protocol secure in the s$^+$-ID model is also secure in the s-ID model, though the converse is not necessarily true.

We show that the security reduction for BB-HIBE [Boneh and Boyen, 2004] satisfies the notion of s$^+$-ID security. On the other hand, the security proof of the BBG-HIBE given in [Boneh et al., 2005] does not go through in the s$^+$-ID model. A simple modification of this proof gives a proof of security for the BBG-HIBE in the s$^+$-ID model. But this proof yields a multplicative security degradation by a factor of h, where h is the maximum number of levels in the HIBE.

Our idea of modifying the proof of the BBG-HIBE protocol can be utilised to show that any protocol secure in the s$^+$-ID model is also secure in the s-ID model with a security degradation by a factor of h. Admittedly, a security degradation by a factor of h is not much. However, the s-ID and the s$^+$-ID models are really restrictive models and hence one would like to obtain a protocol without any security degradation.

We next modify this construction to obtain a constant-size ciphertext HIBE, $\mathcal{G}_2$ which is proved to be secure in the generalised s-ID model $\mathcal{M}_2$ (see Section 4.2 and [Chatterjee and Sarkar, 2006a]) augmented in the line of s$^+$-ID model.

Our third construction is a product construction, in the sense that the constructed HIBE can be seen to be a "product" of two individual HIBEs. A product construction combining the BB-HIBE and the BBG-HIBE has been presented earlier in [Boneh et al., 2005].

We consider the product of $\mathcal{H}_1$ of [Chatterjee and Sarkar, 2006a] with $\mathcal{G}_2$ to obtain a new HIBE $\mathcal{G}_3$. This HIBE is secure in generalised s-ID model $\mathcal{M}_1$ (see Section 4.2 and [Chatterjee and Sarkar, 2006a]) and reduces the size of the ciphertext in $\mathcal{H}_1$ by a factor of h, where h is the number of levels in $\mathcal{G}_2$. The decryption subkey (i.e., the part of the private key required for decryption) for both $\mathcal{G}_1$ and $\mathcal{G}_2$ are equal to that of BBG-HIBE. While in $\mathcal{G}_3$ the size of the

decryption subkey is reduced by a factor of h over the size of the decryption subkeys in $\mathcal{H}_1$.

2 Definitions

2.1 Cryptographic Bilinear Map

Let G_1 and G_2 be cyclic groups of the same prime order p and such that $G_1 = \langle P \rangle$, i.e., P generates the group G_1, where we write G_1 additively and G_2 multiplicatively. A mapping $e : G_1 \times G_1 \to G_2$ is called a cryptographic bilinear map if it satisfies the following properties:

– Bilinearity: $e(aP, bQ) = e(P, Q)^{ab}$ for all $P, Q \in G_1$ and $a, b \in \mathbb{Z}_p$.

– Non-degeneracy: If $G_1 = \langle P \rangle$, then $G_2 = \langle e(P, P) \rangle$.

– Computability: There exists an efficient algorithm to compute $e(P, Q)$ for all $P, Q \in G_1$.

Since $e(aP, bP) = e(P, P)^{ab} = e(bP, aP)$, the map e also satisfies the symmetry property. The modified Weil pairing [Boneh and Franklin, 2003] and the Tate pairing [Barreto et al., 2002, Galbraith et al., 2002] are examples of cryptographic bilinear maps.

2.2 HIBE Protocol

Following [Horwitz and Lynn, 2002, Gentry and Silverberg, 2002] a hierarchical identity-based encryption (HIBE) scheme is specified by four algorithms: Setup, Key Generation, Encryption and Decryption. Note that, for a HIBE of height h (henceforth denoted as h-HIBE) any identity v is a tuple $(\mathsf{v}_1, \ldots, \mathsf{v}_\tau)$ where $1 \leq \tau \leq h$.

Setup: It takes as input a security parameter and returns the system parameters together with the master key. The system parameters include a description of the message space, the ciphertext space and the identity space. These are publicly known while the master key is known only to the private key generator (PKG).

Key Generation: It takes as input an identity $\mathsf{v} = (\mathsf{v}_1, \ldots, \mathsf{v}_\tau)$ and the private key $d_{\mathsf{v}|\tau-1}$ for the identity $(\mathsf{v}_1, \ldots, \mathsf{v}_{\tau-1})$ and returns a private key d_v for v. The identity v is used as the public key while d_v is the corresponding private key.

Encrypt: It takes as input the identity v and a message from the message space and produces a ciphertext in the cipher space.

Decrypt: It takes as input the ciphertext and a private key d_v of the corresponding identity v and returns the message or bad if the ciphertext is not valid.

2.3 Hardness Assumption

Security of our HIBE scheme is based on the so-called *decisional weak bilinear Diffie-Hellman inversion* problem (wDBDHI*) introduced by Boneh-Boyen-Goh in [Boneh et al., 2005]. An instance of the h-wDBDHI* problem in $\langle G_1, G_2, e\rangle$ consists of the tuple $(P, Q, aP, a^2P, \ldots, a^hP, Z)$ for some $a \in \mathbb{Z}_p$ and the task is to decide whether $Z = e(P,Q)^{a^{h+1}}$ or Z is random.

The advantage of a probabilistic algorithm $\mathcal{B}$ that outputs a bit in solving this decision problem is defined as

$$\mathsf{Adv}_{\mathcal{B}}^{h\text{-wDBDHI}^*} = \left|\Pr[\mathcal{B}(P, Q, \overrightarrow{Y}, e(P,Q)^{a^{h+1}}) = 1] - \Pr[\mathcal{B}(P, Q, \overrightarrow{Y}, Z) = 1]\right|$$

where $\overrightarrow{Y} = (aP, a^2P, \ldots, a^hP)$, and Z is a random element of G_2. The probability is calculated over the random choices of $a \in \mathbb{Z}_p$ and $Z \in G_2$ and also the random bits used by $\mathcal{B}$. The quantity $\mathsf{Adv}^{h\text{-wDBDHI}^*}(t)$ denotes the maximum of $\mathsf{Adv}_{\mathcal{B}}^{h\text{-wDBDHI}^*}$ where the maximum is taken over all algorithms running in time at most t.

3 Previous HIBE Constructions

We briefly describe the BB-HIBE and the BBG-HIBE. Let G_1, G_2 and e be as defined in Section 2.

3.1 BB-HIBE

Identities of depth u are of the form $(\mathsf{v}_1, \ldots, \mathsf{v}_u)$ where each $\mathsf{v}_i \in \mathbb{Z}_p$. Messages are elements of G_2. **Setup:** Select a random generator $P \in G_1^*$, a random $x \in \mathbb{Z}_p$ and set $P_1 = xP$. Also pick random elements $Q_1, \ldots, Q_h, P_2 \in G_1$. The public parameters are

$$(P, P_1, P_2, Q_1, \ldots, Q_h)$$

whereas the master secret key is xP_2. The maximum height of the HIBE is h. Define a publicly computable family of functions $F_j : \mathbb{Z}_p \to G_1$ for $j \in \{1, \ldots, h\}$: $F_j(\alpha) = \alpha P_1 + Q_j$.

Key Generation: Given an identity $\mathsf{v} = (\mathsf{v}_1, \ldots, \mathsf{v}_j)$ of depth $j \le h$, pick random $r_1, \ldots, r_j \in \mathbb{Z}_p$ and compute

$$d_{\mathsf{v}} = xP_2 + \sum_{i=1}^{j} r_i F_i(\mathsf{v}_i), r_1P, \ldots, r_jP.$$

Note that, d_{v} can also be generated given the private key $d_{\mathsf{v}|_{j-1}}$ of $\mathsf{v}|_{j-1} = (\mathsf{v}_1, \ldots, \mathsf{v}_{j-1})$.

Encrypt: Encrypt $M \in G_2$ for $\mathsf{v} = (\mathsf{v}_1, \ldots, \mathsf{v}_j)$ as

$$C = (e(P_1, P_2)^s \times M, sP, sF_1(\mathsf{v}_1), \ldots, sF_j(\mathsf{v}_j))$$

where s is a random element of $\mathbb{Z}_p$.

Decrypt: Decrypt $C = (A, B, C_1, \ldots, C_j)$ using the private key $d_{\mathsf{v}} = (d_0, d_1, \ldots, d_j)$ as

$$A \times \frac{\prod_{i=1}^{j} e(C_i, d_i)}{e(B, d_0)} = M$$

3.2 BBG-HIBE

Here, identities of depth u are of the form $(\mathsf{v}_1, \ldots, \mathsf{v}_u)$ where each $\mathsf{v}_i \in \mathbb{Z}_p^*$. (In contrast, recall that, in BB-HIBE identity components are elements of $\mathbb{Z}_p$). Messages are elements of G_2.

Setup: Choose a random $\alpha \in \mathbb{Z}_p$ and set $P_1 = \alpha P$. Choose random elements $P_2, P_3, Q_1, \ldots, Q_h \in G_1$. Set the public parameter as $(P, P_1, P_2, P_3, Q_1, \ldots, Q_h)$ while the master key is $P_4 = \alpha P_2$.

Key Generation: Given an identity $\mathsf{v} = (\mathsf{v}_1, \ldots, \mathsf{v}_k)$ of depth $k \leq h$, pick a random $r \in \mathbb{Z}_p$ and output

$$d_{\mathsf{v}} = (\alpha P_2 + r(\mathsf{v}_1 Q_1, \ldots, \mathsf{v}_k Q_k + P_3), rP, rQ_{k+1}, \ldots, rQ_h).$$

Encrypt: To encrypt $M \in G_2$ under the identity $\mathsf{v} = (\mathsf{v}_1, \ldots, \mathsf{v}_k)$, pick a random $s \in \mathbb{Z}_p$ and output

$$\mathsf{CT} = (e(P_1, P_2)^s \times M, sP, s(\mathsf{v}_1 Q_1 + \ldots + \mathsf{v}_k Q_k + P_3))\,.$$

Decrypt: To decrypt $\mathsf{CT} = (A, B, C)$ using the private key $d_{\mathsf{v}} = (a_0, a_1, b_{k+1}, \ldots, b_h)$, compute

$$A \times \frac{e(a_1, C)}{e(B, a_0)} = M.$$

4 Security Models

We now discuss variants of the selective-ID security models. The security of a HIBE protocol is defined in terms of a game between an adversary and a simulator. [Boneh and Franklin, 2003] introduced the full security model for IBE. Extension to HIBE was made by [Gentry and Silverberg, 2002]. The weaker selective-ID model was introduced in [Canetti et al., 2003, Canetti et al., 2004]. We define the selective identity, chosen-ciphertext security (IND-sID-CCA) of a HIBE of maximum height h, in terms of the following game.

4.1 Selective-ID Model

Initialization: The adversary outputs a target identity $\mathsf{v}^* = (\mathsf{v}_1^*, \ldots, \mathsf{v}_u^*)$ with $u \leq h$, on which it wishes to be challenged. **Setup:** The challenger sets up the HIBE and provides the adversary with the system public parameters.

Phase 1: Adversary $\mathcal{A}$ makes a finite number of queries where each query is addressed either to the decryption oracle or to the key-extraction oracle. In a query to the decryption oracle it provides the ciphertext as well as the identity under which it wants the decryption. Similarly, in a query to the key-extraction oracle, it asks for the private key of the identity it provides. Further, $\mathcal{A}$ is allowed to make these queries adaptively, i.e., any query may depend on the previous queries as well as their answers. The only restriction is that it cannot ask for the private key of v^* or any of its ancestors such as $(\mathsf{v}_1^*, \ldots, \mathsf{v}_j^*)$, $j < u$.

Challenge: At this stage $\mathcal{A}$ outputs two equal length messages M_0, M_1 and gets a ciphertext C^* which is an encryption of M_γ under v^*, where γ is chosen uniformly at random from $\{0, 1\}$.

Phase 2: $\mathcal{A}$ now issues additional queries just like Phase 1, with the (obvious) restriction that it cannot ask the decryption oracle for the decryption of C^* under v^* nor the key-extraction oracle for the private key of any prefix of v^*.

Guess: $\mathcal{A}$ outputs a guess γ' of γ.

The advantage of the adversary $\mathcal{A}$ in attacking the HIBE scheme is defined as:

$$\mathsf{Adv}_{\mathcal{A}}^{\mathsf{HIBE}} = |\Pr[(\gamma = \gamma')] - 1/2| .$$

The quantity $\mathsf{Adv}^{\mathsf{HIBE}}(t, q_{\mathsf{ID}}, q_{\mathsf{C}})$ denotes the maximum of $\mathsf{Adv}_{\mathcal{A}}^{\mathsf{HIBE}}$ where the maximum is taken over all adversaries running in time at most t and making at most q_{C} queries to the decryption oracle and q_{ID} queries to the key-extraction oracle. Any HIBE scheme secure against such an adversary is said to be secure against chosen-ciphertext attack (in short, IND-sID-CCA-secure). We may restrict the adversary from making any query to the decryption oracle. A HIBE protocol secure against such an adversary is said to be secure against chosen-plaintext attacks (in short, IND-sID-CPA-secure). $\mathsf{Adv}^{\mathsf{HIBE}}(t, q)$ in this context denotes the maximum advantage where the maximum is taken over all adversaries running in time at most t and making at most q queries to the key-extraction oracle.

Generic [Canetti et al., 2004, Boneh and Katz, 2005] as well as non-generic techniques [Boyen et al., 2005] are known for converting a CPA-secure HIBE to a CCA-secure HIBE. In view of this, it is more convenient to initially construct a CPA-secure HIBE and then convert it into a CCA-secure one.

4.2 Generalised Selective-ID Model

[Chatterjee and Sarkar, 2006a] introduced two new security models, $\mathcal{M}_1$ and $\mathcal{M}_2$. Here we briefly describe these two models.

In $\boldsymbol{\mathcal{M}}_1$ the adversary fixes a set of target identities $\mathcal{I}^*$ before the protocol is set up where $|\mathcal{I}^*| = n$. In Phase 1 and 2 the adversary cannot make any query to the key extraction oracle for the private key of an identity tuple v all of whose components are in $\mathcal{I}^*$. On the other hand, in the Challenge stage it must ask for encryption under an identity tuple v^* all of whose components are in $\mathcal{I}^*$. This model is parametrised by the maximum height h of the HIBE and n. This is explicitly written as (h, n)-$\boldsymbol{\mathcal{M}}_1$ model.

$\boldsymbol{\mathcal{M}}_2$ generalises the s-ID model in the following manner. Before the set-up of the protocol, the adversary commits to sets of identities $\mathcal{I}_1^*, \ldots, \mathcal{I}_\tau^*$, where $1 \le \tau \le h$ and h is the maximum number of levels of the HIBE. Let $|\mathcal{I}_i^*| = n_i$. The adversary's commitment fixes the length of the challenge identity to be τ. Also, the set $\mathcal{I}_i^*$ corresponds to the set of committed identities for the ith level of the HIBE.

In Phases 1 and 2, the adversary is not allowed to query the key extraction oracle on any identity $(\mathsf{v}_1, \ldots, \mathsf{v}_j)$ such that $j \le \tau$ and $\mathsf{v}_i \in \mathcal{I}_i^*$ for all $1 \le i \le j$. The challenge identity is a tuple $(\mathsf{v}_1^*, \ldots, \mathsf{v}_\tau^*)$ where $\mathsf{v}_i^* \in \mathcal{I}_i^*$ for all $1 \le i \le \tau$.

The model $\boldsymbol{\mathcal{M}}_2$ is parametrized by h and a tuple $(n_1, \ldots, n_h)$ of positive integers. This is explictly written as $(h, n_1, \ldots, n_h)$-$\boldsymbol{\mathcal{M}}_2$ model. This model is a generalization of the s-ID-model which can be seen by fixing all the $\mathcal{I}_i^*$s to be singleton sets. More specifically, $(h, 1, \ldots, 1)$-$\boldsymbol{\mathcal{M}}_2$ is the s-ID-model.

4.3 Selective$^+$-ID Model

We modify the challenge phase of the selective-ID model to give more power to the adversary. **Challenge:** $\mathcal{A}$ outputs two equal length messages M_0, M_1 and an identity v^+ where v^+ is either v^* or any of its prefixes. In response it receives an encryption of M_γ under v^+, where γ is chosen uniformly at random from $\{0, 1\}$.

We refer to this new model as the selective$^+$-ID model (s$^+$-ID model in short). This model is more general than the s-ID model because now the adversary is allowed to ask for a challenge ciphertext not only on v^* but also on any of its prefixes. In case of IBE both the models are same as we have only one level. For HIBE, a protocol secure in the selective$^+$-ID model is obviously secure in the selective-ID model.

5 Constant Size Ciphertext HIBE Secure in Selective$^+$-ID Model

We augment the BBG-HIBE to obtain a new constant-size ciphertext HIBE secure in the *selective*$^+$-ID model without any security degradation. We call this new protocol $\mathcal{G}_1$. The basic idea is to replace P_3 in BBG-HIBE by a vector $\overrightarrow{P}_3 = (P_{3,1}, \ldots, P_{3,h})$ where $P_{3,i}$ corresponds to the ith level of the HIBE. It is this feature that allows identity components to be elements of $\mathbb{Z}_p$ and a proof

(without security degradation) in the s^+-ID model. Also, it is this feature which increases the size of the public parameters and the private key.

Let G_1, G_2 and e be as defined in Section 2. Let the maximum height of the HIBE be h. The identities at a depth $u \le h$ are of the form $\mathsf{v} = (\mathsf{v}_1, \ldots, \mathsf{v}_u)$ where each $\mathsf{v}_i \in \mathbb{Z}_p$. Note that, unlike the BBG-HIBE, we allow 0 as a valid identity component. Messages are elements of G_2.

Setup: Choose a random $\alpha \in \mathbb{Z}_p$ and set $P_1 = \alpha P$. Choose a random element $P_2 \in G_1$ and two random h length vectors $\overrightarrow{P}_3, \overrightarrow{Q}$ where $\overrightarrow{P}_3 = (P_{3,1}, \ldots, P_{3,h})$ and $\overrightarrow{Q} = (Q_1, \ldots, Q_h)$. Set the public parameters to be $(P, P_1, P_2, \overrightarrow{P}_3, \overrightarrow{Q})$ while the master key is $P_4 = \alpha P_2$. Instead of P_1, P_2, $e(P_1, P_2)$ can also be kept as part of PP. This avoids the pairing computation during encryption.

Key Generation: Given an identity $\mathsf{v} = (\mathsf{v}_1, \ldots, \mathsf{v}_k)$ of depth $k \le h$, pick a random $r \in \mathbb{Z}_p$ and output

$$d_{\mathsf{v}} = \left(\alpha P_2 + r \sum_{j=1}^{k} V_j,\ rP,\ rP_{3,k+1}, \ldots, rP_{3,h},\ rQ_{k+1}, \ldots, rQ_h\right)$$

where $V_j = P_{3,j} + \mathsf{v}_j Q_j$. The private key at level k consists of $2(h-k+1)$ elements of G_1. Among these $2(h-k+1)$ elements only the first two are required in decryption, the rest are used to generate the private key for the next level as follows: Let the secret key corresponding to the identity $(\mathsf{v}_1, \ldots, \mathsf{v}_{k-1})$ be $(A_0, A_1, B_k, \ldots, B_h, C_k, \ldots, C_h)$, where $A_0 = \alpha P_2 + r' \sum_{j=1}^{k-1} V_j$, $A_1 = r'P$, and for $k \le j \le h$, $B_j = r' P_{3,j}$, $C_j = r' Q_j$. Pick a random $r^* \in \mathbb{Z}_p$ and compute

$$\begin{aligned} d_{\mathsf{v}} = (&A_0 + B_k + \mathsf{v}_k C_k + r^* \textstyle\sum_{j=1}^{k} V_k,\ A_1 + r^* P, \\ &B_{k+1} + r^* P_{3,k+1}, \ldots, B_h + r^* P_{3,h}, \\ &C_{k+1} + r^* Q_{k+1}, \ldots, C_h + r^* Q_h). \end{aligned}$$

If we put $r = r' + r^*$, then d_{v} is a proper private key for $\mathsf{v} = (\mathsf{v}_1, \ldots, \mathsf{v}_k)$.

Encrypt: To encrypt $M \in G_2$ under the identity $(\mathsf{v}_1, \ldots, \mathsf{v}_k)$, pick a random $s \in \mathbb{Z}_p$ and output

$$\mathsf{CT} = \left(e(P_1, P_2)^s \times M, sP, s\left(\sum_{j=1}^{k} V_j\right)\right)$$

where V_j is as defined in Key Generation.

Decrypt: To decrypt $\mathsf{CT} = (A, B, C)$ using the private key $d_{\mathsf{v}} = (d_0, d_1, \ldots)$, compute

$$A \times \frac{e(d_1, C)}{e(B, d_0)} = e(P_1, P_2)^s \times M \times \frac{e\left(rP, s\sum_{j=1}^{k} V_j\right)}{e\left(sP, \alpha P_2 + r\sum_{j=1}^{k} V_j\right)} = M.$$

5.1 Discussion

The protocol $\mathcal{G}_1$ is a modification of the BBG-HIBE with a different $P_{3,i}$ for each level of the HIBE. This is required to get a proof of security in the augmented s$^+$-ID model without any security degradation as is shown in the next section. Additionally, it allows identities to be elements of $\mathbb{Z}_p$, instead of $\mathbb{Z}_p^*$ as in BBG-HIBE. On the other hand, this modification only affects the efficiency of the BBG-HIBE in a small way. The first thing to note is the size of the ciphertext is still constant (three elements). Secondly, the size of the public parameter as well as private key is linear in the length of the HIBE and decreases as we "go down" the HIBE. These two properties ensure that the applications mentioned in [Boneh et al., 2005] also hold for the new HIBE described above. In particular, it is possible to combine the new HIBE with the BB-HIBE of [Boneh and Boyen, 2004] to get an intermediate HIBE with controllable trade-off between the size of the ciphertext and the size of the private key. Further, the application to the construction of forward secure encryption protocol mentioned in [Boneh et al., 2005] can also be done with the new HIBE. The resulting protocols will be secure in the augmented selective$^+$-ID model. However, the actual details for these applications will be a little different from what is mentioned in [Boneh et al., 2005].

A comparison of the features of $\mathcal{G}_1$ with the BB-HIBE and the BBG-HIBE is given in Table 1 for h-level HIBEs. Here the column "decryption subkey size" denotes the number of elements of the private key which is actually required for decryption. The entire private key is required for key delegation, i.e., generating private keys for the lower level identities , which is a relatively infrequent activity. As mentioned above, the BBG-HIBE has many applications. The modified protocol $\mathcal{G}_1$ can be used for all such applications.

6 Security

Semantic security (i.e., (CPA-security) of the above scheme in the s$^+$-ID model is proved under the h-wDBDHI* assumption.

THEOREM 6.1. *For* $t \geq 1, q \geq 1; \mathsf{Adv}^{\mathcal{G}_1}(t,q) \leq \mathsf{Adv}^{h\text{-wDBDHI}^*}(t + O(\tau q))$, *where* τ *is the time for a scalar multiplication in* G_1.

Proof : Suppose $\mathcal{A}$ is a (t,q)-CPA adversary for the $\mathcal{G}_1$, then we construct an algorithm $\mathcal{B}$ that solves the h-wDBDHI* problem. $\mathcal{B}$ takes as input a tuple $(P, Q, Y_1, \ldots, Y_h, Z)$ where $Y_i = \alpha^i P$ for some random $\alpha \in \mathbb{Z}_p^*$ and Z is either equal to $e(P,Q)^{\alpha^{h+1}}$ or a random element of G_2. We define the s$^+$-ID game between $\mathcal{B}$ and $\mathcal{A}$ as follows.

Initialization: $\mathcal{A}$ outputs an identity tuple $\mathsf{v}^* = (\mathsf{v}_1^*, \ldots, \mathsf{v}_u^*) \in \mathbb{Z}_p^u$ for any $u \leq h$. The restriction on $\mathcal{A}$ is that it cannot ask for the private key of v^* or

Table 1: Comparison of HIBE protocols Secure in s-ID/s$^+$-ID Model.

protocol	security model	id comp	public parameter	max pvt key size	decryption subkey size
$\mathcal{G}_1$	s$^+$-ID	$\mathbb{Z}_p$	$3+2h$	$2h$	2
BBG	s$^+$-ID	$\mathbb{Z}_p^*$	$4+h$	$h+1$	2
BBG	s-ID	$\mathbb{Z}_p^*$	$4+h$	$h+1$	2
BB	s$^+$-ID	$\mathbb{Z}_p$	$3+h$	$h+2$	$h+2$

protocol	ciphertext expansion	encryption efficiency	decryption efficiency	Security degradation
$\mathcal{G}_1$	2	$h+2$	2	Nil
BBG in s$^+$-ID	2	$h+2$	2	h
BBG in s-ID	2	$h+2$	2	Nil
BB	$h+1$	$2h+1$	$h+1$	Nil

For a HIBE of maximum height h, the columns for public parameter, max pvt key size, decryption subkey size and ciphertext expansion denote the number of elements of G_1, encryption efficiency denotes the number of scalar multiplications in G_1 and decryption efficiency denotes the number of pairing computations.

any of its prefix and in challenge it asks for an encryption under v^* or any of its prefix. In case $u < h$, $\mathcal{B}$ chooses random $\mathsf{v}^*_{u+1}, \ldots, \mathsf{v}^*_h$ from $\mathbb{Z}_p$ and keeps these extra elements to itself. (Note that $\mathcal{B}$ is *not* augmenting the target identity to create a new target identity.)

Setup: $\mathcal{B}$ picks random β, $\beta_1, \ldots, \beta_h$ and $c_1, \ldots, c_h$ in $\mathbb{Z}_p$. It then sets

$$P_1 = Y_1 = \alpha P; P_2 = Y_h + \beta P = (\alpha^h + \beta)P;$$

and

$$\begin{array}{lll} \text{for } 1 \le j \le u, & Q_j = \beta_j P - Y_{h-j+1}; & P_{3,j} = c_j P + \mathsf{v}^*_j Y_{h-j+1}; \\ \text{for } u < j \le h, & Q_j = \beta_j P; & P_{3,j} = c_j P + \mathsf{v}^*_j Y_{h-j+1}. \end{array}$$

The public parameters are $(P, P_1, P_2, \overrightarrow{P}_3, \overrightarrow{Q})$, where $\overrightarrow{Q} = (Q_1, \ldots, Q_h)$, $\overrightarrow{P}_3 = (P_{3,1}, \ldots, P_{3,h})$. The corresponding master key $\alpha P_2 = Y_{h+1} + \beta Y_1$ is unknown to $\mathcal{B}$. $\mathcal{B}$ defines the functions $F_j = \mathsf{v}^*_j - \mathsf{v}_j$ for $1 \le j \le u$ and $F_j = \mathsf{v}^*_j$ for $u < j \le h$ and $J_j = c_j + \beta_j \mathsf{v}_j$ for $1 \le j \le h$.

Phase 1: Suppose $\mathcal{A}$ asks for the private key corresponding to an identity $\mathsf{v} = (\mathsf{v}_1, \ldots, \mathsf{v}_\tau)$ for $\tau \le h$. Note that for any $j \le u$,

$$\begin{aligned} V_j &= P_{3,j} + \mathsf{v}_j Q_j \\ &= c_j P + \mathsf{v}^*_j Y_{h-j+1} + \mathsf{v}_j(\beta_j P - Y_{h-j+1}) \end{aligned}$$

$$= (\mathsf{v}_j^* - \mathsf{v}_j)Y_{h-j+1} + (c_j + \beta_j \mathsf{v}_j)P$$
$$= F_j Y_{h-j+1} + J_j P.$$

Similarly, for $u < j \leq h$

$$V_j = P_{3,j} + \mathsf{v}_j Q_j = c_j P + \mathsf{v}_j^* Y_{h-j+1} + \mathsf{v}_j \beta_j P = F_j Y_{h-j+1} + J_j P.$$

Hence, V_j for $1 \leq j \leq h$ is computable from what is known to $\mathcal{B}$.

Recall that u is the length of v^* that the adversary committed to before the set-up phase. If $\tau \leq u$, then there must be a $k \leq \tau$ such that $F_k \neq 0$, as otherwise the queried identity is a prefix of the target identity. In case $\tau > u$, it is possible that $F_1 = \cdots = F_u = 0$. Then by construction, $F_{u+1} \neq 0$. We now proceed under the assumption that there is a k such that $F_k \neq 0$ and k is the smallest such index. $\mathcal{B}$ picks a random $r \in \mathbb{Z}_p$ and assigns $d_{0|k} = (-J_k/F_k)Y_k + \beta Y_1 + rV_k$ and $d_1 = (-1/F_k)Y_k + rP$. Now,

$$d_{0|k} = -\frac{J_k}{F_k} Y_k + \beta Y_1 + \alpha^k Y_{h-k+1} - \alpha^k \frac{F_k}{F_k} Y_{h-k+1} + rV_k = \alpha P_2 + \tilde{r} V_k$$

where $\tilde{r} = (r - \frac{\alpha^k}{F_k})$. Also $d_1 = -\frac{1}{F_k} Y_k + rP = -\frac{\alpha^k}{F_k} P + rP = \tilde{r} P$. For any $j \in \{1, \ldots, \tau\} \setminus \{k\}$ we have

$$\tilde{r} V_j = (r - \frac{\alpha^k}{F_k})(F_j Y_{h-j+1} + J_j P)$$
$$= r(F_j Y_{h-j+1} + J_j P) - \frac{1}{F_k}(F_j Y_{h+k-j+1} + J_j Y_k).$$

For $j < k$, $F_j = 0$, so $\mathcal{B}$ can compute all these $\tilde{r}V_j$s from what it has. It forms

$$d_0 = d_{0|k} + \sum_{j \in \{1,\ldots,\tau\} \setminus \{k\}} \tilde{r} V_j = \alpha P_2 + \tilde{r} \sum_{j=1}^{\tau} V_j.$$

To form a valid private key $\mathcal{B}$ also needs to compute $\tilde{r} P_{3,j}$ and $\tilde{r} Q_j$ for $\tau < j \leq h$. Now,

$$\tilde{r} P_{3,j} = \left(r - \frac{\alpha^k}{F_k}\right)(c_j P + \mathsf{v}_j^* Y_{h-j+1})$$
$$= r(c_j P + \mathsf{v}_j^* Y_{h-j+1}) - \frac{1}{F_k}\left(c_j Y_k + \mathsf{v}_j^* Y_{h+k-j+1}\right);$$

For $j \leq u$,

$$\tilde{r} Q_j = \left(r - \frac{\alpha^k}{F_k}\right)(\beta_j P - Y_{h-j+1}) = r(\beta_j P - Y_{h-j+1}) - \frac{1}{F_k}(\beta_j Y_k - Y_{h+k-j+1})$$

and for $u < j \leq h$,

$$\tilde{r} Q_j = \left(r - \frac{\alpha^k}{F_k}\right)\beta_j P = r\beta_j P - \frac{1}{F_k}\beta_j Y_k.$$

All these values are computable from what is known to $\mathcal{B}$. Hence, $\mathcal{B}$ forms the private key as:

$$d_{\mathsf{v}} = (d_0, d_1, \tilde{r}P_{3,\tau+1}, \ldots, \tilde{r}P_{3,h}, \tilde{r}Q_{\tau+1}, \ldots, \tilde{r}Q_h)$$

and provides it to $\mathcal{A}$.

Challenge: After completion of Phase 1, $\mathcal{A}$ outputs two messages $M_0, M_1 \in G_2$ and the challenge identity $\mathsf{v}^+ = \mathsf{v}_1^*, \ldots, \mathsf{v}_{u'}^*$ where $u' \leq u \leq h$. $\mathcal{B}$ picks a random $b \in \{0,1\}$ and provides $\mathcal{A}$ the challenge ciphertext

$$\mathsf{CT} = \left(M_b \times T \times e(Y_1, \beta Q),\ Q,\ \left(\sum_{j=1}^{u'} (c_j + \beta_j \mathsf{v}_j^*) \right) \times Q \right).$$

Suppose, $Q = \gamma P$ for some unknown $\gamma \in \mathbb{Z}_p$. Then

$$\begin{aligned}
\left(\sum_{j=1}^{u'} c_j + \beta_j \mathsf{v}_j^* \right) \times Q &= \gamma \left(\sum_{j=1}^{u'} c_j + \beta_j \mathsf{v}_j^* \right) P \\
&= \gamma \sum_{j=1}^{u'} \left(c_j P + \mathsf{v}_j^* Y_{h-j+1} + \mathsf{v}_j^* (\beta_j P - Y_{h-j+1}) \right) \\
&= \gamma \sum_{j=1}^{u'} \left(P_{3,j} + \mathsf{v}_j^* Q_j \right) \\
&= \gamma \left(\sum_{j=1}^{u'} V_j \right).
\end{aligned}$$

If the input provided to $\mathcal{B}$ is a true h-wDBDHI* tuple, i.e., $Z = e(P,Q)^{(\alpha^{h+1})}$, then

$$Z \times e(Y_1, \beta Q) = e(P,Q)^{(\alpha^{h+1})} \times e(Y_1, \beta Q) = e(Y_h + \beta P, Q)^{\alpha} = e(P_1, P_2)^{\gamma}.$$

So, the challenge ciphertext is

$$\mathsf{CT} = \left(M_b \times e(P_1, P_2)^{\gamma}, \gamma P, \gamma \left(\sum_{j=1}^{u'} V_j \right) \right).$$

CT is a valid encryption of M_b under $\mathsf{v}^+ = (\mathsf{v}_1^*, \ldots, \mathsf{v}_{u'}^*)$. On the other hand, when Z is random, CT is random from the view point of $\mathcal{A}$.

Phase 2: This is similar to Phase 1. Note that $\mathcal{A}$ places at most q queries in Phase 1 and 2 together.

Guess: Finally, $\mathcal{A}$ outputs its guess $b' \in \{0,1\}$. $\mathcal{B}$ outputs $1 \oplus b \oplus b'$.

$\mathcal{A}$'s view in the above simulation is identical to that in a real attack. This gives us the required bound on the advantage of the adversary in breaking the HIBE protocol. ■

7 More on the Selective$^+$-ID Model

We analyse the BB-HIBE and the BBG-HIBE with respect to the s$^+$-ID model. It is easy to show that the BB-HIBE is secure in the s$^+$-ID model without any security degradation. The details are given in Section A. The case of BBG-HIBE is more interesting and is discussed below.

7.1 Boneh-Boyen-Goh HIBE

The BBG-HIBE is proved to be secure in the s-ID model (Theorem 3.1 of [Boneh et al., 2005]). We first argue that the given proof is not sufficient for the s$^+$-ID model. Using the intuition developed in the argument, we later sketch a proof of security for the BBG-HIBE in the s$^+$-ID model, though with a multiplicative security degradation by a factor of h.

In the s-ID model, an adversary declares an identity v^* that it intends to attack before the system is set up. Suppose $\mathsf{v}^* = (\mathsf{v}_1^*, \ldots, \mathsf{v}_m^*)$ where $m \leq h$. In the reduction given in [Boneh et al., 2005], the following is done. If $m < h$ then the simulator appends $(h - m)$ zeros to v^* so that v^* is a vector of length h. Recall that, in the protocol, individual comonents of an identity are elements of $\mathbb{Z}_p^*$ so the adversary is restricted from making a query where one or more components of the identity is 0. (BB-HIBE does not have this restriction.) The reduction in [Boneh et al., 2005] crucially depends on this step.

In the protocol, a single element of G_1 (i.e. Q_i) is associated with the ith level of the HIBE and we have another element, namely P_3 which is required for the security reduction.

The simulator $\mathcal{B}$ is given as input a random tuple $(P, Q, Y_1, \ldots, Y_h, T)$ where $Y_i = \alpha^i P$s for $1 \leq i \leq h$ for some unknown α. The task of $\mathcal{B}$ is to decide whether $T = e(P, Q)^{\alpha^{h+1}}$ or T is a random element of G_2.

We now reproduce the relevant steps of the reduction in Theorem 3.1 in [Boneh et al., 2005].

Setup: $\mathcal{B}$ picks a random $\gamma \in \mathbb{Z}_p$ and sets $P_1 = Y_1 = \alpha P$ and $P_2 = Y_h + \gamma P$. Next, $\mathcal{B}$ picks random $\gamma_1, \ldots, \gamma_h \in \mathbb{Z}_p$ and sets $Q_j = \gamma_j P - Y_{h-j+1}$ for $j = 1, \ldots, h$. $\mathcal{B}$ also picks a random $\delta \in \mathbb{Z}_p$ and sets $P_3 = \delta P + \sum_{j=1}^{h} \mathsf{v}_j^* Y_{h-j+1}$. $\mathcal{B}$ gives $\mathcal{A}$ the public parameters $(P, P_1, P_2, P_3, Q_1, \ldots, Q_h)$.

Note that, the effect of $\mathsf{v}^* = (\mathsf{v}_1^*, \ldots, \mathsf{v}_m^*)$ is assimilated in P_3. In case, m (the depth of the challenge identity tuple v^*) is less than h, we have $\mathsf{v}_{m+1}^* = \cdots = \mathsf{v}_h^* = 0$, so $\mathsf{v}_j^* Y_{h-j+1}$ for $m < j \leq h$ has no effect on P_3. The Q_js in the public parameter are independent of the target identity and depend only on the Y_js after suitable randomization. In contrast, in case of the BB-HIBE, each Q_j depends on v_j^* i.e., the component corresponding to level j in target identity v^*.

Given this setup, Boneh, Boyen and Goh show that all the private key queries of $\mathcal{A}$ can be answered (see Phase 1 in the proof of Theorem 3.1 in

[Boneh et al., 2005] for details).

Now, suppose in the challenge phase $\mathcal{A}$ asks the encryption under v^+ which is a prefix of v^*, i.e., $\mathsf{v}^+ = (\mathsf{v}_1^*, \ldots, \mathsf{v}_\mu^*)$, $\mu \le m$. If $\mu = m$, then the original reduction goes through and we get a proper encryption of M_b provided the input instance is a true h-wDBDHI* instance. However, if $\mu < h$, then the original reduction in [Boneh et al., 2005] *does not* give a proper encryption of M_b even if the input is a true h-wDBDHI* instance as we show below.

Let $Q = cP$ for some unknown $c \in \mathbb{Z}_p$, then the third component of the challenge ciphertext is

$$C = \left(\delta + \sum_{j=1}^{h} \mathsf{v}_j^* \gamma_j\right) Q = c\left(\sum_{j=1}^{h} \mathsf{v}_j^*(\gamma_j P - Y_{h-j+1}) + \delta P + \sum_{j=1}^{h} \mathsf{v}_j^* Y_{h-j+1}\right)$$
$$= c(\mathsf{v}_1^* Q_1 + \ldots, \mathsf{v}_m^* Q_m + P_3) \text{ since } \mathsf{v}_{m+1}^* = \cdots = \mathsf{v}_h^* = 0$$

However, this corresponds to an encryption under v^* and *not* v^+. To get a valid encryption under $\mathsf{v}^+ = \mathsf{v}_1^*, \ldots, \mathsf{v}_\mu^*$, the third component of the ciphertext should be of the form

$$\begin{aligned} C' &= c(\mathsf{v}_1^* Q_1 + \cdots + \mathsf{v}_\mu^* Q_\mu + P_3) \\ &= c\left(\sum_{j=1}^{\mu} \mathsf{v}_j^*(\gamma_j P - Y_{h-j+1}) + \delta P + \sum_{j=1}^{h} \mathsf{v}_j^* Y_{h-j+1}\right) \\ &= c\left(\sum_{j=1}^{\mu} \mathsf{v}_j^* \gamma_j P + \delta P + \sum_{j=\mu+1}^{m} \mathsf{v}_j^* Y_{h-j+1}\right) \\ &= \left(\delta + \sum_{j=1}^{\mu} \mathsf{v}_j^* \gamma_j\right) Q + c \sum_{j=\mu+1}^{m} \mathsf{v}_j^* Y_{h-j+1} \end{aligned}$$

This C' cannot be computed by $\mathcal{B}$ without the knowledge of c. To see this note that $\mathcal{B}$ knows Q, the Y's, the γ's and δ. If $\mathcal{B}$ can compute C' then using the knowledge of these quantities, $\mathcal{B}$ can also compute c.

A difference in the BB-HIBE and the BBG-HIBE is that in the former, components of identities are elements of $\mathbb{Z}_p$, whereas in the later the identity components are elements of $\mathbb{Z}_p^*$. It is an easy observation that if zero is allowed to be an identity component, then the BBG-HIBE is not secure. A sketch of the argument is as follows. In the s-ID game, an adversary has to commit to an identity before the HIBE is set-up. Let adersary $\mathcal{A}$ commit to an identity $\mathsf{v}^* = (\mathsf{v}_1^*, \ldots, \mathsf{v}_k^*)$ for some k with $1 \le k < h$. In the query phase, $\mathcal{A}$ issues a private key query for the identity $\mathsf{v} = (\mathsf{v}_1^*, \ldots, \mathsf{v}_k^*, 0)$ which is a valid query *if* 0 is allowed. In return, $\mathcal{A}$ is provided the private key of $d_{\mathsf{v}} = (d_0, d_1, \ldots)$. Then $d_0 = \alpha P_2 + r(\mathsf{v}_1^* Q_1, \ldots, \mathsf{v}_k^* Q_k + 0 \cdot Q_{k+1} + P_3)$ and $d_1 = rP$ for some random

$r \in \mathbb{Z}_p$. Using (d_0, d_1), $\mathcal{A}$ can decrypt any message encrypted for v^*. Removing 0 from the identity space avoids this situation and allows a proof of the BBG-HIBE in the s-ID model.

7.2 Modified Security Reduction for the BBG-HIBE.

We modify the security reduction of BBG-HIBE in the following way. Suppose, as before that the adversary committed to an identity tuple $\mathsf{v}^* = (\mathsf{v}_1^*, \ldots, \mathsf{v}_m^*)$ in the commitment stage. During setup, $\mathcal{B}$ choses a random μ from $\{1, \ldots, m\}$ and forms the public parameters as in the original reduction of [Boneh et al., 2005] *assuming* that $\mathsf{v}^+ = (\mathsf{v}_1^*, \ldots \mathsf{v}_\mu^*)$ will be the target identity in challenge stage. This means that during setup, the simulator augments v^+ by appending zeros and forming a tuple of length h.

The above change does not affect the simulator's ability to answer key-extraction queries. During the challenge phase, the simulator can form a proper encryption *only if* the target identity tuple is v^+. The actual target identity submitted by the adversary has to be a prefix of v^*. If this is not equal to v^+, the simulator aborts the game and outputs one with probability half. Otherwise, it returns a proper challenge ciphertext as in the original reduction in [Boneh et al., 2005].

Since, $1 \leq \mu \leq m \leq h$ and μ is chosen uniformly at random, we have $\Pr[\overline{\mathsf{abort}}] \geq 1/h$. This leads to a multiplicative degradation by a factor of h, i.e., $\epsilon \leq h\epsilon'$, where ϵ is the maximum advantage of attacking the BBG-HIBE and ϵ' is the maximum advantage of solving h-wDBDHI*.

7.3 Passing From s-ID model to the s$^+$-ID model.

It is not difficult to see that the idea of modifying the proof of the BBG-HIBE protocol to attain security in the s$^+$-ID model is quite general. This idea does not depend upon the particular algebraic construction of the BBG-HIBE and hence can be applied to any HIBE which is secure in the s-ID model. Thus, any HIBE which is secure in the s-ID model is also secure in the s$^+$-ID model but with a security degradation by a factor of h. Though small, in certain cases this can be avoided, e.g., the BB-HIBE and $\mathcal{G}_1$ as shown earlier. The other issue is that the s-ID and the s$^+$-ID models are really restrictive security models and it would be nice to obtain tight security reductions in these models.

8 Augmenting to $\mathcal{M}_2^+$

Like the augmentation of the *selective*-ID model to selective$^+$-ID model, we can augment $\mathcal{M}_2$ proposed in [Chatterjee and Sarkar, 2006a] in an obvious way to $\mathcal{M}_2^+$. Suppose the adversary of an h-HIBE has committed to a set of target

identities, $\mathcal{I}_1^*, \ldots, \mathcal{I}_u^*$ where $u \leq h$. Then in the challenge phase it outputs a target identity $\mathsf{v}_1^*, \ldots, \mathsf{v}_{u'}^*$ where $1 \leq u' \leq u$ and each $\mathsf{v}_j^* \in \mathcal{I}_j^*$.

The HIBE $\mathcal{H}_2$ proposed in [Chatterjee and Sarkar, 2006a] is also secure in $\boldsymbol{\mathcal{M}}_2^+$. ccHIBE of [Chatterjee and Sarkar, 2006b] secure in $\boldsymbol{\mathcal{M}}_2$ can be proved to be secure in $\boldsymbol{\mathcal{M}}_2^+$ with a multiplicative security degradation of h. Here, we show how $\mathcal{G}_1$ can be augmented to $\boldsymbol{\mathcal{M}}_2^+$.

8.1 Construction

We augment $\mathcal{G}_1$ to obtain security in model $\boldsymbol{\mathcal{M}}_2^+$ and call this new protocol $(h, n_1, \ldots, n_h)$-$\mathcal{G}_2$ or simply $\mathcal{G}_2$.

The maximum height of the HIBE be h. The identities at a depth $u \leq h$ are of the form $\mathsf{v} = (\mathsf{v}_1, \ldots, \mathsf{v}_u) \in (\mathbb{Z}_p)^u$. Messages are elements of G_2.

Setup: Let $\langle P \rangle = G_1$. Choose a random $\alpha \in \mathbb{Z}_p$ and set $P_1 = \alpha P$. Choose a random element $P_2 \in G_1$ and a random h length vector $\overrightarrow{P}_3 = (P_{3,1}, \ldots, P_{3,h})$, where each $P_{3,i} \in G_1$. Also choose random vectors $\overrightarrow{Q}_1, \ldots, \overrightarrow{Q}_h$ where each $\overrightarrow{Q}_i$ consists of n_i elements of G_1. Set the public parameter as

$$\mathsf{PP} = (P, P_1, P_2, \overrightarrow{P}_3, \overrightarrow{Q}_1, \ldots, \overrightarrow{Q}_h)$$

while the master key is $P_4 = \alpha P_2$. Instead of P_1, P_2, $e(P_1, P_2)$ can also be kept as part of PP. This avoids the pairing computation during encryption.

Note that, while the original BBG scheme and
ccHIBE of [Chatterjee and Sarkar, 2006b] had a single element P_3 in the public parameter, we have a vector $\overrightarrow{P}_3$ of length h.

Key-Gen: Let, $V(i, y) = y^{n_i} Q_{i,n_i} + \cdots + y Q_{i,1}$ for any $y \in \mathbb{Z}_p$. Given an identity $\mathsf{v} = (\mathsf{v}_1, \ldots, \mathsf{v}_k) \in \mathbb{Z}_p^k$ of depth $k \leq h$, pick a random $r \in \mathbb{Z}_p$ and output

$$d_{\mathsf{v}} = \left(\alpha P_2 + r \sum_{j=1}^{k} V_j,\ rP,\ rP_{3,k+1}, \ldots, rP_{3,h},\ r\overrightarrow{Q}_{k+1}, \ldots, r\overrightarrow{Q}_h \right)$$

where $V_j = P_{3,j} + V(j, \mathsf{v}_j)$. The private key at level k consists of $(2 + h - k + \sum_{i=k+1}^{h} n_i)$ elements of G_1. Among these, only the first two are required in decryption, the rest are used to generate the private key for the next level as follows:
Let the secret key corresponding to the identity $\mathsf{v}_{|k-1} = (\mathsf{v}_1, \ldots, \mathsf{v}_{k-1})$ be

$$d_{\mathsf{v}_{|k-1}} = (A_0, A_1, B_k, \ldots, B_h, \overrightarrow{C}_k, \ldots, \overrightarrow{C}_h)$$

where $A_0 = \alpha P_2 + r' \sum_{j=1}^{k-1} V_j$, $A_1 = r'P$, and for $k \leq j \leq h$, $B_j = r' P_{3,j}$, $\overrightarrow{C}_j = r' Q_{j,1}, \ldots, r' Q_{j,n_j} = \langle C_{j,n_j} \rangle$ Pick a random $r^* \in \mathbb{Z}_p$ and compute

$$\begin{aligned} d_{\mathsf{v}} = (&A_0 + B_k + \textstyle\sum_{i=1}^{n_k} \mathsf{v}_k^i C_{k,i} + r^* \sum_{j=1}^{k} V_j,\ A_1 + r^* P, \\ &B_{k+1} + r^* P_{3,k+1}, \ldots, B_h + r^* P_{3,h}, \\ &\overrightarrow{C}_{k+1} + r^* \overrightarrow{Q}_{k+1}, \ldots, \overrightarrow{C}_h + r^* \overrightarrow{Q}_h). \end{aligned}$$

If we put $r = r' + r^*$, then d_{v} is a proper private key for $\mathsf{v} = (\mathsf{v}_1, \ldots, \mathsf{v}_k)$.

Encrypt: To encrypt $M \in G_2$ under the identity $(\mathsf{v}_1, \ldots, \mathsf{v}_k) \in (\mathbb{Z}_p)^k$, pick a random $s \in \mathbb{Z}_p$ and output

$$\mathsf{CT} = \left(e(P_1, P_2)^s \times M, sP, s\left(\sum_{j=1}^{k} V_j\right)\right)$$

where V_j is as defined in Key Generation.

Decrypt: To decrypt $\mathsf{CT} = (A, B, C)$ using the private key $d_{\mathsf{v}} = (d_0, d_1, \ldots)$ of $\mathsf{v} = (\mathsf{v}_1, \ldots, \mathsf{v}_k)$, compute

$$A \times \frac{e(d_1, C)}{e(B, d_0)} = e(P_1, P_2)^s \times M \times \frac{e\left(rP, s\sum_{j=1}^{k} V_j\right)}{e\left(sP, \alpha P_2 + r\sum_{j=1}^{k} V_j\right)} = M.$$

8.2 Security

Semantic security (i.e., CPA-security) of the above scheme in model $\mathcal{M}_2^+$ is proved under the h-wDBDHI* assumption. Note that, the additional flexibility in terms of choosing the target identity that we allowed to the adversary in the s$^+$-ID model is also applicable here.

THEOREM 8.1. *Let $n_1, \ldots, n_h, q$ and $n'_1, \ldots, n'_h$ be two sets of positive integers with $n'_i \leq n_i$ for $1 \leq i \leq h$. Then for $t \geq 1, q \geq 1$*

$$\mathsf{Adv}^{(h,n_1,\ldots,n_h)\text{-}\mathcal{G}_2}_{(h,n'_1,\ldots,n'_h)\text{-}\mathcal{M}_2^+}(t, q) \leq \mathsf{Adv}^{\mathsf{h\text{-}wDBDHI^*}}(t + O(\tau n q))$$

where $n = \sum_{i=1}^{h} n_i$.

Proof : Suppose $\mathcal{A}$ is a (t, q)-CPA adversary for $\mathcal{G}_2$, then we construct an algorithm $\mathcal{B}$ that solves the h-wDBDHI* problem. $\mathcal{B}$ takes as input a tuple $\langle P, Q, Y_1, \ldots, Y_h, T\rangle$ where $Y_i = \alpha^i P$ for some random $\alpha \in \mathbb{Z}_p^*$ and T is either equal to $e(P, Q)^{\alpha^{h+1}}$ or a random element of G_2. We define the modified $\mathcal{M}_2^+$ game between $\mathcal{B}$ and $\mathcal{A}$ as follows.

Initialization: $\mathcal{A}$ outputs sets of target identities for each level of the HIBE as $(\mathcal{I}_1^*, \ldots, \mathcal{I}_u^*)$ where each $\mathcal{I}_i^*$ is a set of cardinality n'_i for any $u \leq h$.

Setup: $\mathcal{B}$ defines polynomials $F_1(x), \ldots, F_h(x)$ where for $1 \leq i \leq u$,

$$\begin{aligned} F_i(x) &= \prod_{\mathsf{v}\in\mathcal{I}_i^*} (x - \mathsf{v}) \\ &= x^{n'_i} + a_{i,n'_i-1}x^{n'_i-1} + \ldots + a_{i,1}x + a_{i,0} \end{aligned}$$

For $u < i \leq h$, define $F_i(x) = a_{i,0}$ where $a_{i,0}$ is a random element of $\mathbb{Z}_p^*$. For $1 \leq i \leq u$, let $a_{i,n'_i} = 1$ and $a_{i,n_i} = \cdots = a_{i,n'_i+1} = 0$. For $u < i \leq h$ we set $n'_i = 0$ and $a_{i,n_i} = \cdots = a_{i,1} = 0$. For $1 \leq i \leq h$ define

$$J_i(x) = b_{i,n_i} x^{n_i} + b_{i,n_i-1} x^{n_i-1} + \ldots + b_{i,1} x + b_{i,0}$$

where $b_{i,j}$ are random elements of $\mathbb{Z}_p$. It then sets

$$P_1 = Y_1 = \alpha P; \qquad P_2 = Y_h + \beta P = (\alpha^h + \beta)P;$$

and for $1 \leq i \leq h$, $1 \leq j \leq n_i$,

$$\begin{aligned} Q_{i,j} &= b_{i,j} P + a_{i,j} Y_{h-i+1}; \\ P_{3,j} &= b_{i,0} P + a_{i,0} Y_{h-i+1}. \end{aligned}$$

$\mathcal{B}$ declares the public parameters to be

$$(P, P_1, P_2, \overrightarrow{P}_3, \overrightarrow{Q}_1, \ldots, \overrightarrow{Q}_h),$$

where $\overrightarrow{P}_3 = (P_{3,1}, \ldots, P_{3,h})$ and $\overrightarrow{Q}_i = (Q_{i,1}, \ldots, Q_{i,n_i})$. The corresponding master key $\alpha P_2 = Y_{h+1} + \beta Y_1$ is unknown to $\mathcal{B}$. The distribution of the public parameter is as expected by $\mathcal{A}$. **Phase 1:** Suppose $\mathcal{A}$ asks for the private key corresponding to an identity $\mathsf{v} = (\mathsf{v}_1, \ldots, \mathsf{v}_{h'})$ for $h' \leq h$. Note that for any $i \leq \eta'$,

$$\begin{aligned} V_i &= P_{3,i} + \sum_{j=1}^{n_i} \mathsf{v}_i^j Q_{i,j} \\ &= b_{i,0} P + a_{i,0} Y_{h-i+1} + \sum_{j=1}^{n_i} \mathsf{v}_i^j (b_{i,j} P + a_{i,j} Y_{h-i+1}) \\ &= F_i(\mathsf{v}_i) Y_{h-i+1} + J_i(\mathsf{v}_i) P. \end{aligned}$$

Hence, V_i is computable from what is known to $\mathcal{B}$.

Recall that $\mathcal{A}$ initially committed to sets of identities up to level u before the set-up phase. If $h' \leq u$, then there must be a $k \leq h'$ such that $F_k(\mathsf{v}_k) \neq 0$, as otherwise $\mathsf{v}_j \in \mathcal{I}_j^*$ for each $j \in \{1, \ldots, h'\}$ – which the adversary is not allowed by the rules of the Game. In case $h' > u$, it is possible that $F_1(\mathsf{v}_1) = \cdots = F_u(\mathsf{v}_u) = 0$. Then by construction $F_{u+1} \neq 0$. So, in either case there is a k such that $F_k(\mathsf{v}_k) \neq 0$. Moreover, k is the first such index in the range $\{1, \ldots, h'\}$. $\mathcal{B}$ picks a random $r \in \mathbb{Z}_p$ and assigns $d_{0|k} = (-J_k(\mathsf{v}_k)/F_k(\mathsf{v}_k))Y_k + \beta Y_1 + rV_k$ and $d_1 = (-1/F_k(\mathsf{v}_k))Y_k + rP$. Now,

$$\begin{aligned} d_{0|k} &= -\frac{J_k(\mathsf{v}_k)}{F_k(\mathsf{v}_k)} Y_k + \beta Y_1 + \alpha^k Y_{h-k+1} - \alpha^k \frac{F_k(\mathsf{v}_k)}{F_k(\mathsf{v}_k)} Y_{h-k+1} + rV_k \\ &= -\frac{J_k(\mathsf{v}_k)}{F_k(\mathsf{v}_k)} \alpha^k P + \alpha P_2 - \alpha^k \frac{F_k(\mathsf{v}_k)}{F_k(\mathsf{v}_k)} Y_{h-k+1} + rV_k \\ &= \alpha P_2 + \tilde{r} V_k \end{aligned}$$

where $\tilde{r} = (r - \frac{\alpha^k}{F_k(\mathsf{v}_k)})$. Also $d_1 = -\frac{1}{F_k(\mathsf{v}_k)} Y_k + rP = -\frac{\alpha^k}{F_k(\mathsf{v}_k)} P + rP = \tilde{r}P$. For any $j \in \{1, \ldots, h'\} \setminus \{k\}$ we have

$$\begin{aligned}\tilde{r}V_j &= (r - \frac{\alpha^k}{F_k(\mathsf{v}_k)})(F_j(\mathsf{v}_j)Y_{h-j+1} + J_j(\mathsf{v}_j)P) \\ &= r(F_j(\mathsf{v}_j)Y_{h-j+1} + J_j(\mathsf{v}_j)P) - \frac{1}{F_k(\mathsf{v}_k)}(F_j(\mathsf{v}_j)Y_{h+k-j+1} + J_j(\mathsf{v}_j)Y_k).\end{aligned}$$

Recall that, k is the smallest in the range $\{1, \ldots, h'\}$, such that, $F_k(\mathsf{v}_k) \neq 0$. Hence, for $j < k$, $F_j(\mathsf{v}_j) = 0$ and $\tilde{r}V_j = rJ_j(\mathsf{v}_j)P - \frac{J_j(\mathsf{v}_j)Y_k}{F_k(\mathsf{v}_k)}$. For $j > k$, $Y_{h+k-j+1}$ varies between Y_1 to Y_h. So $\mathcal{B}$ can compute all these $\tilde{r}V_j$s from the information it has. It forms

$$d_0 = d_{0|k} + \sum_{j \in \{1, \ldots, h'\} \setminus \{k\}} \tilde{r}V_j = \alpha P_2 + \tilde{r} \sum_{j=1}^{h'} V_j.$$

To form a valid private key, $\mathcal{B}$ also needs to compute $\tilde{r}P_{3,i}$ and $\tilde{r}\overrightarrow{Q}_i$ for $h' < i \leq h$. Now,

$$\begin{aligned}\tilde{r}P_{3,i} &= \left(r - \frac{\alpha^k}{F_k(\mathsf{v}_k)}\right)(b_{i,0}P + a_{i,0}Y_{h-i+1}) \\ &= r(b_{i,0}P + a_{i,0}Y_{h-i+1}) - \frac{1}{F_k(\mathsf{v}_k)}(b_{i,0}Y_k + a_{j,0}Y_{h+k-i+1}); \\ \tilde{r}Q_{i,j} &= \left(r - \frac{\alpha^k}{F_k(\mathsf{v}_k)}\right)(b_{i,j}P + a_{i,j}Y_{h-i+1}) \\ &= r(b_{i,j}P + a_{i,j}Y_{h-i+1}) - \frac{1}{F_k(\mathsf{v}_k)}(b_{i,j}Y_k + a_{i,j}Y_{h+k-i+1}).\end{aligned}$$

All these values are computable from what is known to $\mathcal{B}$. Hence, $\mathcal{B}$ forms the private key as:

$$d_{\mathsf{v}} = \left(d_0, d_1, \tilde{r}P_{3,\tau+1}, \ldots, \tilde{r}P_{3,h}, \tilde{r}\overrightarrow{Q}_{\tau+1}, \ldots, \tilde{r}\overrightarrow{Q}_h\right)$$

and provides it to $\mathcal{A}$.

Challenge: After completion of Phase 1, $\mathcal{A}$ outputs two messages $M_0, M_1 \in G_2$ together with a target identity $\mathsf{v}^* = (\mathsf{v}_1^*, \ldots, \mathsf{v}_{u'}^*)$, $u' \leq u$, on which it wishes to be challenged. The constraint is each $\mathsf{v}_j^* \in \mathcal{I}_j^*$ and hence $F_j(\mathsf{v}_j^*) = 0$ for $1 \leq j \leq u' \leq u$. $\mathcal{B}$ picks a random $b \in \{0, 1\}$ and provides $\mathcal{A}$ the challenge ciphertext

$$\mathsf{CT} = \left(M_b \times T \times e(Y_1, \beta Q),\ Q,\ \left(\sum_{i=1}^{u'} J_i(\mathsf{v}_i^*)\right) \times Q\right).$$

Suppose, $Q = \gamma P$ for some unknown $\gamma \in \mathbb{Z}_p$. Then

$$\sum_{j=1}^{u'} J_j(\mathsf{v}_j^*)Q = \gamma \sum_{j=1}^{u'} \left(J_j(\mathsf{v}_j^*)P + F_j(\mathsf{v}_j^*)Y_{h-j+1}\right) = \gamma \left(\sum_{j=1}^{u'} V_j\right).$$

If the input provided to $\mathcal{B}$ is a true h-wDBDHI* tuple, i.e., $T = e(P,Q)^{(\alpha^{h+1})}$, then

$$T \times e(Y_1, \beta Q) = e(P,Q)^{(\alpha^{h+1})} \times e(Y_1, \beta Q) = e(Y_h + \beta P, Q)^{\alpha} = e(P_1, P_2)^{\gamma}.$$

So, the challenge ciphertext is

$$\mathsf{CT} = \left(M_b \times e(P_1,P_2)^{\gamma}, \gamma P, \gamma \left(\sum_{j=1}^{u'} V_j\right)\right).$$

CT is a valid encryption of M_b under $\mathsf{v}^* = (\mathsf{v}_1^*, \ldots, \mathsf{v}_{u'}^*)$. On the other hand, when T is random, CT is random from the view point of $\mathcal{A}$.

Phase 2: This is similar to Phase 1. Note that $\mathcal{A}$ places at most q queries in Phase 1 and 2 together.

Guess: Finally, $\mathcal{A}$ outputs its guess $b' \in \{0,1\}$. $\mathcal{B}$ outputs $1 \oplus b \oplus b'$.

$\mathcal{A}$'s view in the above simulation is identical to that in a real attack. This gives us the required bound on the advantage of the adversary in breaking the HIBE protocol. ■

9 Product Scheme

Boneh-Boyen-Goh [Boneh et al., 2005] had proposed a "product" construction based on the BBG-HIBE and the BB-HIBE. A similar construction is possible based on the HIBE $\mathcal{G}_1$ of Section 5 and BB-HIBE. The resulting HIBE is secure in s$^+$-ID model. On the other hand,
in [Chatterjee and Sarkar, 2006a] we have presented a construction $\mathcal{H}_1$ which is secure in model $\boldsymbol{\mathcal{M}}_1$. This construction is in a sense an extension of the BB-HIBE. We propose a composite scheme based on $\mathcal{H}_1$ and $\mathcal{G}_2$ which we denote as (h,n)-$\mathcal{G}_3$ or simply $\mathcal{G}_3$, where h is the maximum number of levels in $\mathcal{G}_3$ and n is a parameter that comes from the underlying security model $\boldsymbol{\mathcal{M}}_1$.

The essential idea, as in [Boneh et al., 2005] is to form a product of two HIBEs. For this we represent an identity tuple in the form of a matrix (say $\mathbb{I}$) having (a-priori) fixed number of columns, ℓ_2 (say). When we look at a row of $\mathbb{I}$, it forms a constant-size ciphertext HIBE, $\mathcal{H}$, while each row taken together

as a single identity forms another HIBE, $\mathcal{H}'$. We obtain a product construction by instantiating $\mathcal{H}'$ to be $\mathcal{H}_1$ of [Chatterjee and Sarkar, 2006a] and $\mathcal{H}$ to be the constant-size ciphertext HIBE $\mathcal{G}_2$ of Section 8. In this case, the components of the identity tuples are from $\mathbb{Z}_p$ and we obtain security in $\boldsymbol{\mathcal{M}}_1$. Since $\boldsymbol{\mathcal{M}}_1$ allows the target identity to be of any length up to the maximum height of the HIBE, the adversary has the flexibility to choose the length of the target identity in the challenge phase.

9.1 Construction

Let the maximum depth of the HIBE be $h \leq \ell_1 \times \ell_2$. Here individual identity components are elements of $\mathbb{Z}_p$. **Setup:** Let P be a generator of G_1. Choose a random secret $x \in \mathbb{Z}_p$ and set $P_1 = xP$. Randomly choose P_2; an $\ell_1 \times \ell_2$ matrix $\mathcal{R}$ where

$$\mathcal{R} = \begin{bmatrix} R_{1,1} & \cdots & R_{1,\ell_2} \\ \vdots & \vdots & \vdots \\ R_{\ell_1,1} & \cdots & R_{\ell_1,\ell_2} \end{bmatrix}$$

and ℓ_2 many vectors $\overrightarrow{U_1}, \ldots, \overrightarrow{U_{\ell_2}}$ from G_1, where each $\overrightarrow{U_i} = (U_{i,1}, \ldots, U_{i,n})$, n being a parameter. The public parameters are $\langle P, P_1, P_2, \mathcal{R}, \overrightarrow{U_1}, \ldots, \overrightarrow{U_{\ell_2}} \rangle$, while the master secret is xP_2.

Key Generation: Given an identity $\mathsf{v} = (\mathsf{v}_1, \ldots, \mathsf{v}_u)$, for any $u \leq h$, this algorithm generates the private key d_{v} of v as follows.

Let $u = k_1\ell_2 + k_2$ with $k_2 \in \{1, \ldots, \ell_2\}$. We represent v by a (possibly incomplete) $(k_1 + 1) \times \ell_2$ matrix $\mathcal{I}$ where the last row has k_2 elements. Choose $(k_1 + 1)$ many random elements $r_1, \ldots, r_{k_1}, r_{k_2} \in \mathbb{Z}_p$ and output

$$\begin{aligned} d_{\mathsf{v}} = \Big(&xP_2 + \sum_{i=1}^{k_1} r_i \sum_{j=1}^{\ell_2} (V_{i,j} + R_{i,j}) + r_{k_2} \sum_{j=1}^{k_2} (V_{k_1+1,j} + R_{k_1+1,j}), r_1P, \ldots, r_{k_1}P, \\ &r_{k_2}P,\, r_{k_2}R_{k_1+1,k_2+1}, \ldots, r_{k_2}R_{k_1+1,\ell_2}, r_{k_2}\overrightarrow{U_{k_2+1}}, \ldots, r_{k_2}\overrightarrow{U_{\ell_2}}\Big) \\ = (&a_0, a_1, \ldots, a_{k_1}, a_{k_1+1}, b_{k_2+1}, \ldots, b_{\ell_2}, \overrightarrow{c}_{k_2+1}, \ldots, \overrightarrow{c}_{\ell_2}) \quad \text{say.} \end{aligned}$$

where $V_{i,j} = \sum_{k=1}^{n} \mathsf{v}_{i,j}^k U_{j,k}$ and $r_{k_2}\overrightarrow{U_i}$ denotes that each element of $\overrightarrow{U_i}$ is multiplied by the scalar r_{k_2}.

Note: Here $u = k_1\ell_2 + k_2$, so the first $k_1\ell_2$ components of the identity tuple can be arranged as the first k_1 rows of a matrix having ℓ_2 many columns. Each of these rows taken separately can be viewed as an identity tuple for a constant-size ciphertext HIBE, $\mathcal{H}$, having maximum depth ℓ_2. Similarly, the last $k_2 \leq \ell_2$ components of the identity tuple can be viewed as a separate identity tuple of the same constant-size ciphertext HIBE. Next, we view each of the first k_1 rows

as a single identity component of another HIBE, $\mathcal{H}'$. We now take a closer look at the structure of d_{v}. Let,

$$\begin{aligned} a_0 &= xP_2 + \sum_{i=1}^{k_1} r_i \sum_{j=1}^{\ell_2} (V_{i,j} + R_{i,j}) + r_{k_2} \sum_{j=1}^{k_2} (V_{k_1+1,j} + R_{k_1+1,j}) \\ &= A_1 + A_2 + A_3 \end{aligned}$$

Here, $A_1 = xP_2$ is the master key and $A_2 + A_3$ is used to generate the private key for v by suitably masking the master secret. $A_2 = \sum_{i=1}^{k_1} r_i \sum_{j=1}^{\ell_2} (V_{i,j} + R_{i,j})$ – the inner sum is over a single row which forms a full-length identity tuple for the constant-size ciphertext HIBE $\mathcal{H}$; while the outer sum is over the first k_1 rows where we treat each row as a single identity component for $\mathcal{H}'$. $A_3 = r_{k_2} \sum_{j=1}^{k_2} (V_{k_1+1,j} + R_{k_1+1,j})$ is for the remaining row having $k_2 \leq \ell_2$ many elements and this row forms an identity tuple of depth k_2 for $\mathcal{H}$. Altogether we have $k_1 + 1$ levels in $\mathcal{H}'$ and $a_1, \ldots, a_{k_1+1}$ correspond to each of these levels. These elements i.e, $(a_0, a_1, \ldots, a_{k+1})$ are sufficient for decryption as we will see in the Decryption algorithm. The rest of the elements, i.e., b_is and $\overrightarrow{c}_i$s are required for generating the private key for the next level as we show below.

The private key of v can also be generated given the private key of $\mathsf{v}_{|_{u-1}} = \mathsf{v}_1, \ldots, \mathsf{v}_{u-1}$ as required. There are two cases to be considered.

Case 1: Suppose $u - 1 = k_1\ell_2 + \ell_2 = (k_1 + 1)\ell_2$, then

$$\begin{aligned} d_{\mathsf{v}_{|_{u-1}}} &= \left(xP_2 + \sum_{i=1}^{k_1+1} r_i \sum_{j=1}^{\ell_2} (V_{i,j} + R_{i,j}), r_1P, \ldots, r_{k_1}P, r_{k_1+1}P \right) \\ &= (a_0, a_1, \ldots, a_{k_1}, a_{k_1+1}) \quad (say) \end{aligned}$$

Choose a random $r^* \in \mathbb{Z}_p$ and form d_{v} as

$$\begin{aligned} d_{\mathsf{v}} = (&a_0 + r^*(V_{k_1+2,1} + R_{k_1+2,1}), a_1, \ldots, a_{k_1+1}, r^*P, \\ &r^*R_{k_1+2,2}, \ldots, r^*R_{k_1+2,\ell_2}, r^*\overrightarrow{U_2}, \ldots, r^*\overrightarrow{U_{\ell_2}}). \end{aligned}$$

Case 2: Let, $u - 1 = k_1\ell_2 + k_2'$ with $k_2' < \ell_2$ then,

$$\begin{aligned} d_{\mathsf{v}_{|_{u-1}}} &= xP_2 + \sum_{i=1}^{k_1} r_i \sum_{j=1}^{\ell_2} (V_{i,j} + R_{i,j}) + r'_{k_2} \sum_{j=1}^{k_2'} (V_{k_1+1,j} + R_{k_1+1,j}), r_1P, \ldots, \\ &\qquad r_{k_1}P, r'_{k_2}P, r'_{k_2}R_{k_1+1,k_2'+1}, \ldots, r'_{k_2}R_{k_1+1,\ell_2}, r'_{k_2}\overrightarrow{U}_{k_2'+1}, \ldots, r'_{k_2}\overrightarrow{U}_{\ell_2}\Big) \\ &= (a_0, a_1, \ldots, a_{k_1}, a_{k_1+1}, b_{k_2'+1}, \ldots, b_{\ell_2}, \overrightarrow{c}_{k_2'+1}, \ldots, \overrightarrow{c}_{\ell_2}) \quad (say) \end{aligned}$$

Choose a random $r^* \in \mathbb{Z}_p$ and form d_{v} as

$$d_{\mathsf{v}} = a_0 + \sum_{j=1}^{n} \mathsf{v}_u^j c_{k_2'+1,j} + b_{k_2'+1} + r^* \sum_{j=1}^{k_2'+1} (V_{k_1+1,j} + R_{k_1+1,j}), a_1, \ldots, a_{k_1},$$

$$a_{k_1+1} + r^*P, b_{k'_2+2} + r^*R_{k_1+1,k'_2+2}, \ldots, b_{\ell_2} + r^*R_{k_1+1,\ell_2},$$
$$\overrightarrow{c}_{k'_2+2} + r^*\overrightarrow{U}_{k'_2+2}, \ldots, \overrightarrow{c}_{\ell_2} + r^*\overrightarrow{U}_{\ell_2}$$

It can be verified that d_{v} is a proper private key for v.

Encrypt: To encrypt a message $M \in G_2$ under the public key $\mathsf{v} = (\mathsf{v}_1, \ldots, \mathsf{v}_u)$ choose a random $s \in \mathbb{Z}_p$ and then the ciphertext is

$$C = \Bigg(e(P_1, P_2)^s \times M, sP, s\sum_{j=1}^{\ell_2}(V_{1,j} + R_{1,j}), \ldots, \sum_{j=1}^{\ell_2}(V_{k_1,j} + R_{k_1,j}), s\sum_{j=1}^{k_2}(V_{k_1+1,j} + R_{k_1+1,j})\Bigg)$$
$$= (A, B, C_1, \ldots, C_{k_1}, C_{k_1+1}) \quad \text{(say)}$$

where $V_{i,j}$ is as defined in Key Generation part. Each C_i correponds to the ith row of the identity matrix for v.

Decrypt: Let $\mathsf{CT} = (A, B, C_1, \ldots, C_{k_1}, C_{k_1+1})$ be a cipher text and $\mathsf{v} = \mathsf{v}_1, \ldots, \mathsf{v}_u$ be the corresponding identity represented as a $(k_1 + 1) \times \ell_2$ matrix. Then we decrypt CT using $d_{\mathsf{ID}} = (d_0, d_1, \ldots, d_{k_1+1}, \ldots)$ as

$$A \times \frac{\prod_{i=1}^{k_1+1}(d_i, C_i)}{e(B, d_0)} = M.$$

9.2 Security

Security of the above hybrid construction in the generalised selective-ID model (h, n')-$\boldsymbol{\mathcal{M}}_1$ of [Chatterjee and Sarkar, 2006a] can be reduced from the hardness of ℓ_2-wDBDHI* problem. Here we give a sketch of the proof.

THEOREM 9.1. *Let h, n, q be positive integers and n' be another positive integer with $n' \leq n$. Then*

$$\mathsf{Adv}^{(h,n)\text{-}\mathcal{G}_3}_{(h,n')\text{-}\boldsymbol{\mathcal{M}}_1}(t, q) \leq \mathsf{Adv}^{\ell_2-\mathsf{wDBDHI}^*}(t + O(\tau nq)).$$

Proof : We want to prove (h, n)-$\mathcal{G}_3$ secure in model (h, n')-$\boldsymbol{\mathcal{M}}_1$ using a reductionist security argument where $1 \leq n' \leq n$. This means that the public parameters of the HIBE depend on n, while the adversary commits to a set $\mathcal{I}^*$ of size n' in the commit phase.

The simulator is provided with a tuple $\langle P, Q, Y_1, \ldots, Y_{\ell_2}, T\rangle \in G_1^{\ell_2+2} \times G_2$. It has to decide whether this is a proper ℓ_2-wDBDHI* instance or not.

Adversary's commitment: $\mathcal{A}$ commits to a set $\mathcal{I}^*$, where $|\mathcal{I}^*| = n'$. The restriction on the adversary is that in the private key extraction query at least one component of the identity tuple should be outside $\mathcal{I}^*$; while in the challenge phase it asks for the encryption under an identity v^* all of whose components are from $\mathcal{I}^*$.

Set-up: The simulator defines

$$F(x) = \prod_{\mathsf{v}\in\mathcal{I}^*}(x - \mathsf{v}) = x^{n'} + \cdots + a_1 x + a_0$$

$$J_i^{(j)}(x) = b_{i,n}x^n + \cdots + b_{i,1}x + b_{i,0}^{(j)} \text{ for } 1 \le i \le \ell_1, \ \ 1 \le j \le \ell_2$$

where each $b_{i,k}$ and $b_{i,0}^{(j)}$ is chosen at random from $\mathbb{Z}_p^*$. Define $a_{n'} = 1$ and $a_n = a_{n-1} = \cdots = a_{n'+1} = 0$. The simulator defines $P_1 = Y_1$, $P_2 = Y_{\ell_2} + \beta P$ in a similar manner as in the set-up of Section 6. It further defines $U_{i,j} = b_{i,j}P + a_i Y_{h-i+1}$ for $1 \le i \le \ell_2$, $1 \le j \le n$ and $R_{k,j} = b_{k,0}^{(j)}P + a_0 Y_{\ell_2-j+1}$ for $1 \le k \le \ell_1$, $1 \le j \le \ell_2$.

The simulator gives the public parameters $\langle P, P_1, P_2, \mathcal{R}, \overrightarrow{U_1}, \ldots, \overrightarrow{U_{\ell_2}}\rangle$ to $\mathcal{A}$, while the corresponding master secret is unknown to the simulator.

Phase 1: Suppose $\mathcal{A}$ asks for the private key of an identity $\mathsf{v} = \mathsf{v}_1, \ldots, \mathsf{v}_m$ where $m = k_1 \times \ell_2 + k_2$. The simulator first forms the $(k_1 + 1) \times \ell_2$ matrix $\mathcal{I}$ where v_1 is indexed as $\mathsf{v}_{1,1}$ and v_m as v_{k_1+1,k_2}. The last row of the matrix may have elements less than ℓ_2. As per the rule of the game there is at least one identity, say v_l, such that $F(\mathsf{v}_l) \neq 0$. Suppose, v_l is indexed as k_1', k_2' in $\mathcal{I}$. Now consider the identity tuple $(\mathsf{v}_{k_1',1}, \ldots, \mathsf{v}_{k_1',k_2'})$. This by itself can be seen as a valid identity tuple of depth k_2' for the HIBE $\mathcal{H}$. Using the technique of Section 6, the simulator forms a private key for $(\mathsf{v}_{k_1',1}, \ldots, \mathsf{v}_{k_1',k_2'})$ as $(a_0', a_{k_1'}, b_{k_2'+1}, \ldots, b_{\ell_2}, \overrightarrow{c}_{k_2'+1}, \ldots, \overrightarrow{c}_{\ell_2})$. Note that, this is a valid private key for an identity tuple of depth k_2' in the constant-size ciphertext HIBE $\mathcal{H}$. It next chooses $r_1, \ldots, r_{k_1'-1} \in \mathbb{Z}_p$ and computes the private key for $(\mathsf{v}_1, \ldots, \mathsf{v}_l)$ as

$$a_0 = a_0' + \sum_{i=1}^{k_1'-1} r_i \sum_{j=1}^{\ell_2}(V_{i,j} + R_{i,j})$$

$$a_i = r_i P \quad \mathit{for} \quad 1 \le i \le k_i' - 1$$

Note that, $V_{i,j} = \sum_{k=1}^{n} \mathsf{v}_{i,j}^k U_{j,k}$, so

$$\begin{aligned}
V_{i,j} + R_{i,j} &= \sum_{k=1}^{n} \mathsf{v}_{i,j}^k U_{j,k} + R_{i,j} \\
&= \sum_{k=1}^{n} \mathsf{v}_{i,j}^k (b_{j,k}P + a_j Y_{\ell_2-j+1}) + b_{i,0}^{(j)}P + a_0 Y_{\ell_2-j+1} \\
&= F(\mathsf{v}_{i,j})Y_{\ell_2-j+1} + J_i^{(j)}(\mathsf{v}_{i,j})P
\end{aligned}$$

The simulator can compute all these from the information it possesses. Hence,

$$(a_0, a_1, \ldots, a_{k_i'-1}, a_{k_1'}, b_{k_2'+1}, \ldots, b_{\ell_2}, c_{k_2'+1}, \ldots, c_{\ell_2})$$

is a proper private key for $\mathsf{v}_1, \ldots, \mathsf{v}_l$ from which the simulator forms a private key for v and gives it to $\mathcal{A}$.

Challenge: At this stage, $\mathcal{A}$ produces two equal length messages $M_0, M_1 \in G_2$ and a challenge identity v^*. The challenge identity $\mathsf{v}^* = (\mathsf{v}_1^*, \ldots, \mathsf{v}_u^*)$ should have each $\mathsf{v}_j \in \mathcal{I}^*$ and hence $F(\mathsf{v}_j^*) = 0$ for $1 \leq j \leq u$. Based on this fact the simulator is able to form a proper encryption of M_γ where γ is chosen uniformly at random from $\{0, 1\}$, if the tuple provided to it is a true h-wDBDHI* instance.

Phase 2: The key extraction queries in this stage are handled as in Phase 1.

Guess: The adversary outputs a guess γ'. The simulator outputs 1 if $\gamma = \gamma'$, else it outputs 0.

$\mathcal{A}$'s view in the above simulation is identical to that in a real attack if the given instance is a true ℓ_2-wDBDHI* instance.

The above shows that an adversary's ability to attack (h, n)-$\mathcal{G}_3$ HIBE in model (h, n')-$\boldsymbol{\mathcal{M}}_1$ can be converted into an algorithm for solving ℓ_2-wDBDHI* problem. The bound on the advantage follows from this fact. ■

Note that, in the commitment stage we may give the adversary some more flexibility by allowing it to commit to sets of identities $\mathcal{I}_1^*, \ldots, \mathcal{I}_h^*$, where $\mathcal{I}_j^*$ corresponds to the commitment for the jth level of the constant-size ciphertext HIBE. In this case the restrictions in $\boldsymbol{\mathcal{M}}_2$ regarding the private key queries and challenge generation apply. This added flexibility, however, does not affect the efficiency of the protocol.

10 Discussion

The private key corresponding to an identity in a HIBE has two roles. The first role is to enable decryption of a message encrypted using this identity, while the second role is to enable generation of lower level keys. Not all components of the private key are necessarily required for decryption, i.e., the decryption subkey can have strictly fewer components than the whole private key. This has also been observed in [Boneh et al., 2005] and in case of the BBG-HIBE, the decryption subkey consists of only two components. In $\mathcal{G}_1$ and $\mathcal{G}_2$, the decryption subkeys also consist of two components as in the BBG-HIBE. In $\mathcal{G}_3$ the size of the decryption subkey is reduced by a factor of h compared to the size of the decryption subkeys in $\mathcal{H}_1$.

Having a small decryption subkey is important, since the decryption subkey may need to be loaded on to smart cards for frequent and online decryptions. This is achieved in all the HIBE constructions described in this work. On the other hand, the entire private key is required for key delegation to lower level entities. Key delegation is a relatively infrequent activity which will typically be done by an entity from a workstation. Storage in a workstation is less restrictive and a larger size private key required only for key delegation is more tolerable.

The size of the private key in the BBG-HIBE and $\mathcal{G}_1$ is proportional to the number of levels in the HIBE. For $\mathcal{G}_2$ this size is proportional to $n \times h$, where h

is the number of levels of the HIBE and n is the maximum number of challenge identities that the adversary can commit to for any level. The size of the private key in $\mathcal{G}_3$ varies cyclically with the number of components j in the identity. Let $j = j_1h+j_2$, where h is the number of levels in $\mathcal{H}$ used in the product construction and $j_2 \in \{1,\ldots,h\}$. The size of the private key then varies as $j_1+n\times j_2$, where n is the number of elements in the set from which the adversary can construct the challenge identity. Since j_2 varies in a cyclic manner with period h, the size of the private key also shows a similar behaviour. (A similar behaviour is also shown by the size of the private key in the product construction in [Boneh et al., 2005].) A modification of the protocols eliminates the dependence of the size of the private key on j_2. Suppose that key delegation is only allowed to be performed by the PKG and entities at levels $h, 2h, 3h, \ldots$. For example, in a big organisation, the hierarchy may be divided into sub-hierarchies. The entities at levels $h, 2h$ etcetera are the system administrators for the sub-hierarchy of depth h and the delegation of private key is solely managed by them. The other entities in the sub-hierarchy are not involved with the business of key-delegation but they can still access the secret information encrypted for their subordinates. In this scenario, the size of the private key varies only with j_1 and in fact, the private key and the decryption subkey become identical.

11 Conclusion

In this work, we have augmented the selective-ID security model for hierarchical identity-based encryption by allowing the adversary some flexibility in choosing the target identity tuple during the challenge phase of the security reduction. We have denoted this model by selective$^+$-ID model (s$^+$-ID model). The Boneh-Boyen HIBE satisfies this notion of security while the constant-size ciphertext HIBE of Boneh, Boyen and Goh needs some modification in the security reduction to do so. This modification introduces a multiplicative security degradation. We have further augmented the BBG-HIBE to construct a new protocol secure in s$^+$-ID model without any degradation which maintains all the attractive features of BBG-HIBE. We build on this new construction another constant-size ciphertext HIBE. The security of our second construction is proved under a generalization of the selective-ID security model. Our third construction of HIBE is a "product" construction that allows a controllable trade-off between the ciphertext size and the private key size.

Acknowledgement

We thank the anonymous reviewers for their detailed and valuable comments.

References

[Barreto et al., 2002] Barreto, P. S. L. M., Kim, H. Y., Lynn, B., and Scott, M. (2002). Efficient Algorithms for Pairing-Based Cryptosystems. In Yung, M., editor, *CRYPTO*, volume 2442 of *Lecture Notes in Computer Science*, pages 354–368. Springer.

[Boneh and Boyen, 2004] Boneh, D. and Boyen, X. (2004). Efficient Selective-ID Secure Identity-Based Encryption Without Random Oracles. In Cachin, C. and Camenisch, J., editor, *EUROCRYPT*, volume 3027 of *Lecture Notes in Computer Science*, pages 223–238. Springer.

[Boneh et al., 2005] Boneh, D., Boyen, X., and Goh, E.-J. (2005). Hierarchical Identity Based Encryption with Constant Size Ciphertext. In Cramer, R., editor, *EUROCRYPT*, volume 3494 of *Lecture Notes in Computer Science*, pages 440–456. Springer. Full version available at Cryptology ePrint Archive; Report 2005/015.

[Boneh and Franklin, 2003] Boneh, D. and Franklin, M. K. (2003). Identity-Based Encryption from the Weil Pairing. *SIAM J. Comput.*, 32(3):586–615. Earlier version appeared in the proceedings of CRYPTO 2001.

[Boneh and Katz, 2005] Boneh, D. and Katz, J. (2005). Improved Efficiency for CCA-Secure Cryptosystems Built Using Identity-Based Encryption. In Menezes, A., editor, *CT-RSA*, volume 3376 of *Lecture Notes in Computer Science*, pages 87–103. Springer.

[Boyen et al., 2005] Boyen, X., Mei, Q., and Waters, B. (2005). Direct Chosen Ciphertext Security from Identity-Based Techniques. In Atluri, V., Meadows, C., and Juels, A., editors, *ACM Conference on Computer and Communications Security*, pages 320–329. ACM.

[Canetti et al., 2003] Canetti, R., Halevi, S., and Katz, J. (2003). A Forward-Secure Public-Key Encryption Scheme. In Biham, E., editor, *EUROCRYPT*, volume 2656 of *Lecture Notes in Computer Science*, pages 255–271. Springer.

[Canetti et al., 2004] Canetti, R., Halevi, S., and Katz, J. (2004). Chosen-Ciphertext Security from Identity-Based Encryption. In Cachin, C. and Camenisch, J., editor, *EUROCRYPT*, volume 3027 of *Lecture Notes in Computer Science*, pages 207–222. Springer.

[Chatterjee and Sarkar, 2006a] Chatterjee, S. and Sarkar, P. (2006a). Generalization of the Selective-ID Security Model for HIBE Protocols. In Yung, M., Dodis, Y., Kiayias, A., and Malkin, T., editors, *Public Key Cryptography*, volume 3958 of *Lecture Notes in Computer Science*, pages 241–256. Springer. Revised version available at Cryptology ePrint Archive, Report 2006/203.

[Chatterjee and Sarkar, 2006b] Chatterjee, S. and Sarkar, P. (2006b). New Constructions of Constant Size Ciphertext HIBE Without Random Oracle. In Rhee, M. and Lee, B., editors, *ICISC*, volume 4296 of *Lecture Notes in Computer Science*, pages 310–327. Springer.

[Galbraith et al., 2002] Galbraith, S. D., Harrison, K., and Soldera, D. (2002). Implementing the Tate Pairing. In Fieker, C. and Kohel, D. R., editors, *ANTS*, volume 2369 of *Lecture Notes in Computer Science*, pages 324–337. Springer.

[Gentry and Silverberg, 2002] Gentry, C. and Silverberg, A. (2002). Hierarchical ID-Based Cryptography. In Zheng, Y., editor, *ASIACRYPT*, volume 2501 of *Lecture Notes in Computer Science*, pages 548–566. Springer.

[Horwitz and Lynn, 2002] Horwitz, J. and Lynn, B. (2002). Toward Hierarchical Identity-Based Encryption. In Knudsen, L. R., editor, *EUROCRYPT*, volume 2332 of *Lecture Notes in Computer Science*, pages 466–481. Springer.

[Shamir, 1984] Shamir, A. (1984). Identity-Based Cryptosystems and Signature Schemes. In Blakley, G. R. and Chaum, D., editors, *CRYPTO*, volume 196 of *Lecture Notes in Computer Science*, pages 47–53. Springer.

A The Case of Boneh-Boyen HIBE

The original reduction in [Boneh and Boyen, 2004] goes through without almost any modification for the s$^+$-ID model. The only change is in challenge generation as described below.

Initialization: A commits to a target identity $\mathsf{v}^* = (\mathsf{v}_1^*, \ldots, \mathsf{v}_k^*)$ of height $k \leq h$. If $k < h$, B adds extra random elements from $\mathbb{Z}_p$ to make v^* an identity of height h.

Setup: B picks random $\alpha_1, \ldots, \alpha_h \in \mathbb{Z}_p$ and defines $Q_j = \alpha_j P - \mathsf{v}_j^* P_1$ for $1 \leq j \leq h$. It gives A the public parameters $\mathsf{PP} = \langle P, P_1, P_2, Q_1, \ldots, Q_h \rangle$. Here the $\mathsf{msk} = aP_2 = abP$ is unknown to B. Define the function $F_j(x) = xP_1 + Q_j = (x - \mathsf{v}_j^*)P_1 + \alpha_j P$ for $1 \leq j \leq h$.

Phase 1 and Phase 2: As in [Boneh and Boyen, 2004].

Challenge: After completion of Phase 1, A outputs two messages $M_0, M_1 \in G_2$ and an identity tuple $\mathsf{v}^+ = (\mathsf{v}_1^*, \ldots, \mathsf{v}_\tau^*)$, $\tau \leq k$. B chooses a random bit γ and forms the ciphertext $C = (M_\gamma \cdot Z, cP, \alpha_1 cP, \ldots, \alpha_\tau cP)$. Note that, $F_i(\mathsf{v}_i^*) = \alpha_i P$, so

$$C = \langle M_\gamma \cdot Z, cP, cF_1(\mathsf{v}_1^*), \ldots, cF_\tau(\mathsf{v}_\tau^*) \rangle.$$

If $Z = e(P, P)^{abc} = e(P_1, P_2)^c$ then C is a valid encryption of M_γ.
