# Supplementary material for: In-text citation’s frequencies-based recommendations of relevant research papers
Source: PeerJ Comput Sci. 2021 Jun 4;7:e524. doi: 10.7717/peerj-cs.524 (PMC8189020; doi:10.7717/peerj-cs.524)
Supplement: Supplemental Information 2 — These articles are available under a CC BY-ND 4.0 license. [file peerj-cs-07-524-s002.zip › Dataset/Papers Dataset.zip!/Papers Dataset/jucs_114.pdf]

Journal of Universal Computer Science, vol. 11, no. 12 (2005), 2114-2124
submitted: 2/11/05, accepted: 25/11/05, appeared: 28/12/05 © J.UCS

Hausdorff Measure and Łukasiewicz Languages[1]

Ludwig Staiger
(Martin-Luther Universtät Halle-Wittenberg, Germany
staiger@informatik.uni-halle.de)

Abstract: The paper investigates fixed points and attractors of infinite iterated function systems in Cantor space. By means of the theory of formal languages simple examples of the non-coincidence of fixed point and attractor (closure of the fixed point) are given.

Key Words: Iterated function system, attractor, fractals, Hausdorff measure, Cantor space, ω-languages, fractals, simple deterministic languages
Category: F.4.1,F.1.1

1 Introduction

Iterated function systems (IFS) are well-known in fractal geometry as a means to describe sets of fractal nature [Edgar 1990, Falconer 1990]. Usually, an IFS consists of a finite set of contracting mappings of a metric space $\mathcal{M}$ into itself and defines in a unique way a (largest) fixed point which is also called its attractor. This fixed point (or attractor) is a nonempty closed subset of $\mathcal{M}$.

If one considers infinite iterated function systems (IIFS) (cf. [Fernau 1994a, Fernau 1994b, Mauldin 1995, Mauldin and Urbański 1996]) unlike the case of finite IFS the fixed point need not be closed. Thus, for IIFS, fixed point and attractor, which we define as the closure of the fixed point[2], in general, do not coincide.

In a recent paper [Staiger 2005a] we provided a series of simple examples for several levels of the non-coincidence of fixed point and attractors for infinite iterated function systems using means of formal language theory. As a criterion for the distinction we used a combination of Hausdorff dimension and Hausdorff measure. The underlying space is the Cantor space (X^ω, ρ), the contracting mappings ϕ_w were defined by pre-multiplication with finite strings $w \in X^*$, $\phi_w(\xi) := w\xi$ for $\xi \in X^\omega$, and, therefore, the IIFS $(\phi_w)_{w\in W}$ considered are most simply described by formal languages $W \subseteq X^*$.

For languages simplicity can be expressed in terms of structure and complexity. The structure we required in [Staiger 2005a] was prefix-freeness, that is, for $w, v \in W, w \neq v$ the images $\phi_w(X^\omega)$ and $\phi_v(X^\omega)$ are disjoint. This results

[1] C. S. Calude, H. Ishihara (eds.). *Constructivity, Computability, and Logic. A Collection of Papers in Honour of the 60th Birthday of Douglas Bridges.*
[2] Since there is no standard terminology, we use these terms to denote the two sets related to an IIFS.

also in a simple topological structure of the fixed point. From the complexity point of view, with three exceptions, the languages (examples) constructed in [Staiger 2005a] were context-free languages having low complex acceptors (cf. [Autebert et al. 1997]): they are accepted by one- or two-turn deterministic one-counter automata.

In this paper, we continue this line of investigation to construct simple IIFS in Cantor space which exhibit a certain level of distinction between fixed point and attractor and which are described by prefix-free deterministic context-free languages.

As an additional instance we investigate the possibility to obtain fixed points and attractors which exhibit, besides the self-similarity induced by the generating IIFS, a certain kind of finite self-similarity as described in the graph-directed constructions of [Bandt 1989, Mauldin and Williams 1988] (see also [Edgar 1990]). In our special case of Cantor space such sets are also known as finite-state subsets of X^ω (see [Staiger 1983, Trakhtenbrot 1962]).

In our examples we shall use Łukasiewicz languages (see [Staiger 2005b]). Their construction exhibits interesting information-theoretic properties (see [Kuich 1970, Staiger 2005b]), which in view of the close relation between the entropy of languages and Hausdorff dimension (see [Staiger 1993]) could result in IIFS whose fixed points have the desired properties and whose underlying languages can be constructed as deterministic context-free languages of low complex structure.

2 Notation and Preliminary Results

Next we introduce the notation used throughout the paper. By $\mathbb{N} = \{0, 1, 2, \ldots\}$ we denote the set of natural numbers. Let X be an alphabet of cardinality $|X| = r$. By X^* we denote the set (monoid) of words on X, including the *empty word* e, and X^ω is the set of infinite sequences (ω-words) over X. For $w \in X^*$ and $\eta \in X^* \cup X^\omega$ let $w \cdot \eta$ be their *concatenation*. This concatenation product extends in an obvious way to subsets $W \subseteq X^*$ and $B \subseteq X^* \cup X^\omega$. For a language W let $W^* := \bigcup_{i\in\mathbb{N}} W^i$ be the *submonoid* of X^* generated by W, and by $W^\omega := \{w_1 \cdots w_i \cdots : w_i \in W \setminus \{e\}\}$ we denote the set of infinite strings formed by concatenating words in W. Furthermore $|w|$ is the *length* of the word $w \in X^*$ and $\mathbf{A}(B)$ is the set of all finite prefixes of strings in $B \subseteq X^* \cup X^\omega$. We shall abbreviate $w \in \mathbf{A}(\eta)$ $(\eta \in X^* \cup X^\omega)$ by $w \sqsubseteq \eta$.

A language $V \subseteq X^*$ is called a *prefix-free* provided for arbitrary $w, v \in V$ the relation $w \sqsubseteq v$ implies $w = v$.

Further we denote by $B/w := \{\eta : w \cdot \eta \in B\}$ the *left derivative* or *state* of the set $B \subseteq X^* \cup X^\omega$ generated by the word w. We refer to B as *finite-state* provided the set of states $\{B/w : w \in X^*\}$ is finite. As usual a finite-state language $W \subseteq X^*$ is called *regular*.

In the case of ω-languages *regular ω-languages*, that is, ω-languages accepted by finite automata, are the finite unions of sets of the form $W \cdot V^\omega$, where W and V are regular languages (cf. e.g. [Staiger 1997a]). In particular, every regular ω-language is finite-state, but, as it was observed in [Trakhtenbrot 1962], not every finite-state ω-language is regular (cf. also [Staiger 1983]).

In the sequel we assume the reader to be familiar with basic facts of language theory (e.g. [Berstel and Perrin 1985, Hopcroft and Ullman 1979] or Vol. 1 of [Rozenberg and Salomaa 1997])

For a language $W \subseteq X^*$ let $\mathsf{s}_W : \mathbb{N} \to \mathbb{N}$ where $\mathsf{s}_W(n) := |W \cap X^n|$ be its *structure function.* The *structure generating function* corresponding to s_W is

$$\mathfrak{s}_W(t) := \sum\nolimits_{i \in \mathbb{N}} \mathsf{s}_W(i) \cdot t^i. \tag{1}$$

$\mathfrak{s}_W$ is a power series with convergence radius $\mathsf{rad}\, W := \liminf\limits_{n\to\infty} \frac{1}{\sqrt[n]{\mathsf{s}_W(n)}}$. It is convenient to consider $\mathfrak{s}_W$ also as a function mapping $[0,\infty)$ to $[0,\infty) \cup \{\infty\}$. If $W \not\subseteq \{e\}$ then $\mathfrak{s}_W$ is a continuous and strictly increasing mapping on $[0, \mathsf{rad}\, W)$.

The convergence radius $\mathsf{rad}\, W$ is closely related to the entropy of the language (cf. [Kuich 1970, Staiger 1993, Staiger 2005b]),

$$\mathsf{H}_W = \limsup\nolimits_{n\to\infty} \frac{\log_r(1+\mathsf{s}_W(n))}{n} \ .$$

The parameter $\mathbf{t}_1(W) := \sup\{t : t \geq 0 \wedge \mathfrak{s}_W(t) \leq 1\} \leq \mathsf{rad}\, W$ is important for the calculation of $\mathsf{rad}\, W^*$. It fulfills the following (see [Kuich 1970, Staiger 1993]).

Lemma 1. *It holds* $\mathfrak{s}_W(\mathbf{t}_1(W)) = 1$ *or* $\mathfrak{s}_W(\mathsf{rad}\, W) < 1$.

If $\mathfrak{s}_W(\mathsf{rad}\, W) \leq 1$*, then* $\mathbf{t}_1(W) = \mathsf{rad}\, W = \mathsf{rad}\, W^*$*. If* $\mathfrak{s}_W(\mathsf{rad}\, W) > 1$ *then* $\mathsf{rad}\, W^* \leq \mathbf{t}_1(W)$.

If W is prefix-free then we have always $\mathsf{rad}\, W^* = \mathbf{t}_1(W)$ *and, moreover,* $\mathfrak{s}_W(\mathsf{rad}\, W^*) = 1$ *or* $\mathsf{rad}\, W^* = \mathsf{rad}\, W$.

We consider the set X^ω as a metric space (Cantor space) (X^ω, ρ) of all ω-words over the alphabet X where the metric ρ is defined as follows.

$$\rho(\xi, \eta) := \inf\{r^{-|w|} : w \sqsubset \xi \wedge w \sqsubset \eta\}$$

This space is a compact, and $\mathcal{C}(F) := \{\xi : \mathbf{A}(\xi) \subseteq \mathbf{A}(F)\}$ is the *closure* of the set F (smallest closed subset containing F) in (X^ω, ρ).

The mapping $\phi_w(\xi) := w \cdot \xi$ is a contracting similitude if only $w \neq e$. Thus a language $W \subseteq X^* \setminus \{e\}$ defines a possibly infinite IFS (IIFS) in (X^ω, ρ). Its (maximal) *fixed point* is the ω-power W^ω of the language W. It was observed in [Staiger 1997b] that, in general, the IIFS $(\phi_w)_{w\in W}$ has a great variety of fixed points, that is, solutions of the equation $\bigcup_{w\in W} \phi_w(F) = F$. All of these fixed points are contained in W^ω, and, except for the empty set $\emptyset$, their closure equals $\mathcal{C}(W^\omega)$, which is the *attractor* of $(\phi_w)_{w\in W}$.

If $e \notin W$ and W is prefix-free its ω-power satisfies $W^\omega = \bigcap_{i\in\mathbb{N}} W^i \cdot X^\omega$, that is, is a G_δ-set (a countable intersection of open sets) in (X^ω, ρ). In general, the topological structure of W^ω can be more complex (cf. [Finkel 2001, Staiger 1997a, Staiger 1997b]).

Next we recall the definition of the Hausdorff measure and Hausdorff dimension of a subset of (X^ω, ρ) (see [Edgar 1990, Falconer 1990]). In the setting of languages and ω-languages this can be read as follows (see [Staiger 1993, Merzenich and Staiger 1994]). For $F \subseteq X^\omega$ and $0 \le \alpha \le 1$ the equation

$$\mathbb{L}_\alpha(F) := \lim_{l\to\infty} \inf\Big\{ \sum_{w\in W} r^{-\alpha\cdot|w|} : F \subseteq W \cdot X^\omega \wedge \forall w(w \in W \rightarrow |w| \ge l)\Big\} \quad (2)$$

defines the α-dimensional metric outer measure on X^ω. The measure $\mathbb{L}_\alpha$ satisfies the following.

Corollary 2. *If $\mathbb{L}_\alpha(F) < \infty$ then $\mathbb{L}_{\alpha+\epsilon}(F) = 0$ for all $\epsilon > 0$.*

Then the *Hausdorff dimension* of F is defined as

$$\dim F := \sup\{\alpha : \alpha = 0 \vee \mathbb{L}_\alpha(F) = \infty\} = \inf\{\alpha : \mathbb{L}_\alpha(F) = 0\}\,.$$

It should be mentioned that dim is countably stable and shift invariant, that is,

$$\dim \bigcup_{i\in\mathbb{N}} F_i = \sup\{\dim F_i : i \in \mathbb{N}\} \quad \text{and} \quad \dim w \cdot F = \dim F\,. \quad (3)$$

We list some relations of the Hausdorff dimension and measure for ω-power languages to the properties of the structure generation functions of the corresponding languages (see [Staiger 1993, Merzenich and Staiger 1994] or, in a more general setting [Fernau and Staiger 2001]).

$$\dim W^\omega = -\log_r \mathsf{rad}\, W^* \quad (4)$$

Proposition 3. *If $\alpha = \dim W^\omega$ then $\mathbb{L}_\alpha(W^\omega) \le 1$.*

If, moreover, W is a regular language then $0 < \mathbb{L}_\alpha(W^\omega) \le \mathbb{L}_\alpha(\mathcal{C}(W^\omega)) \le 1$, and if W is regular and prefix-free then $\mathbb{L}_\alpha(W^\omega) = \mathbb{L}_\alpha(\mathcal{C}(W^\omega))$.

From [Staiger 1997b] we have the following connection between finite-state and regular ω-powers.

Proposition 4. *If V^ω is finite-state then $\mathcal{C}(V^\omega)$ is regular and there is a regular language W such that $\mathcal{C}(V^\omega) = \mathcal{C}(W^\omega)$.*

The following direct connections between the structure generation function $\mathfrak{s}_W$ and Hausdorff measure $\mathbb{L}_\alpha(W^\omega)$ or $\dim W^\omega$ are helpful.

Proposition 5. *1. If $\mathfrak{s}_W(r^{-\alpha}) < 1$ then $\mathbb{L}_\alpha(W^\omega) = 0$.*

2. If W is prefix-free and $\mathfrak{s}_W(r^{-\alpha}) = 1$ then $\alpha = \dim W^\omega$.

3 Properties of Łukasiewicz languages

In this section we recall known and derive some new properties of Łukasiewicz languages. We start with the definition of the $\{C, B\}$-n-Łukasiewicz language (cf. [Staiger 2005b]). Let $C, B \subseteq X^* \setminus \{e\}$ be two disjoint languages. Then

$$Ł = C \cup B \cdot Ł^n \tag{5}$$

is the n-Łukasiewicz language derived from C and B. Closely related to Ł is its derived language defined as follows.

$$\mathrm{K} = \bigcup_{i=0}^{n-1} B \cdot Ł^i \,. \tag{6}$$

The languages Ł and K have the following properties (see [Staiger 2005b]).

Proposition 6. *Let $C \cap B = \emptyset$.*

1. $Ł \subseteq (C \cup B)^* \cdot C \subseteq (C \cup B)^*$
2. $\mathbf{A}(Ł^*) = \mathbf{A}((C \cup B)^*)$
3. *If $C \cup B$ is prefix-free then Ł is also a prefix-free and K is the union of n prefix-free languages $B \cdot Ł^i$ $(i = 0, \ldots, n-1)$.*
4. $\mathbf{A}(Ł) = \mathrm{K}^* \cdot \mathbf{A}(C \cup B)$ *and if Ł is prefix-free then* $\mathrm{K}^* \subseteq \mathbf{A}(Ł) \setminus Ł$.
5. $(C \cup B)^* = Ł^* \cdot \mathrm{K}^*$, *and if $C \cup B$ is prefix-free every $w \in (C \cup B)^*$ has a unique factorisation $w = v \cdot u$ where $v \in Ł^*$ and $u \in \mathrm{K}^*$.*

Since $\mathbf{A}(W^* \setminus \{e\}) = \mathbf{A}(W^\omega)$ from Proposition 6.2 we have the following.

$$\mathcal{C}((C \cup B)^\omega) = \mathcal{C}(Ł^\omega) \tag{7}$$

Under certain assumptions we can express $(C \cup B)^\omega$ in terms of Ł and K.

Theorem 7. *Let $C, B \subseteq X^*$ be disjoint, $C \cup B$ prefix-free and Ł and K defined as in Eqs. (5) and (6), respectively. Then*

$$(C \cup B)^\omega = Ł^\omega \cup Ł^* \cdot \mathrm{K}^\omega \text{ and} \tag{8}$$

$$I\!\!L_\alpha\left((C \cup B)^\omega\right) = I\!\!L_\alpha\left(Ł^\omega\right) + \sum_{i \in \mathbb{N}} \mathfrak{s}_Ł(r^{-\alpha})^i \cdot I\!\!L_\alpha\left(\mathrm{K}^\omega\right) \tag{9}$$

Before we proceed to the proof we need some preparatory considerations which can be found e.g. in [Staiger 1997b].

Let $W^\delta := \{\xi : \mathbf{A}(\xi) \cap W \text{ is infinite}\}$. This δ-limit and the ω-power of W are related via the following equations.

$$(W \cdot V)^\delta = W \cdot V^\delta \cup W^\delta \text{ if } e \in V \tag{10}$$

$$(W^*)^\delta = W^\omega \cup W^* \cdot W^\delta \tag{11}$$

Proof. First observe that, since $C \cup B$ is prefix-free, we have $|\mathbf{A}(\xi) \cap (C \cup B)| \leq 1$ and from Proposition 6.3 we have that $|\mathbf{A}(\xi) \cap \mathrm{K}| \leq n$ for arbitrary $\xi \in X^\omega$. Thus $(C \cup B)^\delta = \mathrm{K}^\delta = \emptyset$ and from Eq. (11) $((C \cup B)^*)^\delta = (C \cup B)^\omega$ and $(\mathrm{K}^*)^\delta = \mathrm{K}^\omega$ follow.

Now we apply Eq. (10) to Proposition 6.5, and we obtain Eq. (8) $(C \cup B)^\omega = Ł^\omega \cup Ł^* \cdot \mathrm{K}^\omega$.

Since Ł is also prefix-free, Proposition 6.4 implies $Ł^* \cdot \mathrm{K}^\omega \cap Ł^\omega = \emptyset$ and also $Ł^i \cdot \mathrm{K}^\omega \cap Ł^j \cdot \mathrm{K}^\omega = \emptyset$ for $i \neq j$. Then Property 2 of [Merzenich and Staiger 1994] proves Eq. (9). $\square$

Next we investigate the relations between $(C \cup B)^\omega$ and $Ł^\omega$. It turns out that the value of $\mathfrak{s}_Ł(\mathsf{rad}\,(C \cup B)^*)$ plays a crucial rôle in this respect.

To this end we recall the following properties of $\mathsf{rad}\, Ł^*$ which can be found in Section 4 of [Staiger 2005b].

Proposition 8. *Let $C \cap B = \emptyset$ and $C \cup B$ prefix-free. Then*

1. $\mathsf{rad}\, C \cup B \geq \mathsf{rad}\, Ł \geq \mathsf{rad}\, Ł^* \geq \mathsf{rad}\,(C \cup B)^*$
2. *It holds* $\mathsf{rad}\, Ł^* = \mathsf{rad}\,(C \cup B)^*$ *or* $\mathsf{rad}\, Ł^* = \mathsf{rad}\, Ł$.
3. $\mathfrak{s}_Ł(t) \leq \mathfrak{s}_{C \cup B}(t) \leq 1$ *for* $0 \leq t \leq \mathsf{rad}\,(C \cup B)^*$.
4. *If* $\mathfrak{s}_Ł(t) = 1$ *for some* $0 \leq t \leq \mathsf{rad}\, Ł$ *then we have also* $\mathfrak{s}_{C \cup B}(t) = 1$.

As a corollary we obtain the following.

Corollary 9. *If* $\mathfrak{s}_Ł(\mathsf{rad}\,(C \cup B)^*) < 1$ *then* $\mathfrak{s}_Ł(t) < 1$ *for* $0 \leq t \leq \mathsf{rad}\, Ł$.

For the sake of completeness we give a short proof.

Proof. If $t \leq \mathsf{rad}\,(C \cup B)^*$ the assertion is trivial. In case $t > \mathsf{rad}\,(C \cup B)^*$ we obtain from $\mathfrak{s}_Ł(t) = 1$ via Proposition 8.4 that $\mathfrak{s}_{C \cup B}(t) = 1$ which is impossible, since $\mathfrak{s}_{C \cup B}$ is strictly increasing. $\square$

This much of preparations yields the following results. Observe that $\dim W^\omega = -\log_r \mathsf{rad}\, W^*$ in view of Eq. (4).

Lemma 10. *Let $C \cap B = \emptyset$ and $C \cup B$ prefix-free.*

1. *If* $\mathfrak{s}_Ł(\mathsf{rad}\,(C \cup B)^*) = 1$ *then* $\dim Ł^\omega = \dim (C \cup B)^\omega$ *and* $\mathbb{L}_\alpha(Ł^\omega) = \mathbb{L}_\alpha((C \cup B)^\omega)$ *for* $\alpha = \dim Ł^\omega$.
2. *If* $\mathsf{rad}\, C \cup B = \mathsf{rad}\,(C \cup B)^*$ *then* $\dim Ł^\omega = \dim (C \cup B)^\omega$.
3. *If* $\mathsf{rad}\, C \cup B > \mathsf{rad}\,(C \cup B)^*$ *and* $\mathfrak{s}_Ł(\mathsf{rad}\,(C \cup B)^*) < 1$ *then* $\dim Ł^\omega < \dim (C \cup B)^\omega$ *and* $\mathbb{L}_\alpha(Ł^\omega) = 0$ *for* $\alpha = \dim Ł^\omega$.

Proof. The first property follows from Eq. (9) and Proposition 3, and the second is an immediate consequence of Proposition 8.1.

In order to prove the third one observe that in view of Lemma 1 $\mathsf{rad}\, C \cup B > \mathsf{rad}\,(C \cup B)^*$ implies $\mathfrak{s}_{C\cup B}(\mathsf{rad}\,(C \cup B)^*) = 1$. Now by the results of the table in Section 4.3 of [Staiger 2005b] $\mathfrak{s}_{\mathrm{Ł}}(\mathsf{rad}\,(C\cup B)^*) < 1$ implies $\mathfrak{s}_B(\mathsf{rad}\,(C\cup B)^*) > \frac{1}{n}$. Then $\mathfrak{s}_C(\mathsf{rad}\,(C \cup B)^*) < \frac{n-1}{n}$ and $\mathfrak{s}_C(\mathsf{rad}\,(C \cup B)^*)^{n-1} \cdot \mathfrak{s}_B(\mathsf{rad}\,(C \cup B)^*) < \frac{(n-1)^{n-1}}{n^n}$.

The functions $\mathfrak{s}_C(t), \mathfrak{s}_B(t)$ are continuous and increasing in $[0, \mathsf{rad}\, C \cup B)$. Consequently, $\mathfrak{s}_C(\mathsf{rad}\,(C \cup B)^* + \varepsilon)^{n-1} \cdot \mathfrak{s}_B(\mathsf{rad}\,(C \cup B)^* + \varepsilon) < \frac{n-1}{n}$ for some $\varepsilon > 0$ and Eq. (21) of [Staiger 2005b] gives the following estimate $\mathsf{rad}\,\mathrm{Ł} = \sup\{t : \mathfrak{s}_C(t)^{n-1} \cdot \mathfrak{s}_B(t) \leq \frac{(n-1)^{n-1}}{n^n}\} \geq \mathsf{rad}\,(C \cup B)^* + \varepsilon$.

Now, Corollary 9 shows $\mathfrak{s}_{\mathrm{Ł}}(t) < 1$ for $0 \leq t \leq \mathsf{rad}\,\mathrm{Ł}$ whence, using again Lemma 1 we obtain $\mathsf{rad}\,\mathrm{Ł}^* = \mathsf{rad}\,\mathrm{Ł} > \mathsf{rad}\,(C \cup B)^*$.

Finally, the assertion $I\!L_\alpha\,(\mathrm{Ł}^\omega) = 0$ follows from $\mathfrak{s}_{\mathrm{Ł}}(\mathsf{rad}\,\mathrm{Ł}^*) < 1$. □

From the preceding consideration the following corollary is immediate.

Corollary 11. *Let $C \cap B = \emptyset$ and $C \cup B$ prefix-free. Then* $\mathfrak{s}_{\mathrm{Ł}}(\mathsf{rad}\,(C \cup B)^*) < 1$ *if and only if* $\mathfrak{s}_{C\cup B}(\mathsf{rad}\,(C \cup B)^*) < 1$ *or* $\mathfrak{s}_{\mathrm{Ł}}(\mathsf{rad}\,(C \cup B)^*) = 1$ *and* $\mathfrak{s}_B(\mathsf{rad}\,(C \cup B)^*) > \frac{1}{n}$.

4 The Łukasiewicz Construction

This last section is devoted to the construction of the our examples. In [Staiger 2005a] we considered twelve cases for the relations between the dimensions $\alpha = \dim V^\omega$, $\hat{\alpha} = \dim \mathcal{C}(V^\omega)$ and the corresponding measures $I\!L_\alpha\,(V^\omega)$ and $I\!L_{\hat{\alpha}}\,(\mathcal{C}(V^\omega))$ of the fixed point and the attractor of IIFS derived from a language $V \subseteq X^* \setminus \{e\}$. What concerns $I\!L_\alpha\,(V^\omega)$ and $I\!L_{\hat{\alpha}}\,(\mathcal{C}(V^\omega))$ we distinguished only the three cases of null measure, non-null finite measure and infinite measure. Due to the constraints $\alpha \leq \hat{\alpha}$ and $I\!L_\alpha\,(V^\omega) \leq 1 < \infty$ (see Proposition 3) in total twelve cases are possible.

As it was mentioned above the examples found in [Staiger 2005a] are, with the exception of three cases, context-free languages accepted by deterministic one- or two-turn one-counter automata. Only the following ones (L_9, L_{10} and L_{11} in [Staiger 2005a]) were not supported by examples of context-free languages:

1. $\alpha = \hat{\alpha}$, $I\!L_\alpha\,(L_9^\omega) = 0$ and $0 < I\!L_{\hat{\alpha}}\,(\mathcal{C}(L_9^\omega)) < \infty$
2. $\alpha < \hat{\alpha}$, $I\!L_\alpha\,(L_{10}^\omega) = 0$ and $I\!L_{\hat{\alpha}}\,(\mathcal{C}(L_{10}^\omega)) = 0$, and
3. $\alpha < \hat{\alpha}$, $I\!L_\alpha\,(L_{11}^\omega) > 0$ and $I\!L_{\hat{\alpha}}\,(\mathcal{C}(L_{11}^\omega)) = 0$

In the subsequent part of this section we investigate in which cases the results of Section 3 might be helpful to generate less complex examples than L_9, L_{10} and L_{11} mentioned just now.

4.1 The limitations of the construction

First we focus on the the case finite-state fixed points V^ω. This issue was not taken into account in [Staiger 2005a]. Only in the case of regular prefix-free languages V where $\alpha = \hat{\alpha}$ and $0 < I\!L_\alpha(V^\omega) = I\!L_\alpha(\mathcal{C}(V^\omega)) < \infty$ we have a finite-state (even regular) fixed point V^ω in [Staiger 2005a].

If V^ω is finite-state then $\mathcal{C}(V^\omega)$ is a regular ω-language and in view of Proposition 4 we can find a regular language $W \subseteq X^*$ such that $\mathcal{C}(V^\omega) = \mathcal{C}(W^\omega)$. Thus $0 < I\!L_{\widehat{\alpha}}(\mathcal{C}(V^\omega)) \le 1$ for $\widehat{\alpha} = \dim \mathcal{C}(V^\omega)$.

Consequently, for our levels of distinction between fixed point V^ω and attractor $\mathcal{C}(V^\omega)$, we may find examples of finite-state fixed points V^ω only if $0 < I\!L_{\widehat{\alpha}}(\mathcal{C}(V^\omega)) < \infty$.

Next we turn to the limitations of the construction of Łukasiewicz languages. We start with a prefix-free language V and split it into disjoint nonempty parts C and B, choose an $n \in I\!N$, $n \ge 2$ and define Ł according to Eq. (5). Then Eq. (7) shows $\mathcal{C}(V^\omega) = \mathcal{C}(\text{Ł}^\omega)$ independently of the splitting and the choice of the parameter $n \in I\!N$.

What concerns the relation between the dimensions and the measures of Ł^ω and V^ω Lemma 10.1 shows that for $\mathfrak{s}_{\text{L}}(\mathsf{rad}\, V^*) = 1$ these values coincide. Consequently, in this case the Łukasiewicz construction does not yield ω-languages with new parameters.

In order to obtain languages Ł for which at least one of the values $\dim \text{Ł}^\omega$ and $\dim V^\omega$ or $I\!L_\alpha(\text{Ł}^\omega)$ and $I\!L_\alpha(V^\omega)$ differ we have to choose our splitting in such a way that $\mathfrak{s}_{\text{L}}(\mathsf{rad}\, V^*) < 1$. Then by Corollary 9 and Proposition 5.1 we get necessarily $I\!L_\alpha(\text{Ł}^\omega) = 0$ for $\alpha = \dim \text{Ł}^\omega$. Thus it it not to expect to simplify the example of language L_{11} of [Staiger 2005a] (see also Item 3 above).

Moreover, if $\mathfrak{s}_{\text{L}}(\mathsf{rad}\, V^*) < 1$ then Lemma 10 shows that $\mathsf{rad}\, V^* = \mathsf{rad}\, V$ implies $\dim \text{Ł}^\omega = \dim V^\omega$, and $\mathsf{rad}\, V^* < \mathsf{rad}\, V$ implies $\dim \text{Ł}^\omega < \dim V^\omega \le \dim \mathcal{C}(\text{Ł}^\omega)$.

4.2 Examples

The examples presented here are simple deterministic context-free languages (cf. [Autebert et al. 1997]) and yield, in two cases, finite-state fixed points. The third case has $I\!L_{\widehat{\alpha}}(\mathcal{C}(V^\omega)) = 0$. As mentioned before, we can address only two of the above mentioned three items.

We start with an extra example of a Łukasiewicz language Ł showing that Ł^ω is finite-state, $\alpha = \dim \text{Ł}^\omega < \widehat{\alpha} = \dim \mathcal{C}(\text{Ł}^\omega)$, $I\!L_\alpha(\text{Ł}^\omega) = 0$ and $I\!L_{\widehat{\alpha}}(\mathcal{C}(\text{Ł}^\omega)) = 1$. Here the language L_7 in Example 7 of [Staiger 2005a] which is accepted by a deterministic two-turn one-counter automaton defines a fixed point L_7^ω which is not finite-state.

Example 1. (see also Example 6 of [Staiger 1993]) Let $X := \{a, b\}$ and define $Ł_1 = \{a\} \cup b \cdot {Ł_1}^3$. Then $C \cup B = \{a, b\}$ is a regular language, $\mathsf{rad}\,\{a, b\}^* = \frac{1}{2}$ and $\dim \mathcal{C}({Ł_1}^\omega) = 1$.

Now, since $\mathfrak{s}_{C\cup B}(\frac{1}{2}) = 1$ and $\mathfrak{s}_B(\frac{1}{2}) = \frac{1}{2} > \frac{1}{3}$, Corollary 11 and Lemma 10.3 yield $\dim {Ł_1}^\omega < 1$ and $I\!\!L_\alpha\,({Ł_1}^\omega) = 0$.

${Ł_1}^\omega = (\{a\} \cup b \cdot {Ł_1}^3) \cdot {Ł_1}^\omega = \{a, b\} \cdot {Ł_1}^\omega$ proves that ${Ł_1}^\omega / w = {Ł_1}^\omega$ for all $w \in \{a, b\}^*$. Thus ${Ł_1}^\omega$ is finite-state.

Finally, $\mathcal{C}({Ł_1}^\omega) = \{a, b\}^\omega$, whence $I\!\!L_{\widehat{\alpha}}\,(\mathcal{C}({Ł_1}^\omega)) = 1$. □

The next example addresses Item 1. It provides a Łukasiewicz language Ł for the case that $I\!\!L_\alpha\,(Ł^\omega) = 0$ and $I\!\!L_\alpha\,(\mathcal{C}(Ł^\omega)) = 1$ where, additionally, $Ł^\omega$ is finite-state.

Example 2. We let $X := \{a, b\}$ and we start with $C \cup B = V$ where V is the Łukasiewicz language defined by $V = \{a\} \cup b \cdot V^2$. This language has $\mathsf{rad}\, V = \mathsf{rad}\, V^* = \frac{1}{2}$ and $\mathfrak{s}_V(\frac{1}{2}) = 1$ (see [Kuich 1970]).

We define $Ł_2 = (V \setminus \{a\}) \cup a \cdot {Ł_2}^3$. Since $\mathfrak{s}_B(\frac{1}{2}) = \frac{1}{2} > \frac{1}{3}$, from Corollary 11 and Lemma 10.2 we have $\alpha = \dim {Ł_2}^\omega = \dim V^\omega = 1$ and $I\!\!L_\alpha\,({Ł_2}^\omega) = 0$.

In order to show that ${Ł_2}^\omega$ is finite-state we calculate ${Ł_2}^\omega = ((V \setminus \{a\}) \cup a \cdot {Ł_2}^3) \cdot {Ł_2}^\omega = V \cdot {Ł_2}^\omega$. This yields $\{a, b\} \cdot {Ł_2}^\omega = a \cdot {Ł_2}^\omega \cup b \cdot V^2 \cdot {Ł_2}^\omega = V \cdot {Ł_2}^\omega = {Ł_2}^\omega$.

As in the previous example we have also $\mathcal{C}({Ł_2}^\omega) = \{a, b\}^\omega$ and, therefore, $I\!\!L_\alpha\,(\mathcal{C}({Ł_2}^\omega)) = 1$.

Finally, we show that the language $Ł_2$ is a simple deterministic context-free language giving a corresponding grammar $(\{a, b\}, \{S, A\}, S, P)$ with rules P:

$$S \to a \cdot SSS \mid b \cdot AA$$
$$A \to a \mid b \cdot AA$$

□

The last example provides a Łukasiewicz language Ł for which $\alpha = \dim Ł^\omega < \widehat{\alpha} = \dim \mathcal{C}(Ł^\omega)$ and $I\!\!L_\alpha\,(Ł^\omega) = I\!\!L_{\widehat{\alpha}}\,(\mathcal{C}(Ł^\omega)) = 0$, thus addressing Item 2.

Example 3. We start with the language $V := \{\widetilde{d}^{3|w|} w : w \in \{a, b\}^* \setminus \{e\}\} \subseteq \{a, b, d, \widetilde{d}\}^*$ from Example 2 of [Staiger 2005a]. For this language, it is shown that $\mathsf{rad}\, V > \mathsf{rad}\, V^* = \frac{1}{\sqrt{2}}$, $\mathfrak{s}_V(\mathsf{rad}\, V^*) = 1$, $\widehat{\alpha} = \dim V^\omega = \dim \mathcal{C}(V^\omega) = \frac{1}{4}$ and $I\!\!L_{\widehat{\alpha}}\,(V^\omega) = I\!\!L_{\widehat{\alpha}}\,(\mathcal{C}(V^\omega)) = 0$

If we split $V = C \cup B$ with $B := \{\widetilde{d}^3 a, \widetilde{d}^3 b\}$ and $C := V \setminus B$ we have $\mathfrak{s}_B(\mathsf{rad}\, V^*) = \frac{1}{2}$. Then according to Corollary 11 and Lemma 10.3 the language $Ł_3$ defined by $Ł_3 = C \cup B \cdot {Ł_3}^3$ satisfies $\dim {Ł_3}^\omega < \widehat{\alpha} = \dim \mathcal{C}({Ł_3}^\omega)$ and $I\!\!L_\alpha\,({Ł_3}^\omega) = 0$.

Again we show that the language $Ł_3$ is a simple deterministic context-free language by giving a corresponding grammar $(\{a, b, d, \widetilde{d}\}, \{S, S', A, B\}, S, P)$:

$$P: S \to \widetilde{d}^3 \cdot S' \qquad S' \to a \cdot S^3 \mid b \cdot S^3 \mid \widetilde{d}^3 \cdot AB$$
$$A \to \widetilde{d}^3 \cdot AB \mid a \mid b \qquad B \to a \mid b$$

As it is clear from the discussion above the ω-language $Ł_3{}^\omega$ in Example 3 cannot be finite-state. □

4.3 Concluding Remark

On the one hand, our Examples 2 and 3 improve the results of Examples 9 and 11 of [Staiger 2005a], because the languages L_9 and L_{11} given there were not even context-free, and on the other hand, Examples 1 and 2 give a new insight by constructing languages Ł for which the fixed point $Ł^\omega$ is finite-state. This is another indication for the fact observed in [Kuich 1970, Staiger 2005b] that Łukasiewicz languages have remarkable information-theoretic properties.

References

[Autebert et al. 1997] Autebert, J.-M., Berstel, J., Boasson, L.: "Context-free languages and pushdown automata", In [Rozenberg and Salomaa 1997], Vol. 1, 111–174.

[Bandt 1989] Bandt, C.: "Self-similar sets 3. Constructions with sofic systems"; Monatsh. Math., 108 (1989), 89–108.

[Berstel and Perrin 1985] J. Berstel J., Perrin, D.: "Theory of codes"; Academic Press, Orlando (1985).

[Edgar 1990] Edgar G. A.: "Measure, topology, and fractal geometry"; Springer, New York (1990).

[Falconer 1990] K.J. Falconer, K.J.: "Fractal geometry"; Wiley, New York (1990).

[Fernau 1994a] Fernau, H.: "Iterierte Funktionen, Sprachen und Fraktale"; BI-Verlag, Mannheim (1994).

[Fernau 1994b] Fernau, H.: "Infinite IFS"; Mathem. Nachr., 169 (1994), 79–91.

[Fernau and Staiger 2001] Fernau, H., Staiger, L.: "Iterated function systems and control languages"; Inform. & Comput. 168 (2001), 125–143.

[Finkel 2001] Finkel, O.: " Wadge hierarchy of omega context-free languages"; Theoret. Comput. Sci., 269 (2001), 283–315.

[Hopcroft and Ullman 1979] Hopcroft, J.E., Ullman, J.D.: "Introduction to automata and language theory", Addison-Wesley, Reading MA, (1979).

[Kuich 1970] Kuich, W.: "On the entropy of context-free languages"; Inf. & Contr., 16 (1970), 173–200.

[Mauldin 1995] Mauldin, R.D.: "Infinite iterated function systems: Theory and applications"; In Fractal geometry and stochastics, Vol. 37 of Progress in Probability, Basel, Birkhäuser (1995), 91–110.

[Mauldin and Urbański 1996] Mauldin, R.D., M. Urbański, M.: "Dimensions and measures in infinite iterated function systems"; Proc. Lond. Math. Soc., III. Ser. 73 (1) (1996), 105–154.

[Mauldin and Williams 1988] Mauldin, R.D., Williams S.C.: "Hausdorff dimension in graph directed constructions"; Trans. AMS, 309 (2) (1988) 811–829.

[Merzenich and Staiger 1994] Merzenich, W., Staiger, L.: "Fractals, dimension, and formal languages"; RAIRO – Theoretical Informatics and Applications, 28 (3–4) (1994), 361–386.

[Rozenberg and Salomaa 1997] G. Rozenberg and A. Salomaa (eds.) "Handbook of formal languages"; Springer, Berlin (1997).

[Staiger 1983] Staiger, L.: "Finite-state ω-languages"; J. Comput. System Sci., 27, 3 (1983), 434-448.

[Staiger 1993] Staiger, L.: "Kolmogorov complexity and Hausdorff dimension"; Inform. & Comput., 103 (1993), 159–194.

[Staiger 1997a] Staiger, L.: "ω-languages"; In [Rozenberg and Salomaa 1997], Vol. 3, 339–387.

[Staiger 1997b] Staiger, L.: "On ω-power languages"; In Păun, Gh., Salomaa, A. (eds.): New Trends in Formal Languages, Control, Cooperation, and Combinatorics, Lecture Notes in Comput. Sci., Vol. 1218, Springer, Berlin (1997), 377–393.

[Staiger 2005a] Staiger, L.:"Infinite iterated function systems in Cantor space and the Hausdorff measure of ω-power languages"; Intern. J. Found. Comput. Sci., 16, 4 (2005) 787-802.

[Staiger 2005b] Staiger, L.: "The entropy of Łukasiewicz languages"; RAIRO – Theoretical Informatics and Applications, 39, 4 (2005) 621-640.

[Trakhtenbrot 1962] Trakhtenbrot, B.A.: "Finite automata and monadic second order logic"; Sibirsk. Mat. J. 3 (1962), 103–131. [Russian]
English translation: AMS Transl. 59 (1966), 23–55.
