# Supplementary material for: In-text citation’s frequencies-based recommendations of relevant research papers
Source: PeerJ Comput Sci. 2021 Jun 4;7:e524. doi: 10.7717/peerj-cs.524 (PMC8189020; doi:10.7717/peerj-cs.524)
Supplement: Supplemental Information 2 — These articles are available under a CC BY-ND 4.0 license. [file peerj-cs-07-524-s002.zip › Dataset/Papers Dataset.zip!/Papers Dataset/jucs_1140.pdf]

Journal of Universal Computer Science, vol. 13, no. 10 (2007), 1449-1461
submitted: 12/6/06, accepted: 24/10/06, appeared: 28/10/07 © J.UCS

An Approach to Polygonal Approximation of Digital Curves Based on Discrete Particle Swarm Algorithm

Fangmin Dong, Renbin Xiao, Yifang Zhong
(CAD Center, Huazhong University of Science and Technology, Wuhan, PRC
fmdong@ctgu.edu.cn, rbxiao@163.com, yfzhong@hust.edu.cn)

Yong Liu
(College of Electrical Engineering and Information Technology, China
Three Gorges University, Yichang, PRC
yongliu@ctgu.edu.cn)

Abstract: An approach to polygonal approximation of regular digital curves based on PSO algorithm is presented. In this paper, each particle corresponds to a candidate solution to the polygonal approximation problem, which is represented as a binary vector. The offset error of centroid between the original curve and the approximation polygon, and the variance of distance error for each approximation segment are adopted in the fitness function to evaluate the feasibility degree of the candidate solution. The sigmoid function of iteration times is used as the acceleration factors instead of the constant factors to improve the global searching characteristics. Experimental results show that the proposed approach can get suitable approximation results for preserving the features of original curves.

Key Words: Polygonal approximation, PSO, Centroid, Sigmoid function

Category: I.2.8, I.3.5

1 Introduction

Polygonal approximation of digital curves is an important research content in the fields of model simplification, image processing and analysis, computer vision and so on. Generally, it is solved by taken as an optimization problem, which can be described as follows: to find the minimum number of the vertices in polygon that approximates the original digital curves under the condition of preserving the characteristics of digital curves in acceptable tolerance, or to find the optimal solution that can preserve the characteristics of digital curves (viz. approximation error is smallest) while the number of vertices approximate polygonal is given.

Much research works about polygonal approximation of digital curves and have been done with many algorithms proposed. These algorithms can be classified into the following categories [Yin 2004]: (1) the ones using local optimization method, representative algorithms in which are sequential approaches [Sklansky and Gonzalez 1980], [Kurozumi and Davis 1982],[Ray and Ray 1994],

[Salotti 2002], split-and-merge approaches [Leu and Chen 1988, Ray et al. 1995] and using dominant point-detection approaches [Ray and Ray 1992],[Wu 2003], [Cho-Huak and Roland 1989]. Since they are local optimization methods, the quality of approximating results is dependent upon the location of the initial point and is easily converge to local minimum. (2) The ones using global optimization method, such as dynamic programming algorithm for polygonal approximation of digital curves proposed in references [Horng and Li 2002] and [Kolesnikov and Fränti 2003], but these algorithms with high computational complexity are not fit for the polygon approximation problem that has a large number of vertices. With the appearance of intelligence optimization algorithms such as genetic algorithm, ant colony algorithm and particle swarm optimization, they have been shown to be effective for exploring an NP-complete search space and have popularly attracted research interest for solving various optimization problems [Ho and Chen 2001]. Recently, those intelligence optimization algorithms are also used to the solution of polygonal approximation of digital curves and result in better effects [Yin 2004, Yin 1998, Ru et al. 2004, Ho and Chen 2001, Yin 2003]. From the existed documents at present, most of these algorithms take quadratic sum (or mean variance sum, area difference) of Euclid distances between vertices of the original curve and the corresponding line segments of approximation polygon as fitness functions. However, seldom algorithm can control the error of feature characteristic changes of shapes of approximation polygon and can preserve the characteristic shapes of digital curves with uniform geometry, especially for symmetric shapes.

Aiming at the features preserving in the process of polygon simplified approximation, an improved algorithm is proposed based on the one in the literature [Yin 2004] for mesh model simplification in this paper, in which the offset error of centroid between the original curve and the approximation polygon, the variance of distance of every replaced line segment in the process of simplification are adopted in the fitness function. Further, an improved discrete particle swarm optimization algorithm is used to obtain the optimal approximate solution of polygon approximation of digital curve under the condition of given number of vertices.

2 Problem Description

The problem of polygon approximation of digital curve can be classified into two categories: the first one is to solve the least error in approximated polygon with given number of vertices; another one is to solve the least number of vertices while least error is given. In model simplification the process in this paper, the first kind of polygon approximation needs to be solved, which can be described as follows: suppose the original digital curve $C = \{P_1, P_2, \ldots, P_n\}$ as a closed

curve which composed of P_i, $i = 1, 2, \ldots, n$, then approximate polygon $Ca = \{P_{a1}, P_{a2}, \ldots, P_{am}\}$ of the curve C is composed of the number of m order vertices P_{ai}, $i = 1, 2, \ldots, m$, and satisfy the condition of $Ca \subset C$and least error E, m is the given number of vertices of simplified approximate polygon; E is the total error between approximate polygon and original digital curve, generally using quadratic sum, mean variance sum, or area difference of distances from vertices of the original curve to the corresponding line section of approximation polygon to estimate.

The optimization model is:

$$\min \mid E \mid$$
$$\text{s.t. } \begin{cases} 3 \leq n_p \leq m \\ Ca \subset C \end{cases} .$$

where E is the total error, n_p is the number of vertices of simplified polygon, m is the number of vertices of the given approximate polygon, C and Ca are original digital curve composed of order vertices and simplified approximate polygon respectively.

3 The Solution Polygonal Approximation by Means of Discrete Particle Swarm Algorithm

3.1 Discrete Particle Swarm Algorithm

The particle swarm optimization (PSO) algorithm was proposed by Kennedy and Eberhart (1995), and had been successfully applied to solution of several kinds of combinatorial optimization problems in many fields such as energy, electric power, manufacture. Generally, PSO algorithm is used to solve the optimization problems of continuous function, applications using the improved discrete PSO algorithm proposed by Kennedy and Eberhart (1997) to solve discrete combinatorial optimization problem is also increasing.

The general principle for the PSO algorithms are stated as follows [Yin 2004]. Given an optimization function $f(P)$ where P is a vector of n real-valued random variables, the PSO initializes a swarm of particles, each of which is consisted of $P_i = \{p_{i1}, p_{i2}, \ldots, p_{in}\}$, $i = 1, 2, \ldots, k$, where k is the swarm size. Thus, each particle is a candidate solution to the optimization function and is randomly positioned in the n-dimensional real number space. The PSO is an evolutionary computation algorithm and in the process of generation$g$, $pbest_i$ remembers the best position that particle $i$has visited so far, and $gbest$ remembers the swarm's best position that the swarm have visited so far, $g = 1, 2, \ldots, N$, where N is the given cycle index of biggest iteration, then particle$i$ adjusts its velocity v_{ij} and position p_{ij} by referring to the best positions using Eq. (1) and (2) as follows:

$$v_{ij} = wv_{ij} + c_1 r_1 (pbest_{ij} - p_{ij}) + c_2 r_2 (gbest_j - p_{ij}) \tag{1}$$

$$p_{ij} = p_{ij} + v_{ij} \tag{2}$$

where r_1 andr_2 are random real numbers drawn from $U(0,1)$; $i = 1, 2, \ldots, k$, where k is the swarm size; $j = 1, 2, \ldots, n$, n is the number of vertices of original curve; p_{ij} and v_{ij} are components of dimension j which are position and velocity of particle i respectively, $pbest_{ij}$ is component of dimension j which is the best position of particle j; $gbest_j$ is component of dimension j, which is the best position of the whole particle swarm; w is the parameter i that controls convergence velocity, c_1 and c_2 are parameters of global search and local search respectively, here, set $w = 1$.

In this paper, sigmoid function is used to calculate the value of c_1, c_2:

$$c_1 = \frac{2}{1 + e^{\alpha(-i+\frac{N}{2})}}, \quad c_2 = 4 - c_1. \tag{3}$$

where i is the number of iterations, N is the given cycle index of biggest iteration, α is a constant, and is set to 0.05 in this paper. The curve corresponding to Eq. (3) is shown in Figure1, from which it can seen that the initial value of the parameter of local search c_2 approaches to "0", with the increase of the number of iterations the value increases gradually and approaches to "2" finally; the value of c_1 decreases gradually from the initial value "4" and approach to "2" finally correspondingly; constant α controls the rate of speed of the increasing c_2. Then it can guarantee the PSO algorithm has more global search characteristics in early iterations and thus can avoid premature of the PSO algorithm and convergent to local optimal solution to the greatest extent. In the final period of the algorithm, it has much characteristics of local search that can guarantee a given convergence velocity.

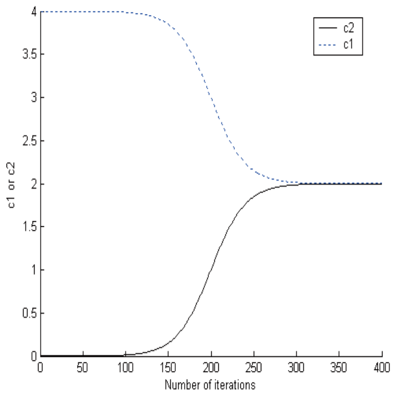

Figure 1: Curve of sigmoid function. Constant c_1 or c_2 versus number of iterations.

In order to avoid missing the optimal solution while the particles fly so fast in the evolutionary process, generally set the thresholds of the evaluated velocity, in this paper, absolute value of V_{max} is set to 4 [Huang et al. 2005].

For discrete PSO algorithm, Kennedy and Berhart use the length of n binary vector to present the particle position and use Eq. (4) to replace Eq. (2), according to the velocity of particle to determine the possibilities of getting value "1" of the n binary vector.

$$p_{ij} = \begin{cases} 0,\, r_3 \geq S(v_{ij}) \\ 1,\, r_3 < S(v_{ij}) \end{cases} . \tag{4}$$

where r_3 is random real numbers drawn from $U(0,1)$, in this paper, s is used to denote Sigmoid function [Huang et al. 2005]

$$S(v_{ij}) = \frac{1}{1 + \exp(-v_{ij})}.$$

3.2 Discrete Particle Swarm Algorithm for Polygonal Approximation

Description of polygonal approximation problem by means of discrete particle swarm algorithm [Yin 2004].

The solution of polygonal approximation problem is the process that suitable vertices are selected from all vertices as the vertices of approximate polygon. In PSO algorithm, each particle is presented as a candidate solution. When using discrete PSO for solving the polygonal approximation problem, the original curve can be presented as that the binary vector sequence are set "1" and the length is the number of the original curve. Each particle is taken as the candidate solution in the iterations, when the vertex of the original curve is selected as a candidate solution, the value of corresponding bits are set "1" else "0", that is

$$P_i = (p_{i1}, p_{i2}, ..., p_{in}), \quad p_{ij} \in \{0, 1\} . \tag{5}$$

when given the number of polygon's vertices, it should satisfy

$$3 \leq \sum_{j=1}^{n} p_{ij} \leq m. \tag{6}$$

where $i = 1, 2 \ldots k$, $j = 1, 2 \ldots n$, k is the swarm size, n is the number of vertices of original curve.

The digital curve $C = \{P_1, P_2, \ldots, P_n\}$ consist of n sequential vertices P_i, $i = 1, 2, \ldots, n$, is used to present a part of curve which consist of vertices of P_i, P_{i+1}, $\ldots$, P_j, $\overline{P_i P_j}$ is used to present a line section whose vertices are P_i andP_j, so the quadratic sum of vertical distance from each vertex P_{i+1}, $\ldots$, P_{j-1} to line section $\overline{P_i P_j}$ is used to present the error Ed when $\overline{P_i P_j}$ is taken to

approximate. Given the number of vertices of approximate polygon, total error of the approximate polygon is

$$Ed = \sum_{i=1}^{m} Ed_i. \tag{7}$$

In order to make the error of each line section of curve even, variance Es of the error of each line section is used

$$Es = \frac{1}{m}\sum_{i=1}^{m}(Ed_i - \frac{Ed}{m})^2. \tag{8}$$

In order to better preserve the main shape characteristics after approximation for the curve of regular curves, especially symmetry curves, to simplify the original curve and approximate curve and the offset error of centroid of polygon is considered, quadratic of distance from centroid coordinate of original curve to centroid coordinate of approximate polygon to present is also used.

Set the coordinate of vertex $P_i(x_i,y_i)$, $i = 1, 2, \ldots, n$, the number of vertices of digital curve is n, thus the centroid of $G(x_g,y_g)$ is

$$x_g = \frac{Sx + x_n^2 y_1 - x_1^2 y_n + x_1 x_n y_1 - x_1 x_n y_n}{3(\sum_{i=1}^{n-1}(x_i y_{i+1} - x_{i+1} y_i) + x_n y_1 - x_1 y_n)}$$

$$y_g = \frac{Sy + x_n y_1^2 - x_1 y_n^2 + x_n y_1 y_n - x_1 y_1 y_n}{3(\sum_{i=1}^{n-1}(x_i y_{i+1} - x_{i+1} y_i) + x_n y_1 - x_1 y_n)},$$

where

$$Sx = \sum_{i=1}^{n-1}(x_i^2 y_{i+1} - x_{i+1}^2 y_i + x_i x_{i+1} y_{i+1} - x_i x_{i+1} y_i)$$

$$Sy = \sum_{i=1}^{n-1}(x_i y_{i+1}^2 - x_{i+1} y_i^2 + x_i y_i y_{i+1} - x_{i+1} y_i y_{i+1}) .$$

The error of centroid Eg

$$E_g = (x_{gn} - x_{gm})^2 + (y_{gn} - y_{gm})^2. \tag{9}$$

where $(x_{gn},\ y_{gn})$ and $(x_{gm},\ y_{gm})$ are centroid coordinate of original curve and approximate polygon respectively.

The *fitness* (P_i) in our discrete PSO algorithm is weighted sum of the above three errors

$$fitness(P_i) = c_4 Ed + c_5 Es + c_6 Eg. \tag{10}$$

where c_4, c_5 and c_6 are constants of weighed factors, in this paper, let $c_4 = c_5 = 1$, $c_6 = 10m, m$ is a given number of vertices of approximate polygon. Weighted factors can be adjusted according to the fact, for example, for the approximation problem of free curves c_6 can be set smaller.

3.3 Implementation of the Algorithm

Step1. Initialize position and initial velocity of each particle in the swarm.

Step1.1. Generate initial velocities of k particles randomly according to Eq. (5) and Eq. (6). In this paper, let $k = 30$, and m positions are randomly select from n bits and then set "1" to generate initial values of every particle

Step1.2. Generate component values v_{ij} of every particle's initial velocities, $i = 1, 2 \ldots k$ and $j = 1, 2 \ldots n$, where v_{ij} is randomly drawn from [-v_{max}, v_{max}]

Step2. Repeat the following steps until a given maximal number of iterations is achieved and then go to step3

Step2.1. Calculate *fitness* (P_i) values of each particle in terms of Eq. (7), Eq. (8), Eq. (9) and Eq. (10), select the particle position which has the smaller fitness value visited so far through the evolutionary process as the best position $pbest_i$, $i = 1, 2, \ldots, k$. Select the particle position which has the smallest fitness value of all the particles as the best position *gbest* visited so far of the whole swarm.

Step2.2. Calculate v_{ij} of each particle, and use -v_{max}, v_{max} to replace the value smaller than -v_{max} and the value bigger than v_{max} respectively.

Step2.3. Calculate p_{ij} of each particle in terms of Eq. (4), $i = 1, 2 \ldots k$, $j = 1, 2 \ldots n$.

Step3. Take the best position *gbest* of the whole swarm as the approximate result, select the vertices which corresponding to value "1" in vector string sequentially as vertices of approximate polygon; the algorithm ends.

4 Experimental Results and Analysis

4.1 Experimental Results and Analysis of General Curves

4.1.1 Experimental Results

In this section, leaf and chromosome digital curves, which are frequently used in polygon simplification algorithms, are taken as examples to show the approximate effectiveness of the algorithm when adjust the acceleration factor and fitness function. The number of vertices of original leaf and chromosome digital curves is $n = 117$, 60 respectively, the number of vertices of approximate polygon is $m = 32$, 10 individually, the swarm size is k= 20, and the number of iterations is N= 400.

Experimental results are presented in Figure 2. Figure 2(c) and Figure 2(g) are the approximate results where acceleration factor are adjusted but do not calculate the variance of distance error and offset centroid, Figure 2(d) and Figure 2(h) take *Ed* as fitness function. Figure 3 is the comparison of convergence velocity and results of above three algorithms for leaf digital curve simplification. In this paper, three algorithms are run ten times respectively and average values

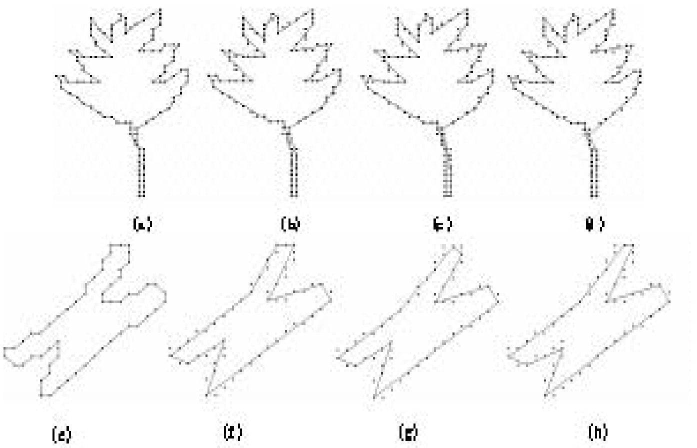

Figure 2: Comparison of the results of polygon approximation. (a) and (e) Original curves. (b) The approximate results using our algorithm, where $c_4 = c_5 = 1$, $c_6 = 320$, $e = 3.7$. (c) Approximate results using variational acceleration factor, where $c_4 = 1$, $c_5 = c_6 = 0$, $e = 3.9$. (d) Approximate results using algorithm in the literature [Yin 2004] , where $c_1 = c_2 = 2$, $c_4 = 1$, $c_5 = c_6 = 0$, $e = 4.3$. (f) The approximate results using our algorithm, where $c_4 = c_5 = 1$, $c_6 = 100$, $e = 8.9$. (g) Approximate results using variational acceleration factor, where $c_4 = 1$, $c_5 = c_6 = 0$, $e = 9.1$. (h) Approximate results using algorithm in the literature [Yin 2004], where $c_1 = c_2 = 2$, $c_4 = 1$, $c_5 = c_6 = 0$, $e = 10.7$.

of gbest of every algorithm are obtained to analysis. In order to increase the comparability, the values of *Ed* corresponding to *gbest* in fitness function are used to replace the average values of *gbest* (see Eq. (10)). From the experimental results it can be seen that among three algorithms, the approximate error of the best approximate solution of our algorithm has the best approximate effect; the algorithm which only adjust acceleration factors and do not consider the offset centroid and variance of distance error has general effect; approximate error of the algorithm in literature [Yin 2004] has worse effect. About the early 50 iterations, the best solution of the swarms of former two algorithms has faster velocity, but between about 50 iterations to 250 iterations, the best solution of the swarms of the latter algorithm exceed the former two and when comes to the 192 iteration, it converges to the approximate best solution, in comparison with 300 iterations of the former two algorithms.

4.1.2 Results Analysis

From results, the algorithm in literature [Yin 2004] uses constants of acceleration factor and convergence velocity of which is much even and the whole convergence

velocity is fast but easily converge to local optimal solution because of premature of the algorithm; the algorithm of adjustable acceleration factor avoid converging to local optimal solution due to the premature thus can improve the solution, because c_1, c_2 vary with the number of iterations and can control its velocity and can also control global and local characteristics in different stages flexibly. Referring to improvement of fitness function, controlling the offset error of centroid of polygon and the variance of distance for irregular digital curves can improve the accuracy of the best solution to some extent but the whole convergence velocity will decrease.

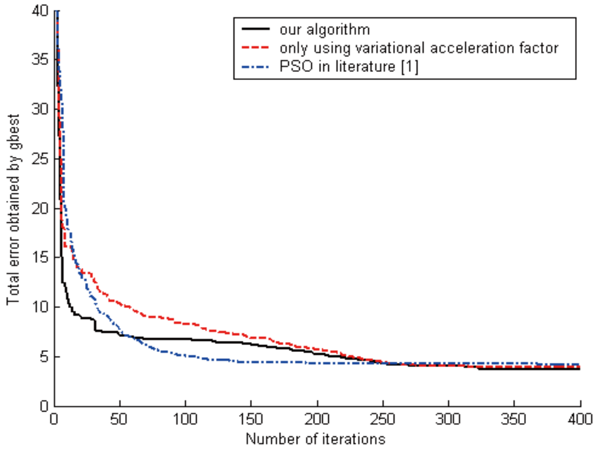

Figure 3: The comparison of approximate errors. Total error obtained by *gbest* versus number of iterations.

4.2 Experimental Results and Analysis of Regular Curves

Contour curves of three-dimension mesh model of a certain part are selected as our experimental curves. The number of vertices of original curve and approximate polygon in Figure 4 is $n = 60$ and $m = 12$ respectively. The swarm size is $k = 40$ and the number of iterations is $N = 400$. The number of vertices of original curve in Figure 5 is $n = 46$, then compare with the number of vertices of approximate polygon $m = 11$, 7 and 6 individually, the swarm size is $k = 20$ and the number of iterations is $N = 400$.

4.2.1 Experimental Results

Experimental results are presented in Figure 4 - Figure 7. Figure 4 is the comparison of errors of approximate polygon results of three algorithms. Figure 5

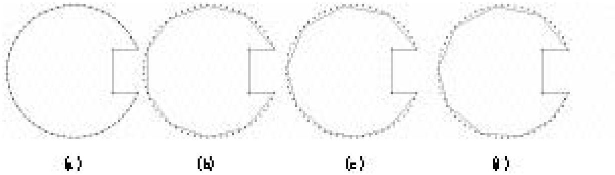

Figure 4: Comparison of the results of polygon approximation. (a) Original curves. (b) The approximate results using our algorithm, where $m = 12$, $c_4 = 1$, $c_5 = 1$, $c_6 = 100$ and $e = 11.78$. (c) Approximate results using variational acceleration factor, where $m = 12$, $c_4 = 1$, $c_5 = 0$, $c_6 = 0$ and $e = 12.83$. (d) Approximate results using algorithm in the literature [Yin 2004], where $m = 12$, $c_4 = 1$, $c_5 = 0$, $c_6 = 0$ and $e = 12.83$.

is the approximate result which using adjustable acceleration factor and fitness function, from left to right are original curve, the number of vertices of approximate polygon m is set to 11, 7 and 6 individually. Figure 6 is the approximate result only using adjustable acceleration factor namely set $c_4 = 1$, $c_5 = c_6 = 0$ in Eq.(10), Figure 7 is the approximate result using the algorithm in literature [Yin 2004], namely using Ed as fitness function and setting $c_1 = c_2 = 2$. Figure 8 is the comparison of convergence velocity and results of above three algorithms, in this paper, three algorithms are run ten times respectively and average values of gbest of every algorithm are obtained to analysis. In order to increase the comparability, the values of Ed corresponding to *gbest* in fitness function are used to replace the average values of *gbest* (see Eq. (10)).

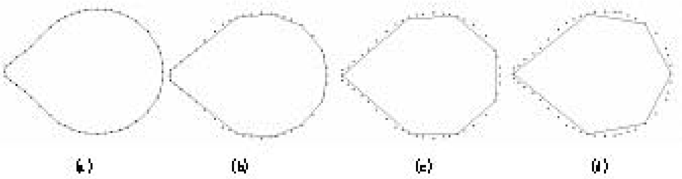

Figure 5: The approximate result using variational acceleration factor and fitness function. (a) Original curve, where $n = 46$. (b) The first approximate result, where $m = 11$, $c_4 = 1$, $c_5 = 0$, $c_6 = 110$, $e = 8.45$. (c) The second approximate result, where $m = 7$, $c_4 = 1$, $c_5 = 0$, $c_6 = 70$, $e = 36.3$. (d) The third approximate result, where $m = 6$, $c_4 = 1$, $c_5 = 0$, $c_6 = 60$, $e = 82.4$.

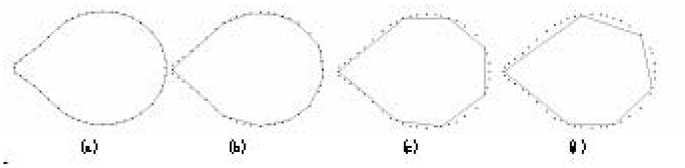

Figure 6: The approximate result only using variational acceleration factor. (a) Original curve, where $n = 46$. (b) The first approximate result, where $m = 11$, $c_4 = 1$, $c_5 = 0$,c_6= 0, $e = 8.4$. (c) The second approximate result, where $m = 7$, $c_4 = 1$, $c_5 = 0$, $c_6 = 0$, $e = 37.0$. (d) The third approximate result, where $m = 6$, $c_4 = 1$, $c_5 = 0$, $c_6 = 60$, $e = 87.8$.

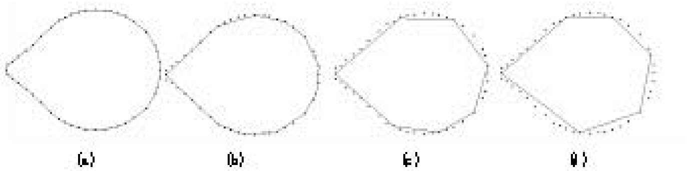

Figure 7: The approximate result using the algorithm in literature [Yin 2004]. (a) Original curve, where $n = 46$. (b) The first approximate result, where $m = 11$, $c_4 = 1$, $c_5 = 0$, $c_6 = 0$, $e = 8.6$. (c) The second approximate result, where $m = 7$, $c_4 = 1$, $c_5 = 0$, $c_6 = 0$, $e = 39.7$. (d) The third approximate result, where $m = 6$, $c_4 = 1$, $c_5 = 0$, $c_6 = 0$, $e = 87.8$.

4.2.2 Results Analysis

From the experimental results it can be seen that among three algorithms, the approximate error of the best approximate solution of our algorithm is small which has the best approximate effect; the algorithm which only adjust acceleration factors and do not consider the offset centroid and variance of distance error has general effect; approximate error of the algorithm in literature [Yin 2004] which has worse effect. In Figure 8, about the early 30 iterations, the best solution of the swarms of former two algorithms has faster velocity, but between about 30 iterations to 250 iterations, the best solution of the swarms of the latter algorithm exceeds the former two and converges to the approximate best solution fast, in comparison with the former two algorithms.

5 Summary

In this paper, the polygonal approximation problem of digital curves is transformed into an optimization problem of discrete sequence and the improved PSO algorithm is used to solve the problem. In order to preserve characteristics of polygon in the process of approximation, especially characteristics of symmetry and equilateral curves, the control of offset error of centroid and variance of error

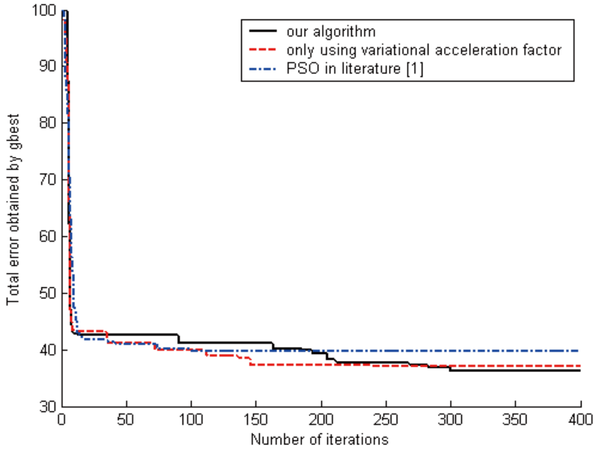

Figure 8: The comparison of approximate errors. Total error obtained by *gbest* versus number of iterations.

are added to the fitness function in PSO algorithm. Further, sigmoid function changed with iterations is used to replace the constant of acceleration factor, then it can guarantee the PSO algorithm has more global search characteristics in early iterations, thus can avoid premature and get local optimal solutions. The experimental results show that this algorithm has better effect for preservation of features of approximate polygon and improvement of approximate solution. However, the automated selection of weighted value of each error component in the fitness function and how to control the change rate of acceleration factor need to be deeply research in the future.

Acknowledgements

The authors would like to thank the reviewers whose suggestions have improved the quality of the paper. This research is partially supported by National Nature Science Foundation of China, under Grant 60474077.

References

[Cho-Huak and Roland 1989] Cho-Huak Teh, Roland T. Chin: "On the Detection of Dominant Points on Digital Curves"; IEEE Transactions on Pattern Analysis and Machine Intelligence, 11, 8(1989), 859-872

[Ho and Chen 2001] Ho S. Y., Chen Y. C.: "An Efficient Evolutionary Algorithm for Accurate Polygonal Approximation"; Pattern Recognition, 34, 12(2001), 2305-2317

[Horng and Li 2002] Horng J. H., Li J. T.: "An Automatic and Efficient Dynamic Programming Algorithm for Polygonal Approximation of Digital Curves"; Pattern Recognition Letters, 23, 1(2002), 171-182

[Huang et al. 2005] Huang Y. X., Zhou C. G., Zou S. X., et al. "A Hybrid Algorithm on Class Covers Problems"; Journal of Software, 16, 4(2005), 513-522

[Kolesnikov and Fränti 2003] Kolesnikov A., Fränti P.: "Reduced-search Dynamic Programming for Approximation of Polygonal Curves "; Pattern Recognition Letters, 24, 14(2003), 2243-2254

[Kurozumi and Davis 1982] Kurozumi Y., Davis W. A.: "Polygonal Approximation by the Minimax Method"; Computer Graphics and Image Processing, 19, 3(1982), 248-264

[Leu and Chen 1988] Leu J. G., Chen L.: "Polygonal Approximation of 2-D Shapes through Boundary Merging"; Pattern Recognition Letters, 7, 4(1988), 231-238

[Ray and Ray 1992] Ray B. K., Ray K. S.: "An Algorithm for Detection of Dominant Points and Polygonal Approximation of Digitized Curves"; Pattern Recognition Letters, 13, 12(1992), 849-856

[Ray and Ray 1994] Ray B. K., Ray K. S.: "A Non-parametric Sequential Method for Polygonal Approximation of Digital Curves"; Pattern Recognition Letters, 15, 2(1994), 161-167

[Ray et al. 1995] Ray B. K., Ray K. S.:"A New Split-and-merge Technique for Polygonal Approximation of Chain Coded Curves"; Pattern Recognition Letters, 16, 2(1995), 161-169

[Ru et al. 2004] Ru S., Zhou M., Geng G.: "Polygonal Approximation of 3D Digitized Curves Using Genetic Algorithms"; Journal of Computer Aided Design & Computer Graphics, 16, 4(2004), 503-507

[Salotti 2002] Salotti M.: "Optimal Polygonal Approximation of Digitized Curves Using the Sum of Square Deviations Criterion"; Pattern Recognition, 35, 2(2002), 435-443

[Sklansky and Gonzalez 1980] Sklansky J., Gonzalez V.: "Fast Polygonal Approximation of Digitized Curves"; Pattern Recognition, 12, 5(1980), 327-331

[Wu 2003] Wu W. Y.: "An Adaptive Method for Detecting Dominant Points"; Pattern Recognition, 36, 10(2003), 2231-2237

[Yin 1998] Yin P. Y.: "A New Method for Polygonal Approximation Using Genetic Algorithms"; Pattern Recognition Letters, 19, 11(1998), 1017-1026

[Yin 2003] Yin P. Y.: "Ant Colony Search Algorithms for Optimal Polygonal Approximation of Plane Curves"; Pattern Recognition, 36, 8(2003), 1783-1797

[Yin 2004] Yin P. Y.,: "A Discrete Particle Swarm Algorithm for Optimal Polygonal Approximation of Digital Curves"; Journal of Visual Communication & Image Representation, 15, 2(2004), 241-260
