# Supplementary material for: In-text citation’s frequencies-based recommendations of relevant research papers
Source: PeerJ Comput Sci. 2021 Jun 4;7:e524. doi: 10.7717/peerj-cs.524 (PMC8189020; doi:10.7717/peerj-cs.524)
Supplement: Supplemental Information 2 — These articles are available under a CC BY-ND 4.0 license. [file peerj-cs-07-524-s002.zip › Dataset/Papers Dataset.zip!/Papers Dataset/jucs_118.pdf]

Journal of Universal Computer Science, vol. 11, no. 12 (2005), 2179-2191
submitted: 17/10/05, accepted: 16/11/05, appeared: 28/12/05 © J.UCS

Sequential Computability of a Function. Effective Fine Space and Limiting Recursion[1]

Mariko Yasugi[2]
(Faculty of Science, Kyoto Sangyo University, Kyoto, Japan
yasugi@cc.kyoto-su.ac.jp)

Yoshiki Tsujii
(Faculty of Science, Kyoto Sangyo University, Kyoto, Japan
tsujiiy@cc.kyoto-su.ac.jp)

Takakazu Mori
(Faculty of Science, Kyoto Sangyo University, Kyoto, Japan
morita@cc.kyoto-su.ac.jp)

Abstract: We consider real sequences in $I = [0, 1)$ and real functions on I. It is first shown that, as for real sequences from I, $\mathbf{R}$-computability (computability with respect to the Euclidean topology) implies "weak Fine-computability." Using this result, we show that "Fine-sequential computability" and "$\mathcal{L}^*$-sequential computability" are equivalent for effectively locally Fine-continuous functions as well as for Fine-continuous functions.

Key Words: Effective Fine Space, Weakly Fine-computable Sequence, Fine-sequential Computability of a Function, $\mathcal{L}^*$-sequential Computability of a Function, Effective Fine-continuous Function, Limiting Recursion
Category: F.0, G.0

1 Introduction

The standard notion of computability of a real number or of a sequence of real numbers as well as that of computability of a continuous function or of a sequence of continuous functions on the real line is generally agreed. As for a continuous real function f defined on a compact interval, for example, f is called computable if the two conditions below are satisfied [Pour-El, Richards89].

(i) (Sequential computability) Given a computable sequence $\{x_m\}$, $\{f(x_m)\}$ is a computable sequence of real numbers.

(ii) (Effective uniform continuity) There is a recursive function α with which holds that $|x - y| < \frac{1}{2^{\alpha(p)}}$ implies $|f(x) - f(y)| < \frac{1}{2^p}$.

This definition can easily be extended to a function which is continuous on the whole real line.

We would also like to attribute a certain kind of computability to some discontinuous functions. In that case, the conditions (i) and (ii) above have to be modified.

[1] C. S. Calude, H. Ishihara (eds.). *Constructivity, Computability, and Logic. A Collection of Papers in Honour of the 60th Birthday of Douglas Bridges.*

[2] This work has been supported in part by Kayamori Foundations of Informational Sciences Advancement K15VIII No.157 and Science Foundations of JSPS No.16340028.

There are many theories of computation of discontinuous functions. We too have proposed some approaches to this problem. Among them, one is to express the value of a function at a jump point in terms of a "limiting recursive" modulus of convergence instead of a recursive one [Yasugi et al.01]. Another is to change the topology of the domain of a function [Tsujii et al.01]. In some cases, these two approaches are equivalent [Yasugi, Tsujii02] [Tsujii et al.05]. As for a sequence of functions with varying jump points, we took up, as an example, the system of Rademacher functions $\{\phi_l\}$ [Yasugi, Washihara03]. In [Yasugi, Washihara03], it was claimed that $\{\phi_l\}$ admits a "weak computation" in the following sense: input a recursive information of a computable sequence of real numbers $\{x_m\}$, a recursive sequence of rational numbers $\{s_{lmn}\}$ converging to $\{\phi_l(x_m)\}$ with a "limiting recursive" modulus of convergence can be found. Such a property of a function (function sequence) has been called "$\mathcal{L}^*$-sequentially computable."

In [Tsujii et al.05] and [Yasugi et al.05], we presented an alternative way of expressing a notion of computability of a function sequence like the Rademacher function system by changing the topology of the real interval $I = [0, 1)$, first by decomposing it into $\{[\frac{k}{2^\nu}, \frac{k+1}{2^\nu})\}_k$ for each ν $(0 \le k \le 2^\nu - 1)$, and then taking a kind of the limit with respect to ν. In this way, we obtain an "effective uniform space" as the limit of an "effective sequence of uniform spaces." The computability of a sequence from I with respect to this limit space is called "diagonal-computatbility." It is based on our theory of the effective uniform space (cf. [Tsujii et al.01], [Yasugi et al.02], [Yasugi, Tsujii02], [Yasugi et al.05]).

On the other hand, Brattka [Brattka02] and Mori [Mori01] worked on the computability in the space of Fine metric. In fact, diagonal-computability and Fine-computability coincide.

In [Yasugi et al.05], it is shown that, input a Fine-computable sequence of real numbers, $\{x_m\}$, the double sequence of values $\{\phi_l(x_m)\}$ of Rademacher functions is $\mathbf{R}$-computable (computable in the Euclidean topology). Such a property of a function (function sequence) will be called "Fine-sequentially computable."

The two notions of sequential computability of a function (a function sequence) appear quite apart, but they can be related as in the case of [Tsujii et al.05] and [Yasugi, Tsujii02].

The *aim* of this article is to *relate these two notions of sequential computability*, one with respect to the *Fine metric* topology and one with respect to *limiting recursive functions*. The domain of discourse is restricted to $I = [0, 1)$.

We first define the notion of "weak Fine-computability" and "right computability" of a sequence of real numbers, and show their equivalence. It is also shown that an $\mathbf{R}$-computable sequence of real numbers is weak Fine-computable (Section 3: Theorem 1).

In Section 4, we introduce two notions of sequential computability of a function, "Fine-sequential computability" and "$\mathcal{L}^*$-sequential computability," citing from [Tsujii et al.05]. We state that $\mathcal{L}^*$-sequential computability implies Fine-sequential computability (without any condition): Theorem 2. For an effectively Fine-uniformly continuous function, the converse has been proved in [Tsujii et al.05] (stated as Theorem 3 here).

We then show that, for an "effectively locally Fine-uniformly continuous" function, Fine-sequential computability implies $\mathcal{L}^*$-sequential computability (Section 5: Theorem 4). The $\mathcal{L}^*$-sequential computability is defined here in terms of an auxiliary notion of "weak $\mathbf{R}$-representation" of a sequence of real

numbers.

Modifying the proof of Theorem 4, a stronger result can be derived, that is, for an "effectively Fine-continuous function," Fine-sequential computability implies $\mathcal{L}^*$-sequential computability (Section 6: Theorem 5).

Some examples of functions relevant to Sections 4∼6 are given in Section 7.

In Section 2, we list some definitions and notations from preceding references for the reader's convenience.

As a way of Appendix, we include a proof of the fact that the family of limiting recursive functions is closed under substitution for an "isolated" argument (Section 8). This fact is tacitly assumed in the proof of Theorem 5.

We cite only those references which have direct applications to the present work. We have consulted [Kawada, Mimura65] for the uniform space and [Schipp et al.90] for the Rademacher functions. As for some notions of computable functions on I, we have also consulted [Mori01] and [Mori02].

2 Preliminaries

For details of basic definitions below, see [Pour-El, Richards89] and [Tsujii et al.01]. In the following, **N** and **R** respectively denote the set of natural numbers and the set of real numbers.

A sequence of rational numbers $\{r_m\}$ is called *recursive* if there is a recursive way to compute r_m for each m. A real number x is called *computable* with respect to the Euclidean topology (**R**-*computable*) if it is approximated by a recursive sequence of rational numbers $\{r_m\}$ with a recursive modulus of convergence α, that is, $|x - r_m| < \frac{1}{2^p}$ for $m \geq \alpha(p)$. We will express such a circumstance as $x \simeq \langle r_m, \alpha(p)\rangle$, or for short, $x \simeq \langle r_m, \alpha\rangle$. These definitions can be extended to a *computable sequence* of real numbers.

We will henceforth confine the domain of discourse to the interval $I = [0, 1)$.

For $n = 0, 1, 2, \cdots$ and $0 \leq k \leq 2^n - 1$, define subintervals of I, $\{I_k^n\}$, as well as a sequence of maps $U_n : I \to P(I)$ as follows, where $P(I)$ denotes the powerset of I. Put

$$I_k^n = [\frac{k}{2^n}, \frac{k+1}{2^n})$$

for $0 \leq k \leq 2^n - 1$. Next define $U_n(x)$ as follows. Let k be the unique k such that $x \in I_k^n$, and define

$$U_n(x) = I_k^n.$$

$\{U_n\}$ forms an "effective uniformity" on I, hence $\mathcal{I} = \langle I, \{U_n\}\rangle$ is an effective uniform space (cf. [Tsujii et al.01]). It can easily been shown that it is topologically equivalent to the effective Fine-metric space, that is, $\{U_n\}$ is a system of fundamental neighborhoods for the Fine-metric topology.

Definition 2.1 (Fine-computable sequence of real numbers) A sequence of real numbers $\{x_m\} \subset I$ is called Fine-*computable* if there is a recursive sequence $\{q_{mp}\} \subset I$ of rational numbers which converges to $\{x_m\}$ effectively with respect to $\{U_n\}$ in a manner that, for a recursive function γ and for $l \geq \gamma(m, p)$, $q_{ml} \in U_p(x_m)$. We will write this property as $x_m \simeq_F \langle q_{ml}, \gamma\rangle$. The definition can be extended to multiple sequences. For example, $x_{nm} \simeq_F \langle q_{nml}, \gamma\rangle$ will express the property that, for $l \geq \gamma(n, m, p)$, $q_{nml} \in U_p(x_{nm})$.

A real number x is called Fine-computable if $\{x, x, x, \cdots\}$ is Fine-computable.

Corollary 1 1) A Fine-computable sequence is **R**-computable, but not conversely. For a single real number in I, it is **R**-computable if and only if it is Fine-computable (cf. [Brattka02], [Mori01],[Yasugi et al.05]).

2) The family of Fine-computable sequences of real numbers (in I), say $\mathcal{C}$, forms a "computability structure" for $\langle I, \{U_n\}\rangle$ (cf. [Tsujii et al.01], for example, for the computability structure of an effective uniform space). This computability is equivalent to "Fine-computability" in [Mori01].

Definition 2.2 (Effective Fine space) The triple $\langle I, \{U_n\}, \mathcal{C}\rangle$ will be called the *effective Fine space.*

Let $r, s \geq 0$ be integers and let g and $\phi_1, \cdots, \phi_r$ be recursive functions, where $g : \mathbf{N}^{r+s+1} \to \mathbf{N}$ and $\phi_i : \mathbf{N} \to \mathbf{N}, i = 1, 2, \cdots r$. The function h defined as follows will be called *limiting recursive* according to Gold [Gold65]:

$$h(p_1, \cdots, p_s) = \lim_n g(\tilde{\phi}_1(n), \cdots, \tilde{\phi}_r(n), p_1, \cdots, p_s, n),$$

where $\tilde{\phi}(n)$ is a code for the finite sequence

$$\langle \phi(0, p_1, \cdots, p_s), \cdots, \phi(n, p_1, \cdots, p_s)\rangle\,,$$

presuming that the limit exists.

Subsequently when we mention a sequence (of numbers or functions), it may be a multiple sequence. For example, a recursive sequence of rational numbers may mean a single sequence $\{r_j\}_j$ or a multiple sequence $\{r_{\mathbf{j}}\}_{\mathbf{j}}$ ($\mathbf{j} = j_1 j_2 \cdots j_n$), as the case may be.

3 R-computability and weak Fine-computability

We will henceforth work in the effective Fine space $\langle I, \{U_n\}, \mathcal{C}\rangle$ (cf. Definition 2.2).

We will first relate **R**-computability and "weak Fine-computability" of a real sequence.

The following lemma will be useful.

Lemma 1 A recursive sequence of rational numbers is Fine-computable.

Definition 3.1 (Weak Fine-computability) Let $\{x_m\}$ be a sequence from I. Suppose that there is a recursive sequence of rational numbers $\{z_{mp}\}$ such that $x_m < z_{mp}$ for all p, and that there is a limiting recursive function $\nu(m, n)$ such that, for all m, n, and for all $q \geq \nu(m, n)$, $z_{mq} \in U_n(x_m)$. Then we call $\{x_m\}$ *weakly* Fine-*computable*, and ν a *weak modulus of convergence* (of $\{z_{mq}\}$ to $\{x_m\}$). We express this property by

$$x_m \simeq_{wF} \langle z_{mp}, \nu\rangle\,,$$

and call any such pair $\langle z_{mp}, \nu\rangle$ a *weak Fine-representation* of $\{x_m\}$.

Corollary 2 1) A Fine-computable sequence is weakly Fine-computable with a "recursive" (weak) modulus of convergence.

2) Weak Fine-computability can be equivalently stated as follows. $\{x_m\}$ is weak Fine-computable if there is a recursive sequence of rationals, say $\{t_{mp}\}$, which is nonincreasing and $x_m \simeq_{wF} \langle z_{mp}, \nu \rangle$.

Proof of 2) Given $x_m \simeq_{wF} \langle z_{mp}, \nu \rangle$, define another recursive sequence of rational numbers $\{t_{mp}\}$, which is nonincreasing and converges to $\{x_m\}$ from above, as follows. $t_{m1} = z_{m1}$. $t_{m(l+1)} = t_{ml}$ if $z_{m(l+1)} > t_{ml}$, and $= z_{m(l+1)}$ otherwise. Then $\{t_{mp}\}$ is nonincreasing and Fine-approximates $\{x_m\}$ from above.

Define $\lambda(m, q)$ as follows. $\lambda(m, 1) = 1$. $\lambda(m, l+1) = \lambda(m, l)$ if $t_{m(l+1)} = t_{ml}$ and $= \lambda(m, l) + 1$ otherwise. Then $\nu_0(m, p) = \nu(m, \lambda(m, p))$ is limiting recursive and serves as a modulus of convergence of $\{t_{mp}\}$ to $\{x_m\}$.

Remark If $x_m \simeq_{wF} \langle z_{mp}, \nu \rangle$, then $0 < z_{mq} - x_m < \frac{1}{2^n}$ for all $q \geq \nu(m, n)$, especially

$$0 < z_{m\nu(m,n)} - x_m < \frac{1}{2^n}. \tag{1}$$

The definition below applies to sequences of real numbers which are not necessarily from I. It is the sequential version of an idea by Zheng and Weihrauch.

Definition 3.2 (Right computability: cf. [Zheng, Weihrauch00]) A sequence of real numbers $\{x_m\}$ is called *right computable* if there is a recursive sequence of rational numbers, say $\{r_{mp}\}$, which converges (classically) to $\{x_m\}$ from above.

Corollary 3 A sequence of real numbers $\{x_m\}$ can be equivalently called right computable if there is a recursive sequence of rational numbers which is nonincreasing and converges (classically) to $\{x_m\}$ from above.

The proof is the same as the first part of the proof of 2), Corollary 2.

Proposition 3.1 (From right computability to weak Fine-computability) Suppose $\{x_m\} \subset I$ is right computable, that is, (by virtue of Corollary 3) there is a recursive, nonincreasing sequence of rational numbers $\{t_{ml}\} \subset I$ which (classically) converges to $\{x_m\}$. Then $\{x_m\}$ is weakly Fine-computable.

Proof Define a recursive function $\kappa(m, n, l)$ by $\kappa(m, n, l) =$ *the* k such that $t_{ml} \in I^n_k = [\frac{k}{2^n}, \frac{k}{2^n} + \frac{1}{2^n})$. Notice that $\kappa(m, n, l) \leq 2^n - 1$, and $\{\kappa(m, n, l)\}_l$ is nonincreasing. $\kappa(m, n, l)$ will be eventually constant, and, with its value $k_{mn} = \lim_l \kappa(m, n, l)$, $x_m \in I^n_{k_{mn}}$ holds.

Define next a recursive function $\nu(m, n, l)$ as follows.

$$\nu(m, n, 1) = 1;$$

$$\begin{aligned} \nu(m, n, l+1) &= \nu(m, n, l) \quad \text{if} \quad \kappa(m, n, l+1) = \kappa(m, n, l); \\ &= l + 1 \quad \text{if} \quad \kappa(m, n, l+1) < \kappa(m, n, l). \end{aligned}$$

Define

$$\nu(m, n) = \lim_l \nu(m, n, l).$$

The limit exists and $\nu(m, n)$ is limiting recursive. Further, $k_{mn} = \kappa(m, n, \nu(m, n))$ holds. If $l \geq \nu(m, n)$, then $t_{ml} \in I^n_{\kappa(m,n,\nu(m,n))}$, and hence $t_{ml} \in U_n(x_m)$. This means that $\{x_m\}$ is weakly Fine-computable, that is, $x_m \simeq_{wF} \langle t_{ml}, \nu \rangle$.

Proposition 3.2 (From weak Fine-computability to right computability) If $\{x_m\}$ is weakly Fine-computable, then it is right computable.

Proof Suppose $\{x_m\}$ is weakly Fine-computable with $x_m \simeq_{wF} \langle z_{mq}, \nu\rangle$. Then, by definition, $\{z_{mp}\}$ converges to $\{x_m\}$ from above.

Proposition 3.3 (From **R**-computability to weak Fine-computability) If $\{x_m\}$ is **R**-computable, then it is weakly Fine-computable.

Proof Suppose $x_m \simeq \langle r_{mp}, \alpha\rangle$. Define $z_{mp} = r_{m\alpha(m,p)} + \frac{1}{2^p}$. Then $\{x_m\}$ is right computable by $\{z_{mp}\}$. So, by Proposition 3.1, $\{x_m\}$ is weakly Fine-computable.

Note The notion of "weak Fine-computability" is slightly different from the corresponding notion of weak $\mathcal{D}$-computability in [Tsujii et al.05], although in effect the present definition of weak Fine-computability is treated there. A direct proof of Proposition 3.3 is given in the proof of Theorem 3.3 of [Tsujii et al.05].

Summing up, we have the following.

Theorem 1 (Notions of computability) In I, the weak Fine-computable sequences of real numbers and the right computable ones coincide, and the **R**-computable real sequences form a subset of either of them. In fact, the inclusion is a strict one, since it is known that there is a real number x which is right computable but is not **R**-computable.

4 Sequential computability of a function

In [Tsujii et al.05], we defined two notions of sequential computability of a real function on I, one with respect to the Euclidean topology and one with respect to $\{U_n\}$, and then related them for "effectively $\{U_n\}$-uniformly continuous functions." We will cite the definitions of these notions by replacing $\{U_n\}$ by "Fine" and with slight modification (cf. Section 4 of [Tsujii et al.05]).

We first define an auxiliary notion of "weak **R**-representaion" of a sequence of real numbers.

Definition 4.1 (Weak **R**-representation) Let $\{y_m\}$ be a sequence of real numbers. If $\{y_m\}$ is approximated by a recursive sequence of rational numbers, say $\{t_{mq}\}$, with a modulus of limiting recursive convergence, say δ, then we will say that $\{y_m\}$ has a *weak* **R**-*representation*, and such a relation will be expressed as $y_m \simeq_{wR} \langle t_{mq}, \delta\rangle$.

Definition 4.2 (Sequential computability of a function) Let $f : I \to \mathbf{R}$ be a real function defined on I.

1) f is called Fine-*sequentially computable* if, for every Fine-computable sequence of real numbers $\{x_m\} \subset I$, $\{f(x_m)\}$ is **R**-computable.
2) f is called $\mathcal{L}^*$-*sequentially computable* if, for any **R**-computable sequence $\{x_m\} \subset I$, $\{f(x_m)\}$ has a weak **R**-representation, say $f(x_m) \simeq_{wR} \langle t_{mq}, \delta\rangle$ (cf. Definition 4.1), where δ is recursive in ν, presuming that $x_m \simeq_{wF} \langle z_{mp}, \nu\rangle$ (cf. Definition 3.1 and Proposition 3.3).

Note 1) $\mathcal{L}^*$-sequential computability is stated slightly differently from the corresponding notion in [Yasugi, Tsujii02], although the two definitions are essentially the same.

2) It is not even required that $f(x_m)$ is $\mathbf{R}$-computable for each m. Even if we required it, the subsequent argument needs not be changed.

3) The Fine-sequential computability of a function in 1) of Definition 4.2 is the same as the usual definition of sequential computability in a metric space.

Remark Fine-sequential computability of a function f on I means that the sequence of values preserves computability only for Fine-computable sequences (of real numbers), while $\mathcal{L}^*$-sequential computability means that for any input of an $\mathbf{R}$-computable sequence, the output can only be claimed to have a weak $\mathbf{R}$-representation. Notwithstanding that these two notions differ in nature, it can be shown that the two notions of sequential computability in Definition 4.2 coincide for functions satisfying a certain effective continuity.

One direction is immediate: we will reproduce the statement and the proof from [Tsujii et al.05], modified to the language in this article.

Theorem 2 (From $\mathcal{L}^*$-sequential computability to Fine-sequential computability: Theorem 4.2 in [Tsujii et al.05]) If f is $\mathcal{L}^*$-sequentially computable, then f is Fine-sequentially computable.

Proof Let $\{x_m\} \subset I$ be Fine-computable. Then, it is weakly Fine-computable with a recursive modulus of convergence ν (Corollary 2). Suppose f is $\mathcal{L}^*$-sequentially computable. Since $\{x_m\}$ is $\mathbf{R}$-computable, $\{f(x_m)\}$ has a weak $\mathbf{R}$-representation, say $f(x_m) \simeq_{wR} \langle t_{mq}, \delta \rangle$, where δ is recursive in ν. As above, ν is in fact recursive, and hence δ can be recursive, and so $\{f(x_m)\}$ is $\mathbf{R}$-computable.

Notice that no particular condition (of continuity) is imposed on f in this direction.

We have considered in [Tsujii et al.05] the family of functions which are "effectively Fine-uniformly continuous." A function in this family is, in the Euclidean topology, not necessarily continuous. In fact, it is not even necessarily piecewise continuous. (An example of non-piecewise continuous but is effectively Fine-uniformly continuous function will be given in Section 7.) Of these functions, the following has been shown in [Tsujii et al.05].

Theorem 3 (Fine-uniformly continuous case: [Tsujii et al.05]) If f is an "effectively Fine-uniformly continuous" and "Fine-sequentially computable" function, then f is $\mathcal{L}^*$-sequentially computable (cf. Definition 4.3 and Theorem 4.4 in [Tsujii et al.05] for details).

Some examples are seen in [Tsujii et al.05]. The result also holds for a sequence of Fine-uniformly continuous functions such as the Rademacher function system.

5 Locally Fine-uniformly continuous function

Our primary objective in this article is to extend Theorem 3 to locally Fine-uniformly continuous functions as well as to Fine-continuous functions.

Let $\{e_i\}$ be a recursive enumeration of dyadic rational numbers. We will fix such an enumeration.

Note The subsequent argument goes through for any such enumeration.

Definition 5.1 (Effective local Fine-uniform continuity) A function $f : I \to \mathbf{R}$ is called *effectively locally Fine-uniformly continuous*, if there are recursive functions γ and α such that

$$\cup_{i=1}^{\infty} U_{\gamma(i)}(e_i) = I; x, y \in U_{\gamma(i)}(e_i) \wedge y \in U_{\alpha(i,p)}(x) \to |f(x) - f(y)| < \frac{1}{2^p}. \quad (2)$$

Theorem 4 (From Fine-sequential computability to $\mathcal{L}^*$-sequential computability: locally Fine-uniformly continuous function) Let f be an effectively locally Fine-uniformly continuous function on I. If f is Fine-sequentially computable, then it is $\mathcal{L}^*$-sequentially computable.

Proof Recall that, for any real number in I, say r, $U_n(r)$ denotes the fundamental neighborhood of r of size n, say $[e, e + \frac{1}{2^n})$. Since f is effectively locally Fine-uniformly continuous, there are recursive γ and α as in (2).

Suppose $\{x_m\}$ is an $\mathbf{R}$-computable sequence. Then, by Proposition 3.3, there are a recursive sequence of rational numbers $\{z_{mp}\}$ and a limiting recursive function ν so that

$$q \geq \nu(m, n) \to z_{mq} \in U_n(x_m). \quad (3)$$

Since $\{z_{mp}\}$ is a recursive sequence of rational numbers, it is Fine-computable by Lemma 1, and hence, if f is Fine-sequentially computable, then there are a recursive sequence of rational numbers $\{s_{mql}\}$ and a recursive function β so that $f(z_{mp}) \simeq \langle s_{mql}, \beta \rangle$, or

$$l \geq \beta(m, q, n) \to |f(z_{mq}) - s_{mql}| < \frac{1}{2^n}. \quad (4)$$

Define a recursive sequence of rational numbers $\{t_{mn}\}$ by

$$t_{mn} = s_{mn\beta(m,n,n)}. \quad (5)$$

Suppose $x_m \in U_{\gamma(i)}(e_i)$. For the time being, we assume that $i (= i_m)$ can be computed from m. (For the simplicity of notation, we will simply write i instead of i_m in the subsequent proof.) Putting $n = \gamma(i)$ in (3), we have

$$q \geq \nu(m, \gamma(i)) \to z_{mq} \in U_{\gamma(i)}(x_m) = U_{\gamma(i)}(e_i). \quad (6)$$

Putting $n = \alpha(i, p + 1)$ in (3), we obtain

$$q \geq \nu(m, \alpha(i, p + 1)) \to z_{mq} \in U_{\alpha(i,p+1)}(x_m). \quad (7)$$

If we put $x = x_m$ and $y = z_{mq}$ in (2), and if we take $p + 1$ instead of p, then by (6) and (7), we obtain

$$q \geq \nu(m, \gamma(i)), \nu(m, \alpha(i, p + 1)) \to |f(z_{mq}) - f(x_m)| < \frac{1}{2^{p+1}}. \quad (8)$$

Now define a function δ as follows.

$$\delta(m,p) = \max(\nu(m,\gamma(i)), \nu(m,\alpha(i,p+1)), p+1). \tag{9}$$

Suppose $q \geq \delta(m,p)$. Then by (8), (5) and (4), we have

$$|f(x_m) - t_{mq}| \leq |f(x_m) - f(z_{mp})| + |f(z_{mp}) - t_{mq}|$$

$$< \frac{1}{2^{p+1}} + |f(z_{mp}) - s_{mq\beta(m,q,q)}| < \frac{1}{2^{p+1}} + \frac{1}{2^q} \leq \frac{1}{2^{p+1}} + \frac{1}{2^{p+1}} = \frac{1}{2^p}.$$

Summing up,

$$q \geq \delta(m,p) \to |f(x_m) - t_{mq}| < \frac{1}{2^p}. \tag{10}$$

If we can show that $i = i_m = \iota(m)$ is limiting recursive in ν, then $\delta(m,p)$ is recursive in ν. (See Note below.) So, $\{f(x_m)\}$ has a weak **R**-representaion, $\langle t_{mq}, \delta\rangle$, where δ is recursive in ν, from the equation (9).

By the first condition of (2), for each m, there is an i such that $x_m \in U_{\gamma(i)}(e_i)$, or $U_{\gamma(i)}(e_i) = U_{\gamma(i)}(x_m)$. To find such an i, check if

$$z_{m,\nu(m,\gamma(i))} \in U_{\gamma(i)}(e_i)$$

holds for $i = 1, 2, 3, \cdots$ successively. (There is such an i, and the process of finding i is effective in ν.) Once such an i is hit, put $\iota(m) = i_m = i$. ι is recursive in ν. It holds, for such i,

$$U_{\gamma(i)}(e_i) = U_{\gamma(i)}(z_{m\nu(m,\gamma(i))}).$$

Since $z_{m\nu(m,\gamma(i))} \in U_{\gamma(i)}(x_m)$,

$$U_{\gamma(i)}(x_m) = U_{\gamma(i)}(z_{m\nu(m,\gamma(i))}),$$

and hence $U_{\gamma(i)}(e_i) = U_{\gamma(i)}(x_m)$. So, the ι as above will do.

Note To claim that δ is recursive in ν from (9) when i_m is recursive in ν, we need to know that the operation of substituting a limiting recursive function for a variable of another one can be reduced to a single limit ("merger of limits"). In fact it is a known fact (cf. [Nakata, Hayashi01], for example), but we will present a proof in Section 7 for the reader's convenience.

It is now reasonable to define a function to be "locally Fine-uniformly computable" as follows, which is the same as the definition in existing references (cf. [Brattka02], [Mori01],[Mori02],[Mori et al.05]). Namely, a function $f : I \to \mathbf{R}$ is called *locally Fine-uniformly computable* if it is effectively locally Fine-uniformly continuous, and furthermore it is Fine-sequentially computable. From Theorems 2 and 4, the last condition can be replaced by "$\mathcal{L}^*$-sequentially computable."

6 Fine-continuous function

We will next deal with functions of yet weaker continuity.

Definition 6.1 (Effective Fine-continuity) A function $f : I \to \mathbf{R}$ is called *effectively Fine-continuous* if there is a recursive function γ such that

$$\cup_{i=1}^{\infty} U_{\gamma(i,p)}(e_i) = I; x \in U_{\gamma(i,p)}(e_i) \to |f(x) - f(e_i)| < \frac{1}{2^p}. \tag{11}$$

Theorem 5 (From Fine-sequential computability to $\mathcal{L}^*$-sequential computability: Fine-continuous function) Let f be an effectively Fine-continuous function on I. If f is Fine-sequentially computable, then it is $\mathcal{L}^*$- sequentially computable.

Note Since an effectively locally Fine-uniformly continuous function is effectively Fine-continuous, Theorem 4 is a special case of Theorem 5. For the proof of Theorem 5, part of the proof of Theorem 4 can be adopted.

Proof of Theorem 5 By the assumption, the conditions in (11) hold for f.

Suppose $\{x_m\}$ is an $\mathbf{R}$-computable sequence from I. We will use (3,4,5) in the proof of Theorem 4.

Due to (11), for each m and for each p, there is an $i(= i_{mp})$ such that $x_m \in U_{\gamma(i,p+2)}(e_i)$. (For the simplicity of notation, we will write simply i instead of i_m.) Then $U_{\gamma(i,p+2)}(x_m) = U_{\gamma(i,p+2)}(e_i)$. As in the proof of Theorem 4, such an i can be found recursive in ν. Then put $n = \gamma(i, p+2)$ in (3) to obtain

$$q \geq \nu(m, \gamma(i, p+2)) \to z_{mq} \in U_{\gamma(i,p+2)}(x_m) = U_{\gamma(i,p+2)}(e_i). \tag{12}$$

From (12) and (11) with $x = z_{mq}$ and $p+2$ in the place of p, we have

$$q \geq \nu(m, \gamma(i, p+2)) \to |f(z_{mq}) - f(e_i)| < \frac{1}{2^{p+2}}. \tag{13}$$

From (11) with $x = x_m$ and $p+2$ follows

$$|f(x_m) - f(e_i)| < \frac{1}{2^{p+2}}. \tag{14}$$

From (4) with $l = \beta(m, q, q)$, we have

$$|f(z_{mq}) - s_{mq\beta(m,q,q)}| < \frac{1}{2^q}. \tag{15}$$

Define

$$\delta(m,p) := \max(\nu(m, \gamma(i, p+2)), p+2). \tag{16}$$

Assume $q \geq \delta(m,p)$. Then $q \geq \nu(m, \gamma(i, p+2)), p+2$. So we have, from (14), (13) and (15),

$$|f(x_m) - t_{mq}| \leq |f(x_m) - f(e_i)| + |f(e_i) - f(z_{mq})| + |f(z_{mq}) - t_{mq}|$$
$$< \frac{1}{2^{p+2}} + \frac{1}{2^{p+2}} + \frac{1}{2^q} \leq \frac{3}{2^{p+2}} < \frac{1}{2^p}.$$

So, $\{f(x_m)\}$ is approximated by a recursive sequence of rational numbers $\{t_{mq}\}$ with a modulus of convergence $\delta(m,p)$, which is recursive in ν and i, where ν is limiting recursive.

Now, $i = \iota(m,p)$ can be defined in a manner similar to $\iota(m)$ in the previous section with p added as another argument.

Similarly to the previous case, it is reasonable to define "Fine-computable function " as follows, which is the same as the definition in existing references (cf. [Brattka02], [Mori01], [Mori02], [Mori et al.05]). Namely, a function $f : I \to \mathbf{R}$ is called *Fine-computable* if it is effectively Fine-continuous, and furthermore it is Fine-sequentially computable. As before, the last condition can be replaced by "$\mathcal{L}^*$-sequentially computable."

7 Some examples

Let us first give an example of a Fine-uniformly computable function of interesting nature, taken from Example 5.1 in [Mori et al.05].

Define first a function on I:

$$\tilde{\chi_c}(x) = \chi_{[0,c)}(x),$$

where $\chi_A(x)$ is the characteristic function of the set A. Using $\tilde{\chi_c}(x)$, define next

$$f_n(x) = \sum_{i=1}^{n} 2^{-i}\tilde{\chi}_{e_i}(x) \quad \text{and} \quad f(x) = \sum_{i=1}^{\infty} 2^{-i}\tilde{\chi}_{e_i}(x).$$

According to [Mori et al.05], $\{f_n\}$ is a "uniformly Fine-computable" sequence of functions and "Fine-converges effectively uniformly" to f. So, f is Fine-uniformly computable (uniformly Fine-computable in [Mori et al.05]) by Theorem 1 in [Mori et al.05]. While it is continuous at every irrational number, it is discontinuous at every dyadic rational with respect to the Euclidean metric, since $f(x) - f(e_i) > 2^{-i}$ for any $x < e_i$. So, f is, though Fine-uniformly continuous, not even piecewise continuous in the Euclidean topology.

There are many functions on I which are not Fine-uniformly continuous, that is, not uniformly continuous in the space $\langle I, \{U_n\}\rangle$, but are locally Fine-uniformly continuous or Fine-continuous. They are listed in [Tsujii et al.05], but we reproduce them here.

The following are some examples of locally uniformly Fine-computable functions which are not uniformly Fine-continuous.

Pick up disjoint intervals $\{I^n_{2^n-2}\}_{n=1,2,\ldots}$, where $I^n_{2^n-2} = [\frac{2^n-2}{2^n}, \frac{2^n-1}{2^n})$. Then it holds that $\cup_{n=1}^{\infty} I^n_{2^n-2} = [0,1) = I$. Define a function by $\phi(x) = n$ if $x \in I^n_{2^n-2}$. ϕ is not Fine-uniformly continuous, but it is locally Fine-uniformly computable. $\psi(x) = n + x, x \in I^n_{2^n-2}$ is also an example of this sort. Another such example is the following function: $\mu(x) = \frac{1}{1-2x}$ if $0 \le x < \frac{1}{2}$ and $\mu(x) = 1$ if $\frac{1}{2} \le x < 1$. These functions are locally Fine-uniformly computable.

An example of a Fine-computable function which is not locally Fine-uniformly continuous has been constructed by Brattka in Theorem 12 of [Brattka02]. It has been generalized in [Mori et al.05].

Note In [Tsujii et al.01], notions of "computability" and "uniform computability" have been defined for the effective uniform space. Fine-computability satisfies the condition of "computability" in [Tsujii et al.01], and Fine-uniform computability satisfies the condition of "uniform computability" in [Tsujii et al.01].

References

[Brattka02] Brattka,V., *Some notes on Fine computability*, JUCS, 8(3) (2002),382-395.

[Gold65] Gold, E.M., *Limiting recursion*, JSL, 30-1 (1965), 28-48.

[Kawada, Mimura65] Kawada, Y., Mimura, Y., *Outline of Modern Mathematics* (in Japanese), Iwanami Shoten (1965).

[Mori01] Mori, T., *Computabilities of Fine continuous functions*, Computability and Complexity in Analysis, LNCS 2064, Springer (2001) 200-221.

[Mori02] Mori, T., *On the computability of Walsh functions*, TCS 284 (2002) 419-436.

[Mori et al.05] Mori, T., Tsujii, Y., Yasugi, M., *Fine-computable functions and Effective Fine-convergence*, Proceedings of CCA2005, available at: http://www.kyoto-su.ac.jp/~yasugi/page/recent.html

[Nakata, Hayashi01] Nakata, M., Hayashi, S., *A limiting first order realizability interpretation*, SCMJ Online 5(2001), 421-434.

[Pour-El, Richards89] Pour-El, M.B., Richards, J.I., *Computability in Analysis and Physics*, Springer-Verlag (1989).

[Schipp et al.90] Schipp, F., Wade, W.R., Simon, P., *Walsh Series*, Adam Hilger (1990).

[Tsujii et al.01] Tsujii, Y., Yasugi, M., Mori, T., *Some properties of the effectively uniform topological space*, Computability and Complexity in Analysis, LNCS 2064, Springer (2001) 336-356.

[Tsujii et al.05] Tsujii, Y., Yasugi, M., Mori, T., *Sequential computability of a function -diagonal space and limiting recursion-*, ENTCS vol.120, 187-199, 2005.

[Yasugi et al.01] Yasugi, M., Brattka, V., Washihara, M., *Computability aspects of some discontinuous functions*, Scientiae Mathematicae Japonicae (SCMJ) Online, vol.5 (2001) 405-419.

[Yasugi et al.05] Yasugi, M., Mori, T., Tsujii, Y., *Effective sequence of uniformities and its effective limit*, Proceedings of CCA2005, available at: http://www.kyoto-su.ac.jp/~yasugi/page/recent.html

[Yasugi, Tsujii02] Yasugi, M., Tsujii, Y., *Two notions of sequential computability of a function with jumps*, ENTCS 66 No.1 (2002) 11 pages available at: http://www.elsevier.nl/locate/entcs/volume66.html.
Revised version: *Computability of a function with jumps -Effective uniformity and limiting recursion-*, Topology and its Applications(Elsevier Science), 146-147(2005), 563-582.

[Yasugi et al.02] Yasugi, M., Tsujii, Y., Mori, T., *Metrization of the uniform space and effective convergence*, Math. Log. Quart. 48 (2002) Suppl.1, 123-130.

[Yasugi, Washihara03] Yasugi, Y., Washihara, M., *A note on Rademacher functions and computability*, Words, Languages and Combinatorics III, World Scientific (2003) 466-475.

[Zheng, Weihrauch00] Zheng, X., Weihrauch, K., *Weakly computable real numbers*, Journal of Complexity 16 (2000), 676-690.

Appendix: Merger of limits

Here we show the property of "merger of limits" as announced in the previous section.

Theorem 6 (Merger of limits) Let $f(y,t,x)$ and $g(s,x)$ be recursive functions. Suppose $\lim_t f(y,t,x)$ and $\lim_s g(s,x)$ exist. Then

$$\lim_t f(\lim_s g(s,x),t,x) = \lim_t f(g(t,x),t,x). \tag{17}$$

Note We say that the substituted term $\lim_s g(s,x)$ is "isolated" (in the left side of (17)) due to the fact that it does not contain the "bound variable" t of another limit.

Proof

$$\lim_t f(\lim_s g(s,x),t,x) = a \leftrightarrow \exists t_1 \forall t \geq t_1.f(\lim_s g(s,x),t,x) = a, \tag{18}$$

and

$$\lim_t f(g(t,x),t,x) = a \leftrightarrow \exists t_0 \forall t \geq t_0.f(g(t,x),t,x) = a. \tag{19}$$

We show that the right hand sides of (18) and (19) are equivalent.

Note first that

$$\lim_s g(s,x) = b \leftrightarrow \exists s_1 \forall s \geq s_1.g(s,x) = b.$$

Suppose $\lim_s g(s,x) = b$. Then the right hand side of (18) can be expressed as follows.

$$\exists s_1 \forall s \geq s_1.g(s,x) = b \wedge \exists t_1 \forall t \geq t_1.f(b,t,x) = a. \tag{20}$$

This is equivalent to the following.

$$\exists s_1 \forall s \geq s_1 \exists t_1 \forall t \geq t_1.g(s,x) = b \wedge f(b,t,x) = a. \tag{21}$$

Taking $t_0 = \max(s_1, t_1)$, (21) implies

$$\exists t_0 \forall t \geq t_0.g(t,x) = b \wedge f(b,t,x) = a,$$

hence

$$\exists t_0 \forall t \geq t_0.f(g(t,x),t,x) = a.$$

This shows that the right hand side of (18) implies that of (19).

For the converse, take in particular $t \geq t_0, s_1$ (cf. (19) and 20). Then $g(t,x) = b$. So, putting $t_1 = \max(t_0, s_1)$, we obtain from (19)

$$\exists t_1 \forall t \geq t_1.f(b,t,x) = a,$$

or

$$\exists t_1 \forall t \geq t_1.f(\lim_s g(s,x),t,x) = a,$$

which is the right hand side of (18).
