# Supplementary material for: In-text citation’s frequencies-based recommendations of relevant research papers
Source: PeerJ Comput Sci. 2021 Jun 4;7:e524. doi: 10.7717/peerj-cs.524 (PMC8189020; doi:10.7717/peerj-cs.524)
Supplement: Supplemental Information 2 — These articles are available under a CC BY-ND 4.0 license. [file peerj-cs-07-524-s002.zip › Dataset/Papers Dataset.zip!/Papers Dataset/jucs_218.pdf]

Journal of Universal Computer Science, vol. 10, no. 9 (2004), 1035-1064
submitted: 15/12/03, accepted: 18/7/04, appeared: 28/9/04 *© J.UCS*

Incremental Maintenance of Data Warehouses Based on Past Temporal Logic Operators

Sandra de Amo
(Universidade Federal de Uberlândia, Brazil
deamo@ufu.br)

Mírian Halfeld Ferrari Alves
(Université François Rabelais - LI/Antenne de Blois, France
mirian@univ-tours.fr)

Abstract: We see a temporal data warehouse as a set of temporal views defined in the past fragment of the temporal relational algebra extended with set-valued attributes and aggregation. This paper proposes an incremental maintenance method for temporal views that allows improvements over the re-computation from scratch. We introduce a formalism for temporal data warehouse specification that summarizes information needed for its incremental maintenance. According to this formalism, a temporal data warehouse **W** is a pair of two sets of views: the *materialized component* and the *virtual component*. The materialized component of **W** represents the set of views physically stored in the warehouse. The virtual component of **W** is a set of non-temporal expressions involving only relations kept in the materialized component. Several features of our approach make it especially attractive as a maintenance method for warehouses: (a) there is no need for storing the entire history of source databases, (b) maintenance of the temporal data warehouse is reduced to maintaining the (non-temporal) materialized component, and (c) the materialized component is self-maintainable. We build a uniform algorithm by combining two previously unrelated techniques based on auxiliary views. Our method is sufficiently general so that it can be easily adapted to treating databases with complex-valued attributes.

Key Words: Temporal data warehouse, temporal databases, temporal relational algebra, temporal logic, self-maintenance.

Category: F.4.1, H.2, H.2.3, H.2.5

1 Introduction

A warehouse integrates disparate data sources to provide consolidate, summary data that can be used by online analytical processing (OLAP) and data mining tools. This aggregated data speeds up the query processing and facilitates high-level analysis, helping managers in their day-to-day decisions. In this paper, we consider the maintenance of a data warehouse composed of a set of materialized temporal views. Each temporal view originates from multiple, autonomous, heterogeneous and non-temporal sources. When an update is performed over a source relation, temporal views in the warehouse should change to reflect this update. The question arises whether the materialized views should be recomputed from scratch after each source update or whether they should be obtained from the old materialized views. Considering both approaches, one notices that:

• In a warehouse environment the cost of recomputing views from scratch is high since it involves consulting different sites, and thus implies a large overhead in terms of communication cost. In a temporal warehouse this approach requires keeping all the database history. As sources are usually non-temporal, this solution implies the storage of the history of all sources in the warehouse.
• The computation of new views from old ones usually requires querying source data (to obtain extra information). This computation may be expensive. To avoid this process, some methods allowing data warehouse self-maintenance have been introduced [Laurent et al. 2001, Mohania and Kambayashi 2000, Quass et al. 1996].

A naïve solution to the problem of maintaining materialized temporal views requires storing all sources (and their history) in the warehouse. This solution implies redundancies as illustrated by the next example.

Example 1.1 We consider a source database containing relations $UNIV$ and EMP. The relation $UNIV[C, N]$ contains the name *N* of each student admitted in a course *C* at the university. The relation $EMP[N, J]$ contains people's names *N* and their current job *J*. We suppose a data warehouse with just one temporal view V, specified by the following temporal relational algebra expression where $\blacksquare$ means *always in the past* [Clifford and Tuzhilin 1990]:

$$V = \blacksquare \Pi_N(UNIV \bowtie EMP) \tag{1}$$

This view gives the name of people who throughout the past but not necessarily at present have worked and studied at the same time. We call these people the *working students*. In other words, when the expression (1) is evaluated at instant i, it returns a relation containing all tuples $\langle b \rangle$ for which, for all instant k in the past ($k < i$), the tuple $\langle a, b \rangle$ appears in $UNIV$ and the tuple $\langle b, c \rangle$ appears in EMP.
Now, consider our source database evolving in time. At each instant $i \in \{0, 1, 2, 3\}$, the database instance is δ^i. Note that an instance δ^i changes into instance δ^{i+1} due to updates (seen as disjoint sets of insertions and deletions that can be performed in a unique transaction).
Remark: We assume that transactions ensure database consistency w.r.t. integrity constraints.

	$UNIV$
δ^0	$\{\langle cs, manuel\rangle, \langle math, jane\rangle, \langle law, john\rangle\}$
δ^1	$\{\langle cs, manuel\rangle, \langle math, mary\rangle, \langle law, john\rangle\}$
δ^2	$\{\langle cs, manuel\rangle, \langle cs, john\rangle, \langle math, paul\rangle\}$
δ^3	$\{\langle cs, john\rangle, \langle math, paul\rangle\}$

	EMP
δ^0	$\{\langle manuel, teacher\rangle, \langle john, waiter\rangle, \langle mary, nurse\rangle\}$
δ^1	$\{\langle john, waiter\rangle, \langle jane, bookseller\rangle\}$
δ^2	$\{\langle john, waiter\rangle, \langle jane, bookseller\rangle, \langle paul, clerk\rangle\}$
δ^3	$\{\langle john, waiter\rangle, \langle jane, bookseller\rangle\}$

The evaluation of expression (1) at $i = 3$ requires the following computation: $\Pi_N(\delta^0(UNIV) \bowtie \delta^0(EMP)) \cap \Pi_N(\delta^1(UNIV) \bowtie \delta^1(EMP)) \cap \Pi_N(\delta^2(UNIV) \bowtie \delta^2(EMP))$. In our case, we have $\delta^3(V) = \{\langle john\rangle\}$. Thus, considering that all the database history is stored in the warehouse, the computation from scratch of V at instant i requires the execution of i join operations. However, the resulting view contains just the tuples appearing in all these joins. For instance, in our case, the computation of $\Pi_N(\delta^0(UNIV) \bowtie \delta^0(EMP))$ gives $\{\langle manuel\rangle, \langle john\rangle\}$ but $\langle manuel\rangle$ is eliminated from the final result by the intersection operation. In fact, at instant $i = 1$

manuel stops working. This means that at instant $i = 2$ we know that *manuel* cannot be considered as a working student (as defined by expression (1)).
In real databases each join usually involves a large number of tuples. The cost of evaluating V can be very high, affecting the efficiency of the warehouse maintenance. Considering our example, it is easy to imagine the following situation. During the first year at a university, the majority of students work. Then due to a scholarship program, this number decreases considerably. In this case, the number of working students decreases but the evaluation of expression (1), at each instant i, still involves a lot of computation: indeed, the view V at instant i is computed by evaluating the expression $\Pi_N(\delta^0(UNIV) \bowtie \delta^0(EMP)) \cap \Pi_N(\delta^1(UNIV) \bowtie \delta^1(EMP))$ $\cap\ \Pi_N(\delta^2(UNIV) \bowtie \delta^2(EMP)) \cap \ldots ..\cap\ \Pi_N(\delta^{i-1}(UNIV) \bowtie \delta^{i-1}(EMP))$. Instead of computing V from scratch, it would be interesting to find V at instant i by using the instance of V at $i-1$ and some auxiliary information kept in the warehouse. □

As shown in Example 1.1, storing the entire history of sources in the warehouse may be time and space consuming. The aim of this work is to perform the incremental maintenance of a temporal warehouse without querying source data and keeping just a fraction of historical information in the warehouse.

The main contributions of the paper are:

1. An incremental maintenance method for temporal data warehouses that allows improvements over the re-computation of temporal views from scratch. After an update on the sources, the warehouse maintenance is performed by using only the updates on sources together with the old instance of the view and some auxiliary views stored in the warehouse. Not the entire history of source databases is stored in the warehouse.
2. Given a warehouse specification in terms of a set of views defined in the past fragment of the temporal relational algebra (extended with set-valued attributes and aggregation), we introduce a (formal) definition of a warehouse in terms of a materialized and a virtual component. The materialized component contains a set of materialized relational views and the virtual component implements the warehouse specification as a set of non-temporal views defined in terms of the materialized component. Importantly, in our approach (i) maintenance of the temporal data warehouse is reduced to maintaining the (non-temporal) materialized component, and (ii) the materialized component is self-maintainable.

Organization of the paper: Section 2 gives an overview of our approach. Section 3 introduces our temporal data model and describes the syntax and semantics of the temporal algebra EPTA (used to specify temporal views). This algebra is equivalent to temporal logic TL extended with operators to deal with aggregates and set-valued attributes. It generalizes the temporal algebra introduced in [Clifford and Tuzhilin 1990]. Section 4 shows how the materialized and virtual components of our temporal data warehouse are built. Section 5 shows our algorithm for maintaining a temporal data warehouse. Section 6 discusses some related work and Section 7 concludes the paper.

2 General Overview

To outline our approach, we consider its two main features, namely, (i) how to avoid the storage of the entire history of source databases in the warehouse and (ii) how to perform changes without consulting source relations.

2.1 Temporal data warehouses over non-temporal sources

We suppose a set of non-temporal local data sources and a data warehouse containing temporal views over these sources. The evaluation of the temporal views takes into account the history of the local data. As seen in Example 1.1, computing temporal views from scratch can be time and space consuming. In this work we propose an incremental method for maintaining temporal warehouses. To this end, we store auxiliary relations containing only a fraction of source history. After a source update, the computation of a new temporal view consists of the evaluation of an algebraic expression involving some auxiliary relations. We do not need to consult the entire history of source databases. For instance, consider Example 1.1. In our approach, $\delta^i(V)$ (*i.e.*, the view V at instant i) is computed by evaluating $\delta^i(R_\alpha) \cap (\Pi_N(\delta^i(UNIV) \bowtie \delta^i(EMP)))$ where R_α is an auxiliary relation that stores just a fraction of temporal information.

In our approach, as illustrated by the following example, a temporal data warehouse, although specified by a set of temporal expressions, is transformed into a set of non-temporal views over an extended database schema composed of (a) the source database schemas and (b) the auxiliary relation schemas.

Example 2.1 We consider a view V similar to the one presented in Example 1.1, but where we add a new relation $PHD[N, A, T]$ indicating the name of people applying for a PhD thesis T with an adviser A. Now our warehouse contains a temporal view specified by the following expression. It gives working students applying for a PHD degree.

$$V = \Pi_{N,A}(\blacksquare \Pi_N(UNIV \bowtie EMP) \bowtie PHD) \quad (2)$$

We consider that the source database evolves in time. The evolution of $UNIV$ and EMP is the one presented in Example 1.1. The instances of PHD are shown below.

	PHD
δ^0	$\{\langle \textit{manuel}, \textit{smith}, \textit{th1}\rangle, \langle \textit{john}, \textit{dupont}, \textit{th2}\rangle\}$
δ^1	$\{\langle \textit{manuel}, \textit{smith}, \textit{th1}\rangle, \langle \textit{john}, \textit{dupont}, \textit{th2}\rangle, \langle \textit{mary}, \textit{smith}, \textit{th3}\rangle\}$
δ^2	$\{\langle \textit{manuel}, \textit{smith}, \textit{th1}\rangle, \langle \textit{john}, \textit{dupont}, \textit{th2}\rangle, \langle \textit{mary}, \textit{smith}, \textit{th3}\rangle, \langle \textit{paul}, \textit{dubois}, \textit{th5}\rangle\}$
δ^3	$\{\langle \textit{manuel}, \textit{smith}, \textit{th1}\rangle, \langle \textit{john}, \textit{laurent}, \textit{th4}\rangle, \langle \textit{mary}, \textit{smith}, \textit{th3}\rangle, \langle \textit{paul}, \textit{dubois}, \textit{th5}\rangle\}$

The temporal expression $\blacksquare \Pi_N(UNIV \bowtie EMP)$ defines an auxiliary relation R_α. The way this auxiliary relation is incrementally built at each instant is explained below. We translate the temporal expression defining V into the non-temporal expression $V_t = \Pi_{N,A}(R_\alpha \bowtie PHD)$. Now we describe the maintenance of our warehouse, step by step.

Instant i=0: δ^0 is our database instance at instant $i = 0$. By definition, $\delta^0(R_\alpha)$ is empty. Thus, V_t is also empty at $i = 0$.

Update on δ^0: We consider now that an update is applied on instance δ^0. Given a relation R, we denote by $\triangle r^i$ (resp. $\triangledown r^i$) the set of tuples inserted in (resp. deleted from) instance $\delta^i(R)$. Therefore, we have $\triangle univ^0 = \{\langle math, mary\rangle\}$, $\triangledown univ^0 = \{\langle math, jane\rangle\}$, $\triangle emp^0 = \{\langle jane, bookseller\rangle\}$, $\triangledown emp^0 = \{\langle manuel, teacher\rangle, \langle mary, nurse\rangle\}$ and $\triangle phd^0 = \{\langle mary, smith, th3\rangle\}$.

Instant i=1: After performing the update, the new source instance is δ^1. Since in this situation "always in the past" refers only to $i = 0$, to evaluate R_α at instant $i = 1$, we just need to evaluate the expression $\Pi_N(UNIV \bowtie EMP)$ at instant $i = 0$. In this way, we obtain that R_α, at $i = 1$, is $\{\langle manuel\rangle, \langle john\rangle\}$. Now, to find the new instance of V, we compute the expression $\Pi_{N,A}(R_\alpha \bowtie PHD)$ at $i = 1$. Thus at instant $i = 1$, V contains $\{\langle john, dupont\rangle, \langle manuel, smith\rangle\}$.

Update on δ^1*:* $\triangle univ^1 = \{\langle cs, john\rangle, \langle math, paul\rangle\}$, $\bigtriangledown univ^1 = \{\langle law, john\rangle, \langle math, mary\rangle\}$, $\triangle emp^1 = \{\langle paul, clerk\rangle\}$ and $\triangle phd^1 = \{\langle paul, dubois, th5\rangle\}$.

Instant i=2: The new source instance is δ^2. To evaluate R_α at instant $i = 2$, we have to evaluate the expression $R_\alpha \cap (\Pi_N(UNIV \bowtie EMP))$ at instant $i = 1$. We have $\{\langle manuel\rangle, \langle john\rangle\} \cap \{\langle john\rangle\}$. Relation R_α, at $i = 2$, is $\{\langle john\rangle\}$. As before, the new instance of V is calculated by evaluating the relational algebra expression V_t at instant $i = 2$, resulting in $\{\langle john, dupont\rangle\}$.

Update on δ^2*:* $\bigtriangledown univ^2 = \{\langle cs, manuel\rangle\}$, $\bigtriangledown emp^2 = \{\langle paul, clerk\rangle\}$, $\triangle phd^2 = \{\langle john, laurent, th4\rangle\}$ and $\bigtriangledown phd^2 = \{\langle john, dupont, th2\rangle\}$.

Instant i=3: The new source instance is δ^3. To evaluate R_α at instant $i = 3$, we have to evaluate the expression $R_\alpha \cap (\Pi_N(UNIV \bowtie EMP))$ at instant $i = 2$. Thus relation R_α, at $i = 3$, is $\{\langle john\rangle\}$. The new instance of V is calculated by evaluating the relational algebra expression V_t at instant $i = 3$, resulting in $\{\langle john, laurent\rangle\}$. The following table shows the evolution of R_α.

	R_α
δ^0	$\emptyset$
δ^1	$\{\langle manuel\rangle, \langle john\rangle\}$
δ^2	$\{\langle john\rangle\}$
δ^3	$\{\langle john\rangle\}$

□

2.2 Self-maintainable temporal data warehouses

A warehouse is *self-maintainable* if its maintenance is done without source consultation [Laurent et al. 2001, Mohania and Kambayashi 2000, Quass et al. 1996]. From Example 2.1, our first proposal consists of storing, in the warehouse, the auxiliary relation R_α and the non-temporal view V_t over the extended database schema $\{UNIV, EMP, PHD, R_\alpha\}$. However, this is not sufficient to achieve self-maintenance. For instance, suppose we want to find the instance of V at instant $i = 1$. We need to compute the expression $\Pi_{N,A}(R_\alpha \bowtie PHD)$ over instance δ^1, *i.e.*, we have to consult $\delta^1(PHD)$. The question here is how to avoid this kind of operation since it can lead to anomalies [Zhuge et al. 1995]. In fact, the warehouse maintenance is decoupled from source updates and sources are not supposed to perform sophisticated operations in support of view maintenance.

To solve this problem, we store in the warehouse two types of auxiliary views, namely the *partial views* and the *complements*. A *partial view* is a non-temporal join view involving source relations and auxiliary relations resulting from the translation of temporal expressions into non-temporal ones. A *complement* is any set of non-temporal views which, together with the partial view, can re-compute all source relations.

Example 2.2 We consider the situation presented in Example 2.1. We define $\partial V = R_\alpha \bowtie PHD$ to be a "partial" view of V_t. Instead of storing V_t we propose to store ∂V in the warehouse. We notice that ∂V involves the join operator which is expensive to evaluate and so, this view is worth being kept materialized in the warehouse. On the other hand, V_t may be calculated by executing a projection over ∂V.
Besides the auxiliary relation R_α and the partial view ∂V, we suppose that the warehouse stores three views C_{EMP}, C_{UNIV} and C_{PHD}, complementary views of source relations EMP, $UNIV$ and PHD, respectively, with respect to the partial view ∂V. These complementary views

are defined in such a way that they satisfy the following properties: $C_{EMP} \cup \Pi_{N,J}\partial V = EMP$, $C_{UNIV} \cup \Pi_{N,C}\partial V = UNIV$ and $C_{PHD} \cup \Pi_{N,A,T}\partial V = PHD$ (see Theorem 4.2).
Remark: A projection over incompatible attributes (*i.e.*, $\pi_X(R)$ where X is not a subset of R's attributes) results in the empty relation over X. Thus, in this example, $\Pi_{N,C}\partial V$ and $\Pi_{N,J}\partial V$ are both empty (and thus, they are omitted in the expression $(\partial V)'$ below).

Now, to update the temporal view V it suffices to update the *materialized view* ∂V. As we will see in Section 5, this is achieved by evaluating the relational expression $(\partial V)'$:

$$(\partial V)' = (R_\alpha \cap \Pi_N(C_{UNIV} \bowtie C_{EMP})) \bowtie (C_{PHD} \cup \Pi_{N,A,T}\partial V \setminus \triangledown PHD \cup \triangle PHD)$$

For instance, in order to update the view V from instant $i = 1$ to instant $i = 2$, we have just to evaluate the *virtual view* $\Pi_{N,A}\partial V$. In our example, we have $\triangle phd^1 = \{\langle paul, dubois, th5\rangle\}$ and $\triangledown phd^1 = \emptyset$. At instant $i = 1$, $R_\alpha = \{\langle \textit{manuel}\rangle, \langle \textit{john}\rangle\}$ and the partial view ∂V = $\{\langle manuel, smith, th1\rangle, \langle john, dupont, th2\rangle\}$. Moreover, C_{UNIV} = $\{\langle cs, manuel\rangle, \langle math, mary\rangle, \langle law, john\rangle\}$, C_{EMP} = $\{\langle john, waiter\rangle, \langle jane, bookseller\rangle\}$ and C_{PHD}= $\{\langle mary, smith, th3\rangle\}$. Thus, at $i = 2$, $\partial V = \{\langle john, dupont, th2\rangle\}$ and $V = \{\langle john, dupont\rangle\}$. □

In Example 2.2 expression $(\partial V)'$ defines how partial view ∂V is updated. We notice that it involves only (i) the updates performed over the source relations and (ii) relations stored in the data warehouse (complementary views, the partial view ∂V, the auxiliary relation R_α). The warehouse maintenance is quite simplified since it can be performed without consulting the sources: one just needs to evaluate $\Pi_{N,A}(\partial V)'$, a non-temporal relational algebra expression.
Remark: Notice that our underlying temporal data model follows a time-implicit perspective. In a time-implicit approach, a temporal database instance is a finite sequence of relational instances. On the other hand, in a time-explicit approach each relation is augmented with an extra column storing the time instants of validity of each tuple. Our choice of an implicit time representation (the so-called *snapshot temporal model*) is due to the fact that within this perspective, the most natural temporal query languages (such as those in [Abiteboul et al. 1996, Chomicki 1994, Clifford and Tuzhilin 1990, Niwinsky and Toman 1996]) are extensions or algebraic counterparts of Temporal Logic TL [Emerson 1990]. As modal logics in general, TL provides a good balance between expressive power and computational complexity [Gradel 1999, Sistla and Clarke 1985, Stockmeyer 1974].

Figure 1 shows how we put together the two main features discussed in this section by illustrating the general structure of our self-maintainable temporal data warehouse. In this work, a warehouse **W** is specified by a set of temporal expressions from which we derive two sets of non-temporal views, W_m and W_v, that we call *the materialized component* and *the virtual component* of **W**, respectively. These two components together completely determine the temporal data warehouse.

For instance, let **W** be the warehouse of Examples 2.1 and 2.2, specified by $V = \Pi_{N,A}(\blacksquare \Pi_N(\ UNIV \bowtie EMP) \bowtie PHD)$. Its materialized component W_m is composed by the auxiliary relation R_α, the partial view ∂V and complementary views $C_{EMP}, C_{UNIV}, C_{PHD}$. The virtual component W_v is defined by $\Pi_{N,A}\partial V$.

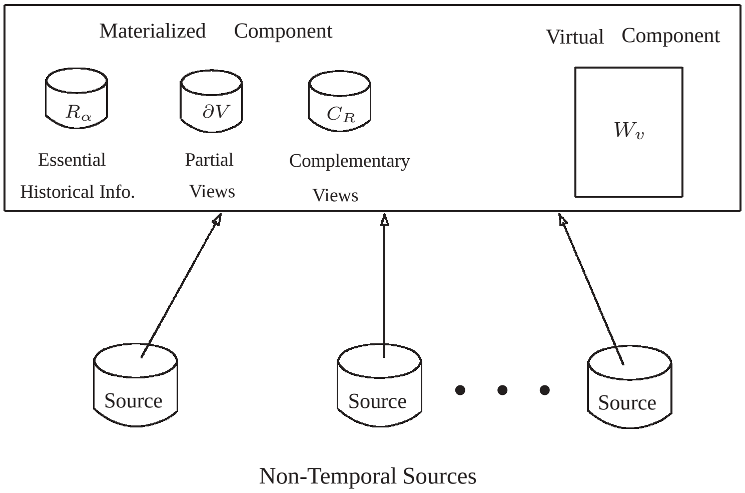

Figure 1: A Self-Maintenable Temporal Data Warehouse and non-temporal sources

It is worth pointing out that, in this paper, we combine in a uniform way two previously unrelated techniques based on auxiliary views: The maintenance of the temporal data warehouse is achieved by means of a technique that was originally used to check temporal integrity constraints [Chomicki 1995], while self-maintainability is achieved by extending a complement-based approach [Laurent et al. 2001] for the temporal setting. In this context, the materialization of auxiliary relations, *i.e.*, information that is not stored for application reasons but for "operational" reasons, is used successfully in two fundamentally different ways, namely, (i) to reduce the temporal setting to a non-temporal one and (ii) to get around the decoupling of non-temporal data sources and temporal data warehouse (which even in the non-temporal setting is the cause of update anomalies).

3 Temporal Data Model

In this section, we introduce the temporal data model underlying our temporal data warehouses and the temporal language used to specify them. We assume the reader to be familiar with the basics of relational databases [Abiteboul et al. 1995] but we briefly recall some definitions and notations used in this paper.

3.1 Relational database basic notations

Let **Atomtype** be a finite set of *atomic types*. A *type* is an atomic type τ or $\{\tau\}$. For each atomic type τ, we assume the existence of a countable infinite set **dom**(τ), called the *domain* of τ, such that **dom**(τ_1) $\cap$ **dom**(τ_2) $= \emptyset$ if $\tau_1 \neq \tau_2$. For non-atomic types $\{\tau\}$,

we define **dom**($\{\tau\}$) as $\wp$(**dom**(τ)), (*i.e.*, the power set of **dom**(τ)). We assume as well the existence of two countable infinite sets: **att**, a set of attributes names and **relname**, a set of relation names. The sets **att**, **relname** and **dom**(τ), for $\tau \in$ **Atomtype**, are assumed to be pairwise disjoint. A type is associated to each attribute A $\in$ **att**. An attribute can be *atomic* or *set-valued* depending if its associated type is atomic or not.

As in [Ozoyoglu et al. 1987], we extend the relational model to incorporate set-valued attributes and aggregate functions. This is justified by the fact that warehouses usually contain aggregated data, which can be naturally manipulated using set-valued attributes. We notice that relations with set-valued attributes constitute a subset of (non first normal form) relations [Abiteboul and Bidoit 1984, Jaeschke and Schek 1982]. In fact, our approach can be easily adapted to treat complex value relations since adding the tuple constructor to the traditional relational data model is considerably easier than adding the set constructor [Abiteboul et al. 1995].

We assume the existence of a function *sort* that associates a finite set of attributes U to each relation name R. A *relation schema* is a relation name R and we write $R[U]$ to indicate that $sort(R) = U$. A *database schema* is a non-empty finite set **R** of relational schemas. A tuple u with sort U is a function that, for each attribute $A \in U$ of type τ, associates a constant $a \in$ **dom**(τ) (if A is an atomic attribute) or a set $s \in \wp$(**dom**(τ)) (if A is a set-valued attribute). The value of tuple u on an attribute $A \in U$ (or on a set of attributes $V \subseteq U$) is denoted by $u[A]$ (respectively $u[V]$). If u and v are two tuples having sort U and $X \subseteq U$ then we write $u[X] = v[X]$ if $u[A] = v[A]$ for all $A \in X$.

An *instance* or *relation* over a schema R of sort U is a finite set of tuples over U. An *instance* over a database schema **R** is a mapping that associates an instance over R to each relation schema $R \in$ **R**. The set of all instances over a relation schema R (resp. a database schema **R**) is denoted by *Inst*(R) (resp. *Inst*(**R**)).

For each *atomic* attribute A, we assume the existence of a countable infinite set **agg** $_A$ of aggregate function names. We assume also that, if A and B are distinct attributes then **agg**$_A$ and **agg**$_B$ are disjoint. To each $f_A \in$ **agg**$_A$ is associated a function f_A : *Inst*$_A \rightarrow$ **dom**(τ), where τ is the type of the attribute A and *Inst* $_A$ = $\{I \in$ *Inst*$(R) \mid R$ is a relation schema and $A \in$ sort$(R)\}$.

We assume that the following *uniformness property* [Klug 1982] is verified: Let A and B be two atomic attributes of the same type τ and let I and J be two instances in *Inst*$_A$ and *Inst*$_B$ respectively. Suppose that the column corresponding to the attribute A of instance I is identical to the column corresponding to the attribute B of instance J, *i.e.*, for each $a \in$ **dom**(τ) there are as many occurrences of a in the A-column of I as in the B-column of J. Then, for each $f_A \in$ **agg**$_A$ there exists $g_B \in$ **agg**$_B$ such that $f_A(I) = g_B(J)$. For instance, let $R[A, B]$ be a relation schema where A and B are atomic attributes of type **Integer**, and let I be an instance over R. Let **sum** $_A$ be the aggregate function which returns the sum of the A-column of I. Then there exists an "identical" aggregate function **sum**$_B$ returning the sum of the elements in the B-column of I. We notice that an aggregate function is applied over a *relation* instance I and

performs a computation over one column of I. This definition of aggregate functions follows the lines of [Klug 1982] and (as it has been pointed out there) is justified by the fact that it avoids dealing with duplicates.

3.2 Temporal databases

Our temporal data model is based on the time-implicit perspective widely adopted for its simplicity [Abiteboul et al. 1996, Chomicki 1994, Niwinsky and Toman 1996]. The structure of time is considered as being isomorphic to natural numbers. A *temporal database (instance)* or a *database history* over a database schema **R** is defined as being a finite sequence $\delta = (\delta^0, \ldots, \delta^n)$ of databases instances over **R**. We sometimes call the instance δ^i the *database state* at instant i. For each $R \in$ **R** and $i \in \{0, \ldots, n\}$ we denote by r^i the instance $\delta^i(R)$, *i.e.*, the relation associated to R at instant i. The last state n is the current state of the temporal database.

3.3 A temporal relational algebra with set-valued attributes and aggregate functions

A temporal algebra is an essential part of a DBMS when it is designed to treat time-varying data. In [Mckenzie and Snodgrass 1991], a variety of temporal algebras, which are extensions of classical relational algebra, have been studied. Our temporal data model is based on one of those temporal algebras, the Tuzhilin's Temporal Algebra (TA for short), introduced in [Clifford and Tuzhilin 1990]. Our choice was motivated by the fact that TA satisfies most of the compatible classification criteria discussed in [Mckenzie and Snodgrass 1991]. Furthermore, its operators were designed to be powerful yet simple and to be based on the well-accepted formalism of temporal logic [Emerson 1990].

To specify temporal views we extend the past fragment of TA, in order to manipulate set-valued attributes and aggregate functions. We denote this extension by EPTA. Our treatment of aggregates and set-valued attributes is based on the formalisms introduced in [Klug 1982, Ozoyoglu et al. 1987]. We recall that in these approaches, in order to simplify the presentation, the underlying domain of attributes is assumed to be the set $\mathbb{N}$ of the natural numbers and relational algebra is presented under the so-called *unnamed perspective* [Abiteboul et al. 1995], *i.e.*, attribute names do not appear in the expressions of the language. In our presentation, in order to make it possible to present more readable and real-world examples, attributes take values in the domain of their associated type and the algebra expressions are defined using the *named perspective*. Next, we briefly present the syntax and the semantics of EPTA.

Syntax of EPTA

Let **R** be a database schema. An EPTA expression over **R** and its *output sort* are both defined inductively. The *basic* EPTA expressions are defined as follows:

1. If A is an attribute of type τ (not necessarily atomic) and $a \in$ **dom**(τ), then $\{\langle A : a\rangle\}$ is a basic EPTA expression with sort($\{\langle A : a\rangle\}$) = $\{A\}$.
2. Each R ($R \in$ **R**) is a basic EPTA expression of sort identical to sort(R).

A basic expression is an EPTA expression. Moreover, if S and Q are EPTA expressions then the following expressions are EPTA expressions:

1. *Selection* $\sigma_G Q$, where G is a selection formula, *i.e.*, a conjunction of formulas of the form $A\Theta B$ where (i) $A \in sort(Q)$, (ii) $B \in sort(Q)$ or $B \in$ **dom**(τ) where τ is the type of A and, depending on the type of A and B, (iii) Θ is either a predicate for comparing sets ($=, \subseteq, \subset, \neq, \ldots$), or the membership predicates $\in$, $\notin$ or a predicate for comparing constants ($=, \leq, \geq, \ldots$). Moreover, $sort(\sigma_G Q)$ = $sort(Q)$.
2. *Projection*: $\Pi_X Q$, where X is a finite subset of **att**. We define $sort(\Pi_X Q) = X$.
3. *Renaming*: ρQ, where ρ is a renaming of the attributes in $sort(Q)$ and we assume that for each attribute A, $\rho(A)$ and A are attributes of the same type. We define $sort(\rho Q)$ = $\rho(sort(Q))$.
4. *Natural join*: $S \bowtie Q$, where $sort(S \bowtie Q) = sort(S) \cup sort(Q)$.
5. *Union*: $S \cup Q$, where $sort(S \cup Q) = sort(S) = sort(Q)$.
6. *Difference* : $S \setminus Q$, where $sort(S \setminus Q) = sort(S) = sort(Q)$.
7. *Aggregate formation*: $\langle [X], f_A : B\rangle Q$, where $X \subseteq sort(Q)$, the function $f_A \in$ **agg**$_A$ and A and B are atomic attributes of the same type such that $A \in sort(Q)$, $A \notin X$ and $B \notin sort(Q)$. We define $sort(\langle [X], f_A : B\rangle Q) = X \cup \{B\}$;
8. *Unpack*: $U_{A:B} Q$, where $A \in sort(Q)$, $B \in$ **att** is a new atomic attribute w.r.t. $sort(Q)$ ($B \notin sort(Q)$). Moreover, A and B have both the same atomic type τ or A has type $\{\tau\}$ and B has type τ, where τ is an atomic type. We define $sort(U_{A:B} Q) = (sort(Q) \setminus \{A\}) \cup \{B\}$.
9. *Pack*: $P_{A:B} Q$, where $A \in sort(Q)$, $B \in$ **att** is a new set-valued attribute w.r.t. $sort(Q)$ ($B \notin sort(Q)$). Moreover, A and B have both the same type $\{\tau\}$ or A has type τ and B has type $\{\tau\}$, where τ is an atomic type. We define $sort(P_{A:B} Q) = (sort(Q) \setminus \{A\}) \cup \{B\}$.
10. *Previous*: **prev** Q, where $sort($**prev** $Q)$ = $sort(Q)$.
11. *Since*: S **since** Q, where $sort(S$ **since** $Q) = sort(S) = sort(Q)$.
12. *Sometime in the past*: $\blacklozenge Q$ where $sort(\blacklozenge Q) = sort(Q)$.
13. *Always in the past*: $\blacksquare Q$, where $sort(\blacksquare Q) = sort(Q)$.

Semantics of EPTA

A temporal expression Q of EPTA is evaluated on a temporal database $\delta = (\delta^0$, $\ldots$, $\delta^n)$ at an instant $i, 0 \leq i \leq n$. This evaluation (denoted by $(\delta, i)(Q)$) gives the answer of the query specified by the EPTA expression Q. It represents the set of tuples of $sort(Q)$ satisfying the expression Q at instant i. For expressions 1. to 6., $(\delta, i)(Q)$ is defined as in relational algebra. For instance, $(\delta, i)(\sigma_G Q)$ = $\{u \mid u \in (\delta, i)(Q)$ and u

satisfies the selection formula $G\}$. For the projection $\Pi_X Q$, where $X \not\subseteq sort(Q)$, we define $(\delta, i)(Q) = \emptyset$. We note that the *intersection operator* $P \cap Q$ (with its obvious semantics) can be easily derived from join and selection [Abiteboul et al. 1996]. For expressions 7. to 13., the semantics is given below.

7. Let $\circ$ denote tuple concatenation. Then,

   $$(\delta, i)(\langle [X], f_A : B\rangle Q) = \{u[X] \circ y \mid u \in (\delta, i)Q \wedge \\ y = f_A(\{u' \mid u' \in (\delta, i)Q \text{ and } u'[X] = u[X]\})\}$$

   Intuitively, this operation assembles all tuples belonging to the answer of Q at instant i which have the same values for the attributes of X and executes f_A over the A-column of the resulting set of tuples. The attribute B is associated to the column corresponding to the result of the application of f_A.
8. Let Y denote the set $sort(Q) \setminus \{A\}$. In the case that A is a non-atomic attribute, let $\mathcal{A}$ be the set

   $$\{u \mid u \text{ is a tuple over } Y \cup \{B\} \text{ such that there exists} \\ v \in (\delta, i)(Q) \text{ with } u[B] \in v[A] \text{ and } u[Y] = v[Y]\}$$

   Then, $(\delta, i)(U_{A:B}Q) = \begin{cases} \rho Q, & \text{where } \rho \text{ is a renaming of } A \text{, with } \rho(A) = B \\ & \text{if } A \text{ is an atomic attribute} \\ \mathcal{A} & \text{if } A \text{ is a set-valued attribute} \end{cases}$

   Intuitively, $(\delta, i)(U_{A:B}Q)$ is the set of all tuples obtained by transforming each tuple in Q which has the form $\langle\{a_1, ..., a_n\}, c_1, ..., c_m\rangle$ into the set of tuples $\{\langle a_1, c_1, \ldots, c_m\rangle, \langle a_2, c_1, \ldots, c_m\rangle, \ldots, \langle a_n, c_1, \ldots, c_m\rangle\}$.
9. Let Y denote the set $sort(Q) \setminus \{A\}$. For each $v \in (\delta, i)(Q)$, let $\mathcal{B}_v$ denote the set $\{z[A] \mid z \in (\delta, i)Q \text{ and } z[Y] = v[Y]\}$. Then,

   $$(\delta, i)(P_{A:B}Q) = \{u \mid u \text{ is a tuple over } Y \cup \{B\} \text{ and there exists } v \in (\delta, i)(Q) \\ \text{with } u[Y] = v[Y] \text{ and } u[B] \text{ is defined by equation (3) below}\}$$

   $$u[B] = \begin{cases} \mathcal{B}_v & \text{if } A \text{ is an atomic attribute} \\ \bigcup_{\mathcal{B} \in \mathcal{B}_v} \mathcal{B} & \text{otherwise} \end{cases} \quad (3)$$

   Intuitively, $(\delta, i)(P_{A:B}Q)$ is the set obtained by transforming packages of tuples in Q that have the form $\{\langle a_1, c_1, \ldots, c_m\rangle, \langle a_2, c_1, \ldots, c_m\rangle, \ldots, \langle a_n, c_1, \ldots, c_m\rangle\}$ into a unique *packed tuple* $\langle\{a_1, \ldots, a_n\}, c_1, \ldots, c_m\rangle$.

We illustrate the use of operators 7. to 9. over a non-temporal instance.

Example 3.1 We consider relations $R[A, B]$ and $S[A, C]$ where A and B are atomic attributes and C is a set-valued attribute. We assume instances over R as $\{\langle a1, 5\rangle\ \langle a1, 20\rangle,\ \langle a2, 45\rangle\}$ and over S as $\{\langle a1, \{20, 30\}\rangle, , \langle a3, \{40, 50\}\rangle\}$. The relations created by $P_{B:M}R$, $U_{C:N}S$, and $\langle [A], sum_B : Tot\rangle R$ are the following:

$P_{B:M}R$:	$\{\langle a1, \{5, 20\}\rangle, \langle a2, \{45\}\rangle\}$
$U_{C:N}S$:	$\{\langle a1, 20\rangle\ , \langle a1, 30\rangle\ , \langle a3, 40\rangle\ , \langle a3, 50\rangle\}$
$\langle [A], sum_B : Tot\rangle R$	$\{\langle a1, 25\rangle\ , \langle a2, 45\rangle\}$

□

At first glance, we can imagine $pack$ and $unpack$ to be inverse operations. However, in general, $Q \neq U_{B:A}(P_{A:B}(Q))$ and $Q \neq P_{B:A}(U_{A:B}(Q))$ as the following example illustrates. Clearly, when A is an atomic attribute, $Q = U_{B:A}(P_{A:B}(Q))$.

Example 3.2 We consider the following instance over $Q[X, A]$.

Q	$\{\langle x1, \{20, 30\}\rangle, \langle x1, \{2, 3\}\rangle, \langle x2, \{2, 3\}\rangle\}$
$P_{A:B}(Q)$	$\{\langle x1, \{2, 3, 20, 30\}\rangle, \langle x2, \{2, 3\}\rangle\}$
$U_{B:A}(P_{A:B}(Q))$	$\{\langle x1, 20\rangle, \langle x1, 30\rangle, \langle x1, 2\rangle, \langle x1, 3\rangle, \langle x2, 2\rangle, \langle x2, 3\rangle\}$
$U_{A:B}(Q)$	$\{\langle x1, 20\rangle, \langle x1, 30\rangle, \langle x1, 2\rangle, \langle x1, 3\rangle, \langle x2, 2\rangle, \langle x2, 3\rangle\}$
$P_{B:A}(U_{A:B}(Q))$	$\{\langle x1, \{2, 3, 20, 30\}\rangle, \langle x2, \{2, 3\}\rangle\}$

□

For expressions $10.$ to $13.$ we use the following notation: if Q is an EPTA expression, we denote $(\delta, j)(Q)$ by q^j.

10. $(\delta, i)(\textbf{prev}\ Q) = \begin{cases} q^{i-1} & \text{if } i \geq 1 \\ \emptyset & \text{otherwise} \end{cases}$
11. For each $k \in \{0, \ldots, n\}$, let e^k be inductively defined by: $e^0 = \emptyset$ and $e^k = s^k \cap (q^{k-1} \cup e^{k-1})$, for $k > 0$. We define: $(\delta, i)(S\ \textbf{since}\ Q) = e^i$, for $i \in \{0, \ldots, n\}$.

    Intuitively, $(\delta, i)(S\ \textbf{since}\ Q)$ is the set of all tuples u such that there exists an instant $j < i$ where $u \in (\delta, j)(Q)$ and for all k such that $j < k \leq i$, we have $u \in (\delta, k)(S)$.
12. For each $k \in \{0, \ldots, n\}$, let e^k be inductively defined by: $e^0 = \emptyset$ and $e^k = q^{k-1} \cup e^{k-1}$, for $k > 0$. We define: $(\delta, i)(\blacklozenge Q) = e^i$, for $i \in \{0, \ldots, n\}$.

    Intuitively, $(\delta, i)(\blacklozenge Q)$ is the set of all tuples u such that there exists an instant $j < i$ where $u \in (\delta, j)(Q)$.
13. For each $k \in \{0, \ldots, n\}$, let e^k be inductively defined by: $e^0 = \emptyset$, $e^1 = q^0$ and $e^k = q^{k-1} \cap e^{k-1}$, for $k > 1$. We define: $(\delta, i)(\blacksquare Q) = e^i$, for $i \in \{0, \ldots, n\}$.

    Intuitively, $(\delta, i)(\blacksquare Q)$ is the set of all tuples u such that for all instants $j < i$ one has $u \in (\delta, j)(Q)$.

Example 3.3 We consider the source database from Example 1.1 and the EPTA expression S **since** Q, where $S = \Pi_N UNIV$ and $Q = \Pi_N EMP$. This expression gives the names of people who have been studying at the university since the moment they got a job. At instant $i = 3$, the answer to this query is computed as follows:

$$(\delta, 3)(S\ \textbf{since}\ Q) = s^3 \cap (q^2 \cup (s^2 \cap (q^1 \cup (s^1 \cap (q^0 \cup (s^0 \cap \emptyset))))))$$

We have: $(s^0 \cap \emptyset) = \emptyset$, $q^0 \cup \emptyset = \{\langle manuel\rangle, \langle john\rangle, \langle mary\rangle\}$,

$s^1 \cap \{\langle manuel\rangle, \langle john\rangle, \langle mary\rangle\} = \{\langle manuel\rangle, \langle john\rangle, \langle mary\rangle\}$

$q^1 \cup \{\langle manuel\rangle, \langle john\rangle, \langle mary\rangle\} = \{\langle manuel\rangle, \langle john\rangle, \langle mary\rangle, \langle jane\rangle\}$,

$s^2 \cap \{\langle manuel\rangle, \langle john\rangle, \langle mary\rangle, \langle jane\rangle\} = \{\langle manuel\rangle, \langle john\rangle\}$

$q^2 \cup \{\langle manuel\rangle, \langle john\rangle\} = \{\langle manuel\rangle, \langle john\rangle, \langle jane\rangle, \langle paul\rangle\}$,

$s^3 \cap \{\langle manuel\rangle, \langle john\rangle, \langle jane\rangle, \langle paul\rangle\} = \{\langle john\rangle, \langle paul\rangle\}$

Only John and Paul have been studying at the university since they got a job. □

If α is an EPTA expression and β a sub-expression of α, we say that β is a *pure temporal sub-expression* of α if β has the form **prev** Q, S **since** Q, $\blacklozenge Q$ or $\blacksquare Q$ for some EPTA expression Q.

4 A Temporal and Self-Maintainable Data Warehouse

In our approach a temporal data warehouse **W** is specified by a set of EPTA expressions over **R**. From this set of temporal expressions, we derive two sets of non-temporal views W_m and W_v which completely characterize **W**. In this way, we define a temporal data warehouse **W** as a pair (W_m,W_v) of sets of non-temporal views. The first set W_m, called the *materialized component* of **W**, represents the information physically stored in the data warehouse. The second set W_v, called the *virtual component* of **W**, contains ERA (Extended Relational Algebra) expressions involving only relations stored in the materialized component. We emphasize that, although both components are derived from the temporal expressions specifying **W**, they are non-temporal entities. Moreover, the materialized component gives a flexibility to our approach, since the auxiliary views stored there can be used not only to compute the specified views of **W** but also to answer potential ad-hoc queries.

Before introducing the components W_m and W_v, we need to establish some notations concerning the evolution of source relations and the data warehouse. Given a database instance $\delta = (\delta^0, \ldots, \delta^n)$ over **R**, there is a correspondence between the evolution of the database and the evolution of the warehouse, that is, the data warehouse history associated to δ is $w = (w^0, \ldots, w^n)$ where for each $0 \leq i \leq n$, w^i is the warehouse corresponding to δ^i. Thus, there is a complete order-preserving mapping between the states of the warehouse and the states of the sources, *i.e.*, in response to an update u applied to the instance δ^i to obtain δ^{i+1}, the current warehouse state w^i is modified to produce w^{i+1}.

The transitions between states δ^i and δ^{i+1} are obtained by inserting tuples to δ^i or deleting tuples from δ^i. These updates are performed by consistency-preserving transactions. Therefore, for each source relation r^i at instant i, its updated version r^{i+1} at instant $i+1$ is obtained by $r^{i+1} = r^i \setminus \bigtriangledown r^i \cup \bigtriangleup r^i$, where $\bigtriangledown r^i$ is the set of tuples deleted from r^i and $\bigtriangleup r^i$ is the set of tuples inserted in r^i. We assume that $\bigtriangleup r^i \cap \bigtriangledown r^i = \emptyset$ and that ERA expressions without parenthesis are evaluated from left to right.

4.1 The materialized component W_m

In this section we concentrate our attention on the materialized component of our temporal data warehouse. In fact, the materialized component W_m is defined as a triple[1] $W_m = (\mathcal{T}, \partial\mathcal{V}, \mathcal{C})$ where $\mathcal{T}$ is a set of auxiliary relations containing essential temporal information to maintain the warehouse, $\partial\mathcal{V}$ is a set of non-temporal views (involving only the join operator) over $\mathbf{R} \cup \mathcal{T}$ and $\mathcal{C}$ is a set of complementary views allowing the warehouse maintenance without consulting the source databases.

[1] Sometimes, we prefer a simpler representation for W_m as the set of views $\mathcal{T} \cup \partial\mathcal{V} \cup \mathcal{C}$.

4.1.1 Constructing the auxiliary relations $\mathcal{T}$

In this section, we consider two important aspects of our approach.

1. The construction of the set $\mathcal{T}$, included in the materialized component W_m, containing essential temporal information for the warehouse maintenance.
2. The translation of the temporal expressions that specify the temporal views of our warehouse in terms of non-temporal ones.

To achieve these goals, we adapt to the data warehouse context the method introduced in [Chomicki 1995] for checking temporal constraints. We also extend it to support aggregates and set-valued attributes. The basic idea of this approach is that in order to evaluate a given finite set of temporal views it is *not* necessary to store the entire database history. Instead, the data warehouse is augmented with new auxiliary relations.

We define the auxiliary relation schemas associated to the set of $EPTA$ expressions defining **W**. In the remainder of this section, we assume that the $EPTA$ expressions specifying the temporal views are defined over the (source) database schema **R**. For each temporal view V and each pure temporal sub-expression α of V with *sort*(α) = $\{A_1, A_2, \ldots, A_m\}$, we associate a new relational schema $R_\alpha[A_1, A_2, \ldots, A_m]$. We denote by $\mathcal{T}$ the set of all these new auxiliary relation schemas associated to the $EPTA$ expressions specifying the temporal views. For instance, if V is the temporal view of Example 2.1, then V has only one pure temporal sub-expression α = $\blacksquare \Pi_N\ (UNIV \bowtie EMP)$. So, we associate to V one auxiliary relation schema $R_\alpha[N]$ and $\mathcal{T}$ = $\{R_\alpha\}$.

Now, we describe the semantics of the auxiliary relations. More precisely, we show how the *contents* of the auxiliary relations are modified when non-temporal source relations are updated. We emphasize that auxiliary relations must store *essential* temporal information to maintain the temporal views of our data warehouse.

Let $\delta = (\delta^0, \ldots, \delta^n, \delta^{n+1})$ be a temporal instance over **R**. We will associate to δ an *extended temporal instance* $\hat{\delta}$ =($\hat{\delta}^0, \ldots, \hat{\delta}^n, \hat{\delta}^{n+1}$) over the *extended database schema* **S** = **R** $\cup\, \mathcal{T}$.

For each instant i:

– $\hat{\delta}^i(R)$ = $\delta^i(R)$ (denoted by r^i) for $R \in$ **R** and
– $\hat{\delta}^i(R_\alpha)$ is denoted by r^i_α, for $R_\alpha \in \mathcal{T}$.

We recall that at any instant i, the relations r^i_α are stored in the current data warehouse and the relations r^i are stored in the source databases.

The construction of auxiliary relations r^i_α is defined inductively over the structure of the expression α in such a way that r^i_α contains exactly the values that make α true in i. By definition, each auxiliary relation r^i_α is empty at the initial instant $i = 0$, because each *past* temporal expression α is evaluated as *empty* at $i = 0$ (see the semantics of EPTA expressions 10. to 13. in Section 3). Relations r^{i+1}_α will be obtained from r^i_α and the *source* instances δ^i and δ^{i+1}. More precisely, r^{i+1}_α is evaluated as the answer to a *relational query* $\alpha^{i,i+1}$ over $\hat{\delta}^i$ and δ^{i+1}. So, $r^0_\alpha = \emptyset$ and r^{i+1}_α = $\{u \mid u$ is an answer of $\alpha^{i,i+1}$ evaluated over $\hat{\delta}^i$ and $\delta^{i+1}\}$.

Table 4.1 contains a description of the construction of the relational expression $\alpha^{i,i+1}$ which is inductive on the structure of α. We notice that in order to define each $\alpha^{i,i+1}$ we make use of an auxiliary expression $\alpha^{i,i}$ which is also inductively defined in Table 4.1. As the expression $\alpha^{i,i+1}$ will be evaluated over *the union* of instances $\hat{\delta}^i$ and δ^{i+1}, we denote by R^i (resp. R^{i+1}) the relational expression R ($R \in$ **S**) which should be evaluated on δ^i (resp. δ^{i+1}).

Table 4.1 - Inductive procedure for constructing the auxiliary views. (Q and P are EPTA expressions).

α	$\alpha^{i,i}$	$\alpha^{i,i+1}$
R	R^i	R^{i+1}
$\sigma_G Q$	$\sigma_G Q^{i,i}$	$\sigma_G Q^{i,i+1}$
$\Pi_X Q$	$\Pi_X Q^{i,i}$	$\Pi_X Q^{i,i+1}$
ρQ	$\rho Q^{i,i}$	$\rho Q^{i,i+1}$
$Q \bowtie P$	$Q^{i,i} \bowtie P^{i,i}$	$Q^{i,i+1} \bowtie P^{i,i+1}$
$Q \cup P$	$Q^{i,i} \cup P^{i,i}$	$Q^{i,i+1} \cup P^{i,i+1}$
$Q \setminus P$	$Q^{i,i} \setminus P^{i,i}$	$Q^{i,i+1} \setminus P^{i,i+1}$
$\langle [X], f_{A_i} : B \rangle Q$	$\langle [X], f_{A_i} : B \rangle Q^{i,i}$	$\langle [X], f_{A_i} : B \rangle Q^{i,i+1}$
$U_{A:B} Q$	$U_{A:B} Q^{i,i}$	$U_{A:B} Q^{i,i+1}$
$P_{A:B} Q$	$P_{A:B} Q^{i,i}$	$P_{A:B} Q^{i,i+1}$
prev Q	R^i_α	$Q^{i,i}$
P ***since*** Q	R^i_α	$(P$ ***since*** $Q)^{i,i} \cup Q^{i,i}) \cap P^{i,i+1}$
$\blacklozenge Q$	R^i_α	$(\blacklozenge Q)^{i,i} \cup Q^{i,i}$
$\blacksquare Q$	R^i_α	$(\blacksquare Q)^{i,i} \cap Q^{i,i}$ *for* $i \geq 1$ *and* $Q^{0,0}$ *for* $i = 0$

For each temporal view V, we associate an extended relational algebra expression V_t over the extended database schema **S** by replacing each *maximal* pure temporal sub-expressions[2] α of V by its corresponding relation schema R_α.

Example 4.1 below illustrates how to use Table 4.1 to construct the auxiliary relations associated to a temporal view V (specified by an EPTA expression) and to translate this temporal view into a view V_t specified by an ERA (Extended Relational Algebra) expression. Note that, in Section 1, we have illustrated our method by a simple example where only atomic types are used. From now on, we use a running example where non-atomic types are considered.

Running Example 4.1 Let **INFO1** = $\{UNIV, EMP, PROF\}$ be a database schema. We have $UNIV[C, N]$ and $EMP[N, J]$ (see Example 1.1) and the new relation $PROF[C, I]$ which stores information about courses *C* and professors *I*. We consider the following temporal database instance.

	$UNIV$
δ^0	$\{\langle cs, manuel \rangle, \langle math, jane \rangle\}$
δ^1	$\{\langle cs, manuel \rangle, \langle math, mary \rangle, \langle law, john \rangle\}$
δ^2	$\{\langle cs, manuel \rangle, \langle cs, john \rangle\}$

[2] Maximal pure temporal sub-expressions are those expressions which are not a proper sub-expression of a pure temporal sub-expression of V.

	EMP
δ^0	$\{\langle manuel, teacher\rangle, \langle john, waiter\rangle, \langle mary, nurse\rangle\}$
δ^1	$\{\langle john, waiter\rangle, \langle jane, bookseller\rangle, \langle manuel, teacher\rangle\}$
δ^2	$\{\langle jane, bookseller\rangle, \langle paul, clerk\rangle\}$

	$PROF$
δ^0	$\{\langle cs, pr1\rangle, \langle math, pr1\rangle\}$
δ^1	$\{\langle cs, pr1\rangle, \langle cs, pr2\rangle, \langle math, pr2\rangle, \langle law, pr3\rangle, \langle law, pr4\rangle\}$
δ^2	$\{\langle cs, pr1\rangle, \langle cs, pr2\rangle, \langle math, pr2\rangle, \langle law, pr3\rangle, \langle law, pr4\rangle\}$

We suppose a data warehouse over **INFO1**, having just one temporal view specified by the EPTA expression:

$$V = P_{I:SetProf}(\Pi_C(UNIV \bowtie \blacklozenge EMP) \bowtie PROF) \tag{4}$$

This view V gives the university courses having students who have had (at least once in the past) an employment and, for each of these courses, the view prints the set of professors currently associated to it.
Clearly, V has only one pure temporal sub-expression $\alpha = \blacklozenge EMP$ which we associate to the auxiliary relation R_α. From Table 4.1 (row 13), we know that, at each instant i, $\alpha^{i,i+1} = \blacklozenge EMP^{i,i} \cup EMP^{i,i}$ and $\alpha^{i,i} = R^i_\alpha$. Thus, to compute the contents of the auxiliary relation R_α at each instant $i \in \{0, 1, 2\}$ we proceed as follows:

– By definition, $r^0_\alpha = \emptyset$. We have $\alpha^{0,1} = (\blacklozenge EMP)^{0,0} \cup EMP^{0,0}$ and, using Table 4.1 (row 13, column 1), we find $\alpha^{0,1} = R^0_\alpha \cup EMP^0$. Thus, the instance r^1_α is obtained by the union of the instances $r^0_\alpha \cup emp^0$.
– $\alpha^{1,2} = (\blacklozenge EMP)^{1,1} \cup EMP^{1,1} = R^1_\alpha \cup EMP^1$. Thus, the instance r^2_α is $r^1_\alpha \cup emp^1$.

	R_α
δ^0	$\emptyset$
δ^1	$\{\langle manuel, teacher\rangle, \langle john, waiter\rangle, \langle mary, nurse\rangle\}$
δ^2	$\{\langle manuel, teacher\rangle, \langle john, waiter\rangle, \langle mary, nurse\rangle, \langle jane, bookseller\rangle\}$

The above table describes the relation r^i_α for $i \in \{0, 1, 2\}$. Note that the ERA expression associated to the temporal view V is $V_t = P_{I:SetProf}(\Pi_C(UNIV \bowtie R_\alpha) \bowtie PROF)$. □

One of the main results of this section is the following theorem which guarantees that evaluating the relational algebra expression V_t at the current extended instance $\hat{\delta}^i$ is the same as evaluating the $EPTA$ expression V at the temporal instance $(\delta^0, \ldots, \delta^i)$ at instant i, *i.e.*, the auxiliary relations r^i_α contain essential historical information to evaluate the temporal expression V.

Theorem 4.1 *Given a database schema **R** and a set $\mathcal{T}$ of auxiliary relations, let $\delta = (\delta^0, \ldots, \delta^n)$ be a temporal instance over **R** and $(\hat{\delta}^0, \ldots, \hat{\delta}^n)$ the associated extended instance over* **S**$=$ ***R*** $\cup \mathcal{T}$*. If V is an EPTA expression over **R** and V_t is its translation into an ERA expression over* **S***, then for all $i \in \{0, \ldots, n\}$: $\{u \mid u$ is an answer of V_t on instance $\hat{\delta}^i\} = \{u \mid u$ is an answer of V on $(\delta, i)\}$.*

Proof: We can view $\hat{\delta}$ as a non-temporal instance containing all the relations over **S** $=$ **R**$\cup\mathcal{T}$ from instant 0 to instant $i+1$. We affirm that for any pure temporal sub-expression α of V and any i, $0 \leq i < n$, we have:

$(*)$ $\{u \mid u$ is an answer of $\alpha^{i+1,i+1}$ on $\hat{\delta}\} = \{u \mid u$ is an answer of $\alpha^{i,i+1}$ on $\hat{\delta}\}$

The left side of (*) represents the set of answers of the non-temporal expression $\alpha^{i+1,i+1}$ on instance $\hat{\delta}$. The right side of (*) represents the set of answers of the non-temporal expression $\alpha^{i,i+1}$ on instance $\hat{\delta}$. However, we notice that the evaluation of $\alpha^{i,i+1}$ on $\hat{\delta}$ uses only the relations of $\hat{\delta}^i$ and the relations of δ^{i+1}.

This can be proved without difficulty, by double induction on the structure of the sub-expression α and on i, using the Table 4.1 The details of this proof is omitted here, since it is a straightforward adaptation of a similar proof in [Chomicki 1995] for the temporal logic counterpart of the algebraic expressions $\alpha^{i,i}$ and $\alpha^{i,i+1}$. As we know [Clifford and Tuzhilin 1990], the past fragment of temporal logic is equivalent to the temporal algebra TA (without aggregate and set-valued operators). The only new features introduced here are the aggregate, pack and unpack operators which do not have equivalent counterparts in the temporal logic described in [Chomicki 1995]. However, as these are *snapshot* operators, *i.e.*, their evaluation at instant $i+1$ does not involve past instants, the induction step for this case is carried out without any difficulty. The proof of the theorem is obtained by induction on the structure of the temporal expression V, using the result (*) in the induction step corresponding to the case where $V = V_1$ **since** V_2 or $V =$ **prev** V_1 or $V = \blacklozenge V_1$ or $V = \blacksquare V_1$. □

4.1.2 Constructing the partial views $\partial\mathcal{V}$

We have just seen that each temporal view of our warehouse, initially specified by an EPTA expression, can be translated into an ERA expression involving only relations in $\mathcal{T}$ and relations in the source databases. However, we want to refine this translation in order to obtain an ERA expression where no source relations appear. We do this by defining the two other types of relations that compose W_m: the partial views and complementary views. In this section we consider only the definition of the partial views.

Considering an ERA expression that defines a non-temporal view V_t, resulting from the translation of a temporal view V, partial views are sub-expressions containing only the join operator in it. They define the parts of V_t we decide to materialize due to two main reasons: (i) complementary views are computed with respect to them and (ii) they compose a rather large kernel which is good for answering many queries. It may be possible to define more "sophisticate" partial views (involving the selection, for instance), however they usually imply a less "uniform" algorithm - one whose goal is to distinguish and treat different kinds of views. We consider that this optimization should be analyzed, but, in this paper, we discuss the maintenance of temporal warehouses more generally and we build a uniform algorithm.

Definition 4.1 - Partial View: Given a database schema **R** and a temporal view V over **R**, let $\mathcal{T}$ be the set of auxiliary relations derived from V. Let V_t be the translation of V into ERA expressions over **R** $\cup\, \mathcal{T}$ (as described in Section 4.1.1). Let $R_1, \ldots, R_s \in$ **R** and $R_{\alpha_1}, \ldots, R_{\alpha_l} \in \mathcal{T}$. A *partial view* of V_t is a sub-expression of V_t of the form

$$\partial V = R_1 \bowtie \ldots \bowtie R_s \bowtie R_{\alpha_1} \bowtie \ldots \bowtie R_{\alpha_l} \quad (5)$$

which is *maximal*, *i.e.*, there is no sub-expression V' of V_t respecting the form (5) and having ∂V as a proper sub-expression. □

Obviously, V_t can contain several partial views $\partial V_1, \dots \partial V_m$. The expression that defines V_t is an ERA expression over these partial views as we can see in the example below.

Running Example 4.2 We consider the view
$V = P_{I:SetProf}(\Pi_C(UNIV \bowtie \blacklozenge EMP) \bowtie PROF)$
of the Running Example 4.1. We have seen that it can be translated into the ERA expression

$$V_t = P_{I:SetProf}(\Pi_C(UNIV \bowtie R_\alpha) \bowtie PROF) \quad (6)$$

V_t contains two partial views: $\partial V_1 = UNIV \bowtie R_\alpha$ and $\partial V_2 = PROF$. Thus, we can rewrite the expression (6) to obtain:

$$V_t = P_{I:SetProf}(\Pi_C \partial V_1 \bowtie \partial V_2). \quad \square \quad (7)$$

4.1.3 Constructing the complement $\mathcal{C}$

We now introduce *complementary views* to assure that the warehouse maintenance is accomplished without querying source databases. Definition 4.1 still uses source relations to specify partial views. In this section, we continue to refine the definition of V_t in order to obtain an expression involving only relations in $\mathcal{T}$ and relations in $\mathcal{C}$ (the set of complementary views for **W**).

Definition 4.2 - Complementary views : Given a set $\mathcal{V}$ of EPTA expressions over a database schema **R**, consider the translation of these expressions into ERA expressions (as described in Section 4.1.1) and let $\partial\mathcal{V}$ be the set of partial views associated to them (according to Definition 4.1). For each $R \in$ **R** we define C_R to be a complementary view of R with respect to $\partial\mathcal{V}$ if C_R is a (non-temporal) view such that sort(C_R) = $sort(R)$ and such that it is possible to compute R from C_R and the partial views in $\partial\mathcal{V}$ (by means of a relational expression). □

Informally, the complementary views C_R of a relation $R \in$ **R** is a view containing the information related to R which is missing in the warehouse. Keeping complementary views of source relations in the warehouse assures its self-maintenance. We would like (if possible) to define complementary views as being views containing the minimum amount of information to achieve self-maintenance. In order to understand what we mean by *minimum*, one notion of view ordering is necessary:

Definition 4.3 - View Ordering [Laurent et al. 2001]: Let U and V be two views over a database schema **R** with $sort(U) = sort(V)$. We say that $U \leq V$ if for every database instance δ, the inclusion $\delta(U) \subseteq \delta(V)$ holds. We say that $U < V$ if $U \leq V$ and there is an instance δ' such that $\delta'(U) \subset \delta'(V)$ and $\delta'(U) \neq \delta'(V)$. This definition is extended to sets of views in the obvious way: If $\mathcal{U} = \{U_1, \dots, U_k\}$ and $\mathcal{V} = \{V_1, \dots, V_k\}$ are sets of views over **R** then $\mathcal{U} \leq \mathcal{V}$ if $U_i \leq V_i$ for each $i \in \{1, \dots, k\}$. □

A complement is minimal if there is no smaller one. Before presenting a method for building complementary views, we should point out a problem we can find during their computation.

In our approach, complementary views are always computed w.r.t. partial views. Partial views are defined by a relational expression containing only the join operator. At a first glance, we could imagine that, due to this fact, the computation presented in [Laurent et al. 2001] could be applied to all source relations. However, in some cases, the join expression corresponding to a partial view can involve auxiliary relations whose translation into ERA expression contains the *union* operator. Unfortunately, the definition of complement introduced in [Laurent et al. 2001] is not valid for views involving union. Thus, in several cases, this definition cannot be applied to compute complementary views of source relations *embedded* in a pure temporal sub-expression of the original temporal view specifying the data warehouse. To illustrate this drawback, we consider Running Example 4.1 and 4.2. We have auxiliary relation R_α associated to the pure temporal sub-expression $\alpha = \blacklozenge EMP$ and partial views ∂V_1 = $UNIV \bowtie R_\alpha$ and ∂V_2 = $PROF$. It is clear, from Table 4.1 that the translation of α involves the union operator. Let us assume that a complementary view for EMP is calculated as in [Laurent et al. 2001]. In that case, C_{EMP} is $EMP \setminus \Pi_{N,J}\partial V_1$ and we are expected to re-calculate the source relation EMP using the relational expression $EMP = C_{EMP} \cup \Pi_{N,J}\partial V_1$. However, this is not possible. Indeed, at instant $i = 1$, the complementary view c^1_{emp} is:
$emp^1 \setminus (\Pi_{N,J}\partial v^1_1) = \{\langle john, waiter\rangle, \langle jane, bookseller\rangle, \langle manuel, teacher\rangle\} \setminus$
$\{\langle john, waiter\rangle, \langle manuel, teacher\rangle, \langle mary, nurse\rangle\} = \{\langle jane, bookseller\rangle\}$.
However, $c^1_{emp} \cup (\Pi_{N,J}\partial v^1_1) = \{\langle jane, bookseller\rangle, \langle john, waiter\rangle, \langle manuel, teacher\rangle, \langle mary, nurse\rangle\}$ and thus $c^1_{emp} \cup (\Pi_{N,J}\partial v^1_1) \neq emp^1$.

We say that relations having this "misbehavior" are *hidden*. The problem behind their "misbehavior" can be analyzed (i) if we consider the complementary view definition of R w.r.t. V, proposed in [Laurent et al. 2001]: $C_R = R \setminus \Pi_{sort(R)}V$ and (ii) if we assume that $V = R \cup S$. Then, in general, we have $R \neq C_R \cup \Pi_{sort(R)}V$ because V may contain tuples of $sort(R)$ that do not belong to R.

In the following, we formally define a hidden relation, illustrating it by an example.

Definition 4.4 - Hidden Relations: Given a set of partial views $\partial\mathcal{V}$, let $\partial V \in \partial\mathcal{V}$ be a partial view whose expression $R_1 \bowtie \ldots \bowtie R_s \bowtie R_{\alpha_1} \bowtie \ldots \bowtie R_{\alpha_l}$ involves a relation $R \in$ **R**. We say that R is a *hidden relation* with respect to ∂V, if *all the following conditions* hold: (i) $R \neq R_i$ for all $1 \leq i \leq s$, (ii) there exists $1 \leq j \leq l$ such that R appears in α_j and (iii) $sort(R) \subseteq sort(\partial V)$. If R is not hidden, we say that R is *normal* with respect to ∂V. □

Running Example 4.3 As in Running Example 4.2, let us consider the temporal view

$$V = P_{I:SetProf}(\Pi_C(UNIV \bowtie \blacklozenge EMP) \bowtie PROF)$$

and its associated partial views ∂V_1 = $UNIV \bowtie R_\alpha$ and ∂V_2 = $PROF$. For each relation, we

consider, firstly, the set of partial views involving them. $UNIV$ and EMP are involved in ∂V_1 (notice that EMP appears in α), while $PROF$ is involved in ∂V_2. From Definition 4.4, we can see that $UNIV$ and $PROF$ are normal relations w.r.t. ∂V_1 and ∂V_2, respectively (none of them respect condition (i) of Definition 4.4). However, EMP is hidden w.r.t. ∂V_1 because: (i) it does not appear explicitly in ∂V_1, (ii) it appears in the expression $\alpha = \blacklozenge EMP$ associated to ∂V_1 and (iii) $sort(EMP) \subseteq sort(\partial V_1)$. □

We slightly change the complement computation proposed in [Laurent et al. 2001] in order to calculate complementary views for "exclusively hidden relations", *i.e.*, relations that are hidden with respect to all partial views they are involved in.

Theorem 4.2 *Given a set $\mathcal{V}$ of EPTA expressions over a database schema **R** and their translation into ERA expressions, let $\partial\mathcal{V}$ be the set of partial views associated to them. For each relation $R \in$ **R** involved in some partial view $\partial V \in \partial\mathcal{V}$, let $V_R = \{\partial V \mid \partial V \in \partial\mathcal{V}$ and R is normal w.r.t. $\partial V\}$. Let C_R be the view defined as follows:*

if $V_R = \emptyset$
  then $C_R = R$ */*Complementary view of a "exclusively hidden relation´´*
  else $C_R = R \setminus \bigcup_{\partial V \in V_R} \Pi_{sort(R)} \partial V$ *(and each relation R is re-computed by*
  $R = C_R \cup \bigcup_{\partial V \in V_R} \Pi_{sort(R)} \partial V)$

Then the view C_R is a complementary view of relation R and it is minimal for source relations for which $V_R \neq \emptyset$.
Proof: For each relation R, the view C_R is its complementary view: Relations for which $V_R = \emptyset$ are re-computed in the obvious way ($R = C_R$) and relations for which $V_R \neq \emptyset$ are re-computed by $R = C_R \cup \bigcup_{\partial V \in V_R} \Pi_{sort(R)} \partial V$. For each relation R such that $V_R \neq \emptyset$ we know that (i) C_R is computed only from the partial views ∂V w.r.t. which R is normal, (ii) a partial view ∂V is defined by an expression containing only join operators and (iii) in [Laurent et al. 2001] it is proved that complementary views of SJ views (in the normal form [Abiteboul et al. 1995]) are minimal. Therefore, we conclude that complementary views of relations for which $V_R \neq \emptyset$ are minimal. □

We refer to [Lechtenborger and Vossen 2003] for a good discussion concerning the problem of determining the minimal complement of a relation R w.r.t. a set of views defined by general algebra relational expressions.

The following example illustrates the computation of complementary views. Given a temporal instance δ over **R**, we denote by c^i_R and ∂v^i, respectively, the evaluation of the complementary view C_R and partial view ∂V over the extended temporal instance $\hat{\delta}$ at instant i.

Running Example 4.4 We consider the same situation described in the Running Example 4.3. According to Theorem 4.2, we have: $C_{EMP} = EMP$ (since EMP is hidden w.r.t. ∂V_1), $C_{UNIV} = UNIV \setminus \Pi_{C,N} \partial V_1$ and $C_{PROF} = PROF \setminus \Pi_{I,C} \partial V_2 = \emptyset$. □

It is interesting to analyze why normal relations w.r.t. at least one partial view are "well-behaved" concerning the computation of complementary views. Suppose that R

is a *normal* relation w.r.t. a partial view $\partial V = R_1 \bowtie \ldots \bowtie R_s \bowtie R_{\alpha_1} \bowtie \ldots \bowtie R_{\alpha_l}$. The complementary view of R is given by $C_R = R \setminus \Pi_{sort(R)} \partial V$. Then, one of the following cases holds:

• R is a relation in the join expression $R_1 \bowtie \ldots \bowtie R_s$. Even if R also appears in one α_i, for $i = 1, \ldots, l$, the view ∂V contains the *intersection* between the result computed by α_i and R. In that way, when we re-compute relation R from its complementary view (via the formula $R = C_R \cup \Pi_{sort(R)} \partial V$) there is no way to introduce in R tuples not coming from R (contrary to what happens to hidden relations).
• R is not a relation that appears explicitly in the expression $R_1 \bowtie \ldots \bowtie R_s$, it appears in one α_i but $sort(R) \not\subseteq sort(\partial V)$. In this case, C_R is always equal to R, since the projection $\Pi_{sort(R)} \partial V = \emptyset$.

The next definition puts together all the concepts introduced so far.

Definition 4.5 - Materialized Component of a Temporal Data Warehouse: Given a database schema **R** and a set of temporal views $\mathcal{V}$ specifying a temporal data warehouse **W**, we define W_m, the *materialized component* of **W**, as a triple $W_m = (\mathcal{T}, \partial\mathcal{V}, \mathcal{C})$ where:
• $\mathcal{T}$ is the finite set of auxiliary relations associated to $\mathcal{V}$ as described in Section 4.1.1 Each relation $R_\alpha \in \mathcal{T}$ corresponds to a pure temporal sub-expression α of a view V, for $V \in \mathcal{V}$.
• $\partial\mathcal{V}$ is the finite set of partial views associated to $\mathcal{V}$. Each view $\partial V \in \partial\mathcal{V}$ is given by Definition 4.1.
• $\mathcal{C}$ is the set of complementary views w.r.t. partial views in $\partial\mathcal{V}$ computed as shown in Theorem 4.2. □

We finish this section by stressing the importance of the materialized component, *i.e.*, the importance of storing auxiliary views in the warehouse. In fact, the materialized component allows not only the reduction of the temporal setting to a non-temporal one but also the warehouse self-maintenance. Besides, the materialized component gives a flexibility to our warehouse, since it represents a kernel over which ad-hoc queries can be formulated, which resembles the concept of query-independent concept discussed in [Laurent et al. 2001].

4.2 Defining the virtual component W_v

In the previous sections we have defined the three kinds of views that compose the materialized component of a temporal data warehouse **W**: auxiliary relations, partial views and complementary views. In this section, we shall use the partial views and the ERA expressions corresponding to the translations of the the EPTA expressions, which specify the temporal data warehouse **W** (as discussed in Section 4.1.1), to build the virtual component W_v. We call W_v a *virtual* component because it represents views that do not need to be materialized - they can be computed by ERA expressions over the materialized relations in W_m. However, it is important to emphasize that a virtual component,

despite its name, may be materialized, depending on the cost of its calculation. This dichotomy between materialized and virtual components aims at giving more flexibility concerning the storage and representation of the different types of information composing a temporal data warehouse. We refer to [Gupta and Mumick 2004] for a theoretical formulation of the general view-selection problem in a warehouse.

Definition 4.6 - Virtual Component of a Temporal Data Warehouse: Let $\mathcal{V}$ be a set of EPTA expressions over a database schema **R** and consider its translation into ERA expressions, as discussed in Section 4.1.1. We define W_v, the *virtual component* of **W**, as the set of views V resulting from this translation and respecting the following grammar:

$$V ::= \partial V \mid \beta(V) \mid V \gamma V$$

where ∂V is a partial view (Definition 4.1), β is an unary ERA operator and γ is a binary operator of the relational algebra. □

Running Example 4.5 The materialized and virtual components of the warehouse in our running example are given by: $W_m = (\{R_\alpha\}, \{\partial V_1, \partial V_2\}, \{C_{EMP}, C_{UNIV}, C_{PROF}\})$ and $W_v = \{P_{I:SetProf}(\Pi_C \partial V_1) \bowtie \partial V_2\}$ □

As we said in the beginning of this section, given a temporal instance $\delta = (\delta^0, \ldots, \delta^n)$, there is a correspondence between the evolution of the database and the evolution of the warehouse $w = (w^0, \ldots, w^n)$: w^i is the warehouse corresponding to δ^i , for each $i = 0, \ldots, n$. It is important to notice that the contents of w^i corresponds exactly to the contents of the virtual views in W_v at instant i, which depends essentially on the contents of the partial views in $\partial\mathcal{V}$.

In the next section, we will describe how a current state w^i of the data warehouse is maintained, when δ^i is updated. We will see that the new state w^{i+1} is built taking into account: (i) the partial views ∂v^i, (ii) the complementary views c^i_R of each relation $R \in$ **R**, (iii) the auxiliary relations r^i_α and (iv) the updates over the source relations $R \in$ **R**.

5 Maintaining the Temporal Data Warehouse

Given a temporal data warehouse **W** $= (W_m, W_v)$, let $\delta = (\delta^0, \ldots, \delta^n)$ be a temporal database over the database schema **R** and $w = (w^0, \ldots, w^i)$ the corresponding temporal instances of **W**. In response to a set of updates u applied to the instance δ^i to obtain δ^{i+1}, the current warehouse state w^i is modified to produce w^{i+1}. We denote by w^i_m the set of materialized views at instant i. The following algorithm computes w^{i+1}_m from u and w^i_m. From w^{i+1}_m it is possible to calculate w^{i+1}_v (and so w^{i+1}) since the virtual views in W_v depend only on the contents of the partial views, which are stored in the data warehouse.

In the following we present the algorithm responsible for the maintenance of all three kinds of relations composing the materialized component $W_m = (\mathcal{T}, \partial\mathcal{V}, \mathcal{C})$ and we explain the procedures used in it.

Algorithm 5.1 - Maintenance Algorithm
Input: (u, w_m^i) where $u = \{\triangle r^i \mid R \in \mathbf{R}\} \cup \{\triangledown r^i \mid R \in \mathbf{R}\}$ and w_m^i is the materialized component at instant i
Output: w_m^{i+1}
Main procedure:
`for` *each auxiliary relation* R_α *in* $\mathcal{T}$ `do` $FindChangeAuxRel(\triangle r_\alpha^i, \triangledown r_\alpha^i)$;
/*Compute changes over each auxiliary relation R_α
`for` *each each partial view* $\partial V \in \partial\mathcal{V}$ `do` $FindChangePartialView(\triangle(\partial v^i), \triangledown(\partial v^i))$;
/*Compute changes over each partial view $\partial V \in \partial\mathcal{V}$
`for` *each relation* R *in* $\mathbf{R}$ `do` $ComputeComplement(c_R^{i+1})$;
/*Compute new complementary views of the source relations
`for` *each each partial view* $\partial V \in \partial\mathcal{V}$ `do` $ComputePartialView(\partial v^{i+1})$;
/*Compute new partial views
`for` *each auxiliary relation* $R_\alpha \in \mathcal{T}$ `do` $ComputeAuxRel(r_\alpha^{i+1})$;
/*Compute new auxiliary relations □

We consider the procedures used in Algorithm 5.1. Firstly we recall that ERA expressions without parenthesis are evaluated from left to right. Secondly, we introduce, for each $R \in \mathbf{R}$, a relation $\overset{*}{R}$ defined below. To indicate that $\overset{*}{R}$ is computed over join view instances at instant i (resp. $i+1$), we write $\overset{*}{r}{}^i$ (resp. $\overset{*}{r}{}^{i+1}$).

`for` each $R \in \mathbf{R}$ involved in some partial view $\partial V \in \partial\mathcal{V}$ `do`
Let $V_R = \{\partial V \mid R$ is normal w.r.t. $\partial V\}$
`if` $V_R = \emptyset$ `then` $\overset{*}{R}$ is empty $/ *$ R is hidden w.r.t. all partial views it is involved in*/
`else` $\overset{*}{R} = \bigcup_{\partial V \in V_R} \Pi_{sort(R)} \partial V$,

Procedures used in Algorithm 5.1:

1. $FindChangeAuxRel(\triangle r_\alpha^i, \triangledown r_\alpha^i)$
At instant i, compute the update applied to *each* auxiliary relation R_α: $\triangle r_\alpha^i = r_\alpha^{i+1} \setminus r_\alpha^i$ and $\triangledown r_\alpha^i = r_\alpha^i \setminus r_\alpha^{i+1}$
To compute r_α^{i+1}, use Table 4.1 to find the relational algebra query $\alpha^{i,i+1}$. Then, for all source relation $R \in \mathbf{R}$ appearing in $\alpha^{i,i+1}$ replace instance r^i by $[\ c_R^i \cup \overset{*}{r}{}^i\ ]$ and r^{i+1} by $[\ c_R^i \cup \overset{*}{r}{}^i \setminus \triangledown r^i \cup \triangle r^i\ ]$
Remark: The computation of r_α^{i+1} in two steps, (*i.e.*, by using *FindChangeAuxRel* before *ComputeAuxRel*) is explained by the fact that an auxiliary relation can correspond to a temporal expression α_2 that involves another temporal expression α_1. Thus, to find $r_{\alpha_2}^{i+1}$ we may need to know the value of $r_{\alpha_1}^i$. For this reason we defer the "update" of auxiliary relations.
2. $FindChangePartialView(\triangle(\partial v^i), \triangledown(\partial v^i))$
At instant i, compute the modifications applied to *each* partial view $\partial V = R_1 \bowtie \ldots \bowtie R_s \bowtie R_{\alpha_1} \bowtie \ldots \bowtie R_{\alpha_l}$:
$\triangle(\partial v^i) = [\ (\bowtie_{1 \le k \le s}(\ c_{R_k}^i \cup \overset{*}{r}{}_k^i \setminus \triangledown r_k^i \cup \triangle r_k^i)) \bowtie (\bowtie_{1 \le h \le l}(r_{\alpha_h}^i \setminus \triangledown r_{\alpha_h}^i \cup \triangle r_{\alpha_h}^i))\ ] \setminus \partial v^i$
$\triangledown(\partial v^i) = \partial v^i \setminus [\ (\bowtie_{1 \le k \le s}(\ c_{R_k}^i \cup \overset{*}{r}{}_k^i \setminus \triangledown r_k^i \cup \triangle r_k^i)) \bowtie (\ \bowtie_{1 \le h \le l}(r_{\alpha_h}^i \setminus \triangledown r_{\alpha_h}^i \cup \triangle r_{\alpha_h}^i))\ ]$

3. $ComputeComplement(c_R^{i+1})$
Compute complement instances at instant $i+1$ from complement at instant i: At instant $i = 0$ complementary views are computed using source relations (Theorem 4.2) and at instant $i \geq 1$ we have $c_R^{i+1} = [c_R^i \cup \overset{*}{r}{}^i \setminus \bigtriangledown r^i \cup \bigtriangleup r^i] \;\setminus\; \overset{*}{r}{}^{i+1}$
4. $ComputePartialView(\partial v^{i+1})$: $\partial v^{i+1} = \partial v^i \setminus \bigtriangledown(\partial v^i) \cup \bigtriangleup(\partial v^i)$
5. $ComputeAuxRel(r_\alpha^{i+1})$: $r_\alpha^{i+1} = r_\alpha^i \setminus \bigtriangledown r_\alpha^i \cup \bigtriangleup r_\alpha^i$

The following theorem shows that Algorithm 5.1 correctly maintains the materialized views stored in W_m.

Theorem 5.1 *Given a temporal database $\delta = (\delta^0, \ldots, \delta^i)$ and its associated temporal warehouse $w = (w^0, \ldots, w^i)$, let u be the set of updates that we apply to the instance δ^i to obtain instance δ^{i+1}. Let w^{i+1} be the temporal data warehouse obtained by evaluating the EPTA expressions which specify it, over $(\delta^0, \ldots, \delta^i, \delta^{i+1})$ at instant $i+1$. On the other hand, let $\overline{w}^{i+1}$ be the result of evaluating the virtual views in w_v^{i+1}, where the partial views ∂v^{i+1} occurring in them are calculated by applying Algorithm 5.1 on w_m^i. Then $w^{i+1} = \overline{w}^{i+1}$.*

Proof: We want to prove that the virtual views in w_v^{i+1}, computed by evaluating ERA expressions over the partial views in w_m^{i+1} correspond to the virtual views we obtain by evaluating the original EPTA expressions over the temporal database instance at instant $i+1$. To this end, we have to prove that Algorithm 5.1 correctly computes the materialized views stored in w_m^{i+1} from w_m^i and u.

Firstly, we prove the correctness of the procedures *FindChangeAuxRel* and *ComputeAuxRel*. The goal of the procedure *FindChangeAuxRel* is to compute the changes $\bigtriangleup r_\alpha^i$ and $\bigtriangledown r_\alpha^i$ over auxiliary relations due to updates on the source relations. The procedure *ComputeAuxRel* just applies these changes to r_α^i. In fact, the most important point is the computation of the auxiliary relation r_α^{i+1} in the procedure *FindChangeAuxRel* by using Table 4.1. Due to the recursiveness of this computation, we cannot at this point update the relation r_α^i by replacing it by r_α^{i+1}, because the old state may be necessary in later induction steps to calculate r_β^{i+1}, if α is a sub-expression of β. So, the updates of the auxiliary relations are deferred to the final procedure *ComputeAuxRel*. More precisely, the computation of r_α^{i+1} in *FindChangeAuxRel*, corresponds to the evaluation of an ERA expression given by Table 4.1 involving the previous instance r_α^i (available in w_m^i) and source instances at instants i and $i+1$. By Theorem 4.2, we know that each source relation r^i (resp. r^{i+1}) appearing in the ERA expression can be computed by using the complement available in w_m^i, that is, by computing the expression $c_R^i \cup \overset{*}{r}{}^i$ (resp. $c_R^i \cup \overset{*}{r}{}^i \setminus \bigtriangledown r^i \cup \bigtriangleup r^i$).

Secondly, we prove the correctness of the procedures *FindChangePartialView* and *ComputePartialView*. The procedure *FindChangePartialView* aims at computing the changes $\bigtriangleup\partial v^i$ and $\bigtriangledown\partial v^i$ over partial views consequent upon the updating on source relations. The procedure $ComputePartialView$ just applies these changes to ∂v^i. We know that

$\triangle\partial v^i = \partial v^{i+1} \setminus \partial v^i$ and $\bigtriangledown\partial v^i = \partial v^i \setminus \partial v^{i+1}$. Moreover, ∂v^i is one of the materialized views stored in w^i_m. According to Definition 4.1, a partial view at instant $i + 1$ is defined by the following join expression:

$$\partial v^{i+1} = (r_1^{i+1} \bowtie \ldots \bowtie r_s^{i+1}) \bowtie (r_{\alpha_1}^{i+1} \bowtie \ldots \bowtie r_{\alpha_l}^{i+1})$$

For each instance r^i_k $(1 \leq k \leq s)$ of a source relation, the new instance r^{i+1}_k is obtained by applying the corresponding updates on r^i_k, that is, $r^{i+1}_k = r^i_k \setminus \bigtriangledown r^i_k \cup \triangle r^i_k$. We remind that $\triangle r^i_k \cap \bigtriangledown r^i_k = \emptyset$ for any source relation R_k. Theorem 4.2 assures that we can replace r^i_k by $c^i_{R_k} \cup \overset{*}{r}{}^i_k$ to obtain $r^{i+1}_k = c^i_{R_k} \cup \overset{*}{r}{}^i_k \setminus \bigtriangledown r^i_k \cup \triangle r^i_k$. Thus, to compute r^{i+1}_k we need to know the values of: (i) the complementary view $c^i_{R_k}$, available in w^i_m and (ii) $\overset{*}{r}{}^i_k$, computed from the partial view ∂v^i which is stored in w^i_m. The auxiliary relations $r^{i+1}_{\alpha_h}$ $(1 \leq h \leq l)$ are computed by using the result of *FindChangeAuxRel* (that uses Table 4.1).

The proof of the correctness of procedure $ComputeComplement$ is straightforward (just an application of Theorem 4.2). Theorem 4.1 assures that the virtual views obtained by evaluating an ERA expression over a partial view $\partial v^{i+1} \in w^{i+1}_m$, correspond to the virtual views obtained by the evaluation of the original EPTA expressions over the temporal database instance at instant $i + 1$. □

We illustrate the execution of Algorithm 5.1 by considering an update over sources of our running example. We refer to [Amo and Halfeld-Ferrari 2002] for examples over warehouses whose specification includes set-valued operators, aggregate functions and the union operator.

Running Example 5.1 We consider the temporal data warehouse **W** of our running example and the temporal instance presented in the Running Example 4.1. Following the explanations given so far we can verify that, at instant $i = 2$ the materialized component of **W** contains the following materialized views:

r^2_α	$\{\langle manuel, teacher\rangle, \langle john, waiter\rangle, \langle mary, nurse\rangle, \langle jane, bookseller\rangle\}$
∂v^2_1	$\{\langle cs, manuel, teacher\rangle, \langle cs, john, waiter\rangle\}$
∂v^2_2	$\{\langle cs, pr1\rangle, \langle cs, pr2\rangle, \langle math, pr2\rangle, \langle law, pr3\rangle, \langle law, pr4\rangle\}$
c^2_{emp}	$\{\langle paul, clerk\rangle, \langle jane, bookseller\rangle\}$
c^2_{univ}	$\emptyset$
c^2_{prof}	$\emptyset$

Now we suppose that, at instant $i = 2$, the Algorithm 5.1 is executed, having as input the set of materialized views w^2_m given above and a set of updates u, composed of the following updates:

– On EMP: $\triangle emp^2 = \{\langle charles, teacher\rangle\}$ and $\bigtriangledown emp^2 = \emptyset$
– On $UNIV$: $\triangle univ^2 = \{\langle biology, mary\rangle, \langle math, joe\rangle\}$ and $\bigtriangledown univ^2 = \{\langle cs, manuel\rangle\}$
– On $PROF$: $\triangle prof^2 = \emptyset$ and $\bigtriangledown prof^2 = \{\langle math, pr2\rangle, \langle law, pr3\rangle, \langle law, pr4\rangle\}$

The result obtained after the execution of each procedure is the following

– The procedure $FindChangeAuxRel$ returns :
$\triangle r^2_\alpha = \{\langle paul, clerk\rangle\}$ and $\bigtriangledown r^2_\alpha = \emptyset$.
– The procedure $FindChangePartialView$ returns :
$\triangle\partial v^2_1 = \{\langle biology, mary, nurse\rangle\}$ and $\bigtriangledown\partial v^2_1 = \{\langle cs, manuel, teacher\rangle\}$
$\triangle\partial v^2_2 = \emptyset$ and $\bigtriangledown\partial v^2_2 = \{\langle math, pr2\rangle, \langle law, pr3\rangle, \langle law, pr4\rangle\}$.

- The procedure $ComputeComplement$ returns :
  $c^3_{emp} = \{\langle jane, bookseller\rangle, \langle paul, clerk\rangle, \langle charles, teacher\rangle\}$
  $c^3_{emp} = \{math, joe\}$ and
  $c^3_{prof} = \emptyset$
- The procedure $ComputePartialView$ returns :
  $\partial v^3_1 = \{\langle cs, john, waiter\rangle, \langle biology, mary, nurse\rangle\}$
  $\partial v^3_2 = \{\langle cs, pr1\rangle, \langle cs, pr2\rangle\}$
- The procedure $ComputeAuxRel$ returns :
  $r^3_\alpha = \{\langle manuel, teacher\rangle, \langle john, waiter\rangle, \langle mary, nurse\rangle, \langle jane, bookseller\rangle, \langle paul, clerk\rangle\}$□

From the above example, it is clear that the partial views ∂v^3_1 and ∂v^3_2 computed by the Algorithm 5.1 verifies $\partial v^3_1 = univ^3 \bowtie r^3_\alpha$ and $\partial v^3_2 = prof^3$ and that the answer of the EPTA expression V at instant 3 is exactly w^3_v, as it was expected, due to the correctness of Algorithm 5.1. Notice that our approach is independent of the particular way complements are computed. If another kind of computation (respecting Definition 4.2) is used we just need to adapt some procedures of Algorithm 5.1 (to prove the correctness of such a new algorithm we would use the same arguments used in this paper).

We emphasize here that to know the contents of our warehouse at an instant i, we just need to evaluate ERA expressions over the partial views. The auxiliary views in $\mathcal{T}$ and the complementary views in $\mathcal{C}$ are important only during the maintenance step, (*i.e.*, to compute the new partial views $\partial\mathcal{V}$ at instant $i+1$). Thus, the set of partial views represents the kernel of the materialized component W_m.

6 Related Work

Temporal view maintenance is considered in papers such as [Baekgaard and Mark 1995, Jagadish et al. 1995, Yang and Widom 2000]. In [Yang and Widom 2000] the underlying model is *time-explicit* and the language specifying the temporal views allows past and future operators (both the temporal model and temporal query language are based on [Bohlen et al. 1995]) and *retroactive updates*. Their approach is closely related to ours, but aggregate function and set-valued relations are not considered there. Our major contribution with respect to [Yang and Widom 2000] is that our temporal views can be expressed by first order expressions over non-temporal relations.

The paper [Jagadish et al. 1995] addresses the view maintenance problem in the *chronicle model*. Temporal views are *snapshot-reducible* [Bohlen et al. 1995], *i.e.*, they are defined by temporal operators which correspond very closely to their non-temporal counterparts, which is not the case, concerning some EPTA operators. An incremental algorithm is developed in [Baekgaard and Mark 1995] to treat the temporal view self-maintenance problem for time-varying selections. More precisely, they consider time-varying queries in which selection predicates refer to the state of a clock and whose results may change just because time passes. In our approach, time is treated in an abstract context, and can be interpreted in different ways. For instance, it could refer to *valid-time*, the time when the information was satisfied in the real-world, or *transaction-time*, the time when the information was actually entered in the database.

The problem of maintaining temporal aggregate views is treated in some recent work [Yang and Widom 2001, Zhang et al. 2001]. Both papers consider implementation aspects concerning the computation of temporal aggregates in a time-explicit underlying model. To deal with the computation of temporal aggregates and the incremental maintenance of temporal aggregate views, a new kind of index structure, called SB-tree, is introduced in [Yang and Widom 2001]. This structure contains enough information to construct the contents of temporal aggregates that it indexes. In our approach, the underlying model is time-implicit and thus we do not deal with the problem addressed in [Yang and Widom 2001, Zhang et al. 2001]. In fact, in their approaches a database tuple is accompanied by a time interval during which its attribute values are valid. Consequently, the value of a tuple attribute affects the aggregate computation for all those instants included in the tuple's time interval. In our work, the computation of temporal aggregates is similar to its non-temporal counterpart. An aggregate function applied on a relation Q assembles all tuples belonging to Q at instant i and having the same values for a set of attributes X. Then, it executes an operation (such as sum, avg, etc) over a specified column of the resulting set of tuples.

In spite of important results obtained in the research area of incremental view maintenance [Blakeley et al. 1986, Gupta et al.1993, Griffin and Libbin 1995] the maintenance of materialized views in a warehouse environment represents a difficult problem [Agrawal et al. 1997, Laurent et al. 2001, Quass et al. 1996, Zhuge et al. 1995]. The notion of view self-maintainability has attracted the attention of several authors (see, for instance [Laurent et al. 2001, Mohania and Kambayashi 2000, Quass et al. 1996]). In [Laurent et al. 2001] the authors consider the problem of maintaining non-temporal SPJ views in warehouses and propose a method where, by adding complements in the warehouse, it is possible to re-compute all source relations whenever necessary. They develop this idea and generalize self-maintainability to query and update independence.

In [Mohania and Kambayashi 2000], the maintenance of non-temporal aggregate views is considered. To allow self-maintenance, auxiliary relations are stored in the warehouse. The authors propose a method based on the analysis of the optimized tree expression corresponding to each view. Firstly they compute the auxiliary relations (one for each node) necessary to make a view self-maintainable. Secondly, they consider the problem of updates to nodes, which are propagated in a bottom-up fashion: the update to each node in the tree is derived from the updates to its children and the auxiliary relations materialized for the children and the node itself. The self-maintainability of the view is achieved by materializing those base relations which are children of a join node in the view expression tree.

In our approach, different kinds of auxiliary relations are kept in the warehouse not only to assure self-maintenance but also to allow the construction of temporal views without storing the entire history of the sources. To assure self-maintenance, we have two types of materialized views, namely, the complements and the partial

views. View complements have been introduced in [Bancilhon and Spyratos 1981]. In [Lechtenborger 2003] we find an interesting discussion on its impact over view updating. [Lechtenborger and Vossen 2003] shows minimality results concerning complements for relational views w.r.t. information content. The authors argue that it may not be necessary to look for minimal complements in practice.

The idea of using complements in a non-temporal data warehouse environment has been explored in [Laurent et al. 2001] but in our paper we extend this notion to a temporal framework, where we also deal with aggregate functions and set-valued attributes. The partial views are join views used not only to compute complements but also to calculate the pre-defined views that specify a warehouse. In this way, we do not follow the idea of [Mohania and Kambayashi 2000] and instead of storing base relations taking part into a join, we store join views. As in [Mohania and Kambayashi 2000], we can argue that partial views represent a flexibility of our approach, since they can be used as the kernel of potential ad-hoc queries.

Our method is based on the techniques introduced in [Chomicki 1995]. It generalizes the work proposed in [Amo and Halfeld-Ferrari 2000] where the relational algebra expression defining V_t involves only one partial view and is specified by a special relational algebra expression containing only the selection, the projection and the join operator. In the present paper, we treat general expressions which may contain several partial views and any relational algebraic operator. We would like to highlight that the restriction to set-valued attributes, instead of treating general complex-typed attributes [Abiteboul et al. 1995], is a choice aiming at simplifying the presentation. Our approach can be adapted to a more general context where the underlying data model allows complex-typed attributes.

7 Conclusions

We have proposed an incremental method for maintaining a temporal data warehouse originating from multiple, autonomous, heterogeneous and non-temporal sources. Our approach combines in a uniform way two previously unrelated techniques based on auxiliary views. The materialization of auxiliary relations is used successfully not only to reduce the temporal setting to a non-temporal one but also to get around the decoupling of non-temporal data sources and temporal data warehouse (which can cause update anomalies). In our method, there is no need to store the entire history of source databases and the warehouse maintenance is performed without consulting the sources. Although we have only presented the theoretical aspects of our approach, its importance can be justified by the fact that in a warehouse environment the cost of recomputing views from scratch is high since it involves consulting different sites, and thus implies a large overhead in terms of communication cost.

The computation of complementary views used in this paper derives directly from the one introduced in [Laurent et al. 2001]. We notice that an interesting research topic consists in analyzing the application of other kinds of complementary view computation to our context. In particular, some ideas proposed in [Lechtenborger and Vossen 2003]

(such as the non-minimal super-key based complements) may result in improvements to our approach. Moreover, in this paper we have only considered the algebraic counterpart of the past temporal logic fragment. Although we have not treated the future operators **Until** and **Next**, it would be interesting to investigate such an extension, in order to generalize the retroactive updates of [Yang and Widom 2000].

Acknowledgment

We would like to thank the anonymous referees whose really careful reading, useful comments and corrections have lead to significant improvement of our work.

References

[Abiteboul and Bidoit 1984] S. Abiteboul and N. Bidoit. Non first normal form relations to represent hierarchical organized data. In *ACM Symposium on Principles Database Systems*, 191–200, 1984.

[Abiteboul et al. 1996] S. Abiteboul, L. Herr, and J. Van den Bussche. Temporal versus first-order logic to query temporal databases. In *ACM Symposium on Principles of Database System*, 49–57, 1996.

[Abiteboul et al. 1995] S. Abiteboul, R. Hull, and V. Vianu. *Foundations of Databases*. Addison-Wesley Publishing Company, 1995.

[Agrawal et al. 1997] D. Agrawal, A. El Abbadi, A. Singh, and T. Yurek. Efficient view maintenance at data warehouses. In *Proceedings of the ACM-SIGMOD International Conference on Mangement of Data*, 417–427, 1997.

[Amo and Halfeld-Ferrari 2000] S. de Amo and M. Halfeld Ferrari Alves. Efficient maintenance of temporal data warehouses. In *International Database Engeneering and Applications Symposium (IDEAS)*, Japan, 188-196, September 2000.

[Amo and Halfeld-Ferrari 2002] S. de Amo and M. Halfeld Ferrari Alves. Self-maintainable temporal data wahehouses. Technical Report 258, LI/Université François Rabelais Blois - Tours - Chinon, 2002.

[Baekgaard and Mark 1995] L. Baekgaard and L. Mark. Incremental computation of time-varying query expressions. *IEEE Trans. on Knowledge and Data Engineering*, 7(4):583–590, 1995.

[Bancilhon and Spyratos 1981] F. Bancilhon and N. Spyratos. Update semantics of relational views. *ACM Transactions on Database Systems*, 6(4), 398–408, 1981.

[Blakeley et al. 1986] J. A. Blakeley, B. A. Larson, and F. W. Tompa. Efficiently updating materialized views. In *Proceedings of the ACM-SIGMOD International Conference on Mangement of Data*, 61 – 71, 1986.

[Bohlen et al. 1995] M. H. Bohlen, C. S. Jensen, and R. T. Snodgras. Evaluating the completeness of TSQL2. In *Proc. of the Int. Workshop on Temporal Databases*, 153–172, 1995.

[Chomicki 1994] J. Chomicki. Temporal query languages: A survey. In Springer-Verlag, editor, *Temporal Logic, First International Conference*, number 827 in Lecture Notes in Artificial Intelligence (LNAI), 506–534, 1994.

[Chomicki 1995] J. Chomicki. Efficient checking of temporal integrity constraints using bounded history encoding. *ACM Transactions on Database Systems*, 20(2), 149–186, 1995.

[Clifford and Tuzhilin 1990] J. Clifford and A. Tuzhilin. A temporal relational algebra as a basis for temporal completeness. In *Proceedings of Very Large Data Bases*, 13–23, 1990.

[Emerson 1990] E.A. Emerson. Temporal and Modal Logic. In *Handbook of Theoretical Computer Science*, Volume B: Formal Models and Semantics, J.van Leeuwen, ed., 995–1072, Elsevier Science Publishers, 1990.

[Gradel 1999] E. Gradel. Why are modal logics so robustly decidable? In *Bulletin of the European Association of Theoretical Computer Science*, number 68, 90–103, 1999.

[Griffin and Libbin 1995] T. Griffin and L. Libbin. Incremental maintenance of views with duplicates. In *Proceedings of the ACM-SIGMOD International Conference on Mangement of Data*, 328–339, 1995.

[Gupta et al.1993] A. Gupta, I. S. Mamick, and V. S. Subramanian. Maintaining views incrementally. In *Proceedings of the ACM-SIGMOD International Conference on Mangement of Data*, 157 – 166, 1993.

[Gupta and Mumick 2004] H. Gupta and I. Mumick. Selection of views to materialize in a data warehouse. *Transactions of Knowledge and Data Engineering (TKDE)*, (To appear), 2004.

[Jaeschke and Schek 1982] G. Jaeschke and H. J Schek. Remarks on the algebra on non first normal form relations. In *1st ACM Symposium on Principles of Database System*, 124–138, 1982.

[Jagadish et al. 1995] H. V. Jagadish, I. S. Mumick, and A. Silberschatz. View maintenance issues for the chronicle data model. In *ACM Symposium on Principles of Database System*, 113–124, 1995.

[Klug 1982] A. Klug. Equivalence of relational algebra and relational calculus query languages having aggregate funcitions. *Journal of ACM*, 29(3), 699–717, 1982.

[Laurent et al. 2001] D. Laurent, J. Lechtenborger, N. Spyratos, and G. Vossen. Monotonic complements for independent data warehouses. *The VLDB Journal*, 10(4), 295–315, Springer-Verlag, 2001.

[Lechtenborger 2003] J. Lechtenborger. The impact of constant complement approach towards view updating. In *22th ACM Symposium on Principles of Database System*, 49–55, 2003.

[Lechtenborger and Vossen 2003] J. Lechtenborger and G. Vossen. On the computation of relational view complements. *ACM Transactions on Database Systems*, 28(2), 175–208, 2003.

[Mckenzie and Snodgrass 1991] L. E. Mckenzie, R. T. Snodgrass. Evaluation of Relational Algebras Incorporating the Time Dimension in Databases. In *ACM Computing Surveys*, Vol. 23, No. 4, 501–543, December 1991.

[Mohania and Kambayashi 2000] M. Mohania and Y. Kambayashi. Making aggregate views self-maintainable. *Journal of Data and Knowledge Engineering*, 32(1), 87–109, Elsevier, 2000.

[Niwinsky and Toman 1996] D. Niwinsky and D. Toman. First-order queries over temporal databases inexpressible in temporal logic. In *Proceedings of the 5th International Conference on Extending Database Technology (EDBT)*, 307–324, 1996.

[Ozoyoglu et al. 1987] G. Ozoyoglu, Z. M. Ozoyoglu, and V. Matos. Extending relational algebra and relational calculus with set-valued attributes and aggregate functions. *ACM Transactions on Databases Systems*, 12(4), 566–592, 1987.

[Quass et al. 1996] D. Quass, A. Gupta, I. S. Mumick, and J. Widom. Making views self-maintainable for data warehousing. In *Proceedings of the 4th International Conference on Parallel and Distributed Information System*, 158–169, 1996.

[Sistla and Clarke 1985] A. P. Sistla and E. M. Clarke. The complexity of propositional linear temporal logic. *Journal of ACM*, 32(3), 733–749, 1985.

[Stockmeyer 1974] L. J. Stockmeyer. *The complexity of decision problem in automata theory and logic*. PhD thesis, Department of Electrical Engineering, MIT, 1974.

[Yang and Widom 2000] J. Yang and J. Widom. Temporal view self-maintenance. In *Proceedings of the International Conference on Extending Database Technology (EDBT)*, 395–412, 2000.

[Yang and Widom 2001] J. Yang and J. Widom. Incremental computation and maintenance of temporal aggregates. In *Proceedings of the 17th International Conference on Data Engineering*, Heidelberg, Germany, 51–60, 2001.

[Zhang et al. 2001] D. Zhang, D. Gunopulos, A. Markowetz, B. Seeger, and V. Tsotras. Efficient computation of temporal aggregates with range predicates. In *20th ACM Symposium on Principles of Database System*, 237–245, 2001.

[Zhuge et al. 1995] Y. Zhuge, H. Garcia-Molina, J. Hammer, and J. Widom. View maintenance in a data warehousing environment. In *Proceedings of the ACM SIGMOD International Conference on Management of Data*, 316–327, 1995.
