# Supplementary material for: In-text citation’s frequencies-based recommendations of relevant research papers
Source: PeerJ Comput Sci. 2021 Jun 4;7:e524. doi: 10.7717/peerj-cs.524 (PMC8189020; doi:10.7717/peerj-cs.524)
Supplement: Supplemental Information 2 — These articles are available under a CC BY-ND 4.0 license. [file peerj-cs-07-524-s002.zip › Dataset/Papers Dataset.zip!/Papers Dataset/jucs_248.pdf]

Journal of Universal Computer Science, vol. 9, no. 3 (2003), 248-269
submitted: 1/10/02, accepted: 13/3/03, appeared: 28/3/03 *© J.UCS*

An Automatic Verification Technique for Loop and Data Reuse Transformations based on Geometric Modeling of Programs

K.C. Shashidhar
(DESICS Division, IMEC vzw, Kapeldreef 75, B-3001 Heverlee, and
Department of Computer Science, Katholieke Universiteit Leuven, Belgium
kodambal@imec.be)

Maurice Bruynooghe
(Department of Computer Science, Katholieke Universiteit Leuven, Belgium
maurice@cs.kuleuven.ac.be)

Francky Catthoor
(DESICS Division, IMEC vzw, Kapeldreef 75, B-3001 Heverlee, and
Department of ESAT, Katholieke Universiteit Leuven, Belgium
catthoor@imec.be)

Gerda Janssens
(Department of Computer Science, Katholieke Universiteit Leuven, Belgium
gerda@cs.kuleuven.ac.be)

Abstract: Optimizing programs by applying source-to-source transformations is a prevalent practice among programmers. Particularly so, while programming for high-performance and cost-effective embedded systems, where the initial program is subject to a series of transformations to optimize computation and communication. In the context of parallelization and custom memory design, such transformations are applied on the loop structures and index expressions of array variables in the program, more often manually than with a tool, leading to the non-trivial problem of checking their correctness. Applied transformations are *semantics preserving* if the transformed program is *functionally equivalent* to the initial program from the input-output point of view. In this work we present an automatic technique based on geometric modeling to formally check the functional equivalence of initial and transformed programs under loop and data reuse transformations. The verification is *transformation oblivious* needing no information either about the particular transformations that have been applied or the order in which they have been applied. Our technique also provides useful diagnostics to locate the detected errors.

Key Words: Transformation Verification, Program Equivalence, Program Transformations, Restructuring Compilers, Geometric Modeling

Category: D.2.4, F.3.1, D.3.4, I.2.2

1 Introduction

The design of embedded systems for the consumer electronics market, particularly for multimedia signal processing applications, is a complex task. The demands on the optimality of design of these systems in terms of performance, area, power and cost are high. Typically, an initial design is assembled by a straightforward implementation of the specification in a high-level programming language. This initial design, if naively implemented, often leads to an unacceptably sub-optimal system. This has motivated development of frameworks for systematic design of embedded systems. The frameworks call for design exploration and optimization at different levels of abstractions, to arrive at a mapping of the software onto the custom made platform, which is closer to the optimal implementation. An important design rule is that optimizations applied at higher abstraction levels offer greater gains. Hence the initial source code, called the *executable specification*, is the starting point for a systematic exploration which subjects it to source-to-source transformations. For example, in the context of parallelization of programs [Banerjee 1994, Wolf and Lam 1991] and compiling programs for systems with custom-made memories [Catthoor et al. 1998], transformations that modify the loop structure of the program (loop transformations) [Banerjee 1993] and/or introduce caches to reduce the cost of data transfers (data reuse transformations) are very common, since they can lead to significant improvements. Such global transformations are still not within the scope of the current optimizing compilers [Goos 2001]; they are applied at the source level in a pre-compilation phase.

Within the realm of designing embedded systems for multimedia signal processing applications, it is a widespread practice [Catthoor et al. 1998] to start from a specification at the level of C-code. By means of some preparatory simple transformations the code is brought into a form where complex global transformations aiming at optimizing data transfer and storage can be applied. Presently, the designers are themselves controlling the application of these complex transformations. Some code transformation tools exist (for example, [Samsom et al. 1993]), but they offer insufficient flexibility. Often the experienced designers manually apply the transformations. Studying the results produced by the analysis/estimation tools (for example, [Atomium]), they use a combination of application know-how, experience and ingenuity to determine the bottlenecks in the design and to select and perform the transformations that can break them. The problem of ensuring an error-free implementation of today's complex application specifications under time-to-market pressure is difficult enough, which is only being further exacerbated by the applied complex transformations. As a result, a dire need exists to supplement/replace testing with automatic verification tools. In fact, there is a general need to scale up verification and testing techniques, to meet the present challenge in ensuring

correctness of embedded software [Cousot and Cousot 2001].

An often suggested approach to ensure correctness of program transformations is the *a priori* method of allowing only a predefined set of transformations to be applied, which are proven to be semantics preserving. If these transformations are applied by a formally verified tool, the transformed program will be *correct by construction*. But, various practical problems exist with this theoretically elegant approach. Firstly, a customized program transformation tool is rarely available for the given context and programming language. Moreover, typical transformation tools provide designers only with a limited set of predefined transformations. This is often too restrictive for designers; they need the flexibility of applying a transformation that is not in the set when they see a clear gain in doing so. Secondly, extendible tools are not a solution as designers lack the time and the skill to introduce new transformations and to formally prove them correct. Thirdly, the correctness of the tool at hand itself is questionable. Although a predefined set of transformations may have been proven to be formally correct, often there is only a prototype implementation and there is no proof that the prototype is a correct implementation. As a result, whether transformations are applied manually or by means of a tool, there is need for an independent verification of the equivalence between initial and transformed program. Testing is tedious, time consuming and insufficient. An equivalence proof by a separate tool can substantially increase the confidence that the functionality is preserved. This has motivated us to look for an *a posteriori* solution to the problem.

Assuming that the initial source code is correct, in this work, we address the problem of automatically checking the *functional equivalence* of the transformed program with the initial program. In other words, we verify that the transformations do not introduce any subtle bugs. Figure 1 shows a toy example of initial and transformed programs which are functionally equivalent from the input-output point of view, i.e., the sequence of values assigned to `B[][]` are the same in both the programs for a given input `A[]`. This example is representative of the class of programs whose equivalence we want to check. Since the equivalence checking problem for programs is, in general, undecidable [Tsichritzis 1970], an automated check has to be based on a decidable condition that is sufficient for equivalence between initial and transformed programs. If the condition holds, the transformation is *safe*, ensuring the equivalence; otherwise, nothing can be concluded. In the latter case, to be useful, the check should be able to pinpoint a reasonably small program fragment that is at the origin of the failure to prove equivalence.

The technique we present in this paper is applicable for loop and data reuse transformations on sequential programs in dynamic single-assignment form (they are the most complex and error prone transformations in the whole design cycle).

I: Initial Program	T: Transformed Program
`...`<br>`for ( i = 0; i < 10; i++ )`<br>`1: B[i][0] = 0;`<br>`for ( i = 0; i < 10; i++ )`<br>`for ( k = 0; k < 8; k++ )`<br>`2: B[i][k+1] = f(B[i][k], A[i*4+k]);`<br>`...`	`...`<br>`for ( i = 0; i < 10; i++ )`<br>`1′: B[i][0] = 0;`<br>`for ( j = 0; j < 4; j++ )`<br>`2′: buf[0][j] = A[j];`<br>`for ( i = 9; i >= 0; i-- ){`<br>`for ( j = 0; j < 4; j++ )`<br>`3′: buf[10-i][j] = A[4*(10-i)+j];`<br>`for ( k = 0; k < 4; k++ )`<br>`4′: B[9-i][k+1] = f(B[9-i][k], buf[9-i][k]);`<br>`for ( k = 7; k > 3; k-- )`<br>`5′: B[9-i][12-k] = f(B[9-i][11-k], buf[10-i][7-k]);`<br>`}`<br>`...`

Figure 1: An example of source-to-source optimizing program transformation

In a program in dynamic single-assignment form (called *single-assignment form* from now on), each variable (array element) is written only once during the execution of the program [Feautrier 1991]. The addressed transformations affect only statements under the loop structures in the program. Hence, it is practical to restrict the verification to checking the equivalence of executing the program statements under the loop structures in the initial and transformed programs. These program statements usually involve array variables (indexed variables) and transforming the loop structures usually also results in the transformation of index expressions. The semantics of a program statement in single-assignment form that reads a number of (array) data elements and writes an (array) data element can be abstracted by a geometric model that describes precisely which elements are read/written in which iterations. Once these models are extracted, it suffices to check the equivalence conditions on the corresponding models of the initial and the transformed programs. Figure 2 puts our scheme in a nutshell. The equivalence checking itself is done completely oblivious of any information about either the particular transformations that have been applied or the order in which they have been applied. As a result, the check provides an *a posteriori* proof of equivalence *independent of the agent* applying the transformations.

This paper is a revision and extension of [Shashidhar et al. 2002].

Outline of the paper. Section 2 describes the source-to-source transformations that are targeted in this work. Section 3 explains in brief the geometric model that we use in our verification technique. Section 4 presents the technique that we propose to a posteriori verify the correctness of these transformations. Section 5 discusses related work and contrasts the work presented in this pa-

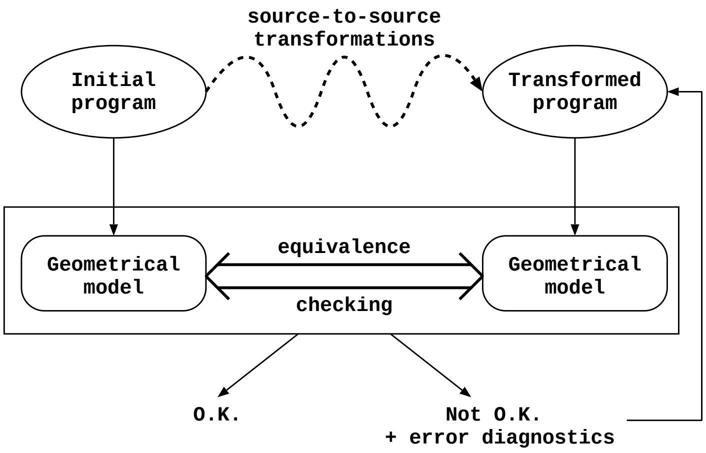

Figure 2: Transformation verification scheme

per with respect to it. We conclude in Section 6 with a brief summary of our contributions and a discussion of future work.

2 Targeted Source-to-source Transformations

2.1 Loop Transformations

Loop transformations play a crucial role in program optimization when the goal is to increase parallelism and to make efficient use of memory hierarchy. They have been well studied as matrix manipulations on index sets. The most primitive of loop transformations are: permutation/interchange, skewing, reversal and bumping on tightly nested loops. A large class of generally applied loop transformations can be derived through successive application of affine unimodular transformations of these primitive types [Banerjee 1994]. Loop distribution/fission/splitting, merging/folding/fusion, strip-mining/tiling, unrolling are other important loop transformations that cannot be derived from the primitives above. Most of the loop transformations applied in practice belong to one of the above types. Also, some of these transformations are just enabling transformations for other loop transformations and do not result in any optimization by themselves.

The loop transformations change only the execution ordering while the overall computation remains essentially the same. If the program is in single-assignment form and every element is written before being read, then the set of elements of the variables read and written, and the dependency between them should remain unaltered by the transformation. As will be explained in Section 3, the

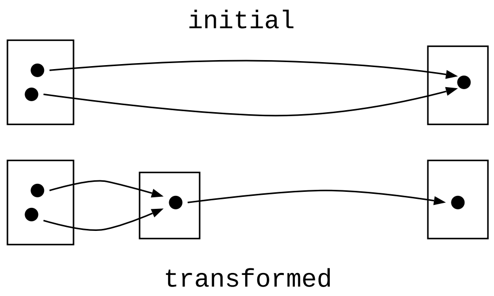

Figure 3: The principle of data reuse transformation

geometric model captures this information independent of the particular loop transformation that is applied, hence, enabling us to verify the whole set of structure preserving and structure modifying loop transformations that were mentioned above.

The example in Figure 1 shows a simple loop distribution transformation on the inner loop and a loop reversal transformation on the outer loop and one of the inner loops, along with a to be explained data reuse transformation. Though the transformations applied in the example are trivial compared to transformations applied in multimedia applications for rigorous optimization, they illustrate the complexity involved in checking the correctness.

2.2 Data Reuse Transformations

Efficient use of a customized memory hierarchy to exploit temporal locality in data accesses is very important for optimal design of embedded systems with less energy consumption in the memory system. Hardware controlled caches exploit this, but at a very significant energy cost. Hence there is an increased interest in software controlled caching. Compile time introduced data reuse transformations on the program enable this in a system-level design framework [Catthoor et al. 1998].

The data reuse transformation involves the introduction of a buffer variable to hold the data element that is accessed multiple times as shown in Figure 3. The introduction of buffer variables usually also requires that loop structure and index expressions of the array variables are transformed. Clearly this transformation is semantics preserving. But, a mistake made during application of such a transformation on non-trivial programs might introduce subtle bugs.

The fairly simple initial and transformed program pair in Figure 1 demonstrates the transformation. Here, the `buf[][]` variable is introduced to hold the reused elements of `A[]`. The `buf[][]` variable has the same number of elements as `A[]`, this is because of the requirement of single-assignment form. Later

transformation steps in the full transformation script remove these redundancies [Catthoor et al. 1998, Quilleré and Rajopadhye 2000]. The example shows data reuse with a single cache, but in practice, multi-level reuse is often required, making it very hard to check manually that the semantics is preserved.

3 Geometric Modeling of Programs

Our approach relies on the use of the geometric model (also called polyhedral model) for abstracting the meaning of programs. Geometric modeling of programs is well known in the parallel compiler and regular array synthesis research domains and is used quite extensively to analyze the execution of program statements [Feautrier 1991, Pugh 1992, Quilleré and Rajopadhye 2000]. The geometric model, while being quite simple, concisely represents all of the necessary information about the data and control flow in the program. We use formulas that encode affine constraints on integer variables, symbolic constants, logical connectives and quantifiers, also called *Presburger formulas*, to symbolically represent the domain spaces and mapping between them. The geometric model is explained at length in [Catthoor et al. 1998]. Here, we give only the definitions that are required to present our technique.

In our notation, we follow the convention that the left super-script denotes the program from which the object has been extracted and the left and right sub-scripts denote the statement number and the program variables referred by the object, respectively.

Definition 1 Iteration domain. Geometric domain in which each point with integer coordinates represents exactly one execution of an assignment statement.

If the execution of the assignment statement is controlled by k iterator variables, the iteration domain will be a k-dimensional *linearly bounded lattice* (LBL) [Thiele and Arzt 1993]. For an assignment statement labelled s in program P, the domain will be denoted by ${}^{\mathtt{P}}_{s}\mathbf{D}^{\mathtt{iter}}$. For example, the iteration domain of statement 2 in the initial program I in Figure 1 is as given below:

$${}^{\mathtt{I}}_{2}\mathbf{D}^{\mathtt{iter}} := \{\, [i,k] \,|\, 0 \leq i \leq 9 \;\wedge\; 0 \leq k \leq 7 \;\wedge\; [i,k] \in \mathbb{Z}^2 \}$$

The `if-then-else` constructs, if present, introduce additional constraints on the domain with their branch conditions.

Definition 2 Definition domain. Geometric domain in which each point with integer coordinates $(i_1, \ldots, i_n)$ represents exactly one write to $v[i_1, \ldots, i_n]$, an element of a variable v defined by an assignment statement.

If v is a defined variable in an assignment statement labelled s in program P, its definition domain is denoted by ${}^{\mathtt{P}}_{s}\mathbf{D}^{\mathtt{def}}_{v}$.

Definition 3 Operand domain. Geometric domain in which each point with integer coordinates $(i_1, \ldots, i_n)$ represents exactly one read from an element $v[i_1, \ldots, i_n]$ of an operand variable v in an assignment statement.

If v is an operand variable in an assignment statement labelled s in program P, its operand domain is denoted by ${}^{\mathtt{P}}_{s}\mathbf{D}^{\mathtt{oper}}_{v}$. For a d-dimensional array variable, the definition and operand domains will be LBLs of the same dimension. For example, the definition domain of `B[][]` and operand domain of `A[]` in statement 2 of program I in Figure 1 is:

$$ {}^{\mathtt{I}}_{2}\mathbf{D}^{\mathtt{def}}_{\mathtt{B}} := \{\, [a_1, a_2] \,|\, a_1 = i \,\wedge\, a_2 = k + 1 \,\wedge\, [i, k] \in {}^{\mathtt{I}}_{2}\mathbf{D}^{\mathtt{iter}} \} $$

$$ {}^{\mathtt{I}}_{2}\mathbf{D}^{\mathtt{oper}}_{\mathtt{A}} := \{\, [a_3] \,|\, a_3 = i * 4 + k \,\wedge\, [i, k] \in {}^{\mathtt{I}}_{2}\mathbf{D}^{\mathtt{iter}} \} $$

Definition 4 Dependency mapping. A mapping associated with an assignment statement, between a defined variable d and an operand variable o. Each pair (i, j) in the mapping indicates that $d[i]$ is written (defined) and $o[j]$ is read by an instance of the statement.

We denote the dependency mapping between the defined variable v and the k-th operand variable w_k in an assignment statement labelled s in program P by ${}^{\mathtt{P}}_{s}\mathbf{M}_{v\,w_k}$. For example, the dependency mapping between the defined variable `B[][]` and the second operand variable `A[]` in statement 2 of I in Figure 1 is:

$$ {}^{\mathtt{I}}_{2}\mathbf{M}_{\mathtt{B}\,\mathtt{A}} := \{\, [a_1, a_2] \rightarrow [a_3] \;|\; a_1 = i \,\wedge\, a_2 = k + 1 \\ \wedge\, a_3 = i * 4 + k \,\wedge\, [i, k] \in {}^{\mathtt{I}}_{2}\mathbf{D}^{\mathtt{iter}} \} $$

The dependency mapping $\mathbf{M} : \mathbf{D}^{\mathtt{def}} \rightarrow \mathbf{D}^{\mathtt{oper}}$, as evident from the definition, is a mapping from a definition domain to an operand domain which is neither surjective nor injective. It is an integer tuple relation describing the complete information about which elements of the defined variable depends on which elements of the operand variable during all possible executions of the statement. Each tuple in the relation corresponds to exactly one dependency mapping between the elements of the defined and operand variables. Without loss of generality, let us assume that an assignment statement has only one defined variable. Therefore, an assignment statement with n operand variables will give n dependency mappings from the defined variable, one to each of its operand variables. If an operand variable appears more than once in an assignment statement, we use an additional subscript to distinguish them by their position. A *copy* statement is a statement with only one operand, on which the identity function is applied. Hence it has only one dependency mapping.

The class of programs we can handle presently are composed of assignment statements, `for`-loop statements, and `if-then-else` statements. The semantics

of the expression in the assignment statement is not interpreted. Such programs can be parsed to obtain the above domains and mappings. An important point to make here is that the above model can be extracted from programs written in any imperative programming language that provides the handled statements. This makes the equivalence checking independent of the particular programming language in which the initial and the transformed programs have been written. The model described in [Catthoor et al. 1998] includes other definitions that capture data flow information like dependency distance vector, direction vector etc. But they are relevant for equivalence checking only to the extent that they provide information about the ordering of reads and writes to elements of array variables.

Given a geometric model of a program, one can identify array elements occurring in an operand domain but not in a definition domain. They are the *inputs* of the program. Similarly, some elements may occur in a definition domain, but not in an operand domain. Assuming the program does not perform useless computations, they are *outputs*. However, also elements that are read may be intended as output. Hence we assume the outputs are declared for the program pair. The semantics (meaning) of a program can be characterized by the function mapping inputs to outputs (*input-output function*). Note that domains are finite, hence that geometric models only describe terminating programs.

3.1 Assumptions

Our transformation verification technique addresses only program pairs that can be abstracted by geometric models. Moreover, we require a rather close correspondence between initial and transformed programs, namely that differences are limited to changes in the loop structure and the index expressions, and to the introduction of buffers. While restrictive, it covers the important and error prone class of transformations applied by designers when optimizing data transfer and storage costs. The assumptions listed below make the requirements explicit.

– In many program transformation frameworks, it is common to transform the program first to the single-assignment form as it provides much more freedom in applying optimizing transformations. Hence, we require that programs are in single-assignment form and also that they are free from pointers. We assume that a preprocessing stage, partly described in [Catthoor et al. 1998], has correctly transformed the source code to the required form.

– The index expressions and the expressions giving the bounds of the iterators are quasi-affine functions (affine functions with mod, div, floor and ceil operations) of only the surrounding iterator variables and symbolic constants.

– The technique is concerned with only the reads and writes of elements of array variables. Scalar variables are taken to be single element arrays under the single-assignment form. The verification of correctness of arithmetic and logic expressions can be handled with other techniques mentioned in Section 5. The integration with other techniques is straightforward if the source code is organized into two layers, wherein, the computation with loop constructs and array variables are sorted into one and the computation with scalar variables into another. This in fact is essential to facilitate manual transformations on the former, while leaving the optimization of the latter to the compiler [Catthoor et al. 1998].

– The transformations do not change the variable names and their types in the program.

– We assume that transformations only involve (1) the introduction of buffer arrays (caches), that are written by copying other array elements and (2) reorganization of the loop structure that preserves the functions applied on the data elements in the right hand side of write statements i.e., only modification of index expressions and replacement of an array being read by buffer(s) are supported by our analysis.

– We assume that in initial and transformed programs each variable element that is read in an assignment statement has either been already defined or is an input variable. This can be ensured by a well known data-flow analysis for array elements [Wolfe 1996].

In the technique that follows, we use the following two operations on integer tuple relations in addition to the usual operations on sets.

Operation and its definition
$F \bowtie G$ (Join of F and G):<br>$x \rightarrow z \in F \bowtie G \Leftrightarrow \exists y$ s.t. $x \rightarrow y \in F \wedge y \rightarrow z \in G$
F^+ (Positive transitive closure of F):<br>$x \rightarrow z \in F^+ \Leftrightarrow x \rightarrow z \in F \vee \exists y$ s.t. $x \rightarrow y \in F \wedge y \rightarrow z \in F^+$

4 Transformation Verification Technique

The transformation verification technique is an implementation of the scheme shown in Figure 2. Given the initial and the transformed program pair (I, T), the geometric models are extracted from the two programs and their equivalence is shown, *i.e.*, it is verified that both programs compute the same function from inputs to outputs. In addition, in case of failure, the purpose is to obtain

I: Initial Program	T: Transformed Program
`{`	`{`
`....`	`....`
`i: v[]= f(u1[],u2[],..,un[]);`	`h': buf[] = u2[];`
`....`	`....`
`j: v[]= g(w1[],w2[],..,wm[]);`	`i': v[]= f(u1[],buf[],..,un[]);`
`....`	`....`
`k: v[]= f(u1[],u2[],..,un[]);`	`j': v[]= g(w1[],w2[],..,wm[]);`
`....`	`....`
`}`	`k': v[]= f(u1[],u2[],..,un[]);`
	`....`
	`l': v[]= f(u1[],buf[],..,un[]);`
	`....`
	`}`

Figure 4: Example to explain statement classes. Each $v[..]$ is a different element of v as the programs are in single-assignment form.

a diagnosis that identifies the statements that are at the origin of the failing verification.

Before we present the technique in detail, we define two notions of equivalence of statements and a partition of statements into classes.

Definition 5 Weakly equivalent statements. Statements s_1 and s_2 are *weakly equivalent* if they define the same array variable and apply the same function on their operand variables.

Definition 6 Equivalent statements. Statements s_1 and s_2 are *equivalent* if they are weakly equivalent and if their corresponding operand variables are identical.

Let ${}^{\mathtt{P}}\mathbf{S}_v^{\mathtt{def}}$ be the set of statements defining the variable v in a program P.

Definition 7 Statement class, $\pi({}^{\mathtt{P}}\mathbf{S}_v^{\mathtt{def}})$ and ${}^{\mathtt{P}}\mathbf{R}_v$. A *statement class* for an array variable v in a program is a maximal subset of equivalent statements from the set ${}^{\mathtt{P}}\mathbf{S}_v^{\mathtt{def}}$ of statements defining v. The set of statement classes for v (a partition of ${}^{\mathtt{P}}\mathbf{S}_v^{\mathtt{def}}$) is given by the function $\pi({}^{\mathtt{P}}\mathbf{S}_v^{\mathtt{def}})$; ${}^{\mathtt{P}}\mathbf{R}_v$ (with an extra superscript if needed) denotes a member of this set, *i.e.* a statement class for v.

For example, in the initial program I in Figure 4, the set of statements defining the array variable v, ${}^{\mathtt{I}}\mathbf{S}_v^{\mathtt{def}} = \{i, j, k\}$ and its partition $\pi({}^{\mathtt{I}}\mathbf{S}_v^{\mathtt{def}}) = \{{}^{\mathtt{I}}\mathbf{R}_v^1, {}^{\mathtt{I}}\mathbf{R}_v^2\}$, where ${}^{\mathtt{I}}\mathbf{R}_v^1 = \{i, k\}$ and ${}^{\mathtt{I}}\mathbf{R}_v^2 = \{j\}$ are statement classes with defined variable v.

${}^{\mathtt{I}}\mathbf{R}_v^1$ has operand variables $u_1, \ldots, u_n$ and applied function f; ${}^{\mathtt{I}}\mathbf{R}_v^2$ has operand variables $w_1, \ldots, w_m$ and applied function g.

As mentioned above, the relevant behavior of a program is described by the input-output function. As programs are in single-assignment form and all statements contribute to the output, the input-output function of the initial program I is completely determined by two things. On one hand the dependency mappings ${}^{\mathtt{I}}_{s}\mathbf{M}_{v\,w}$ of the statements in I and on the other hand the functions applied on the operands in the right-hand side of those statements. Hence a sufficient condition for preserving the input-output function in the transformed program is that these dependency mappings are (directly or indirectly) still present in the transformed program. Part of our assumptions is that array variables are preserved and that also the functions applied on the operands in the right-hand sides of statements are preserved. However, operands can be replaced by buffer variables which in turn are defined in new copy statements. In such cases, dependency mappings are not preserved. By composing the dependency mapping where the operand is a buffer variable with the dependency mapping of the copy statement defining the buffer variable (if needed, repeating this) one eventually obtains an (indirect) dependency mapping relating two variables present in the initial program. This idea is formalized below. In what follows we refer to arrays occurring in the initial program as array variables (or arrays) and to arrays introduced in the transformed program as buffer variables (or buffers).

Definition 8 Related statements $\tau({}^{\mathtt{I}}\mathbf{R}_v)$. Given a statement class ${}^{\mathtt{I}}\mathbf{R}_v$ in the initial program I, the *related statements* in the transformed program, denoted $\tau({}^{\mathtt{I}}\mathbf{R}_v)$, are defined as the set $\{s \mid s \in {}^{\mathtt{T}}\mathbf{S}_v^{\mathtt{def}}$ and s is weakly equivalent with the statements of ${}^{\mathtt{I}}\mathbf{R}_v\}$.

For example, in the transformed program T in Figure 4, $\tau({}^{\mathtt{I}}\mathbf{R}_v^1) = \{i', k', l'\}$. To be able to verify that dependency mappings in the initial program are preserved in the transformed program, we define an indirect dependency mapping that eliminates buffer variables.

Definition 9. Let v and w be array variables and $b_1, \ldots, b_n$ be buffer variables such that: (1) there is a statement s_v with a dependency mapping between v and b_1, (2) there are copy statements $s_1, \ldots, s_{n-1}$ where each s_i has a dependency mapping between b_i and b_{i+1}, and (3) there is a copy statement s_n with a dependency mapping between b_n and w. Then the indirect dependency mapping $\mathbf{M}'_{v\,w}(s_v, s_1, \ldots, s_n)$ is given by:

$$\mathbf{M}'_{v\,w}(s_v, s_1, \ldots, s_n) := {}_{s_v}\mathbf{M}_{v\,b_1} \bowtie {}_{s_1}\mathbf{M}_{b_1\,b_2} \bowtie \cdots \bowtie {}_{s_{n-1}}\mathbf{M}_{b_{n-1}\,b_n} \bowtie {}_{s_n}\mathbf{M}_{b_n\,w}$$

where the operator $\bowtie$ is as defined in Section 3.

In what follows, we call $s_v, s_1, \ldots, s_n$ a buffer chain between v and w. Note that, for given arrays v and w, several buffer chains of possibly different lengths may exist. As the code is in single-assignment form, each chain relates a distinct set of pairs of indices in the arrays v and w. We are interested in the union of all these mappings, not only for a single statement defining v but for a set of weakly equivalent statements. Hence we define:

Definition 10 Indirect dependency mapping $\mathbf{M}^{'}_{v\,w}(S)$. Let S be a set of weakly equivalent statements defining an array v. Let $C(v, w, s)$ be the set of buffer chains between v and w starting in s. Then $\mathbf{M}^{'}_{v\,w}(S)$ is defined as

$$\bigcup_{s \in S} \; \bigcup_{s,\ldots,s_n \in C(v,w,s)} \mathbf{M}^{'}_{v\,w}(s, \ldots, s_n)$$

A computational problem arises when there is a *self dependence* in a copy statement s from a buffer b_i to itself. It is called a *recurrent mapping* and is detected when the intersection ${}^{\mathtt{T}}_{s}\mathbf{D}^{\mathtt{def}}_{b_i} \cap {}^{\mathtt{T}}_{s}\mathbf{D}^{\mathtt{oper}}_{b_i}$ is non-empty. To avoid the inefficiency of having to consider chains $\ldots b_{i-1}, b_i, \ldots, b_i, b_{i+1}, \ldots$ containing variable length subsequences of b_i, we compute a so called end-to-end mapping M containing pairs of indices (k, l) such that $b_i[k]$ is defined by s while $b_i[l]$ is not defined by s and use this mapping M instead of a sequence of $\mathbf{M}_{b_i\,b_i}$ when computing the indirect dependency mapping. It can be calculated as follows:

- Compute the positive transitive closure of the recurrent mapping:
  $m := ({}^{\mathtt{T}}_{s}\mathbf{M}_{b_i\,b_i})^{+}$.
- Get the domain and range of the computed closure:
  $d := \text{domain}(m)$; $r := \text{range}(m)$.
- Get the domain and range of the end to end mapping:
  $d' := (d - r)$; $r' := (r - d)$.
- Restrict the closure to the tuples in the end-to-end mapping:
  $M := \{x \to y \,|\, x \to y \in m \wedge x \in d' \wedge y \in r'\}$

The primitives used in the above procedure are provided by the Omega library [Kelly et al. 1996a]. It is important to remark here that transitive closure is exactly computable only under certain conditions, but this limitation is not a problem for most of the commonly occurring relations in practice [Kelly et al. 1996b]. Another remark is that, we do not have to compute the transitive closure for self dependences on output variables, because the transformations ensure that they are preserved in the transformed program. This is the case with the self dependence of variable `B[][]` in statement 2 in our example in Figure 1.

Now, a sufficient condition for ensuring that the transformed program computes the same input-output function as the initial program is that every tuple (a, b) in a dependency mapping ${}^{\mathtt{I}}_{s}\mathbf{M}_{v\,w}$ of the initial program is part of the indi-

rect dependency mappings between v and w of the weakly equivalent statements of the transformed program. This is given by the following definition.

Definition 11 Equivalence condition. For each variable v defined in the initial program I it must be the case that, $\forall^{\mathtt{I}}\mathbf{R}_v \in \pi({}^{\mathtt{I}}\mathbf{S}_v^{\mathtt{def}})$, $\forall k$ such that $1 \leq k \leq n$:

$$\bigcup_{s \in {}^{\mathtt{I}}\mathbf{R}_v} {}^{\mathtt{I}}_{s}\mathbf{M}_{v\,w_k} \subseteq {}^{\mathtt{T}}\mathbf{M}'_{v\,w_k}(\tau({}^{\mathtt{I}}\mathbf{R}_v))$$

where n is the number of operands in the equivalent statements of ${}^{\mathtt{I}}\mathbf{R}_v$ and w_k is their k-th operand.

The sufficiency of the equivalence condition is formulated in the following theorem:

Theorem 12. *Let I and T be a pair of programs in single-assignment form which have the same inputs and outputs and for which the equivalence condition holds. Then both programs compute the same input-output function.*

Proof. (Sketch.) Without loss of generality, we assume arrays have only one index. We have to prove that if an output array element has a value $\mathbf{v}$ in I then it has the same value $\mathbf{v}$ in T.

Let $o[i]$ be an output array element. The value $\mathbf{v}$ assigned to $o[i]$ in I is given by a function $f(u_1[i_1], \ldots, u_m[i_m])$. The dependency mapping ${}^{\mathtt{I}}\mathbf{M}_{o\,u_k}$ identifies the element of $u_k[i_k]$ that serves as k-th input to f. In T, the value $\mathbf{v}$ assigned to $o[i]$ is given by $f(u'_1[l_1], \ldots, u'_m[l_m])$. The indirect dependency mapping ${}^{\mathtt{T}}\mathbf{M}'_{o\,u_k}$ identifies the element $v_k[j_k]$ that is at the origin of the value of $u'_k[l_k]$ and hence serves as k-th input to f in T.

Hence $o[i]$ is assigned the same value in T when $u_k[i_k] = v_k[j_k]$ for all k. The equivalence condition ensures that $u_k = v_k$ and $i_k = j_k$. It remains to show that $u_k[i_k]$ has the same value in I and T. This holds trivially when it concerns an element of an input array. In the other case, one can apply the same reasoning as for $o[i]$ and conclude, by induction, that indeed all array elements $u_k[i_k]$ have the same value in I and T. □

It is desirable to verify also that the transformed program does not perform useless computations, *i.e.*, does not define more array elements than the initial. An inexpensive check, that can be done before testing the equivalence condition, is to verify that the definition domains in the initial and transformed programs are the same for all array variables.

Formally, the following condition should hold for each defined variable v in the initial program I:

$$\bigcup_{s \in {}^{\mathtt{I}}\mathbf{S}_v^{\mathtt{def}}} {}^{\mathtt{I}}_{s}\mathbf{D}_v^{\mathtt{def}} = \bigcup_{t \in {}^{\mathtt{T}}\mathbf{S}_v^{\mathtt{def}}} {}^{\mathtt{T}}_{t}\mathbf{D}_v^{\mathtt{def}}$$

Example 1. To illustrate the technique, we verify in the example of Figure 1 that the relation between defined variable and the *second* operand variable, i.e., the variable pair (`B[][]`, `A[]`) of statement 2 in I is preserved in T:
The iteration domain is: ${}^{\mathtt{I}}_{2}\mathbf{D}^{\mathtt{iter}} := \{\, [i,k] \,|\, 0 \leq i \leq 9 \;\wedge\; 0 \leq k \leq 7 \;\wedge\; [i,k] \in \mathbb{Z}^2\}$
Let $C_0 := (\, a_4 = i \;\wedge\; a_5 = k+1 \;\wedge\; a_3 = i*4+k \;\wedge\; [i,k] \in {}^{\mathtt{I}}_{2}\mathbf{D}^{\mathtt{iter}})$
The array dependency between `B` and `A` is given by:
${}^{\mathtt{I}}_{2}\mathbf{M}_{\mathtt{B\,A}} := \{\, [a_4, a_5] \to [a_3] \,|\, C_0 \,\}$

The function τ maps statement 2 in I to the statements $4'$ and $5'$ of T. But, array `A` is replaced by `buf`, which is defined in statements $2'$ and $3'$ in T. Hence, the dependency between `buf` and `A` in the statements $2'$ and $3'$ has to be used in computing the indirect dependency between `B` and `A`.
Statement $2'$ of T has the iteration domain:
${}^{\mathtt{T}}_{2'}\mathbf{D}^{\mathtt{iter}} := \{\, [j] \,|\, 0 \leq j \leq 3 \;\wedge\; [j] \in \mathbb{Z}\}$
The following constraint will be used in its dependency mapping:
$C_2 := (\, a_1 = 0 \;\wedge\; a_2 = j \;\wedge\; a_3 = j \;\wedge\; [j] \in {}^{\mathtt{T}}_{2'}\mathbf{D}^{\mathtt{iter}})$
For statement $3'$, iteration domain and the constraint are respectively:
${}^{\mathtt{T}}_{3'}\mathbf{D}^{\mathtt{iter}} := \{\, [i,j] \,|\, 0 \leq i \leq 9 \;\wedge\; 0 \leq j \leq 3 \;\wedge\; [i,j] \in \mathbb{Z}^2\}$ and
$C_3 := (\, a_1 = 10 - i \;\wedge\; a_2 = j \;\wedge\; a_3 = 4*(10-i)+j \;\wedge\; [i,j] \in {}^{\mathtt{T}}_{3'}\mathbf{D}^{\mathtt{iter}})$
Resulting dependency mappings are:
${}^{\mathtt{T}}_{2'}\mathbf{M}_{\mathtt{buf\,A}} := \{\, [a_1, a_2] \to [a_3] \,|\, C_2 \,\}$; ${}^{\mathtt{T}}_{3'}\mathbf{M}_{\mathtt{buf\,A}} := \{\, [a_1, a_2] \to [a_3] \,|\, C_3 \,\}$

For statement $4'$, iteration domain and the constraint are respectively:
${}^{\mathtt{T}}_{4'}\mathbf{D}^{\mathtt{iter}} := \{\, [i,k] \,|\, 0 \leq i \leq 9 \;\wedge\; 0 \leq k \leq 3 \;\wedge\; [i,k] \in \mathbb{Z}^2\}$
$C_4 := (\, a_4 = 9 - i \;\wedge\; a_5 = k+1 \;\wedge\; a_1 = 9 - i \;\wedge\; a_2 = k \;\wedge\; [i,k] \in {}^{\mathtt{T}}_{4'}\mathbf{D}^{\mathtt{iter}})$
Finally, for statement $5'$ iteration domain and the constraint are respectively:
${}^{\mathtt{T}}_{5'}\mathbf{D}^{\mathtt{iter}} := \{\, [i,k] \,|\, 0 \leq i \leq 9 \;\wedge\; 4 \leq k \leq 7 \;\wedge\; [i,k] \in \mathbb{Z}^2\}$
$C_5 := (\, a_4 = 9 - i \;\wedge\; a_5 = 12 - k \;\wedge\; a_1 = 10 - i \;\wedge\; a_2 = 7 - k \;\wedge\; [i,k] \in {}^{\mathtt{T}}_{5'}\mathbf{D}^{\mathtt{iter}})$
Resulting dependency mappings are:
${}^{\mathtt{T}}_{4'}\mathbf{M}_{\mathtt{B\,buf}} := \{\, [a_4, a_5] \to [a_1, a_2] \,|\, C_4 \,\}$; ${}^{\mathtt{T}}_{5'}\mathbf{M}_{\mathtt{B\,buf}} := \{\, [a_4, a_5] \to [a_1, a_2] \,|\, C_5 \,\}$

We have that:
${}^{\mathtt{T}}\mathbf{M}_{\mathtt{buf\,A}} := {}^{\mathtt{T}}_{2'}\mathbf{M}_{\mathtt{buf\,A}} \cup {}^{\mathtt{T}}_{3'}\mathbf{M}_{\mathtt{buf\,A}} := \{\, [a_1, a_2] \to [a_3] \,|\, C_2 \;\vee\; C_3 \,\}$
and ${}^{\mathtt{T}}\mathbf{M}_{\mathtt{B\,buf}} := {}^{\mathtt{T}}_{4'}\mathbf{M}_{\mathtt{B\,buf}} \cup {}^{\mathtt{T}}_{5'}\mathbf{M}_{\mathtt{B\,buf}} := \{\, [a_4, a_5] \to [a_1, a_2] \,|\, C_4 \;\vee\; C_5 \,\}$

Hence the indirect dependency mappings in the transformed program are:
${}^{\mathtt{T}}\mathbf{M}'_{\mathtt{B\,A}}(\{4', 5'\}) := {}^{\mathtt{T}}\mathbf{M}_{\mathtt{B\,buf}} \bowtie {}^{\mathtt{T}}\mathbf{M}_{\mathtt{buf\,A}} := \{\, [a_4, a_5] \to [a_3] \,|\, (C_2 \vee C_3) \wedge (C_4 \vee C_5) \,\}$

Define: $C_1 := ((C_2 \;\vee\; C_3) \;\wedge\; (C_4 \;\vee\; C_5))$
Now, the equivalence condition is satisfied when:
${}^{\mathtt{I}}_{2}\mathbf{M}_{\mathtt{B\,A}} - {}^{\mathtt{T}}\mathbf{M}'_{\mathtt{B\,A}}(\{4', 5'\}) := \{\, [a_4, a_5] \to [a_3] \,|\, C_0 \;\wedge\; \neg C_1\} = \emptyset$
This can be verified with the Omega test framework [Pugh 1992].

4.1 Error Diagnosis

A successful verification implies that initial and final programs are equivalent. Failure indicates either a genuine error or that the transformation is beyond the assumptions about the syntactical correspondence between initial and transformed program, e.g. that functions in right hand side of statements have been modified or that operations other than plain copy operations are used when filling the buffer arrays. If a condition does not hold it means that some points are missing in the domain or the mapping in question. Since our condition checks are made by calculating the differences of domains for each variable or mappings for each variable pair separately for each statement class, the resulting non-empty set of points gives enough information about the location of the errors. The variable or the variable pair in question and the missing range of index values is sufficient to direct the designer to the part of the code under an erroneous transformation. This is a very useful property of the presented technique.

4.2 Complexity and Experience

The condition checks as described evaluates the validity of the constraints and the best known upper bound for determining validity in Presburger arithmetic is $2^{2^{2^{pn}}}$ on the length of the formula [Oppen 1978], where $p > 1$ is some constant. The OMEGA test framework [Pugh 1992] based on Fourier-Motzkin variable elimination and a host of heuristics provides an integer programming solver for the Presburger arithmetic which is very efficient in practice. This has prompted us to use the OMEGA calculator [Kelly et al. 1996a] to perform the condition checks on our domains and mappings. The mappings that we check are taken separately for each definition-operand variable pairs and hence, the length of the formula depends solely on the size of the statement classes and in all practical cases the problem size remains reasonable.

We have implemented our technique in a prototype tool which integrates calls to the geometric model extractor and the OMEGA calculator and coordinates the constructed checks and provides error location information to the user. The tool has successfully verified some real life examples with many complex loops and multi-dimensional arrays, like data reuse transformations on MPEG-4 motion estimation kernel and loop transformations on implementations of signal processing application cores like Durbin and updating singular value decomposition (USVD) algorithm. The verification was possible in a *push-button* style and took time only in the order of few seconds. In the USVD case, the tool detected a bug in the transformed USVD (400 lines of C-code in the core), which was traced to a bug in the constant propagation unit of the code generator that a prototype loop transformation tool used. In the past, both testing and manual paper-and-pencil based checking had taken unreasonable amount of time and yet without guarantee of correctness.

5 Related Work

The front line formal verification techniques, model checking and theorem proving, are not suited to the problem that we are addressing. Model checking is not suitable because we are dealing with sequential data dominated programs which are not amenable to be represented as state transition systems. Symbolic model checking of infinite state systems has been presented in [Bultan et al. 1997] for verifying temporal properties, which is not the focus of our work. But, we do use a similar framework in addressing our problem. Theorem proving is unattractive to the designer because of the often quoted requirement of skill. Also, techniques applied for verification of equivalence of implementation to the behavioral specification [Claesen et al. 1992, van Aelten et al. 1994] and other implementation level verification techniques (for example those based on SAT solvers like Chaff [Moskewicz et al. 2001]), are suited for checking arithmetic and logic expressions, but not for loop constructs on array variables in the source code. A solution proposed often is to completely unroll the loop, but this is clearly infeasible given that the loops are nested and the bounds are quite large in real programs, especially in embedded multimedia applications. In particular, SFG-Tracing [Claesen et al. 1992] provides proof of equivalence of loop constructs based on induction with the restriction that loop ordering is unchanged. But, automation has only been possible for non-loop transformations.

The work on translation validation [Pnueli et al. 1999, Necula 2000], with motivation that props our own, addresses a very related problem of *a posteriori* validating whether the target code produced by a compiler is a correct translation of the source program, providing an alternative to the verification of translators/compilers. In this technique, a trade-off exists between the class of transformations that can be checked and the extent of compiler instrumentation that is required to provide enough information to the validator about the transformations applied. In the closely related Verifix project [Goos and Zimmermann 1999], methods were proposed to prove that the implementation of the compiler meets the *compiling specification* and also to check the correctness of the compiled code by program checking. In contrast to *translation/compilation*, our concern is on the source-to-source *transformations*, which is mainly a pre-compilation activity. More importantly, the problem is compounded here by the fact that usually transformations are made manually in the context of system-level hardware-software co-design of embedded systems and it is desirable to have first a verification at the level of the source code before getting down to compilation and synthesis. Our attempt here has been to provide a transformation verification infrastructure which is complementary to translation validation and verification of the implemented compiler. In Figure 5 the line in bold delineates the problem that we are addressing in contrast to other related problems. With an altogether different goal, source-code level approximate equiv-

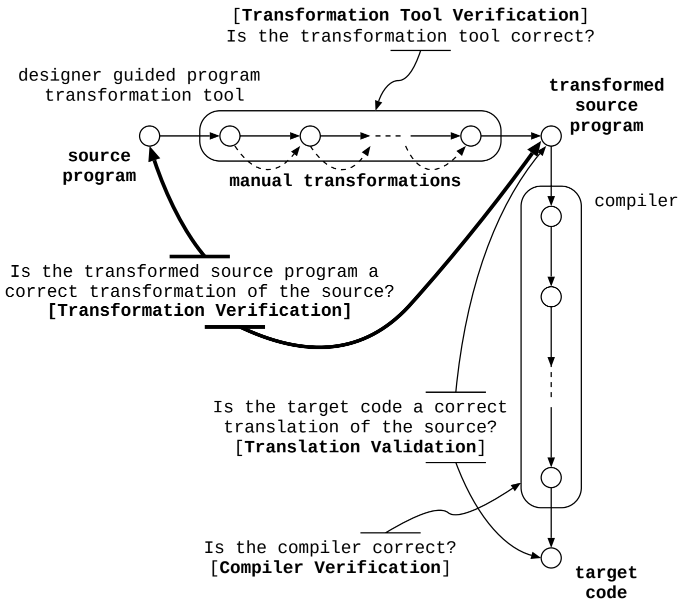

Figure 5: Contrasting with related work

alence checking methods [Yang et al. 1989, Ramalingam and Reps 1989] have been proposed based on program representation graphs and program slicing in the context of program integration. But, their method is restricted to a language subset which omits array variables and hence is not suited to address our problem.

In [Mateev et al. 2001], a technique called fractal symbolic analysis (FSA) is introduced to address the same problem as we are. Their idea is to reduce the difference between the two programs by incrementally applying simplification rules until the two programs become close enough to allow a proof by symbolic analysis. Each simplification rule preserves the meaning of the program. The programmer can also assist the analyzer by providing some problem specific program invariants. The power of FSA depends on the simplification rules that are available. However, the more rules, the larger the search space and it is yet unclear whether the heuristics to measure the difference and to select the most promising simplification rule are sufficient in practice. In comparison, our method, while addressing a more limited (but in practice important) class of transformations, does neither require search nor guidance from the programmer.

In prior work, at Imec, the feasibility of the approach in handling pure loop transformations has been demonstrated [Samsom et al. 1995] and also, a heuristic to handle bigger problem sizes has been proposed [Čupák et al. 1998]. The

approach of [Samsom et al. 1995] was to define for each statement one complex dependency mapping instead of a separate one for each operand. This had prevented handling data reuse transformations in all but very simple cases. Splitting the dependency mapping into a separate one for each operand not only allows the handling of reuse but also substantially reduces the computational complexity of checking the equivalence conditions. As a consequence, we can analyze much larger programs.

6 Conclusions

Correctness checking is a complex problem which manifests itself in many contexts in varied forms eluding a general solution, hence it is important to explore every avenue of entry available to tackle the problem. In this paper we describe a technique that can assist designers of embedded systems for multimedia and telecommunication applications in verifying that the applied loop and data reuse transformations are correct. The main idea behind the technique is to extract the geometric model from the initial and the transformed program and to show that both models define the same input-output function.

Our technique relies on a rather close relationship between initial and transformed programs. Indeed, we assume that all array variables of the initial program are preserved in the transformed program and also that the same functions are applied on the operands of the statements defining these array variables. An interesting question is whether this close correspondence can be relaxed. One way to do this is to start with the goal of showing that the outputs in the two programs are the same. This can be expressed by a finite number of expressions $o_I[i] = o_T[i]$ where i ranges over an LBL (o_I and o_T refer to the outputs in the initial and the transformed programs, respectively). Using the dependency mappings of the defining statements, such a goal can be reduced to showing equalities between the operands, provided that the defining statements apply the same function on their operands. Recursively applying this reduction, one can eventually reduce these goals to equalities between inputs. A very recent work [Barthou et al. 2002], independent to our work [Shashidhar et al. 2002], precisely follows this approach. They represent programs by systems of affine recurrence equations (SAREs), which correspond to geometric models of programs in single-assignment form. In this approach, because of the goal reduction strategy, only the input and output variables of the initial program have to be preserved in the transformed program.

One of the most limiting requirements of our method (as well as other techniques based on geometric modeling) is that programs must be in single-assignment form. For programs that meet all other requirements, there are methods [Wolfe 1996, Feautrier 1988] to transform programs to single-assignment

form. Also, there are methods (for example, [van Engelen and Gallivan 2001]) which aim at converting pointers in a program to array accesses. Such preprocessing techniques could substantially broaden the class of programs that can be verified.

In future work we would like to handle a broader class of transformations and would like to be able to cope with differences in the statements. For example differences that can be explained by the algebraic properties (commutativity, associativity,...) of the applied functions or those which are the result of moving some computation out of a loop (*e.g.* $f(x)$ in one program corresponding to $g(x) + c$ in the other).

Acknowledgements

The authors thank Eddy De Greef for useful discussions during the course of the presented work, Sven Verdoolaege for providing interesting real life examples to verify and anonymous referees for comments which helped improve the quality of the paper.

References

[Atomium] Atomium Tool Suite, IMEC vzw, Belgium; `http://www.imec.be/atomium`

[Banerjee 1993] Banerjee, U.: "Loop Transformations for Restructuring Compilers: The Foundations"; Kluwer Academic Publishers (1993).

[Banerjee 1994] Banerjee, U.: "Loop Parallelization"; Kluwer Academic Publishers (1994).

[Barthou et al. 2002] Barthou, D., P. Feautrier, X. Redon: "On the Equivalence of Two Systems of Affine Recurrence Equations" (Research Note); Proceedings of 8th International Euro-Par Conference, Germany. B. Monien and R. Feldmann (Eds.), LNCS 2400, Springer-Verlag (2002), 309-313.

[Bultan et al. 1997] Bultan, T., R. Gerber, W. Pugh: "Symbolic Model Checking of Infinite State Systems Using Presburger Arithmetic"; Proceedings of 9th International Conference on Computer Aided Verification (CAV), Israel. Orna Grumberg (Ed.), LNCS 1254, Springer-Verlag (1997), 400-411.

[Catthoor et al. 1998] Catthoor, F., S. Wuytack, E. de Greef, F. Balasa, L. Nachtergaele, and A. Vandecappelle: "Custom Memory Management Methodology - Exploration of Memory Organization for Embedded Multimedia System Design"; Kluwer Academic Publishers (1998).

[Claesen et al. 1992] Claesen, L., M. Genoe, E. Verlind, F. Proesmans, H. de Man: "SFG-Tracing: A Methodology of Design for Verifiability"; In "Correct Hardware Design Methodologies", P. Prinetto and P. Camurati (Eds.), North-Holland (1992), 187-202.

[Cousot and Cousot 2001] Cousot, P., R. Cousot: "Verification of Embedded Software: Problems and Perspectives"; Proceedings of the 1st International Workshop on Embedded Software (EMSOFT), USA. LNCS 2211, Springer-Verlag (2001), 97-113.

[Čupák et al. 1998] Čupák, M., F. Catthoor, H. de Man: "Efficient functional validation of system level loop transformations for multimedia applications"; Proceedings of 3rd International Workshop on High Level Design Validation and Test (HLDVT), USA. IEEE Press (1998), 72-79.

[Feautrier 1988] Feautrier, P.: "Array Expansion"; Proceedings of the International Conference on Supercomputing, France. ACM Press (1991), 429-441.

[Feautrier 1991] Feautrier, P.: "Dataflow analysis of array and scalar references"; International Journal of Parallel Programming, 20, 1 (1991), 23-53.

[Goos and Zimmermann 1999] Goos, G., W. Zimmermann: "Verification of Compiler"; In "Correct System Design", E.-R. Olderog and B. Steffen (Eds.), LNCS 1710 Springer-Verlag (1999), 201-230.

[Goos 2001] Goos, G.: "Issues in Compiling"; Journal of Universal Computer Science, 7, 5 (2001), 410-419.

[Kelly et al. 1996a] Kelly, W., V. Maslov, W. Pugh, E. Rosser, T. Shpeisman, D. Wonnacott: "The Omega Calculator and Library Version 1.1.0"; University of Maryland (1996) Available from: `http://www.cs.umd.edu/projects/omega`

[Kelly et al. 1996b] Kelly, W., W. Pugh, E. Rosser, T. Shpeisman: "Transitive Closure of Infinite Graphs and its Applications"; International Journal of Parallel Programming, 24, 6 (1996), 579-598.

[Mateev et al. 2001] Mateev, N., V. Menon, K. Pingali: "Fractal Symbolic Analysis"; Proceedings of the International Conference on Supercomputing, Italy. ACM Press (2001), 38-49.

[Moskewicz et al. 2001] Moskewicz, M.W., C. F. Madigan, Y. Zhao, L. Zhang, S. Malik: "Chaff: Engineering an Efficient SAT Solver"; Proceedings of the 38th Design Automation Conference (DAC), USA. ACM Press (2001), 530-535.

[Necula 2000] Necula, G. C.: "Translation Validation for an Optimizing Compiler"; Proceedings of the ACM Conference on Programming Language Design and Implementation (PLDI), Canada. ACM Press (2000), 83-95.

[Oppen 1978] Oppen, D.: "A $2^{2^{2^{pn}}}$ upper bound on the complexity of Presburger arithmetic"; Journal of Computer and System Sciences, 16, 3 (1978), 323-332.

[Pnueli et al. 1999] Pnueli, A., M. Siegel, O. Shtrichman: "Translation Validation: From SIGNAL to C"; Proceedings of Conference on Correct System Design, E.-R. Olderog and B. Steffen, (Eds.), LNCS 1710, Springer-Verlag (1999), 231-255.

[Pugh 1992] Pugh, W.: "The Omega test: a fast and practical integer programming algorithm for dependence analysis"; CACM, 35, 8 (1992), 102-114.

[Quilleré and Rajopadhye 2000] Quilleré, F., S. Rajopadhye: "Optimizing memory usage in the polyhedral model"; ACM TOPLAS, 22, 5 (2000), 773-815.

[Ramalingam and Reps 1989] Ramalingam, G., T. Reps: "Semantics of program representation graphs"; Technical Report, TR-900, CS Dept., University of Wisconsin-Madison, USA. (1989).

[Samsom et al. 1993] Samsom, H., L. Claesen, H. de Man: "SynGuide: An environment for doing interactive correctness preserving transformations"; Proceedings of 6th Conference on VLSI Signal Processing, L. Eggermont et al. (Eds.), IEEE Press (1993), 269-277.

[Samsom et al. 1995] Samsom, H., F. Franssen, F. Catthoor, H. de Man: "System level verification of video and image processing specifications"; Proceedings of the 8th International Symposium on System Synthesis (ISSS), USA. (1995), 144-149.

[Shashidhar et al. 2002] Shashidhar, K.C., M. Bruynooghe, F. Catthoor, G.Janssens: "Geometric Model Checking: An Automatic Verification Technique for Loop and Data Reuse Transformations"; Proceedings of 1st International Workshop on Compilers Optimization Meets Compiler Verification (COCV), Grenoble, France. In Electronic Notes in Theoretical Computer Science (ENTCS), Elsevier Science, 65, 2 (2002).

[Thiele and Arzt 1993] Thiele, L., U. Arzt: "On the synthesis of massively parallel architectures"; International Journal of High Speed Electronics, 2, 4 (1993), 99-131.

[Tsichritzis 1970] Tsichritzis, D.: "The Equivalence Problem of Simple Programs"; Journal of the ACM, 17, 4 (1970), 729-738.

[van Aelten et al. 1994] van Aelten, F., J. Allen, S. Devadas: "Event-Based Verification of Synchronous, Globally Controlled, Logic Designs Against Signal Flow Graphs";

IEEE Transactions on CAD of Integrated Circuits and Systems, 13, 1 (1994), 122-134.

[van Engelen and Gallivan 2001] van Engelen, R.A., K. A. Gallivan: "An Efficient Algorithm for Pointer-to-Array Access Conversion for Compiling and Optimizing DSP Applications"; Proceedings of the International Workshop on Innovative Architectures for Future Generation High-Performance Processors and Systems (IWIA), USA. IEEE Press (2001), 80-89.

[Wolf and Lam 1991] Wolf, M. E., M. S. Lam: "A Loop Transformation Theory and an Algorithm to Maximize Parallelism"; IEEE Transactions on Parallel and Distributed Systems, 2, 4 (1991), 452-471.

[Wolfe 1996] Wolfe, M. J.: "High Performance Compilers for Parallel Computing"; Addison-Wesley Publishing Company (1996).

[Yang et al. 1989] Yang, W., S. Horwitz, T. Reps: "Detecting program components with equivalent behaviors"; Technical Report, TR-840, CS Dept., University of Wisconsin-Madison, USA. (1989).
