# Supplementary material for: In-text citation’s frequencies-based recommendations of relevant research papers
Source: PeerJ Comput Sci. 2021 Jun 4;7:e524. doi: 10.7717/peerj-cs.524 (PMC8189020; doi:10.7717/peerj-cs.524)
Supplement: Supplemental Information 2 — These articles are available under a CC BY-ND 4.0 license. [file peerj-cs-07-524-s002.zip › Dataset/Papers Dataset.zip!/Papers Dataset/jucs_299.pdf]

Journal of Universal Computer Science, vol. 9, no. 8 (2003), 813-828
submitted: 24/2/03, accepted: 30/5/03, appeared: 28/8/03 *© J.UCS*

Lazy Cyclic Reference Counting

Rafael Dueire Lins
Universidade Federal de Pernambuco, Recife, PE, Brazil
rdl@ee.ufpe.br

Abstract: Reference counting is a widely employed memory management technique, in which garbage collection operations are interleaved with computation. Standard reference counting has the major drawback of being unable to handle cyclic structures. This paper presents an important optimisation to a recently published algorithm for cyclic reference counting. Proofs of the correctness of the original and lazy algorithms are provided, together with performance figures.

Keywords: Garbage Collection, Reference Counting, Cycles, Memory Management
Categories: D.4.2, D.4.3, F.2.2

1 Introduction

Reference counting performs memory management in small steps interleaved with computation. It is the memory management technique of most widespread use today [Bacon 01]. Reference counting has shown to be efficient in concurrent strongly coupled architectures [Bacon 01a, Jones 96, Lins 03], and has also great potential for application in distributed environments [Lins 02a]. In reference counting each data structure keeps the number of external references (or pointers) to it. It was developed by Collins [Collins 60] to avoid user process suspension provoked by the mark-scan algorithm in LISP [McCarthy 60]. In 1963, J.H.McBeth [McBeth 63] noticed that reference counting was unable to reclaim cyclic structures, because the counter of cells on a cycle never drops to zero, causing a space-leak.

Reference [Martinez 90] describes the first widely acknowledged general solution for cyclic reference counting. The Martinez-Wachenchauzer-Lins algorithm performs a local mark-scan whenever a pointer to a shared data structure is deleted. In a recent paper [Lins 02], the constant of linearity of the algorithm in [Martinez 90] was reduced from $3\Theta(n)$ to $2\Theta(n)$, where n is the size of the sub-graph below the deleted (shared) pointer. This gain in performance was made possible by introducing a data structure, called the *Jump_stack*, which stores a reference to the "critical points" in the graph while performing the local marking (after the deletion of a pointer to a shared cell). These nodes are revisited directly, saving a whole scanning phase in [Lins 92, Martinez 90]. The work reported in reference [Salzano 02] makes the use of the Jump_stack more efficient.

Previous work [Lins 92] has shown that delaying the mark-scan by storing a reference to a shared deleted pointer for later analysis largely increases the performance of the algorithm as a whole. A convenient control strategy may even reduce the number of calls to the mark-scan to only collect cycles in the case of an empty free-list [Lins 92, Lins]. This paper makes lazy the algorithm presented in

reference [Salzano 02], yielding a more efficient cyclic reference counting algorithm than its predecessors. The eager and the lazy efficient cyclic reference counting algorithms are also proved correct in this paper. Some performance figures show the gains obtained in performance with the lazy algorithm with respect to the eager one. Reference [Lins 93] presents an optimisation to the lazy algorithm described in [Lins 92], in which the local mark-scan is made more efficient by associating with each cell a creation-time stamp. The convenience of using a similar strategy in the new lazy mark-scan is also analysed herein.

2 Standard Reference Counting

For a matter of simplicity and completeness of presentation this section revises the classical algorithm of Collins for standard reference counting [Collins 60]. In reference counting each data structure or *cell* has a count that stores the number of external references to it. Free cells are linked together in a structure called *free-list*. A cell *B* is *connected* to a cell *A* ($A \rightarrow B$), if and only if there is a pointer <*A, B*>. A cell *B* is *transitively connected* to a cell *A* ($A \xrightarrow{*} B$), if and only if there is a chain of pointers from *A* to *B*. The initial point of the graph to which all cells in use are transitively connected is called *root*.

There are three operations on the graph:

New(R) gets a cell U from the free-list and links it to the graph:

```
New (R) = select U from free-list
          make_pointer <R, U>
```

Copy(R, <S,T>) gets a cell R and a pointer <S, T> to create a pointer <R, T>, incrementing the counter of the target cell:

```
Copy(R, <S,T>) = make_pointer <R, T>
                 Increment RC(T)
```

Delete performs pointer removal:

```
Delete (R,S) = Remove <R,S>
               If (RC(S) == 1) then
                  for T in Sons(S) do
                     Delete(S, T);
                  Link_to_free_list(S);
               else Decrement_RC(S);
```

A cell T belongs to the bag Sons(S) iff there is a pointer <S,T>.

In 1963, J.H.McBeth [McBeth, 63] observed that the algorithm above is unable to collect cyclic structures, as the deletion of the last external pointer to them does not drop counters causing a space-leak.

3 Cyclic Reference Counting with an Optimised Jump-stack

The general idea of the algorithm presented in reference [Lins 02] is to perform a local mark-scan whenever a pointer to a shared structure is deleted. New and Copy remain unchanged while Delete encompasses a local mark-scan whenever a pointer to a shared structure is removed. The algorithm works in two steps. In the first step, the sub-graph below the deleted pointer is scanned, rearranging counts due to internal

references, marking nodes as possible garbage and also storing potential links to root. In step two, the cells pointed at by the links stored are visited directly. If the cell has reference count greater than one, the whole sub-graph below that point is in use and its cells should have their counts updated. In the final part of the second step, the algorithm collects garbage cells.

In addition to the information of number of references to a cell, an extra field is used to store the colour of cells. Two colours are used: green and red. Green is the stable colour of cells. All cells are in the free-list and are green to start with. The algorithm is detailed below.

The code for Delete becomes:

```
Delete (R,S) = Remove <R,S>
               If (RC(S) == 1) then
                  for T in Sons(S) do
                       Delete(S, T);
                  Link_to_free_list(S);
               else Decrement_RC(S);
                     Mark_red(S);
                    Scan(S);
```

One can observe that the only difference to standard reference counting in the algorithm above rests in the last two lines of Delete, which are explained below. The algorithm makes use of a stack, called *Jump_stack*, to store references to nodes which can potentially link the sub-graph to root. The code above for mark_red shows that it is the manager of the insertion of elements in the Jump_stack. The key optimisation proposed in [Salzano 02] is to postpone inserting cells into the Jump_stack for as long as possible. Anticipating calling mark_red recursively and only testing for multiple references to insert onto the Jump_stack afterwards yields the chance of removing a much larger number of references internal to the subgraph under analysis. This drastically reduces the number of candidates to the Jump_stack. The optimised code for mark_red is then:

```
Mark_red(S) =  If (Colour(S) == green) then
                  Colour(S) = red;
                  for T in Sons(S) do
                       Decrement_RC(T);
                  for T in Sons(S) do
                       if (Colour(T) == green)
                          then  Mark_red(T);
                       if (RC(S)>0)
                          then Jump_stack: = S;
```

Notice that the testing for insertion in the Jump_stack is performed in the parent cell. This largely reduces the space and time needed for the management of the Jump_stack with strong impact to the performance of the application. Scan(S) verifies whether the Jump_stack is empty. If so, the algorithm sends cells hanging from S to the free-list. If the Jump_stack is not empty there are nodes in the graph to be analysed. If they are red and their reference count is greater than one, there are external pointers linking the cell under observation to root and counts should be restored from that point on, by calling the ancillary function Scan_green.

```
Scan(S) =  If RC(S)>0 then Scan_green(S); Empty_JS
           else
              While (Jump_stack ≠ empty) do
              T = top_of_Jump_stack;
             Pop_Jump_stack;
             If (Colour(T) == red && RC(T)>0)
             then Scan_green(T);
             Collect(S);
```

Procedure Scan_green restores counts and paints cells green in a sub-graph in use, as follows,

```
Scan_green(S) = Colour(S) = green
                for T in Sons(S) do
                    increment_RC(T);
                    if colour(T) is not green
                    then Scan_green(T);
```

Collect is the procedure in charge of returning garbage cells to the free-list, painting them green and setting their reference count to one, as follows:

```
Collect(S) =  If (Colour(S) == red) then
                 for T in Sons(S) do
                    Remove(<S, T>);
                    RC(S) = 1;
                    Colour(S) = green;
                    free_list = S;
                    if (Colour(T) == red) then
                       Collect(T);
```

The example below, makes clearer the dynamics of the algorithm.

3.1 Example

Attention is focused on the case of Delete, as operations New and Copy remain unchanged from the standard reference counting algorithm and only Delete manages the Jump_stack. The graph presented on the lefthand side of the figure below is exactly the minimal structure for which Salkild [Salkild 87] discovered the error in Brownbridge's algorithm [Brownbridge 85]. In this example, the deletion of a pointer will not cause the recycling of any cell, because of sharing. Consider the deletion of pointer <a,b>.

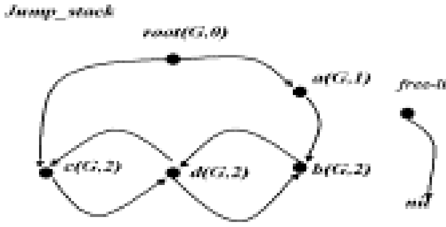

Delete(a,b) is invoked to Remove <a,b>, Decrement_RC(b), and Mark_red(b) starts yielding the graph below:

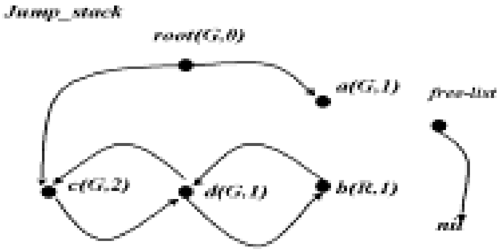

The graph above has pointer <a,b> deleted causing mark_red to be called on b, leaving the graph as,

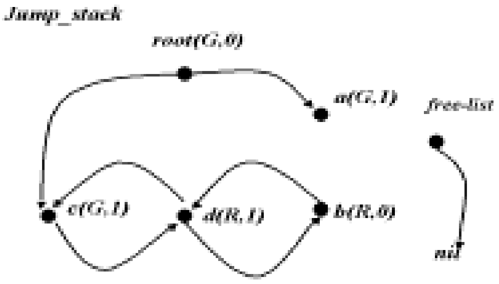

While in the original algorithm [Lins 02] a reference to d would already have been placed in the Jump_stack, in the optimised algorithm [Salzano 02] the Jump_stack remains empty so far.

Mark_red is invoked on d and c leaving the graph in the lay-out presented below,

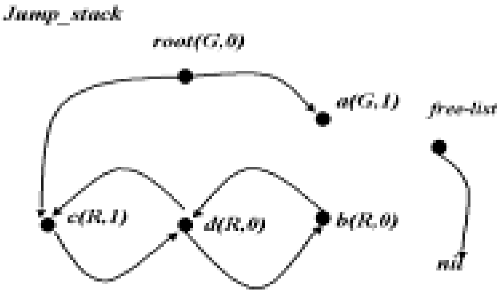

Finished the recursive calls to mark_red the testing of the condition is made and only then a reference is placed onto the Jump_stack as pictured below:

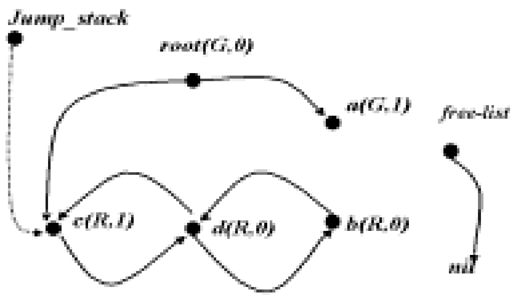

Finally, during scan, scan_green is invoked yielding the graph below, ending the local mark-scan.

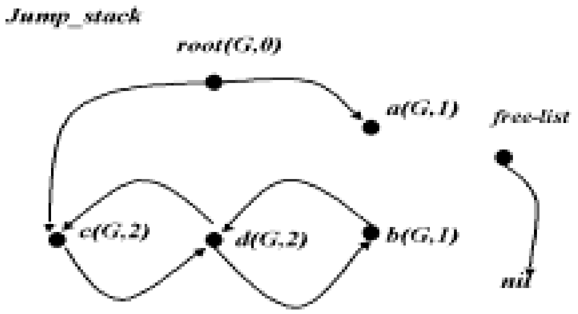

4 The lazy algorithm

The new lazy algorithm presented herein postpones the local mark-scan either for as long as possible or following a pre-determined strategy, by storing a pointer to the shared cell in a control data structure *Q*, which for simplicity is assumed to be a queue. In most applications, graph rewritings happen in "regions", thus chances are that if a pointer to a shared cell is deleted there is a large probability that the remaining pointers may also be deleted. It is likely that the fate of cells is decided without needing to call the local mark-scan at all. Ideally, only pointers to cycles would remain in Q, as their count never drop to zero. In this case, mark-red is used to reclaim those cells.

In addition to the information of number of references to a cell, an extra field is used to store the colour of cells. Three colours are used: *green*, *red* and *black*. Green is the stable colour of cells. All cells are in the free-list and are green to start with. Red is a colour used to control the marking of cells. Black is used to indicate that a cell is already in Q and will have its status defined at a later stage.

The dynamics of the algorithm is detailed below:

New(R) either gets a cell U from the free-list and links it to the graph or if the free-list is empty forces a local mark-scan to take place by calling scan_Q:

```
New (R) = if free-list not empty then
                select U from free-list
                make_pointer <R, U>
             else
                if Q not empty then
                   scan_Q;
                    New (R)
                else
                   write_out "No cells available"
```

Copy(R, <S,T>) gets a cell R and a pointer <S, T> to create a pointer <R, T>, incrementing the counter of the target cell. If a cell is the target of a Copy it is painted green, as one becomes sure it is in use, avoiding later calls to the local mark-scan.

```
Copy(R, <S,T>) = make_pointer <R, T>
                  Increment RC(T);
                 Colour(T) :=green
```

Pointer removal is performed by Delete, which is here far simpler than in the eager algorithm [Lins 02], since mark-scan to multiple referenced cells is performed lazily. The colour of cells is tested black to avoid multiple references on Q.

```
Delete (R,S) = Remove <R,S>
                        If (RC(S) == 1) then
                            for T in Sons(S) do
                                  Delete(S, T);
                            Link_to_free_list(S);
                        else Decrement_RC(S);
                                if colour(S) not black then
                                  colour(S):= black;
                                  Q:= Q ++ S
```

The local mark-scan is performed by scan_Q, by electing a cell and testing its colour. If it remains black its status is still to be analysed by invoking the local mark-scan, otherwise it is simply removed from Q (either Delete sent it directly to the free-list or Copy assured its use).

```
scan_Q = S :=head(Q);
            Q := tail(Q);
             if colour(S) is black then
                            Mark_red(S);
                            Scan(S);
              else
                  if Q not empty then
                     scan_Q
```

Mark_red(S) is modifyied to allow for black cells also, thus.

```
Mark_red(S) =  If (Colour(S) ≠ red) then
                     Colour(S) : = red;
                     for T in Sons(S) do
                           Decrement_RC(T);
                     for T in Sons(S) do
                           if (Colour(T) ≠ red)
                                then  Mark_red(T);
                           if (RC(T)>0 && T not in Jump_stack)
                            then Jump_stack: = T;
```

Routines Scan, Scan_green and Collect are the same as in the eager algorithm [Lins 02], presented in section 3 above.

4.1 Example

The dynamics of the algorithm is illustrated by an example, where one can observe its advantage in relation to the eager algorithm [Lins 02, Salzano 02]. Assume a Control_queue of size 3, which behaves as a stack of references onto the graph. The graph of the figure below suffers the deletion of pointer <root, a>.

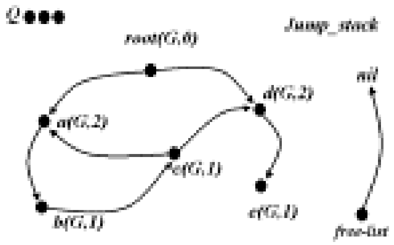

Cell a is a shared data structure within a cycle. While in the eager algorithm a local mark-scan would be automatically started, in the lazy algorithm a reference to a is placed on the Control_queue with no further action, yielding the graph as shown:

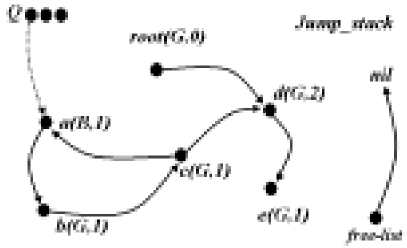

The deletion of pointer <d, e> takes place and cell e is automatically placed by Delete onto the free-list, leaving the graph as,

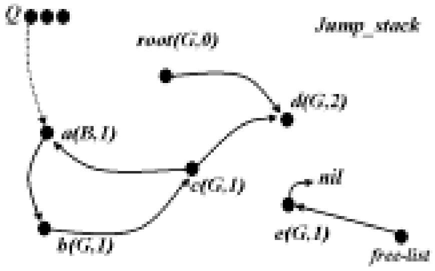

Now, the cycle abc is broken by the deletion of pointer <c, a>. Recursive calls to Delete are made, yielding the graph as depictured in the graph below.

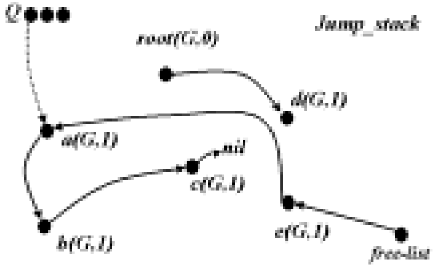

Notice that at the end of this deletion process cells are placed onto the free-list, but a reference to a remains on the Control_queue. Only calls to scan_Q will remove that reference with no further ado as it became green (it either remains on the free-list or is back to the graph in use). It is most important to stress that no call to the mark-scan process was ever made.

5 Proof of the correctness

The invariant conditions for reference counting are:

1. *The number of external references to a cell is equal to the value of the reference count.*
2. *Every cell is either transitively connected to root or is on the free-list, but not on both.*

The conditions above are analysed in standard reference counting, for each of the graph operations:

- New gets a cell from the free-list (with RC=1, observing condition *1*) and (transitively) links it to root, satisfying condition *2*.

- Copy makes a new link to a cell already transitively connected to root (thus preserving condition *2*) and increments the count of the target cell (keeping condition *1* valid).

- Delete removes a pointer to a cell and analyses its count.

  - If its count is 1 and there are no active calls to Delete, the last link to root was deleted and the cell is placed directly onto the free-list satisfying both conditions above.
  - If its count is greater than one, the reference count of the target cell is decremented (keeping condition *1* valid), but in regard to condition *2*, two cases unfold:

    i. shared subgraph – condition *2* remains valid.

    ii. cyclic subgraph – if the link removed was the last connecting to root, there is a space leak and condition *2* does not hold.

5.1 The eager algorithm

The eager cyclic algorithm restores the validity of conditions *1* and *2*, provided that there are no active function calls, by invoking Mark_red and Scan at the target cell of the deleted pointer.

- Mark_red simulates the deletion of the last link to root, placing onto the Jump_stack possible candidates (cells with RC>1) that may still keep the subgraph transitively linked to root. Notice that while Mark_red is active, condition *1* is not valid and condition *2* may not be valid (in the case of cyclic subgraph with space leak).

- Scan checks the members of the Jump_stack:

  - If any of them have reference count greater than one, there is an external link to root. Condition *2* always hold and Scan_green is invoked to restore counters within the subgraph, making condition *1* valid.

  - Cells pointed at from the Jump_stack have reference count 0 and are thus detached from root. Collect is invoked to send cells to the free-list (restoring the validity of condition *2*) and setting their reference counts one (making condition *1* hold).

Notice that no other function was considered as they remain unchanged with relation to the standard reference counting algorithm and that colours are simply marks for termination detection of recursive calls to functions.

5.2 The lazy algorithm

The lazy cyclic reference counting algorithm postpones the local mark-scan for as long as possible, in the hope of not performing it at all (in the case of a shared data structure) or to perform it only to reclaim an island of cells detached from root. Cells referred by the Control_queue are thus of uncertain status and will either be left in the graph (they are in use and linked to root) or reclaimed and sent to the free-list. The invariant conditions to the lazy algorithm may be stated as:

3. *The number of external references to a cell is equal to the value of the reference count, provided there is no outstanding function call to the routines of the lazy/local mark-scan.*
4. *Every cell is either transitively connected to root, transitively connected to the control structure or is on the free-list. No cell is both in the free-list and transitively connected to root.*

The conditions above are analysed in lazy cyclic reference counting, for each of the graph operations:

- New works now with two possibilities:
  - If the free-list is non-empty, it gets a cell from the free-list (with RC=1, observing condition *3*) and (transitively) links it to root. Condition *4* is also met.
  - If the free-list is empty and Q is non-empty, Q is scanned using a chosen control-strategy, by function scan_Q, whose behaviour is analysed further below.
- Copy makes a new link to a cell already transitively connected to root (thus preserving condition *4*) and increments the count of the target cell (keeping condition *3* valid). Painting the target cell *green* only stresses that the cell is connected to root, saving unnecessary calls to scan_Q.
- Delete removes a pointer to a cell and analyses its count.
  - If RC=1, Delete is invoked in the Sons of the cell and the original cell is sent to the free-list, keeping invariants *3* and *4* valid.
  - If RC>1 (the cell is either shared or in a cycle), it has its count decremented (condition *3* holds) and a reference to it is sent to Q (condition *4* valid).
- Scan_Q analyses the colour of the cells in the control structure:
  - if *green* this means that the cell is
    1. in use (linked to root) and was the target either of a Copy operation or a previous call to the mark-scan (conditions *3* and *4* hold).

ii. in the free-list (conditions *3* and *4* are valid).

- if black this means that it remains as when inserted in the control structure, with conditions *3* and *4* being observed. Its status is uncertain and will be analysed by Mark_red and Scan.

- Mark_red was modified only to allow for black cells, as it does not change the connectivity of the graph condition *4* holds. As in the eager version of the algorithm, it simulates internal pointer deletion, thus weakening the invariant in standard reference counting that the count stores the number of external references to it, while active.
- Scan, Scan_green and Collect work as in the eager algorithm and strengthens the validity of conditions *3* and *4* to the more restrictive versions *1* and *2*.

Thus one may see that the dynamics of the lazy algorithm keep the basic conditions above, working properly.

6 Performance Analysis

Some experimental data compares the performance of the eager and lazy algorithms herein. For that purpose, a Turner combinator graph reduction machine was built [Turner 79] to execute a simple functional language. Cycles arise from “knot-tying” the combinator “Y”, responsible for compiling recursion. The Recfat benchmark that make intensive use of sharing and graph transformation was used to produce the data presented below. Recfat generates a somatory of applications of the factorial creating 18 cycles to be reclaimed. The initial size of the graph is 394 cells and the minimal heapsize required is 413. Its execution creates 383 cells and 776 are reclaimed (included the 18 cycles). The total number of evaluations is 911.

Table 01 presents the result of execution of Recfat using the eager optimised algorithm [Salzano 02] and the lazy algorithm as presented in sections 3 and 4 of this paper, respectively. The lazy algorithm adopted a queue of fixed size as control structure *Q* (whose size is shown in parenthesis). Column *#calls* stands for the total number of function calls to the mark-scan, being equal to the sum of the columns of *M_red* (mark_red), *Scan*, *S_green* (Scan_green) and *Collect*. *Sc_Q* stands for the number of scans in *Q*, while In(Q) and Out(Q) stand for the number of insertions and removals from Q, respectively.

	M_red	Scan	S_green	Collect	#calls	Sc_Q	In(Q)	Out(Q)
Eager	2,924	418	2.630	294	6,266	---	----	----
JlazyF(1)	2.384	334	2.070	314	5.102	399	400	399
JlazyF(2)	2.168	287	1.872	296	4.623	191	384	382
JlazyF(3)	2.011	246	1.709	302	4.268	121	366	363
JlazyF(4)	1.955	220	1.651	304	4.130	85	344	340
JlazyF(5)	1.972	230	1.668	304	4.174	71	359	355
JlazyF(10)	1.554	162	1.248	306	3.270	33	333	330
JlazyF(20)	879	72	591	288	1.870	11	238	220
JlazyF(50)	678	47	408	270	1.403	4	226	200

	Sc_JStck	In(JS)	Out(JS)	EmptyJS
Eager	18	1.434	121	1,313
JlazyF(1)	18	1168	102	1066
JlazyF(2)	17	1065	99	966
JlazyF(3)	17	960	94	866
JlazyF(4)	17	921	92	829
JlazyF(5)	17	949	92	857
JlazyF(10)	17	721	90	631
JlazyF(20)	16	359	92	272
JlazyF(50)	15	245	84	161

Table 1: Performance of Recfat

One can also observe that the algorithm introduced herein drastically reduces the management of the Jump_stack. Allocating a control structure of size 20 (less then 5% of the minimum heap for the execution of the benchmark) yielded an economy of more than two-thirds of the total number of function calls.

Reference [Lins] analyses the behaviour of Recfat and other benchmarks, with different control strategies. In all of them the lazy algorithm performed much better than the eager one not only in terms of number of function calls but brought substantial gains also to wall-clock (total) time performance.

7 Variable-size control structures

The algorithm as presented above suggests that the control structure Q is implemented outside the heap as a separate data structure. Cells in the free-list may be used to implement Q, allowing a completely dynamic allocation of resources. Instead of placing a cell *S* in Q, one can get a cell *U* from the free-list, append *U* to Q, and store in *U* a pointer to *S*, thus

```
Delete (R,S) = Remove <R,S>
                     If (RC(S) == 1) then
                        for T in Sons(S) do
                            Delete(S, T);
                        Link_to_free_list(S);
                     else Decrement_RC(S);
                           if colour(S) ≠ black then
                            colour(S):= black;
                            (U  = New(last_of_Q)):=S
                            last_of_Q := U
```

If the free-list is empty New will de_queue cells from Q. Reference [Lins] reports that the adoption of the variable-size control structure for a simple benchmark, with heap of minimum size for its execution, reduced the number of function calls to 25% of the local mark-scan algorithm [Martinez 90], which is equivalent to the performance obtained by a Q of (fixed) size seven.

One may adopt a similar strategy to implement the Jump_stack by using spare cells from the free-list. Observe that in the algorithm presented above the Jump_stack only appears during Mark_red, thus provided there are enough cells in the free-list, the Jump_stack may the implemented with no cost in space.

8 Analysing the Creation-time of Cells

Reference [Lins 93] optimises the performance of the lazy mark-scan algorithm described in [Lins 92], by recording in each cell an age counter, called *AG*. For that purpose, a global time counter is needed. The time counter is initialised with zero and is incremented every time a cell is claimed from the free-list by New. If *AG(R)<AG(U)*, this means that cell *R* is older than cell *U*. In order to spell out the possibility of cycles, Mark_red checks for the condition that all parent cells are older than their sons. If this condition is true the subgraph under analysis is acyclic. This information is stored in a new global variable called no_cycles.The certain absence of cycles allows Scan to either send garbage cells directly to the free-list or restore their original status by invoking Scan_green. The age counter may save a pass through the subgraph at the cost of having to store a large counter in each cell for the creation-time stamp. Notice that rewritings may weaken the "well-ordering" of age counters forcing no_cycles to hold a value *false* even in the absence of cycles. Thus forcing a mark-scan with complexity 3Θ to take place, where Θ is the size of the subgraph below the deleted pointer.

The algorithm presented herein dismisses the need of age counters to obtain a more general solution with no need for extra space in cells to store a creation-time stamp. The Jump_stack visits the critical points of the graph directly, keeping in all cases the complexity of the local mark-scan in 2Θ, where Θ is the size of the subgraph under analysis.

9 Conclusions

The lazy algorithm presented herein largely optimises the algorithm for cyclic reference counting [Lins 02, Salzano 02], by avoiding calls to the local mark-scan. The introduction of the Jump_stack and control structure have drastic impact in the performance of cyclic reference counting acting in almost orthogonal ways. The control structure Q avoids, as much as possible, executing the local mark-scan algorithm. On the other hand, the Jump_stack allows visiting the critical points of the subgraph under observation directly, avoiding an extra pass performed by the algorithms presented in references [Martinez 90, Lins 92]. Thus, one can see that the new algorithm reduces the constant of linear complexity from $3O(n)$ to $2O(n)$, where n is the size of the sub-graph below the deleted (shared) pointer. This is obtained without the need for creation-time stamps and this lower complexity bound is obtained for all subgraphs, being more general and lower cost in time and space than the solution presented in the Generational cyclic reference counting algorithm [Lins 93].

The space complexity is increased in relation to the algorithm presented in [Lins 02], as two bits are needed to store the three colours for algorithm markings and a global data structure is needed for postponing the sub-graph analysis.

Different control strategies may be adopted according to the nature of the application allowing to reduce the number of calls to the local mark-scan. Several of them are proposed and benchmarked in reference [Lins], widening and updating the data reported herein. Recent work developed at IBM T.J.Watson by Bacon and Rajan [Bacon 01] in the context of the Jalapeño JAVA virtual machine [Bacon 01a]

implements Lins' lazy cyclic algorithm using a strategy of running each phase of the mark-scan of each element of the control structure (similarly to the synchronisation barriers introduced for multi-processors in [Lins 03]). Reference [Bacon 01] reports that using a set of eleven benchmark programs including the full SPEC benchmark suite, the Jalapeño garbage collector achieves maximum measured application pause times about two orders of magnitude shorter than the best previously published results and performance similar to a highly tuned non-concurrent but parallel mark-and-sweep garbage collector. It is reasonable to suppose that the implementation of the garbage collection algorithm presented herein in the Jalapeño machine may provide even better performance figures.

Acknowledgements

The author is indebted to J.A.Salzano Filho for his comments.
This work was sponsored by CNPq and Recope-Finep, to whom the author is greatful.

References

[Bacon 01] D.F.Bacon and V.T.Rajan. Concurrent Cycle Collection in Reference Counted Systems, Proceedings of European Conference on Object-Oriented Programming, June, 2001, Springer Verlag, LNCS vol 2072.

[Bacon 01a]D.F.Bacon, C.R.Attanasio, H.B.Lee, R.T.Rajan and S.Smith. Java without the Coffee Breaks: A Nonintrusive Multiprocessor Garbage Collector, Proceedings of the SIGPLAN Conference on Programming Language Design and Implementation, June, 2001 (SIGPLAN Not. 36,5).

[Brownbridge 85] D.R.Brownbridge. Cyclic reference counting for combinator machines, FP&CA'85, LNCS 201, pp. 273-288, Springer Verlag, 1985.

[Collins 60] G.E. Collins, A method for overlapping and erasure of lists, Comm. of the ACM, 3(12):655—657, Dec.1960.

[Jones 96] R.E. Jones and R.D. Lins, Garbage Collection Algorithms for Dynamic Memory Management, John Wiley & Sons, 1996. (Revised edition in 1999.)

[Lins 92] R.D.Lins, Cyclic Reference counting with lazy mark-scan, IPL 44(1992) 215—220, Dec. 1992.

[Lins 93] R.D.Lins, Generational cyclic reference counting, IPL 46(1993) 19—20, 1993.

[Lins 02] R.D.Lins. An Efficient Algorithm for Cyclic Reference Counting, Information Processing Letters, vol 83 (3):145—150, August 2002.

[Lins 02a] R.D.Lins. Efficient Cyclic Weighted Reference Counting, in Proc. of 14th Symp. on Computer Architecture and High Performance Computing, IEEE Press, October 2002.

[Lins 03] R.D.Lins. An Efficient Multi-processor Architecture for Parallel Cyclic Reference Counting, Proceedings of VECPAR'2002, pp. 650—663, LNCS 2565, Springer Verlag, 2003.

[Lins] R.D.Lins, Analysing the Performance of Cyclic Reference Counting Algorithms, in preparation.

[Salzano 02] J.A.Salzano Fº & R.D.Lins, Optimising the Jump_Stack, in Proceedings of SBLP'2002, pp 233—242, June 2002.

[McBeth 63] J.H. McBeth, On the reference counter method, Comm. of the ACM, 6(9):575, Sep. 1963.

[Martinez 90] A.D. Martinez, R. Wachenchauzer and R.D. Lins, Cyclic reference counting with local mark-scan, IPL 34(1990) 31—35, North Holland, 1990.

[Salkild 87] J.D.Salkild. Implementation and Analysis of two Reference Counting Algorithms. Master thesis, University College, London, 1987.

[Turner 79] D.A.Turner. A new implementation technique for applicative languages. Software Practice and Experience, Vol 9, pp 31-49, 1979.
