# Supplementary material for: In-text citation’s frequencies-based recommendations of relevant research papers
Source: PeerJ Comput Sci. 2021 Jun 4;7:e524. doi: 10.7717/peerj-cs.524 (PMC8189020; doi:10.7717/peerj-cs.524)
Supplement: Supplemental Information 2 — These articles are available under a CC BY-ND 4.0 license. [file peerj-cs-07-524-s002.zip › Dataset/Papers Dataset.zip!/Papers Dataset/jucs_53.pdf]

Journal of Universal Computer Science, vol. 11, no. 2 (2005), 250-270
submitted: 31/7/04, accepted: 15/12/04, appeared: 28/2/05 *© J.UCS*

Consensus-Based Hybrid Adaptation of Web Systems User Interfaces

Janusz Sobecki
(Institute of Applied Informatics, Wroclaw University of Technology
Wyb.Wyspianskiego 27, 50-370 Wroclaw, Poland
sobecki@pwr.wroc.pl)

Abstract: Consensus methods proved to be very effective in solving problems in many areas. In this paper a hybrid adaptation of web-based system user interfaces that uses consensus methods is presented. The hybrid recommendation is a combination of the following methods: demographic, content-based, and collaborative. Each of this method has its specific advantages and disadvantages. The hybrid adaptation enables overcoming disadvantages of each separate solution.

Keywords: User Interfaces, Hybrid Adaptation, Web-based systems, Consensus Methods
Categories: H.3.5, H.5.2, H.5.3, H.5.4

1 Introduction

The applications of the consensus methods may be found primarily in the area of social and sociological sciences. These methods have been mainly used for standardization and an agreement determination in solving conflicts [Day 88]. The consensus methods are particularly useful for such conflict systems in which different opinions and uncertainty of information is assumed however the decision making process is required.

Nowadays consensus methods are applied in many areas of computer and information science, for example in database information reconciliation, information retrieval or agent systems [Nguyen 02]. In works [Nguyen and Sobecki 03] and [Sobecki 04] conception and implementation of consensus methods applied in collaborative adaptation of the user interfaces were shown. In this paper consensus methods are applied in hybrid adaptation of user interfaces of web-based systems.

Different types of recommender systems [Montaner et al. 03] are nowadays gaining popularity among internet systems providers owing to their ability to deliver customized information for their users. Recommender systems may be applied in many areas and depending on the application field may be called adaptive or personalized user interfaces [Kobsa et al. 01] or interface agents [Lieberman 97].

An interface agent is defined by P. Maes as an agent that acts as a kind of intelligent assistant to a user with respect to some computer application [Wooldridge and Jennings 95] or as mediator between the human and the cyberspace and are able to personalize the interface by monitoring and sensing users' capabilities [Arafa and Mamdani 00].

Montaner in [Montaner et al. 03] distinguishes three types of recommendations: demographic, content-based and collaborative. All of the recommendation methods

are based on the user model that is usually built of the two elements: the user data and the usage data [Kobsa et al. 01]. The user data contains information on demographic data, users' knowledge, their skills and capabilities, their interests and preferences and also their plans and goals. The second element of the user model, the usage data, is built out of the observation during the user's interactions with web-based systems. The usage data may concern selective operations that express users' interests, unfamiliarity or preferences, temporal viewing behavior, as well as ratings concerning the relevance of these elements.

The user model is used by the recommendation process according to the implemented approach. Demographic filtering [Montaner et al. 03] takes descriptions of people from the user profile to learn the relationship between a particular item and the type of people who like it. Content-based filtering uses descriptions of the content of the items to learn the relationship between a single user and the description of the items according to the user usage description. Finally, the collaborative filtering uses the feedback from a set of somehow similar people concerning a set of items in order to make recommendations.

In this paper, the hybrid architecture of web-based system user interface with application of consensus methods is presented. The proposed architecture is based on the solution that there is some combination of collaborative filtering with elements of demographic recommendation solution presented in [Nguyen and Sobecki 03] and [Sobecki 04] with some elements of content-based approach.

In [Section 2] the consensus theory basics together with conflict systems, conflict situation and consensus determination is presented. In [Section 3] the demographic recommendation of user interfaces and its applications is described. The following [Section 4] describes collaborative filtering applied for user interface adaptation that uses consensus methods for the determination of the recommendation. The collaborative recommendation presented in [Section 4] is based on works [Nguyen and Sobecki 03] and [Sobecki and Weihberg 04]. In [Section 5] some proposition of content-based recommendation and also application of consensus methods for reconciliation of the final recommendation of a particular attribute values are described. In [Section 6] the hybrid recommendation that is combination of the former methods is presented. The last [Section 7] concludes the paper and shows perspectives for the future works.

2 The Model of Consensus

Consensus theory has its general origins in the social sciences and in the theory of choice in particular [Daniłowicz et al. 02]. The social choice theory considers problems of analyzing a decision between a collection of alternatives made by a collection of different voters with separate opinions and the selected choice should reflect the desires of all the individual voters to the possible extent [Social, 04]. The main difference between consensus theory and the choice theory is that the former one does not necessitates the solution belonging to the set of opinions under consideration.

The consensus theory concerns mainly two problems [Barthelemy 88]. First, searching for a latent structure of any object and second, reconciling disagreeing data of the same object. In the first case the data versions reflect an unknown structure

what cause measurement problems, information loss and errors in evaluation criteria. In the second case the data versions differ from each other because they are obtained in different ways (observations, experiments etc.), and the proper version is unknown and consensus problem may be considered as the alternatives ranking problem or committee election problem [Nguyen 01].

In the model of consensus presented here the attribute paradigm will be assumed, so the data versions representing the conflicting content are built by means of some universe of tuples representing potential objects or events etc. The tuples are represented by a set of different attributes and their values, each of which is a set of elementary values. In other words, we use multi-valued attributes to represent data versions. One should underline that the attribute paradigm is very convenient for describing the real world objects, and is very often used. It even seems to be irreplaceable in database models, from hierarchical to object-oriented ones, or in knowledge representation.

2.1 Basic Notions in the Consensus Model

Within the consensus model it is assumed that a real world domain is described by means of a finite set A of attributes and a set V of attribute *elementary values* [Nguyen 02]. We can also define the following notions:

1. Let $B \subseteq A$, a tuple of type B is a function r_B: $B \rightarrow \Pi(V_B)$ where $(\forall b \in B)(r_b \subseteq V_b)$
2. The set of all tuples of type B is denoted by $TYPE(B)$
3. A tuple is elementary if all attribute values are empty sets or 1–element sets
4. The set of elementary tuples of type B is denoted by $E\text{-}TYPE(B)$.
5. An empty tuple which values are all empty sets is denoted by the symbol ϕ
6. A partly empty tuple with at least one empty value is denoted by the symbol θ.
7. A non–empty set R of tuples of type B is called a relation of type B, thus $R \subseteq TYPE(B)$.
8. A sum of 2 tuples r and r' of type B is a tuple r'' of type B ($r''=r \cup r'$) such that $(\forall b \in B)(r''_b=r_b \cup r'_b)$.
9. A product of 2 tuples r and r' of type B is also a tuple r'' of type B ($r''=r \cap r'$) such that $(\forall b \in B)(r''_b=r_b \cap r'_b)$.
10. We say that tuple r is included in tuple r' ($r \prec r'$), where $r,r' \in TYPE(B)$, iff $(\forall b \in B)(r_b \subseteq r'_b)$.
11. We say that relation R of type B is a projection of a relation R' of type B' ($\Pi^B(R')$) if $B \subseteq B'$ and $(\forall b \in B)(r_b=r'_b)$.
12. We say that B is a key of relation R (*key(R)*) if its values identifies each tuple from the relation R uniquely.

2.2 Definition of Conflict System

In the scope of the interest of this paper, i.e. the hybrid adaptation of a web-based system user interface, as the source of opinions we may assume each client in the distributed system, that could be also called agents, or different events of the specified user client. The subjects of agents' interest consist of events occurring in the world, i.e. mainly observing user behavior, interface settings and its usability values. These

observations are called events and are stored as attribute values by a tuple of some type. The conflict system definition is a modification of the one presented in the work [Nguyen and Sobecki 03]. Here the notion of the category is introduced for differentiation of the recommendation type: demographic, collaborative and content-based. The second difference relies on the identification of the information sources. In the conflict system defined in [Nguyen 02] agents are the source of different (conflict) information. The conflict system defined here as the source of conflict information identifies not only separate agents but also depending on the category also different events from the given agent. We can distinguish some subset $T \subseteq A$ that contains attributes for event identification.

Definition 1.
A conflict system of some category c is a quadruple: $S^c = (A, X, P, Z)$, *where:*

- *A – is a finite set of attributes (as defined above), including attributes that identify each event.*
- *X – is a finite set of conflict carriers,* $X = \{\Pi(V_a): a \in A\}$.
- *P – is a finite set of relations on carriers from X, each relation is of some type L (for* $L \subseteq A$ *and L contains attribute or attributes that enable to identify the observation from set* $T \subseteq L$*).*
- *Z – is a finite set of logic formulas for which the model is a relation system (X,P).*

Relations belonging to set *P* are classified in such a way that each of them includes relations representing similar events. In our case these observations and events concerns the interface settings and some other attributes that identify the observed event and also the attributes that have an influence on the interface settings. Some examples of the conflict systems will be shown in the following sections.

2.3 Conflict Profiles

A conflict situation for a given category c of the conflict system S^c contains information about a concrete conflict as follows:

Definition 2.
A conflict situation of a given category c of the conflict situation cs^c *is a pair* $<P, Y \rightarrow B>$, *where Y is a set of attributes that have influence on the interface settings:* $Y \subseteq L \backslash T$ *and* $B \subseteq L \backslash T$ *and* $Y \cap B = \emptyset$ *and* $r_Y \neq \theta$ *for every tuples* $r \in P$.

A conflict situation consists of event identifiers (conflict body) which appear in relations *P* (conflict content) representing the observed (or induced) knowledge of referring to subjects represented by set *B* of attributes, in this case interface settings. Expression $Y \rightarrow B$ means that in the observed events there are differences referring to combinations of values of attributes from *Y* with values of attributes from *B*, and the purpose of the consensus choice is that for a tuple type *Y* at most one tuple of type *B* should be assigned.

For a given situation cs^c, we determine the set of events which take part in the conflict as the projection of the set of relations P to the set of attributes K, Event(cs^c) = $\Pi^K(P)$, where $K \subseteq A$, and K is a key of relation P. Then the set of subject elements (or subjects for short) is defined as the projection of the set of relations P to the set of attributes Y Subject(cs^c) = $\Pi^Y(P)$ where $Y \subseteq L \backslash T$.

We should notice that Event(cs^c) contains relations that identify them and Subject(cs^c) contains relations that are subjects of the event which have interface settings as an object. In other words we can say that Subject(cs^c) determines the values of attributes from the set B. Now for each subject $e \in$ Subject(cs^c) let us determine set with repetitions Profile(e) which include knowledge from events on subject $e \in Subject(cs^c)$, as the set of relations that identify the given subject e reduced to the set of attributes $B \cup K$ of for and they are included Profile(e)=$\{r_{B \cup K}$: $(r \in P) \wedge (e \prec r_A)\}$.

The examples of Event(cs^c), Subject(cs^c) and Profile(e) for each category will be shown in the following sections.

2.4 Consensus Definition and Determination

Below the definition of consensus is presented, its idea is based on the consensus definition given in [Nguyen, 02].

Definition 3.

Consensus on subject $e \in Subject(cs^c)$ of situation cs^c=<P,$Y \rightarrow B$> is a tuple ($C(cs^c,e)$) where $C(cs^c,e) \in TYPE(Y \cup B)$ that fulfils logic formulas from set Z and one of the following postulates are fulfilled:

P1. $$C(cs^c,e)_B \prec \bigcup_{r \in Profile(e)} r_B$$

This postulate called *knowledge closure* states that the consensus should be included in the sum of profile elements.

P2. $$\bigcap_{r \in Profile(e)} r_B \prec C(cs^c,e)_B .$$

This postulate requires the knowledge consistency, meaning that the common part of profile elements should be included in the consensus.

P3. *For* $cs^{c\prime}$ = <P', $Y \rightarrow B$> *and* $cs^{c\prime\prime}$ = <P'', $Y \rightarrow B$>
if $C(cs^{c\prime},e)_B \cap C(cs^{c\prime\prime},e)_B \neq \theta$
then $C(cs^c,e)_B = C(cs^{c\prime},e)_B \cap C(cs^{c\prime\prime},e)_B$
where cs^c=<$P' \cup P''$, $Y \rightarrow B$>.

This postulate P3 is the Condorcet consistency condition for choice.

P4. *For $x \in Profile(e)$ and $x_B \neq C(cs^c,e)_B$ there exists a natural number n such that $x = C(cs^{c\prime},e)_B$ where s' is a situation with the same subject $Y \rightarrow B$, in which $Profile'(e) = Profile(e) \cup \{n*x\}$.*

Postulate P4 states that if a tuple x is not a consensus of a profile, then it should be a consensus of a new profile including the old profile and a sufficient number of tuples x.

The following theorem should enable to determine a consensus that satisfies all the postulates **P1**-**P4**. The proof of this theorem is some modification of the one presented in the work [Nguyen 00].

Theorem 1. *If there is a defined distance function δ between tuples of TYPE(B), then for a given subject e of situation cs^c=<P,Y→B> tuple C(cs^c,e) which satisfies conditions of Definition 3 and minimize the expression* $\sum_{r\in Profile(e)} \delta(r_B, C(cs^c, e)_B)$ *should create a consensus satisfying all postulates* ***P1-P4****.*

2.5 Algorithms for Consensus Determining

In case of all the attributes being independent then the consensus determination in the Profile(*e*) is reduced to the determination of consensus for each attribute in the tuple of *TYPE(B).*

Depending on the microstructure of attribute values such as 1-element sets or sets of values and of objects and macrostructure of their universe (distance function definition) different algorithms for consensus determination could be distinguished.

The simplest microstructure of an attribute $a\in A$ is represented by 1-element sets of values from some the set V_a, there should be also determined the distance function values in this set. Then the consensus determination from the profile based on the consensus choice function from the *Theorem 1* is performed by selection of that value from the profile that some of distances to the rest elements of the profile is minimal.

In case of other microstructures such as number intervals, rankings and sets the algorithms for consensus are more complicated and they can be found in work [Nguyen and Sobecki 03].

3 User Model

In several previous works [Nguyen and Sobecki 03], [Sobecki 03], [Sobecki and Weihberg 04] the user model was divided into two parts : the user profile that contains user data (mainly demographic data) delivered by the user and the interface profile that is designated by the system and may be changed by the user during the personalization process. In this paper, however, the user model like in many other works, for example [Kobsa et al. 01], will contain the whole data.

In the recommendation systems many different profile representations are applied: history of purchases, web navigation or e-mails; an indexed vector of features; a n-gram; a semantic network; an associative network; a classifier including neural networks, decision trees, inducted rules or Bayesian networks; a matrix of ratings and a set of demographic features, tuples and tree structure [Montaner et al. 03], [Sobecki 02]. The user profile may also be initialized in several different ways, manually as well as automatically. The later method may be further divided in at least few types.

3.1 Profile Representation

The easiest, hence very useful, ways to of representing the user profile is registration of the user actions during the interaction with different web based systems (usage data). These actions may be following: user purchases made on the web, visited pages or e-mail's that have been read or sent by the user.

The purchase list may characterize the user's marketing preferences because users are usually purchasing goods that they are personally interested in (with some exceptions). The other type of profile representation is web navigation history that contains the URL list of visited pages. For the specified system when it is exactly known what the particular page contains it could be sufficient to remember only its identifier (URL) to know the information content from the URL only. However usually the URL itself does not bring too much information and sometimes could be even misleading, because of the fact that the same pages may have different URL's. To be more precise the recommendation system remembers words from the visited pages but in this case other representations, such as feature vectors are used. Finally the from field of e-mails or news may identify the person or organization that sent the message, so by having e-mail history it is possible to recommend actions to be taken with new e-mails.

In the vector model all profiles are represented as a vector of features [Montaner et al. 03]. The feature vector is defined as $v=(f_1, f_2,\dots ,f_n)$ where f_i is the value (real number) of the feature $i,\forall i\in\{1,2,..,n\}$. In the work [Basili et al. 03] the following properties of the feature vector representation were given: no explicit relations between features are to be foreseen (a-priori independence); no hierarchy of the values is considered (flatness of the set of set of features); only one value is admitted for each feature (certainty of the observation). The weighted feature vector modifies the ordinary feature vector with some weights that may modify the importance of the specified features in the whole vector. The vector of weights $w=(w_1,w_2,\dots w_n)$ modifies the standard feature vector in the following way: $v=(w_1{*}f_1, w_2{*} f_2,\dots ,w_n{*}f_n)$. The vector representation is very popular among many recommendation systems, they were used by the following systems: Letizia [Lieberman 97], MovieLens, Webmate, WebSail and WebWatcher.

The profile may also be represented as a tuple of values that are defined in the following way: the finite set of the profile attributes A and the set V that contains attribute values, where: $V=\cup_{a\in A}V_a$ (V_a is the domain of attribute a). The tuple t is a function $t{:}A\rightarrow V$ where $\forall a\in A(t(a)\in V_a)$. This type of representation could be also called as a single valued information system that was introduced in work [Pawlak, 81]. This type of representation where values are atomic ones was called by Pawlak [Pawlak, 81] as a single valued information system. However it is also possible to consider so-called multivalued information that enables to be the attribute values not only atomic but also sets of values. In this case we introduce $\Pi(V_a)$ that denote the set of subsets of set V_a and $\Pi(V)=\bigcup_{a\in A}\Pi(V_a)$.

Vectors and touples belong to the most popular profile representation methods but in many recommendation systems other methods have been used, such as weighted n-grams, weighted semantic networks, weighted associated methods, classifier-based models, user item ratings and demographic features [Montaner et al. 03], as well as

tree structures [Sobecki 02], [Sobecki 03]. User ratings and demographic features are some specialization of already presented feature vector or the tuple representations, but the former methods are quite different from the ones already presented.

Weighted semantic networks are devoted to store meanings of represented profiles by some net of connected words or concepts, quite often with the labeled role of the link such as synonymy or superclass-subclass. In the recommendation systems ifWeb or SiteIF concepts and weights represent the degree of the user interest. The other type of representation - weighted associative networks as oppose to the semantic networks do not distinguish different types of links and weights represent only one type of association with some weight. The last type of the presented profile representation is classifier-based models that contain the following methods: neural networks, decision trees and Bayesian networks but their presentation is out of the scope of this paper.

Finally the tree representation is used when the profile could not be easily represented by a linear structure and independence of the features could not be assumed. Different attributes that describe user profile could have hierarchical dependencies and could be represented in an object-oriented manner or by a tree-like structure. In that structure attributes and their values are attached to the tree nodes and edges reflect dependencies among attributes and their values.

3.2 Profile Initialization

The initial profile may be empty, especially in case of content-based recommendations. In other approaches the initial profile is quite often created from the questionnaire that is filled in by the user. The questionnaire usually contains information on user data that contains different information, i.e. demographic data containing: record data (name, address, phone number, e-mail), geographic data (city, region, country and zip-code), user’s characteristics (gender, education, occupation), and some other customer qualifying data. The user data may also contain information on users’ knowledge, their skills, interests and preferences and also their plans and goals.

The usage data, the second element of the user model, is observed and recorded during the whole process of user’s interactions with web-based systems. The usage data may concern selective operations that express users’ interests, unfamiliarity or preferences, temporal viewing behavior, as well as ratings concerning the relevance of these elements. Then during the whole user-system interaction different events, such as opening a page, purchasing a product, sending feedback information to the system are stored. There are of course more sophisticated and general methods for gathering such data, as for example DoubleClick mechanisms. DoubleClick enables tracking the user web activities and serving personalized advertisements [Whalen 02] by using cookies entries with unique identifiers and placing web-bug image on every page that is to be tracked.

The initial profile may be also modified according to the whole user population behavior, which is the case of collaborative recommendation. However this brings some problems with finding similar users that could be solved by clustering methods [Kanungo et al. 02].

3.3 Distance Function Definition

The distance function between values of each attribute of the user profiles is defined as a function $\delta : V_a^u \times V_a^u \rightarrow R_+ \cup \{0\}$ for all $a \in B \subseteq A^u$. This function should be given by the system designer and fulfill all the distance function conditions but not especially all the metrics conditions. The distance function for each attribute may be different. The values of this function may be enumerated or given in any procedural form.

Despite utilization of distance function we can also apply similarity function $\sigma : V_a^u \times V_a^u \rightarrow R_+ \cup \{0\}$. There are many analogies between distance and similarity functions and it is possible to transform any distance function into similarity function and vice versa in one of the following way [Dąbrowski and Laus-Mączyńska 78]:

1. $\sigma(a,b)=1/\delta(a,b)$ dla $\delta(a,b)\neq 0$;
2. $\delta(a,b)=1/\sigma(a,b)$ dla $\sigma(a,b)\neq 0$;
3. $\sigma(a,b)=\max_{x,y\in U} [\delta^n(x,y)]- \delta^n(a,b)$ if exists finite $\max_{x,y\in U} [\delta(x,y)]$ and $n=1,2,...$;
4. $\delta(a,b)=\max_{x,y\in U} [\sigma^n(x,y)]- \sigma^n(a,b)$ if exists finite $\max_{x,y\in U} [\sigma(x,y)]$ and $n=1,2,...$;
5. $\sigma(a,b)=e^{-\delta(a,b)}$;
6. $\delta(a,b)=-\ln\delta(a,b)$ dla $\sigma(a,b)\in(0,1]$.

The final form of distance or similarity depends of the microstructure of the attribute values (i.e. atomic or subset value). Most of the most popular distance or similarity functions are defined for feature vector (binary) or weighted feature vector:

1. Euclidean distance: $\delta(a,b)=\sqrt{\sum_{i=1}^{n}(a_i-b_i)^2}$
2. Euclidean distance with weighted axis: $\delta(a,b)=\sqrt{\sum_{i=1}^{n}\omega_i(a_i-b_i)^2}$
3. Hamming distance : $\delta(a,b)=\sum_{i=1}^{n}|a_i-b_i|$
4. Average Hamming distance (Manhattan distance) [Cho 83]: $\delta(a,b)=\frac{1}{n}\sum_{i=1}^{n}|a_i-b_i|$
5. Canber distance: $\delta(a,b)=\sum_{i=1}^{n}\frac{|a_i-b_i|}{a_i+b_i}$
6. Cosinus similarity: $\beta(a,b)=\frac{\sum_{i=1}^{n}a_i * b_i}{\sqrt{\sum_{i=1}^{n}a_i^2 * \sum_{i=1}^{n}b_i^2}}$

The distance between elements of the user profiles could be defined in many different ways. First, the distance between tuples *i* and *j* could be defined as a simple sum of distances between values of each attribute:

$$\delta(p_i,p_j)=\sum_{a\in B}\delta^{at}(p_i(a),p_j(a)),$$

or we can consider the cosine distance or define the distance as a square root of sum of squares of distances (Euclidean distance):

$$\delta(p_i, p_j) = \sqrt{\sum_{a \in B} \left(\delta^{at}(p_i(a), p_j(a))\right)^2}$$

We can also indicate the importance of each attribute a by using some weight defined as a function $c: A \rightarrow [0,1]$ so the distance is defined as follows:

$$\delta(p_i, p_j) = \sum_{a \in B} [c(a) * \delta^{at}(p_i(a), p_j(a))]$$

The functions shown above are devoted for the user profile representation in form of a tuple, but in the case of the tree structures we must consider other distance functions [Sobecki 03].

3.4 User Clustering Based on the User Profile

Collaborative recommendation of web-based system user interface described in works [Nguyen and Sobecki 03] and [Sobecki and Weihberg 04] uses clustering of user profiles. Clustering problem could be defined as a partition of the given set of user profiles into subsets such that a specific criterion is optimized. The criterion is often defined as the average squared Euclidean distance between a profile and the corresponding cluster center. To minimize this criterion we can use k-means clustering that partitions the set of the profiles into k non-overlapping clusters that are identified by their centers. This problem is known to be NP-hard, but it is still attractive because of its simplicity and flexibility [Kanungo et al. 02]. It has however some disadvantages that reveals especially in case of a large datasets, these are: its low speed and lack of scalability; it is possible to obtain local minima instead of global ones.

In the interface recommendation implementation described in [Sobecki and Weihberg 04] Dattola clustering algorithm [Dattola 68] that is known from the field of Information Retrieval was used. This algorithm is not NP-hard but produces the sub-optimal solution to the clustering problem. In the Dattolla algorithm first the initial centroids must be selected, in our case they are selected by experts and denoted as the values of attributes from the set *N*. Then for each user profile the distance function [see Section 3.3] between the profile and each centroid is determined. The profile is joined to the group with the closest the centroid and also lower that assumed threshold, those above are assigned to the class of so-called isolated elements. Then for each group the centroides are recalculated and the process is repeated until no one profile changes its class assignment. Finally all profiles from the class of isolated elements are assigned to the group with the lowest distance function values or left as a separate group.

In the field of data mining however other algorithm is used to solve k-means problem. Its name is Lloyd's algorithm [Kanungo et al. 02] and its steps are following. First, select randomly *k* elements as the starting centers of the clusters (centroides). Second, assign each element of the set to a cluster according to the smallest distance to its centroid. Third, recompute the centroid of each cluster, for example the average of the cluster's elements. Fourth, repeat steps 2 and 3 until some convergence conditions have not been met (for example centroides do not change).

The attractiveness of this algorithm lies in its simplicity and its ability to terminate when using the above mentioned convergence condition and for configurations without equidistant elements to more than one centroid. There is, however, one important problem with k-means algorithm, namely the algorithm takes a long time to run. First, the step 2 that has to be performed in each iteration costs $O(kdN)$, where d is the dimension of each element and N is the number of elements. Second, algorithm usually needs many iterations to terminate. There are however quite many modification of this algorithm that run faster, for example bisecting k-means that begins with single cluster containing all the elements, then splits it in two clusters and replaces it by split clusters. Splitting a cluster consists of applying k-means algorithm some α times with k=2, keeping the split that average distance between all the elements and the centroid is the smallest.

4 Recommendation Approaches

We can distinguish three basic recommendation approaches: demographic, content-based and collaborative. The demographic approach is using stereotype reasoning [Kobsa et al. 01] and is based on the information stored in the user profile that contains different demographic features [Montaner et al. 03]. Stereotype reasoning is a classification problem aimed at generating initial predictions about the user using the demographic data [Kobsa et al. 01]. This type of recommendation is also used in the initial steps of the collaborative user interface recommendations [Nguyen and Sobecki 03] and [Sobecki and Weihberg 04].

The demographic data, such as for example zip codes of living places may be sufficient to draw quite detailed assumptions on the people's social status, interests and various purchasing behaviors. This method has its origin in the work of Jonathan Robbin, a marketing specialists from the U.S., who noticed over thirty years ago that the address zip code might serve as a very good indicator of quite many assumptions on people's characteristics [Quinn and Pawasarat 01]. However this method is now criticized web-based system users are often asked to enter their zip code, along with answers to more or less personal questions.

The demographic recommendations have however some disadvantages [Montaner et al. 03], [Nguyen and Sobecki 03]:

- for many users generalizations of the user's interests associated with some demographic attribute values may be too general;
- they do not provide any individual adaptation, also when the user interests tend to change over time;
- users are quite often reluctant to submit demographic information or lie in this matter.

Content-based filtering takes descriptions of the content of the previously evaluated items to learn the relationship between a single user and the description of the new items [Montaner et al. 03]. Content-based filtering is a method of recommendation applied in many interface agents. The interface agents developed at MIT [Fleming and Cohen 99] first observe their users and then apply some machine learning mechanisms to draw the recommendation. For each new situation, the agent computes the distances between the current state and each past state that is stored in

the memory. Together with these past states, corresponding user actions are stored. The interface agent recalls action which bears the largest resemblance to the current situation , or in other words, which has the smallest distance from it, and offers it as a recommendation. We can find quite many applications of interface agents. For example, Letizia, an autonomous interface agent for Web browsing [Lieberman 97], records URL's of visited pages and constructs the user profile out of them. Then, using simple keyword-frequency measure, adopted from the field of Information Retrieval, the agent searches the neighborhood of pages currently visited for potentially relevant pages. Another type of interface agent is Apt Decision that learns user's real estates rental preferences to suggest appropriate apartments [Shearin and Lieberman 01]. Apt Decision agent uses initial profile provided by the user as well as descriptions of apartments extracted from offers the user has analyzed so far.

The content-based approach enables personalized and effective recommendations for particular users, but has also some disadvantages:

- content-based approaches depends on so called objective description of the recommended items;
- it tends to overspecialize its recommendations;
- content-based approach is based only on the particular user relevance evaluations, but users usually are very reluctant to give them explicit, so usually other implicit, possibly less adequate, methods must be used.

The last of the distinguished types of recommendations is called collaborative filtering. It is able to deliver recommendations based on the relevance feedback from other similar users. The main advantages of collaborative filtering over the content-based architecture are following [Montaner et al. 03]:

- the community of users can deliver subjective data about items;
- collaborative filtering is able to offer novel items, even such that user have never seen before;
- collaborative recommendation utilizes item ratings of other users to find the best fitting one.

Collaborative recommended agents have also some disadvantages:

- when the number of other similar users is small then the prediction is rather poor;
- the quality of service for users of peculiar tests is also bad; this is rather difficult to get sufficient number of similar users to be able to make proper predictions;
- lack of transparency in the process of prediction and finally the user's personal dislike may be overcome by the number of other similar users opinions.

The disadvantages of each of the recommendation types could be overcome by applying the hybrid solution. In the works [Nguyen and Sobecki 03], [Sobecki and Weihberg 04] the concept and implementation of the collaborative user interface adaptation using consensus method was presented. The disadvantage of the insufficient number of the similar users at the early stages of the system operation was overcome by application of the demographic stereotype reasoning. However in this architecture the content-based recommendation is not implemented, the preferences of each individual user concerning interface settings that are selected manually are stored in the interface profile and used in every system session.

5 Hybrid Recommendation Architecture for Web-based System User Interfaces

In previous works [Nguyen and Sobecki 03], [Sobecki and Weihberg 04] the user interface recommendation was based on the mixture of the demographic and the collaborative recommendation. Basically the hybrid recommendation [Sobecki 04a] is a combination of demographic, collaborative and content based recommendation. However other types of recommendations that are based on: user emotions, user platform or context of use may be also considered. The chapter deals with each of the standard methods separately, gives some remarks on other types of recommendation as well as presents selection rules for the combined recommendation.

In the following sections the examples will be based on the implementation described in [Sobecki and Weihberg 04]. In the user profile several elements may be distinguished. We can distinguish the demographic attributes set D, the demographic attributes of the centroid classes of users set N, the set of recommended interface settings I, the set of the interface setting attributes made directly by the user J, the set of the actual interface setting attributes together with usability valuation values F, the set C of attributes associated with the content (for example visited pages, purchased or ordered items, retrieved elements) and finally, we can distinguish some attributes used for identification and authorization purposes T. So the set of attributes equals the sum of its elements: $A=D\cup J\cup I\cup C\cup T$.

5.1 Demographic Interface Profile Determination

The demographic profile determination is based on stereotype reasoning. The stereotypes are determined by an expert or a group of experts who determines the initial centroids of demographic attributes (set N) values which represent several initial classes of users.

The values of the centroid attributes should be selected in such a way that none of them had all the extreme (maximal or minimal) values and the distance between consecutive centroids was similar. For example in the implementation described in [Sobecki and Weihberg 04] the following values of the centroid demographic attributes *N={c_gender,c_age, c_education, c_number_of_inhabitants, c_type_of_information}* where used:

- male, less than 25 years old, primary education, from 500.000 to 1.000.000 inhabitants, technical information;
- male, over 50 years old, primary education, from 500.000 to 1.000.000 inhabitants, technical information;
- female, from 25 to 50 years old, secondary education, from 100.000 to 500.000 inhabitants, general information;
- female, over 50 years old, higher education, over 1.000.000 inhabitants, general information;

Each of these centroids had the corresponding interface profile assigned by the expert which is recommended to the user after registering to the system by delivering demographic information (entering values of attributes from the set N). In the implementation described in work [Sobecki and Weihberg 04] only single expert

opinion is allowed. We can assume however that more than one expert opinion is allowed and then determine consensus among all the opinions.

In case of the multiple expert opinions we can distinguish two situations. In the first one, all the experts share the same centroid attribute values (concerning demographic attributes) but give different opinions on interface settings. In the second situation, all the experts give opinions on centroides settings and corresponding user interface attributes values.

In both cases however we try to find consensus for each distinct centroid. Concerning the second case, the number of centroid may increase significantly. When they outnumber the desired limit we can group them, find new centroids and then find the consensus within these groups.

To define the conflict situation we should determine the some set of attributes:
N={*c_gender, c_age, c_education ,c_number_of_inhabitants,c_ type_of_information*}
I={*main_menu, option_information, option_colours, option_gallery, option_version, option_files, toolbar, background, music_track, music_loudness, sound_effects, effects_loudness, language, type_of_information*}
T={login, password, name, surname, expert, bookmark_assert_time}
The set of all attributes is defined as: $A = D \cup N \cup J \cup I \cup F \cup C \cup T$, however the precise determination of all the components is not necessary in this place.

$S^c = <A,X,P,Z>$, where *c* is demographic
$X = \{\Pi(V_a): a \in A\}$
$P \subseteq \Pi(V_{expert}) \times \Pi(V_{a1}) \times \Pi(V_{a2}) \times ... \times \Pi(V_{an})$, where $\{a_1, a_2, ..., a_n\} = N \cup I$
Z: does not contains any formulas

The conflict situation is defined as follows $cs^c = <P, Y \rightarrow B>$, where *P* is defined above and $Y=N$ and $B=I$.
Event(cs^c)= $\Pi^{\{expert\}}(P)$
Subject(cs^c)= $\Pi^N(P)$
Profile(e)=$\{r_{I \cup \{expert\}}: (r \in P) \wedge (e \prec r_A)\}$ for each $e \in$ Subject(cs^c).
$C(cs^c, e) \in TYPE(N \cup I)$
So finally the consensus for each subject is determined by finding the values of the interface settings for which $\sum_{r \in \mathrm{Pr}ofile(e)} \delta(r_I, C(cs^c, e)_I)$ is minimal.

5.2 Collaborative Interface Profile Determination by Means of Consensus Methods

The application of collaborative recommendation is possible when significant number of users have been registered, used the system, personalized the interface and delivered information concerning the interface usability. More precisely we must have the group of similar users *G* concerning values of demmographic attributes from the set *D*. The user groups are identified by the centroids, determined by the user clustering described above. are entered into the same consensus profile and then the consensus is calculated.

In the adaptive system using collaborative recommendation presented in work [Sobecki and Weihberg 04] the conflict situation will be defined as follows:

D={ *age, gender, education, number_of_inhabitants, type_of_information*}
J={*j_main_menu, j_option_information, j_option_colours, j_option_gallery, j_option_version, j_option_files, j_toolbar, j_background, j_music_track, j_music_loudness, j_sound_effects, j_effects_loudness, j_language, j_type_of_information, usability*}
T={login, password, name, surname, user_group, expert, bookmark_assert_time}
The set of all attributes is defined as: $A= D\cup N\cup J\cup I\cup F\cup C\cup T$, however the precise determination of all the components is not necessary in this place.

$S^c=<A,X,P,Z>$, where c is collaborative

$X=\{\Pi(V_a): a\in A\}$
$P\subseteq\Pi(V_{login})\times\Pi(V_{a1})\times\Pi(V_{a2})\times...\times\Pi(V_{an})$, where $\{a_1,a_2,...,a_n\}=\{user\_group\}\cup J$
Z: contains following logic formulas, for example
1. *if* $r\in P$ *then* $r_{usability}=\{q\}$ and $q>\varepsilon$ - usability is greater than assumed treshold ε
2. *if* $r\in P$ *then* r_{login} *is a key of relation r*
3. $r_{user\_group}=\{g\}$ where g equals user group identifier that is determined by means of clustering based on the values from the set D

The conflict situation is defined as follows $cs^c=<P, Y\rightarrow B>$, where P is defined above and $Y=\{user\_group\}$ and $B=J\backslash\{usability\}$.
Event(cs^c)= $\Pi^{\{login\}}(P)$
Subject(cs^c)= $\Pi^{\{user\_group\}}(P)$
Profile(e)=$\{r_{J\cup\{login\}}: (r\in P)\wedge(e\prec r_A)\}$ for each $e\in$ Subject(cs^c).
$C(cs^c,e)\in TYPE(\{user\_group\}\cup B)$

So finally the consensus for each subject is determined by finding the values of the interface settings for which $\sum_{r\in Profile(e)} r_{usability} * \delta(r_B, C(cs^c, e)_B)$ is minimal, we should notice that comparing to the expression from the *Theorem 1* the distance is multiplied by the value of the usability of each particular interface.

5.3 Content-based User Interface Recommendation

To deliver content-based recommendation for a particular user we must have sufficient usage data of that user. The second important condition is delivering appropriate inductive rules that will transform this data into the user interface settings.

The rules for efficient content-based recommendations strongly depend on the goals of the web based system. For example for web-based information retrieval systems we can consider the previous relevant items as a basis for recommendation of further retrievals. In this case many different methods can be used: fuzzy retrieval, Bayesian networks or other intelligent information retrieval method. The same methods we can use for other similar tasks such as news and e-mails filtering or spam detection. This kind of methods was especially applied in the field of the interface agents, for example in systems like Letizia [Lieberman 97] or Apt Decisions [Shearin and Lieberman 01].

For quite many systems however, the logic used for the retrieval systems that means the assumption that when a user perceive something as being relevant, he or she will also have the same opinion for the something similar, does not hold. As an example may serve the situation when users buy an article that they need only single item in specified period of time (e.g. car, TV set, DVD player, etc.). So when the user buys that article, it is no need to offer similar ones in the nearest future. In that cases we should rather recommend items that other users who bought the same item were also interested in, for example insurance when a user buys a car or films on DVD when a user buys a DVD player.

The logic of recommended systems may be even more complicated than this presented above. So, for each recommended item, no matter if an element of interface settings or a content item, we shall define precise relationship between the user profile (or also other users profiles) and this particular item.

In the content item recommendation we can also consider application of the design heuristic rules concerning the content and presentation of the information [Pearrow, 00]:

- meaning: the information has meaning when it concerns many people;
- time: the information is delivered on time;
- publicity: the information has a great publicity when it concerns a very well known person or organization;
- adjacency: the information is adjacent when the fact has a place near the place of the user residence;
- conflict: the information is interesting when it concerns the conflict among people;
- peculiarity: the information is interesting when it is quite unusual;
- timeliness: the information is interesting when it concerns current event.

That kind of general rules for content-based recommendation may be implemented for example as ruled based system, Bayesian or neural networks. However the usage data may lead to many different recommendations so there is a place for consensus determination.

It is not necessary that the content-based recommendation delivers the whole interface settings. It is sufficient when we got recommendations concerning some part of the interface settings. For example, when the usage data encounters that the user set the text font size of the browser into large then the corresponding content-based recommendation rules should recommend the same. The other data however, i.e. settings in some particular web-based systems, may suggest other font size settings. The bookmarks may deliver information on most interesting content topics for the user, but the same information may also be delivered by the systems such as DoubleClick.

We can now show the example of potential application of consensus methods applied in user interface adaptation. In this method the attribute *bookmark_assert_time* identifies moments when users enters new bookmarks. In the adaptive system using collaborative recommendation presented in work [Sobecki and Weihberg 04] the conflict situation will be defined as follows:

I={*main_menu, option_information, option_colours, option_gallery, option_version, option_files, toolbar, background, music_track, music_loudness, sound_effects, effects_loudness, language, type_of_information*}
C={bookmark}
T={login, password, name, surname, user_group, expert, bookmark_assert_time}

The set of all attributes is defined as: $A = D \cup N \cup J \cup I \cup F \cup C \cup T$, however the precise determination of all the components is not necessary in this place.

$S^c=<A,X,P,Z>$, where c is content-based

$X=\{\Pi(V_a): a\in A\}$
$P\subseteq\Pi(V_{login}) \times \Pi(V_{a1}) \times \Pi(V_{a2}) \times ... \times \Pi(V_{an})$, where $\{a_1,a_2,...,a_n\}=I\cup C$
Z: contains following logic formulas, for example
1. $\forall_{r\in P}$ $tf(\{auto,moto,engine\}, r_{bookmark})>\varepsilon$ *then* $r_{type\_of\_information}=technical\_detail$
2. $\forall_{r\in P}$ $tf(\{fashion,art\}, r_{bookmark})>\varepsilon$ *then* $r_{type\_of\_information}=general$

The rules mentioned above means that if on the pages from the bookmark list contain words such as auto, moto or engine with term frequency greater than assumed treshold ε then the interface settings of the type of information should be set to the technical detail. In contrary when term frequency of words such as fashion or art is greater than ε then the interface settings of the type of information should be set to the general.

The conflict situation is defined as follows $cs^c=<P, Y\to B>$, where P is defined above and $Y=\{login\}$ and $B=I$.
Event(cs^c)= $\Pi^{\{\text{bookmark\_assert\_}time\}}(P)$
Subject(cs^c)= $\Pi^{\{login\}}(P)$
Profile(e)=$\{r_I: (r\in P) \wedge (e \prec r_A)\}$ for each $e\in$ Subject(cs^c).
$C(cs^c,e)\in TYPE(\{login\}\cup I)$

So finally the consensus for each subject, in this case each user identified by login, is determined by finding the values of the interface settings for which $\sum_{r\in Profile(e)} \delta(r_B, C(cs^c,e)_B)$ is minimal.

5.4 Other Recommendation Methods

Beside above mentioned recommendations: demographic, collaborative and content-based, we should also mention other ones, such as: platform, situation or emotion based. This kind of recommendations may be dealt in two different ways. The first one is based on the expansion of the subject's attribute set with the attribute concerning platform, situation or emotions in standard collaborative or content consensus-based recommendation. The second methods treats these recommendations as a separate ones.

The consensus system for that kind of recommendation is similar to the collaborative one. However despite *user_group* attribute we should use other attributes such as *platform*, *situation* or *user_mood*. As typical platform values may of course serve: desktop, information kiosk, notebook, embedded system (i.e. in car), PDA or advanced mobile phone. We can also distinguish the following typical situations: at work, at home, in a car, meeting, outside, etc.

In the adaptive system using collaborative recommendation presented in work [Sobecki and Weihberg 04] the conflict situation will be defined as follows:
D={ *age, gender, education, number_of_inhabitants, type_of_information* }
J={*j_main_menu, j_option_information, j_option_colours, j_option_gallery, j_option_version, j_option_files, j_toolbar, j_background, j_music_track, j_music_loudness, j_sound_effects, j_effects_loudness, j_language, j_type_of_information, usability*}
T={login,password,name,surname,expert,user_group,bookmark_assert_time, platform}

The set of all attributes is defined as: $A= D\cup N\cup J\cup I\cup F\cup C\cup T$, however the precise determination of all the components is not necessary in this place.

$S^c=<A,X,P,Z>$, where *c* is collaborative

$X=\{\Pi(V_a): a\in A\}$
$P\subseteq\Pi(V_{login})\times\Pi(V_{a1})\times\Pi(V_{a2})\times...\times\Pi(V_{an})$, where $\{a_1,a_2,...,a_n\}=\{platform\}\cup J$
Z: contains following logic formulas, for example
1. $\forall_{r\in P}\ \ r_{usability}=\{q\}$ *and* $q>\varepsilon$ - usability is greater than assumed treshold ε
2. $\forall_{r\in P}\ \ r_{login}$ *is a key of relation r*

The conflict situation is defined as follows $cs^c =<P, Y\rightarrow B>$, where *P* is defined above and $Y=\{user\_group\}$ and $B=J\backslash\{usability\}$.
Event(cs^c)= $\Pi^{\{login\}}(P)$
Subject(cs^c)= $\Pi^{\{platfom\}}(P)$
Profile(e)=$\{r_{J\cup\{login\}}: (r\in P) \wedge (e\prec r_A)\}$ for each $e\in$ Subject(cs^c).
$C(cs^c,e)\in TYPE(\{user\_group\}\cup B)$

So finally the consensus for each subject is determined by finding the values of the interface settings for which $\sum_{r\in \Pr ofile(e)} r_{platform} * \delta(r_B, C(cs^c,e)_B)$ is minimal, we should notice that comparing to the expression from the *Theorem* 1 the distance is multiplied by the value of the usability of each particular interface.

5.5 Hybrid User Interface Recommendation

The result of consensus determination in all the categories: demographic, collaborative, content-based, platform or situation based is a recommendation of the user interface for the particular user. Obviously there could be significant differences

in the recommendation of each user interface attributes. So the question arises, which type of recommendation should be preferred?

In the cases when only one type of recommendation is available and the others do not deliver any (*null* value of the recommendation) then it is obvious that we should use the one that is available. However usually three or more types of recommendation may deliver different settings. In such cases specific selection rules should be applied.

For some attributes concerning information content, i.e. recommended items such as holiday destinations or goods to sell that is presented to the user we should recommend a mixture of collaborative, content-based and situation recommendation. For example in restaurant recommendation, situation may deliver information about the place where the user is at the moment, and then recommend only those restaurants that are nearby.

Generally when the attribute concerns the interface settings that are usually to many different parameters such as text size, menu position (left or right) or type of buttons (text or graphic) we should rather rely on the content-based recommendation. However, more specific interface settings should be rather recommended using collaborative methods because other users that are similar to the particular one have tried already such user interface with same settings and assessed it as a usable one. Demographic recommendation may be chosen for some attributes by the experts, for example the user age may strongly determine the content, language and terminology of the texts presented to the user. Other types of recommendation such as platform may have dramatical influence on the potential different interface settings. In these cases we may try to find such interface settings that are valid for specific platform but in the same time they have the closest distance to all the recommendations.

6 Summary

In this paper different consensus-based recommendation methods, such as demographic, collaborative, content, platform have been presented. The consensus model have been presented in general and then applied in these different recommendation methods. Recent works on several implementations of consensus based collaborative user interface recommendations [Nguyen and Sobecki 03] have proven that the method may be applied in many different web-based information systems, mainly in the field of the product or service promotion. The system concerning a particular car model was analyzed in more details in work [Sobecki and Weihberg 04], the results prove that demographic and collaborative user interface recommendation leads to its improvements. The other systems also proved to be effective in the sense of usability.

The hybrid approach was up till now only partially implemented but comparing to the pure collaborative approach, the experiments requires longer time of users work with the system (also in several separate sessions) to gather sufficient data for content-based recommendation.

References

[Arafa and Mamdani 00] Arafa, Y., Mamdani, A.: “Virtual Personal Service Assistants: Towards Real-time Characters with Artificial Hearts”; Proc. Intelligent User Interfaces New Orleans LA USA (2000), 9-12.

[Barthelemy 88] Barthelemy, J.P.: “Thresholded consensus for n-trees”; J of Classification 5 (1988), 229-236.

[Basili et al. 03] Basili, R., Pazienza, M.T., Zanzotto F.M.: “Exploiting the feature vector model for learning linguistic representations of relational concepts”, Proc. Workshop on Adaptive Text Extraction and Mining ATEM (2003), online Dec 2004/ http://www.dcs.shef.ac.uk/~fabio/ATEM03/basili-ecml03-atem.pdf.

[Daniłowicz et al. 02] Daniłowicz, C., Nguyen, N.T., Jankowski, Ł.: “Methods for the Choice of Representation of Agent Knowledge States in Multiagent Systems”; WUT Press: Wrocław, (2002). (in Polish)

[Dąbrowski and Laus-Mączyńska 78] Dąbrowski, M., Laus-Mączyńska, K.:”Methods of retrieval and classification”. WNT, Warszawa 1978. (in Polish)

[Dattola 68] Dattola, R.T.: “A fast algorithm for automatic classification”; Report ISR-14 National Science Foundation, Section V, Cornell University, Dep. of Computer Science (1968).

[Day 88] Day, W.H.E.: “Consensus methods as tools for data analysis”; Bock, H.H. (eds), Classiication and related Methods of Data Analysis. Proceedings of IFCS'87, North-Holland, (1988) 317-324.

[Fleming and Cohen 99] Fleming, M., Cohen, R.: “User modelling in the design of interactive interface agents”; Proc of the Seventh International Conference on User Modeling, (1999) 67-76.

[Kanungo et al. 02] Kanungo, T., Mount, D.M., Netanyahu, N.S., Piatko, C., Silverman, R., Wu, A.Y.: “An Efficient k-means clustering algorithm: analysis and implementation”; IEEE Tran. On Pattern Analysis And Machine Intelligence 24(7), (2002) 881-892

[Kobsa et al. 01] Kobsa, A., Koenemann, J., Pohl, W.: “Personalized Hypermedia Presentation Techniques for Improving Online Customer Relationships”. Knowledge Eng. Rev. 16(2), (2001) 111-155.

[Lieberman 97] Lieberman, H.: “Autonomous Interface Agents“; Proc. CHI 97, ACM (1997) 67-74.

[Montaner et al. 03] Montaner, M., Lopez, B., de la Rosa, J.P.: “A Taxonomy of Recommender Agents on the Internet”; Artificial Intelligence Review 19, (2003) 285-330.

[Nguyen 00] Nguyen, N.T.: “Using consensus methods for solving conflicts of data in distributed systems”; LNCS 1963, (2000) 409-417.

[Nguyen 01] Nguyen, N.T.: “Conflict Profiles' Susceptibility to Consensus in Consensus Systems”; Bulletin of International Rough Sets Society 5(1/2), (2001) 217-224.

[Nguyen, 02] N.T. Nguyen, Consensus System for Solving Conflicts in Distributed Systems. Information Sciences – An International Journal Vol. 147, 2002, 91-122

[Nguyen and Sobecki 03] Nguyen, N.T., Sobecki, J.: “Using Consensus Methods to Construct Adaptive Interfaces in Multimodal Web-based Systems”; Universal Access in Inf. Society 2(4), (2003) 342-358.

[Pearrow 00] Pearrow, M.: "Web Site Usability Handbook"; Charles River Media (2000).

[Quinn and Pawasarat 01] Quinn, L.M., Pawasarat, J.: "Confronting Anti-Urban Marketing Stereotypes: A Milwaukee Economic Development Challenge June (2001)". online Dec 2002/ http://www.uwm.edu/Dept/ETI/purchasing/markets.htm.

[Shearin and Lieberman 01] Shearin, S., Lieberman, H.: "Intelligent Profiling by Example"; Proc of the Conf on Intelligent User Interfaces, ACM Press, (2001) online Dec. 2004/ http://web.media.mit.edu/%7Esibyl/projects/apt/final-shearin-iui.pdf.

[Sobecki 02] Sobecki, J.: "Interface model in adaptive web-based system"; Cellary, W. and Iyengar, A. (ed.): Internet Technologies, Applications and Societal Impact. Kluver Academic Publishers, Boston, (2002) 93-104.

[Sobecki 03] Sobecki J, XML-based Interface Model for Socially Adaptive Web-Based Systems User Interfaces. Lecture Notes in Computer Science 2660, 2003, 592-598

[Sobecki and Weihberg 04] Sobecki, J., Weihberg, M.: "Consensus-based Adaptive User Interface Implementation in the Product Promotion"; Keates S. (et al.): "Design for a more inclusive world", Springer-Verlag (London), (2004) 111-121.

[Sobecki 04a] Sobecki J.: "Hybrid Adaptation of Web-Based Systems User Interfaces". Proc. of ICCS 2004, Krakow, Poland, Lecture Notes in Computer Science 3038, (2004) 505-512.

[Social 04] Social Choice Theory, (2004). online Oct. 2004 http://mathworld.wolfram.com/ SocialChoiceTheory.html

[Whalen 02] Whalen, D.: "The Unofficial Cookie FAQ, Version 2.54 Contributed to Cookie Central by David Whalen". (2002). online Sep. 2002 http://www.cookiecentral.com/faq/#2.7.

[Wooldridge and Jennings 95] Wooldridge, M., Jennings, N.R.: "Intelligent agents: theory & practice"; Knowledge Engineering Review 100(2), (1995) 115-152.
