# Supplementary material for: In-text citation’s frequencies-based recommendations of relevant research papers
Source: PeerJ Comput Sci. 2021 Jun 4;7:e524. doi: 10.7717/peerj-cs.524 (PMC8189020; doi:10.7717/peerj-cs.524)
Supplement: Supplemental Information 2 — These articles are available under a CC BY-ND 4.0 license. [file peerj-cs-07-524-s002.zip › Dataset/Papers Dataset.zip!/Papers Dataset/jucs_58.pdf]

Journal of Universal Computer Science, vol. 11, no. 6 (2005), 916-925
submitted: 4/10/04, accepted: 26/6/05, appeared: 28/6/05 *© J.UCS*

On Theoretical Upper Bounds for Routing Estimation

Fei He
(Dept. CS&T and School of Software, Tsinghua University, China
hef02@mails.tsinghua.edu.cn)

Lerong Cheng, Guowu Yang, Xiaoyu Song
(Dept. ECE, Portland State University, USA
chenglerong@yahoo.com, {whung, song}@ece.pdx.edu)

Ming Gu, Jiaguang Sun
(School of Software, Tsinghua University, China
{guming, sunjg}@tsinghua.edu.cn)

Abstract: Routing space estimation plays a crucial role in design automation of digital systems. We investigate the problem of estimating upper bounds for global routing of two-terminal nets in two-dimensional arrays. We show the soundness of the bounds for both wiring space and total wire-length estimation.
Key Words: global routing, algorithms, CAD, integrated circuits.
Category: B.7.2, F.2.2

1 Introduction

With deep-submicron technology, the interconnection delay becomes a dominant factor in integrated circuit designs. An efficient physical design entails accurate estimations of the individual modules for area planning, optimal placement, and routability of interconnections. Estimation of interconnects before layout becomes a crucial issue for a hierarchical design process.

In the hierarchical design process, the location of each module is determined during placement stage. After placement, routing process is performed for interconnects. Routing consists of global and local routing [Sherwani 95]. During global routing, an approximate route for each net is determined while in local routing an exact route is fixed for each net. Prior to the routing phase, placement is a difficult task. An ineffective placement may cause a unsolvable routing problem at later stage, thus reducing the routability. Therefore, an early estimation process is crucial to the success of placement.

Global routing of two-terminal nets is known to be NP-complete [Karp 87, Sherwani 95]. There have been many heuristic algorithms for global routing [Burstein 83, Cho 98, Kuh 86, Li 84, Luk 87, Sarrafzadeh 90, Veccchi 83].

Routing is to interconnect individual modules. A netlist provide the detail information for interconnection, where every net in the list specifies which pin in

which module should be connected together. Nets must be routed in area that is not occupied by any module, namely routing region.

In this paper, we present a quick prediction approach. Wire space estimation can provide a deeper insight for the placement stage [Cho 00, Gamal 81, Song 00]. We present theoretical upper bounds for routing estimation on both wire space and length.

Our paper is organized as follows. In Section 2, we give the formulation of the problem. In Section 3, we present a new upper-bound density. In Section 4, the estimation of the total wire length is discussed. Section 5 concludes the paper.

2 Preliminaries

As defined in [Cho 00], consider the global routing for a set of n two-terminal nets in a two-dimensional array. A plane consists of a 2-D $m \times m$ grid with 1×1 being the basic cell-grid size. A path was assumed to go from cell to cell horizontally or vertically. Let $cell(i, j)$ be a cell at the i-th row and the j-th column of the routing region. Let $d_h(i, j)$ denote the number of nets crossing the border of $cell(i, j)$ and $cell(i, j + 1)$. Similarly, let $d_v(i, j)$ denote the number of nets crossing the border of $cell(i, j)$ and $cell(i+1, j)$. The global density d_R is the maximum value of all $d_h(i, j)$ and $d_v(i, j)$, i.e. $d_R = \max_{i,j}\{d_h(i, j), d_v(i, j)\}$.Our purpose is to minimize the global density.

Our estimation approach is based on a top-down hierarchical approach, which partitions a routing region into four square sub-regions recursively [Burstein 83]. For the convenience of notation, we refer to the map to be quad-partitioned as quadrisection map. Let $QM(0)$ denote the original whole routing area. Each quadrisection map $QM(i)$ is partitioned into four quadrants $Q^i_k, k = 1, 2, 3, 4$ (labeled counterclockwise from upper-right corner). A common boundary of two adjacent quadrants is said to be a cut line. There are two vertical and two horizontal cut lines in $QM(i)$, denoted by $C^i_k, k = 1, 2, 3, 4$ (labeled counterclockwise from right horizontal cut line). The length of every cut line in $QM(i)$ is denoted by $L^i = m/2^{i+1}, 0 \leq i \leq log_2 m - 1$ (assume m is a power of 2).

In what follows, we restrict our discussion to two-terminal nets. Two-terminal nets are nets only connecting two pins of modules. It follows that the terminals of any pair of those nets are disjunct, because otherwise the pair of nets would be counted as one net with three terminals. The model can be extended to handle the multi-terminal case by using rectilinear Steiner tree or minimum spanning tree. A level-i net is a net in $QM(i)$ whose two terminals are located in the different quadrants of $QM(i)$. The terminals corresponding to the level-i nets are known as level-i terminals. Assume there are $t(i)$ level-i terminals in $QM(i)$, then the number of level-i nets is obviously $t(i)/2$. These $t(i)$ terminals have been

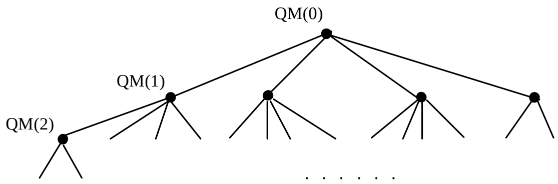

Figure 1: A quad-tree

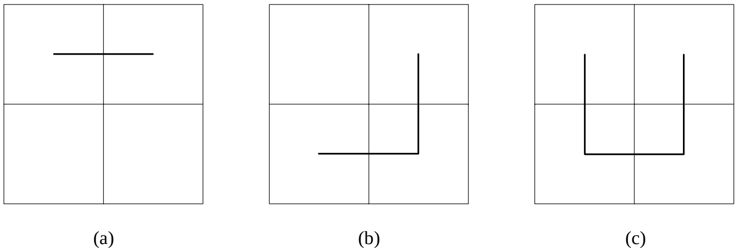

Figure 2: Three types of connections: (a) straight connection; (b) one-bend connection; (c) detour connection

distributed into four quadrants of $QM(i)$. Let $t(i,k)$ be the number of level-i terminals distributed to Q^i_k. We have: $t(i) = \sum_{k=1}^{4} t(i,k)$.

The process of quad-partitioning can be denoted by a quad-tree such that its root is $QM(0)$ and all of its level-i nodes are $QM(i)$, as shown in Fig. 1. It should be noted that there are 4^i $QM(i)$ in the level-i of a quad-tree. Considering the whole routing region as an $m \times m$ grid, the corresponding quad-tree has at most $log_2 m$ levels.

As in [Cho 00], the set of level-i nets in $QM(i)$ can be sorted as two types: type-1 (adjacent combination) and type-2 (diagonal combination). The type-1 nets can be routed using detour connection or straight connection, while the type-2 nets can be routed only using one-bend connection, as shown in Fig. 2. Furthermore, the set of type-1 nets can be sorted as: F^i_{12}, F^i_{23}, F^i_{34} and F^i_{41}; the set of type-2 nets can be sorted as F^i_{13} and F^i_{24}, where F^i_{pq} $(p,q = 1,2,3,4)$ denotes the nets which has one terminal in Q^i_p and the other terminal in Q^i_q. Let f^i_{pq} be the number of nets in F^i_{pq}.

Theorem 1. *[Cho 00] Assuming that nets are distributed evenly over cut lines, the tightly estimated upper bound on the number of tracks required on every cut line of $QM(i)$ is:*

$$\lceil (f^i_{13} + f^i_{24} + f^i_{max1} + f^i_{max2})/2 \rceil,$$

where $f^i_{max1} = \max\{f^i_{12}, f^i_{23}, f^i_{34}, f^i_{41}\}$, *and* $f^i_{max2} = \max\{\{f^i_{12}, f^i_{23}, f^i_{34}, f^i_{41}\} -$

$\{f^i_{max1}\}\}$.

Although some discussion on the proof scheme was given in [Cho 00], we give a rigorous and detailed proof as follows.

Proof. Let F^i_{max1} be the subset of type-1 nets which has f^i_{max1} nets, and C^i_{max1} be the cut lines corresponding to F^i_{max1}. Similarly we define F^i_{max2} and C^i_{max2}. Then consider the following routing strategy.

1) All nets in $F^i_{12} \cup F^i_{23} \cup F^i_{34} \cup F^i_{41} - F^i_{max1}$ are connected by adjacent connections; f^i_{max2} nets in F^i_{max1} are connected by adjacent connections. Among $f^i_{max1} - f^i_{max2}$ remaining nets in F^i_{max1}, half of which are assigned also by adjacent connections, half of which are assigned by detour connection; then there are at most $\lceil (f^i_{max1} + f^i_{max2})/2 \rceil$ tracks required on C^i_{max1} and C^i_{max2} to route these type-1 nets.

2) For type-2 nets, they are assigned evenly, such that there are at most $\lceil (f^i_{13} + f^i_{24})/2 \rceil$ tracks required on C^i_{max1} and C^i_{max2} to route these type-2 nets. Totally, there are at most $\lceil (f^i_{13}+f^i_{24}+f^i_{max1}+f^i_{max2})/2 \rceil$ tracks required on C^i_{max1} and C^i_{max2}. Obviously, the numbers on other cut lines cannot be greater than the number on C^i_{max1} and C^i_{max2}. With the assumption that the nets are distributed evenly, which is stronger than above routing strategy, the number of tracks required on every cut line must not be greater than the number produced by the above routing strategy. That is to say with this assumption, the upper bound for the number of tracks required on every cut line is $\lceil (f^i_{13}+f^i_{24}+f^i_{max1}+f^i_{max2})/2 \rceil$.

3 An Existential Density Upper Bound

Let d^i_k be the density of nets on cut line C^i_k, i.e. the number of nets crossing C^i_k. The global density (d_R) can be estimated as the maximal value of all d^i_k on all cut lines. Obviously, the nets contributing to the densities in $QM(i)$ (i.e. the net densities on cut lines in $QM(i)$) include not only the level-i nets, but also some higher level nets. Here the higher level nets denote the nets whose two terminals are distributed in different quadrants of higher-level nodes in a quad-tree.

Let $sum(i)$ be the sum of the number of nets which do affect the densities in $QM(i)$. From Theorem 1, we can conclude that the number of tracks required on every cut line of $QM(i)$ is at most $\lceil sum(i)/2 \rceil$. The *level density* which only considers the effect of the current level nets is defined as [Cho 00]: $d_0 = \max\{\lceil U^i/(2 \cdot L^i) \rceil \mid 0 \le i \le log_2 m - 1\}$, where $U^i = \max\{t(i,k) \mid 1 \le k \le 4^{i+1}\}$. Consider the level $r-2$ nets that pass $QM(r-1)$ as candidate nets, and define σ to be the maximal value of the propagation ratio of the candidate nets to $QM(r)$ $(2 \le r \le log_2 m-1)$.

Theorem 2. *There are at most* $2\left(1 + \sum_{l=0}^{i} 2^l \sigma^l\right) d_0$ *tracks required at each channel on the cut line in* $QM(i)$.

Proof. When i=0, the number of level-0 terminals in $QM(0)$ is $t(0) = \sum_{k=1}^{4} t(0,k) \leq 4U^0$, and the number of level-0 nets in $QM(0)$ is $t(0)/2 \leq 2U^0 \leq 2 \cdot 2L^0 d_0 = 4L^0 d_0$ (by $d_0 \geq U^0/(2L^0)$). From Theorem 1, we can conclude the maximal number of tracks required on every cut line of $QM(0)$ is no more than half of the total number of level-0 nets, so the density in $QM(0)$ is no more than $4L^0 d_0/(2L^0) = 2d_0$.

When i=1, there are two types of nets that contribute to $QM(1)$, one is the level-1 nets in $QM(1)$, and the other is the level-0 nets that pass $QM(1)$. From the analysis of i=0, the number of level-1 nets in $QM(1)$ is at most $4L^1 d_0$. Considering $QM(1)$ being a quadrant of $QM(0)$, there are two borders between $QM(1)$ and other quadrants of $QM(0)$, each of which can have at most $L^0 d_0$ tracks to connect the level-0 nets (by the definition of d_0). So the maximal number of tracks on the border of $QM(1)$ that can be used to connect level-0 nets is $2L^0 d_0$. In other words, there totally can have $2L^0 d_0$ level-0 nets passing $QM(1)$. Thus, the maximal density in $QM(1)$ is $(4L^1 d_0 + 2L^0 d_0)/(2L^1) = 4d_0$.

When i=2, there are totally three types of nets which contribute to $QM(2)$, type 1 is the level-2 nets in $QM(2)$, type 2 is the level-1 nets that pass $QM(2)$, type 3 is the level-0 nets that pass $QM(2)$. From the analysis of i=0, the maximal number of level-2 nets in $QM(2)$ is $4L^2 d_0$; while from the analysis of i=1, the maximal number of level-1 nets contributing to $QM(2)$ is $2L^1 d_0$. It is a bit harder to decide the propagation of type 3 nets to $QM(2)$. We consider the level-0 nets that pass $QM(1)$ as candidate nets. According to the discussion when i=1, we know the number of the candidate nets is $2L^0 d_0$. The candidate nets will affect $QM(2)$ in two cases: one of the terminals of the net is in $QM(2)$; no terminal is in $QM(2)$ but the wire will cross $QM(i+2)$. For the set of candidate nets, assume the percentages of candidate nets in cases 1 and 2 are r_1 and r_2, respectively. Note that all the candidate nets in case 1 will be propagated to $QM(2)$, i.e. the propagation ratio of the candidate nets in case 1 is 1. Assume the propagation ratio of the candidate nets in case 2 is x, then the propagation ratio of the total candidate nets is $r_1 + r_2 \cdot x$, where the parameters r_1, r_2 and x depend on the global routing. We can deduce the propagation ratio is between x and 1, by $1 \geq r_1 + r_2 \cdot x \geq r_1 \geq x + r_2 \cdot x \geq (r_1 + r_2) \cdot x \geq x$. In this paper, we assume the propagation ratio of any set of candidate nets is no more than σ, where $x \leq \sigma \leq 1$. Then the maximal density in $QM(2)$ is $(4L^2 d_0 + 2L^1 d_0 + \sigma \cdot 2L^0 d_0)/(2L^2) = 4(\sigma + 1)d_0$.

Recursively when i=r, there would be r+1 types of nets that do affect the densities in $QM(r)$, these nets are level-0 to level-r nets respectively. Because the propagation ratio of level-l candidate nets to $QM(l+2)$ is no more than σ, then the propagation ratio of it to $QM(l+3)$ is no more than σ^2, ..., and recursively the propagation ratio of it to $QM(r)$ is no more then σ^{r-l-1}. Then

we can conclude that the maximal density in $QM(r)$ is:

$$
\begin{aligned}
&(4L^r d_0 + 2L^{r-1} d_0 + \sigma \cdot 2L^{r-2} d_0 + \sigma^2 \cdot 2L^{r-3} d_0 + \ldots + \sigma^{r-1} \cdot 2L^0 d_0)/(2L^r) \\
&= 2\left(1 + \sum_{l=0}^{i} 2^l \sigma^l\right) d_0 \\
&= \begin{cases} 2\left(1 + \dfrac{1 - 2^i \sigma^i}{1 - 2\sigma}\right) d_0 & \text{if } \sigma \neq 0.5; \\ 2(i+1) d_0 & \text{if } \sigma = 0.5. \end{cases}
\end{aligned}
$$

Thus the theorem holds.

In [Cho 00], the propagation ratio of any set of candidate nets is implicitly limited to be no more than 0.5 (this conclusion can be easily arrived following the similar analysis as the proof to Theorem 2), and thus the number of tracks required on any channel is at most $2(i+1)d_0$. It is, in fact, a special form of our theorem with $\sigma = 0.5$. Our theorem can be viewed as the extended form of the *Invariant* in [Cho 00].

The corresponding conclusion in [Cho 00] can cover over most of but not all routing cases. As shown in Fig. 3, the routing area is $2^{16} \times 2^{16}$ (m=2^{16}). There are totally 2^{16} 2-terminal nets. These nets are all level-0 nets, because for each of which its two terminals are located in different quadrants of $QM(0)$. For each net, one terminal is in *Area A* and the other terminal is in *Area B*, where *Area A* and *Area B* are $2^8 \times 2^8$ square regions in the upper left quadrant and the lower right quadrant, respectively. There may be other terminals in area besides A and B, but they are not level-0 terminals and are not drawn in Fig. 3. According to our proposed model, *Area A* and *Area B* are corresponded to a level-8 node in the quad-tree, respectively. Especially, we denote *Area A* as $QM(8)$. The nodes on the path from root to $QM(8)$ (not including root and $QM(8)$) in the quad-tree are denoted as $QM(1)$, $QM(2)$, ..., QM(7) orderly. Note that all the terminals in *Area A* are not only in $QM(0)$, but also in $QM(1)$, $QM(2)$, ..., QM(7). Thus, the level-0 nets corresponding to these terminals would all be propagated to $QM(1)$, $QM(2)$, ..., QM(7). If we consider the level-0 nets which pass $QM(1)$ as candidate nets, then this example is just the extreme case with r_1=1, r_2=0 and therefore $\sigma = 1$. Obviously, the conclusion of [Cho 00] cannot hold for this case.

From Theorem 2, we can easily estimate the global density as:

Theorem 3. *In a 2-D $m \times m$ grid, the worst-case upper bound for the global density is:*

$$d_R \leq \max\left\{2\left(1 + \sum_{l=0}^{i} 2^l \sigma^l\right) d_0 \middle| 0 \leq i \leq \log_2 m - 1\right\}$$

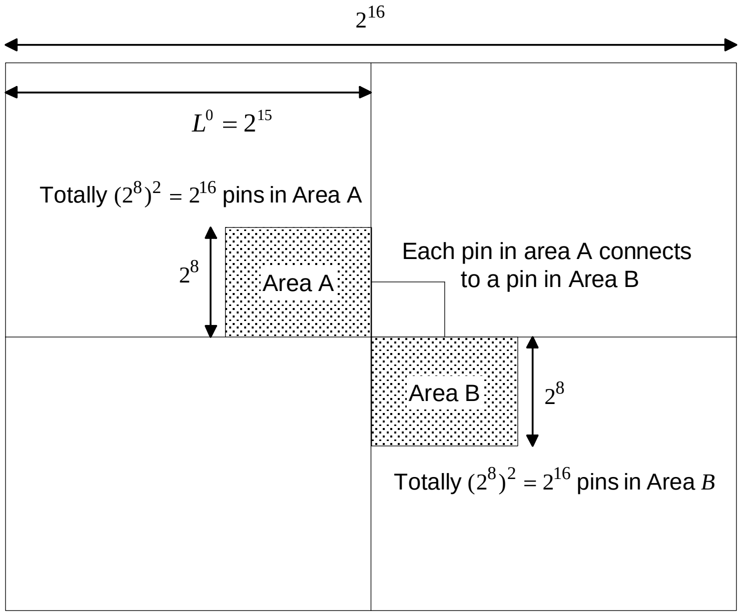

Figure 3: An example of the density upper bound

$$= \begin{cases} 2d_0 & 0 \leq \sigma < 0.5 \\ 2\log_2 m \cdot d_0 & \sigma = 0.5 \\ 2\left(1 + \dfrac{1-(2\sigma)^{\log_2 m - 1}}{1-2\sigma}\right) \cdot d_0 & 0.5 < \sigma \leq 1 \end{cases}$$

Considering the example shown in Fig. 3, if we let $\sigma = 1$, then the estimated worst-case upper bound density is $d_R \leq 2^{16}$.

Because the grid size is $m \times m$, with the upper bound density, the total wire space can be further estimated as no more than ${d_R}^2$.

4 Total Wirelength Estimation

Consider the connections for the set of level-i nets in $QM(i)$. As shown in Fig. 2, the straight connection that crosses one cut line is L^i long, the one-bend connection that crosses two cut lines is $2L^i$ long, and the detour connection that crosses three cut lines is $3L^i$ long. Assume that there are at most f_1^i straight connections, f_2^i one-bend connections and f_3^i detour connections in $QM(i)$, respectively, then the total wire length required to route the level-i nets in $QM(i)$ is $L^i(3f_3^i + 2f_2^i + f_1^i)$. There are totally 4^i level-i nodes in a quad-tree, for $i = 0, \ldots, log_2 m - 1$. Let F_1^i, F_2^i, and F_3^i be the total numbers of straight connections, one-bend connections and detour connections in all 4^i level-i nodes

respectively. Then the total wire length required to route all level-i nets in all level-i nodes, denoted as ω^i, is:

$$\omega^i = L^i \left(3F_3^i + 2F_2^i + F_1^i\right)$$

Note that the total number of the level-i nets in $QM(i)$ is no more than $4L^i d_0$ (refer to Section 3), so $f_3^i + f_2^i + f_1^i \leq 4L^i d_0$, and further: $F_3^i + F_2^i + F_1^i \leq 4^i \times (f_3^i + f_2^i + f_1^i) \leq 4^i \times 4L^i d_0 = 4^{i+1} L^i d_0$.

For the union set of level-i nets distributed in all 4^i level-i nodes, let α^i, β^i be the ratio of type-2 nets (diagonal combination) and type-1 nets (adjacent combination) respectively. Then we have $F_3^i + F_1^i = \beta^i \cdot (F_3^i + F_2^i + F_1^i) \leq \beta^i 4^{i+1} L^i d_0$ and $F_2^i = \alpha^i \cdot (F_3^i + F_2^i + F_1^i) \leq \alpha^i 4^{i+1} L^i d_0$, for $i = 0, \ldots, log_2 m - 1$. For the union set of nets in all levels, let α and β be the ratio of type-2 and type-1 nets, respectively. Obviously they are also the mean values of $\{\alpha^i\}_i$ and $\{\beta^i\}_i$ respectively. Note that the parameters α and β are independent of the propagation ratio. The values for α and β can be easily estimated with the locations of terminals of all nets.

During the global routing, the number of detour connections should be reduced as much as possible to minimize the global density, such that F_3^i is much less than F_1^i and F_2^i. Then ω^i can be estimated as:

$$\omega^i \approx L^i \left((F_3^i + F_1^i) + 2F_2^i\right) \leq (2\alpha^i + \beta^i) 4^{i+1} L^{2i} d_0 = (2\alpha^i + \beta^i) m^2 d_0$$

As a result, the total wire length in all levels of top-down hierarchy is:

$$\Omega = \sum_{i=0}^{\log_2 m - 1} \omega^i \leq \sum_{i=0}^{\log_2 m - 1} (2\alpha^i + \beta^i) m^2 d_0 = (2\alpha + \beta) \log_2 m \cdot m^2 d_0$$

If F_3^i cannot be ignored, notice that f_3^i would get its maximal value only in the case that all level-i nets are type-1 nets and all level-i terminals are distributed in the same two quadrants. In such a case, the half of the nets should be connected by adjacent connections, while the other half of nets should be connected by detour connections. Consider that the number of level-i nets is $f_3^i + f_2^i + f_1^i$, and then the number of detour connections in this case is $(f_3^i + f_2^i + f_1^i)/2$. So the maximal value of f_3^i is $(f_3^i + f_2^i + f_1^i)/2$, and we have:

$$(\omega^i)' = L^i (F_1^i + F_3^i + 2F_2^i + 2F_3^i) \leq L^i (2F_1^i + 2F_3^i + 3F_2^i) = (3\alpha^i + 2\beta^i) m^2 d_0$$

Hence, the total wire length in all levels of top-down hierarchy is:

$$\Omega' = \sum_{i=0}^{\log_2 m - 1} (\omega^i)' \leq \sum_{i=0}^{\log_2 m - 1} (3\alpha^i + 2\beta^i) m^2 d_0 = (3\alpha + 2\beta) \log_2 m \cdot m^2 d_0$$

We summarize the above discussion as follows (similar results can be found in [Cho 00], but they ignored there are 4^i $QM(i)$ in the level-i of a quad-tree):

Theorem 4. *The total wirelength required to achieve the upper bound obtained in Theorem 3 is* $(2\alpha + \beta)(log_2 m) \cdot m^2 d_0$*, where* α *is the proportion of diagonal nets,* β *is the proportion of adjacent nets, and* d_0 *is the estimated level density.*

5 Conclusion

We presented the worst case upper bounds for routing estimation. We proved that the upper bound density is $max \left\{ 2 \left(1 + \sum_{l=0}^{i} 2^l \sigma^l \right) d_0 | 0 \leq i \leq \log_2 m - 1 \right\}$ in a 2-D $m \times m$ grid, where d_0 is the estimated level density, and the total wirelength required to achieve the upper bound is $(2\alpha + \beta)(log_2 m) \cdot m^2 d_0$, where α is the percentage of diagonal combination nets and β is the percentage of adjacent combination nets.

In this paper, we focused on the theoretical analysis on routing estimation, where the propagation ratio of the candidate nets remained to be an unassigned parameter. Further research can be done with the propagation ratio. There can be a quick preprocessing before estimation, so that we can quickly check the distribution of all terminals on the given routing area and then give an appropriate value to the propagation ratio.

References

[Burstein 83] M. Burstein and R. Pelavin: "Hierarchical wire routing"; IEEE Transactions on Computer-Aided Design, 2(4), pp.223-234, October 1983.

[Cho 98] J. D. Cho and M. Sarrafzadeh: "Four-bend top-down global routing"; IEEE Transactions on Computer-Aided Design, 17(9), pp.793-802, September 1998.

[Cho 00] J. D. Cho: "Wiring space and length estimation in two-dimensional arrays"; IEEE Transactions on Computer-Aided Design, 19(5), pp. 612-615, May, 2000.

[Gamal 81] A.A. El Gamal and Z.A. Syed: "A stochastic model for interconnections in custom integrated circuits"; IEEE Transactions on Circuits & Systems, 28(9), pp. 888-894, 1981.

[Karp 87] R. M. Karp, F.T. Leighton, R. L. Rivest, C. D. Thompson, U. V. Vazirani and V. V. Vazirani: "Global wire routing in two-dimensional array"; Algorithmica, 2(1), pp. 113-129, 1987.

[Kuh 86] E. S. Kuh, and M. Marek-Sadowska: "Global routing"; in Layout Design and Verification. T. Ohtsuki, Ed., North-Holland, 1986.

[Li 84] J.-T. Li and M. Marek-Sadowska: "Global routing for gate arrays"; IEEE Transactions on Computer-Aided Design, 3(4), pp. 298-307, October 1984.

[Luk 87] W. K. Luk, P. Sipala, M. Tamminen, D. Tang, L. S. Woo, and C. K. Wong: "A hierarchical global wiring algorithm for custom chip design"; IEEE Transactions on Computer-Aided Design, Vol. 6, pp.518-533, July 1987.

[Sarrafzadeh 90] M. Sarrafzadeh and D. Zhou: "Global routing of short nets in two-dimensional arrays"; International Journal of Computer Aided VLSI Design, Vol. 2, pp. 197-211, 1990.

[Sherwani 95] N. Sherwani: Algorithms for VLSI Physical Design Automation. Kluwer Academic Publishers, 1995.

[Song 00] X. Song, Y. Tang, D. Zhou and Y. Wang: "Wire space estimation and routability analysis"; IEEE Transactions on Computer-Aided Design, 19(5), May 2000.

[Veccchi 83] M. P. Veccchi and S. Kirkpatrick: "GlobaI wiring by simulated annealing"; IEEE Transactions on Computer-Aided Design, 2(4), pp. 215-222, 1983.
